# Small-molecule binding-site discovery using silyl ether-enabled chemoproteomics

**DOI:** 10.1038/s41557-026-02127-4

**Published:** 2026-04-27

**Authors:** Chau Ngo, Sho Takechi, Aditya Sivakumar, Miranda Villanueva, Fengchao Yu, Andréa B. Ball, Javier Rubio, Elijah Biletch, Nikolas R. Burton, Lisa M. Boatner, Phillip Kim, Alexandra C. Turmon, Nithesh Perumal, Marc Liesa, Ajit S. Divakaruni, Alexey I. Nesvizhskii, Keriann M. Backus

**Affiliations:** 1 Department of Biological Chemistry, David Geffen School of Medicine, UCLA, Los Angeles, CA USA; 2 Department of Chemistry and Biochemistry, UCLA, Los Angeles, CA USA; 3 R&D Division, Daiichi Sankyo Co., Ltd, Tokyo, Japan; 4 Molecular Biology Institute, UCLA, Los Angeles, CA USA; 5 Department of Pathology, University of Michigan, Ann Arbor, MI USA; 6 Department of Molecular and Medical Pharmacology, David Geffen School of Medicine, UCLA, Los Angeles, CA USA; 7 Molecular Biology Institute of Barcelona, IBMB, CSIC, Barcelona, Spain; 8 CIBERDEM, Instituto de Salud Carlos III, Barcelona, Spain; 9 Gilbert S. Omenn Department of Computational Medicine and Bioinformatics, University of Michigan, Ann Arbor, MI USA; 10 DOE Institute for Genomics and Proteomics, UCLA, Los Angeles, CA USA; 11 Eli and Edythe Broad Center of Regenerative Medicine and Stem Cell Research, UCLA, Los Angeles, CA USA; 12 Jonsson Comprehensive Cancer Center, UCLA, Los Angeles, CA USA

**Keywords:** Target identification, Proteomics

## Abstract

For chemical probe and drug discovery campaigns, the pairing of mass spectrometry-based chemoproteomics with photoaffinity labelling has emerged as a favoured approach for target discovery and mode of action assignment. However, photocrosslinked peptide-compound adducts raise analytic challenges for quantitative binding site discovery. Here, to address these challenges, we establish the Silyl Ether Enables Chemoproteomic Interaction and Target Engagement (SEE-CITE) method. SEE-CITE incorporates a fully functionalized chemically cleavable photocrosslinking handle that enables precise site-of-labelling identification and head-to-head comparisons of relative binding site engagement by chemically diverse compounds. To ensure high-confidence localization of labelled residues, we extended the MSFragger algorithm of the FragPipe computational platform to report localization scores customized for photoaffinity labelling and SEE-CITE data. When applied to scout fragments and analogues of select FDA-approved kinase inhibitors, SEE-CITE delineates known drug binding sites and uncovers small-molecule binding sites that affect the protein activity of RTN4 and COX5A.

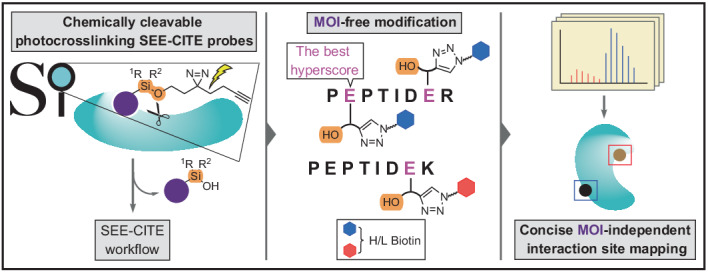

## Abstract (web summary)

Mapping binding sites using photoaffinity labelling (PAL) is highly valuable but challenging for probe and drug discovery campaigns. Now a chemoproteomic approach has been developed that incorporates cleavable silyl ether linkages into PAL probes to pinpoint and quantify the precise sites where small molecules engage protein binding partners.

## Main

**Fig. 1:**
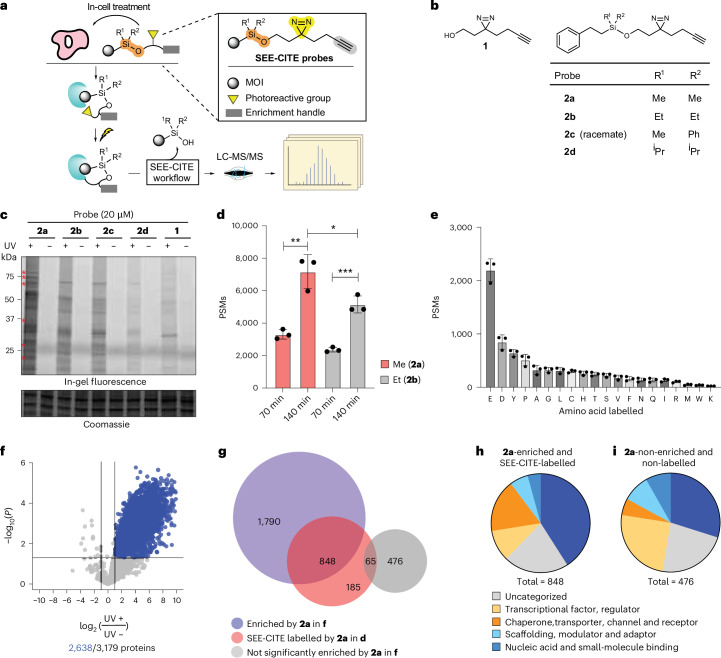
Establishing the SEE-CITE interaction site mapping platform using scout SEE-CITE probes. **a**, Overview of the SEE-CITE chemoproteomics workflow. **b**, Structures of minimalist compound **1** and first-generation scout probes **2a**–**2d**. **c**, Gel-based AfBPP analysis of K562 cells treated with the indicated probes (20 μM, 1 h). Representative data are presented for *n* = 2 independent experiments. **d**, Coverage comparison of SEE-CITE-modified PSMs identified for K562 cells labelled with **2a** (20 μM, 1 h) versus **2b** (20 μM, 1 h) (from left to right, *P* = 0.003476, 0.039729 and 0.000908). Data are the mean ± standard deviation (s.d.) calculated from *n* = 3 biological replicates. Statistical significance was calculated with a two-tailed unpaired Student’s *t*-test with *P < 0.05, **P < 0.01,***P < 0.001, and NS (not significant) *P* > 0.05. **e**, Distribution of identified M2-modified amino acids for **2a-**labelled samples from **d** (140-min gradient). Data are presented as mean values ± s.d. **f**, Protein-directed AfBPP analysis of K562 cells treated with **2a** (20 μM, 1 h) in a UV-dependent manner. The volcano plot displays a comparison between enriched proteins for ±UV-treated groups (*n* = 3 biological replicates for each group). Significant proteins were defined as log_2_(fold change) >1.0 and *P* < 0.05, determined from two-tailed unpaired Student’s *t*-tests. **g**, Overlapping SEE-CITE-labelled peptides by **2a** from **d** (140-min gradient) with protein-directed AfBPP from **f**. **h**,**i**, Protein classes of the enriched (**h**) versus non-enriched (**i**) proteins in ‘F’ with or without SEE-CITE-labelled peptides from **d**. All MS data can be found in Supplementary Table 2. Source data

**Extended Data Fig. 1:**
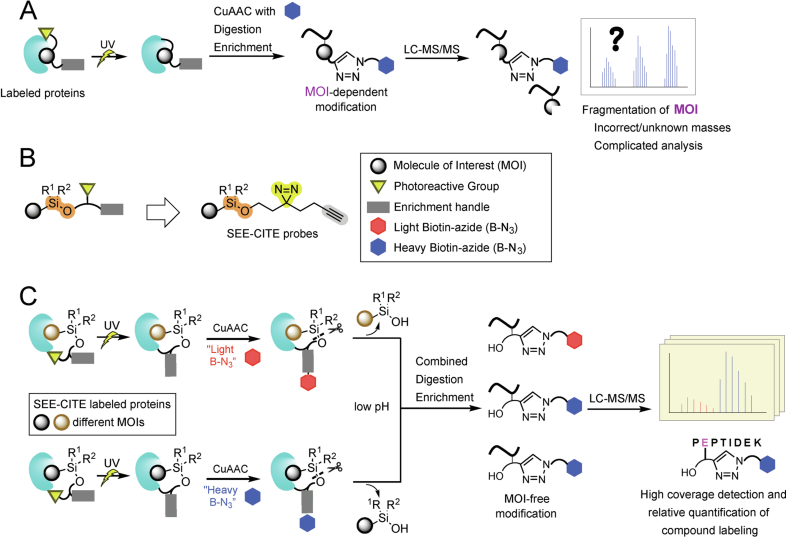
SEE-CITE photoaffinity labeling (PAL). **A**, Established PAL probes generate molecule-of-interest (MOI)-dependent modification of proteins, which can fragment in unanticipated ways, resulting in complex data analysis and lower coverage, particularly for large molecules. **B**, In SEE-CITE, MOIs are connected to diazirine handle **1** via a silyl ether linkage, which allows for straightforward removal of bulky molecules of interest (MOIs) from labeled peptides. **C**, As all SEE-CITE peptide modifications are the same mass, independent of the MOI mass, the relative labeling of different probes can be compared using isotopically enriched biotin-azide capture reagents, which provides a quantitative measure of structure-activity relationships (SAR) at specific binding sites identified.

Deciphering the protein targets, precise sites and modes of binding is a key aspect of most chemical probe and drug discovery campaigns, which is frequently addressed via photoaffinity labelling (PAL)^1–5^. To pinpoint interacting proteins via PAL, a molecule of interest (MOI; Fig. 1a) that has been functionalized with a photo-activatable moiety (for example, diazirine) is introduced to the sample of interest (for example, cells, lysates or purified protein), and the mixture is irradiated with ultraviolet (UV) light, which triggers the rapid release of highly reactive intermediates, such as a carbene^3^. These high-energy species react quickly and irreversibly with proximal proteins, which are then identified by mass spectrometry (MS)-based chemoproteomics^6,7^. PAL has been widely adopted for numerous applications, spanning protein–lipid^8–13^, protein–metabolite^14^, protein–natural products^15–18^, protein–glycan^19^, protein–cofactor^20^, protein–small molecule^4,5,21^ and even protein–drug interactions^22–26^. However, its use in site-of-interaction studies has been hindered by complex fragmentation of crosslinked molecules during tandem MS (MS/MS) analysis^12,27^ (Extended Data Fig. 1a). The use of smaller probes, often functionalized with ‘minimalized’ alkyne- and diazirine-containing alcohol **1** (Fig. 1b)^1^, together with efforts to improve MS/MS identification of modified peptides^5,28–30^, has made some inroads into the site-of-labelling bottleneck. However, coverage, fragmentation and accurate localization of crosslinking sites are still bottlenecks for most PAL workflows.

We report the Silyl Ether Enables Chemoproteomic Interaction and Target Engagement (SEE-CITE) method for site-centric PAL interactomics. SEE-CITE relies on chemically cleavable PAL probes (Fig. 1a and Extended Data Fig. 1a,b) that enable the facile release of bulky drug-like molecules from crosslinked peptide (Fig. 1a and Extended Data Fig. 1c). Implementation of this cleavage step improves peptide gas phase behaviour and enables pair-wise quantification of relative target engagement at specific binding sites. For the recently US Food and Drug Administration (FDA)-approved tyrosine kinase inhibitor asciminib (ABL001)^31,32^, application of the SEE-CITE-method yielded precise interaction sites, both for the known bioactive target (BCR-ABL1 myristoyl binding pocket) and for de novo discovery of additional targets of this anti-chronic myelogenous leukemia (CML) agent.

## Results

### Synthesis and evaluation of silyl ether prototype probes

**Extended Data Fig. 2:**
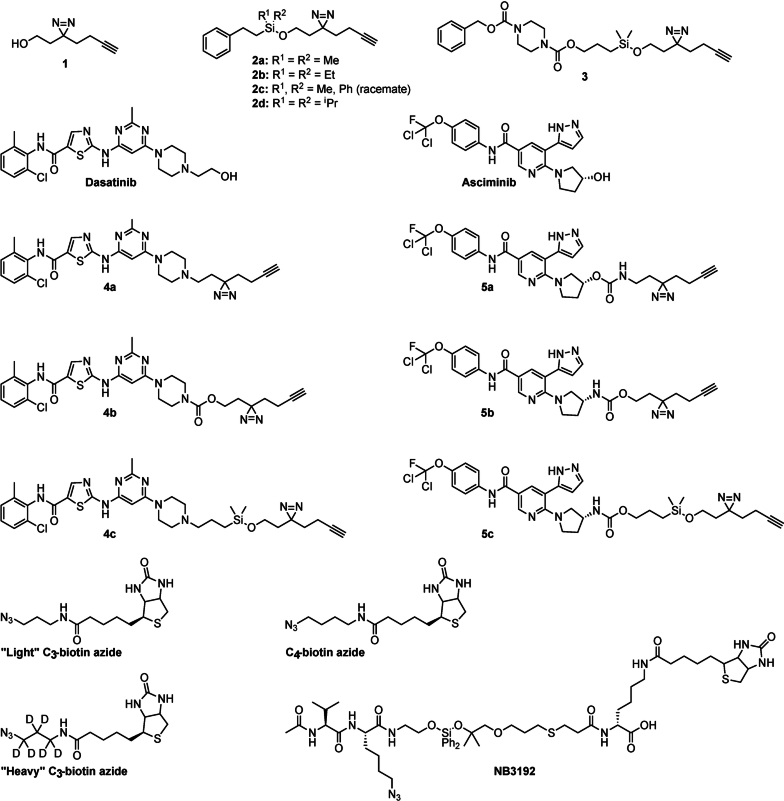
**Structure of all compounds used in the study**.

For our initial design of SEE-CITE probes, we drew inspiration from the proven utility of cleavable linkers, including the dialkoxydiphenylsilane (DADPS)-functionalized cleavable linkers, that enable the facile release of modified peptides in enrichment-based proteomic platforms^33–35^. To our knowledge, the use of DADPS or related silyl groups in photoaffinity probes to improve site of labelling analysis for PAL has yet to be reported. Therefore, our first step was to establish synthetic routes to prototype SEE-CITE probes with minimalist diazirine tag **1** (ref. ^1^). Scout probes **2a–d** were synthesized (Fig. 1b, Extended Data Fig. 2 and Supplementary Scheme 1a,b) and include all requisite elements, including an alkynyl click handle, diazirine for photocrosslinking, and silyl ether moieties functionalized with a range of R groups to assess the impact of substituents on relative protein labelling and modification release.

Compounds **2a–d** were observed to be stable to most conditions assessed, including aqueous buffer (room temperature for 24 h) and cell culture media (37 °C for 1 h); as expected, rapid and complete cleavage of the silyl ether moiety was observed for **2a–c**, with partial cleavage observed for the bulkier **2d**, under acidic conditions mimicking neutravidin elution conditions (Supplementary Fig. 1 and Supplementary Table 1). Gel-based affinity-based protein profiling (AfBPP)^3,4,9,36^ confirmed robust and UV-dependent protein labelling for **2a–d**, with clearly distinct banding patterns compared with **1** (Fig. 1c). As **1** is the expected release product upon silyl ether cleavage, this difference in banding indicates that the silyl ether in **2a**–**d** is not labile in cellulo. Increased protein labelling was observed for dimethyl probe **2a**, consistent with reduced steric hindrance favouring increased protein labelling.

### Achieving interaction site mapping with prototype probes

**Extended Data Fig. 3:**
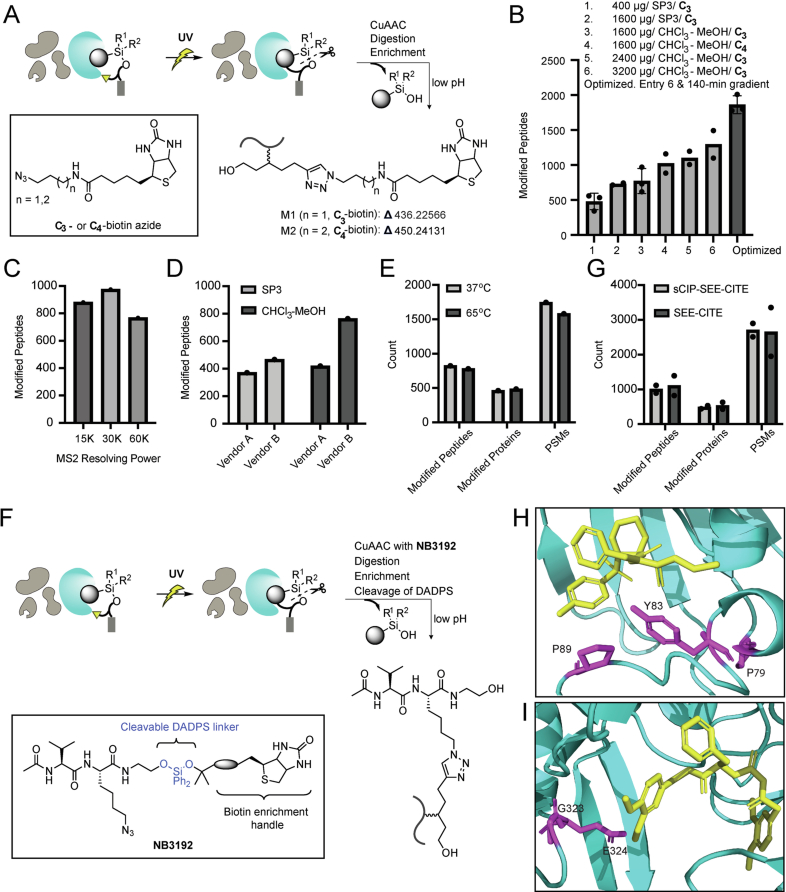
Coverage Improvement of SEE-CITE Workflow and Target Identification. **A**, SEE-CITE AfBPP workflow for comparing C3 and C4 biotin coverage. **B**, Assessing the impact of scaling protein input, sample cleanup by SP3 versus chloroform/methanol and extended gradient on coverage of SEE-CITE-modified peptides identified for 2b (20 μM, 1 h) in K562 cells. Data are mean ± SD (n = 3 biological replicates for entry 1, 3, and ‘optimized,’ n = 2 biological replicates for entry 2, 4-6). Best ‘optimized’ conditions reflect samples prepared using conditions from entry 6 with acquisition using a 140 min gradient. **C**, Optimization of MS2 resolving power (n = 1). The sample in entry 4, Fig. 2c, was used for this analysis. **D**, Comparison of different low-retention plastic containers, Vendor A (Fisher #02-681-320) and Vendor B (Sarstedt #72.706.600) (n = 1). **E**, Coverage comparison of SEE-CITE-labeled peptides undergoing DTT reduction at 37 °C (30 mins) versus 65 °C (15 mins) (n = 1). **F**, Alternative SEE-CITE workflow combined with a clickable biotin capture reagent, sCIP, with a chemically cleavable dialkoxydiphenylsilane (DADPS) linker. **G**, Coverage comparison of peptide-labeled by SEE-CITE with biotin-azide and SEE-CITE paired with sCIP reagent. Data presented are mean ± SD n = 2 biological replicates. **H**, **I**, Mapping sites of labeling by **2a (**20 μM, 1 h) in ‘D’ for ‘H’ human FKBP1A (PDB: 1J4H) and ‘I’ cathepsin D (PDB: 4OD9, (n = 2 biological replicates). All MS data can be found in Supplementary Table 2. Source data

We next evaluated peptide capture with **2a**–**d**. Adapting a single-pot solid-phase enhanced sample preparation (SP3) workflow^37–41^, we subjected K562 cells labelled with **2b** to SEE-CITE analysis, which identified peptides with the expected +436.22565 mass adduct **M1** (Fig. 1a and Extended Data Fig. 3a). However, coverage of labelled peptides was modest (entry 1 in Extended Data Fig. 3b). Alongside the known low diazirine crosslinking efficiency^3,42^, we speculated that incomplete reagent cleavage during sample preparation might also contribute to coverage. However, open search analysis^43–45^ revealed no modification masses for uncleaved species, which excluded this hypothesis (Supplementary Table 2). Sample scale-up (400 μg to 3,200 μg lysate) using chloroform–methanol precipitation^46^ did substantially improve coverage (Extended Data Fig. 3b), which also benefitted from several additional protocol modifications, including replacement of C_3_-biotin with C_4_-biotin^47^ (Extended Data Fig. 3b),)choice of low-retention sample containers^48^ (Extended Data Fig. 3d), acquisition with 30 K MS2 resolving power (Extended Data Fig. 3c), SEE-CITE analysis with more reactive dimethyl compound **2a** (Fig. 1d), and acquisition using a longer 140-min liquid chromatography (LC) gradient (Fig. 1d and Supplementary Tables 2 and 3). Additional efforts failed to further increase identifications (Extended Data Fig. 3e–g), and so, for subsequent experiments, we settled on these sample preparation and acquisition modifications, which together afforded an approximately fourfold net increase in coverage (Extended Data Fig. 3b).

### Protein targets and labelling sites captured by SEE-CITE probes

To evaluate the amino acids modified by **2a**, we next analysed the localization site reported by variable modification search in FragPipe^28,43,45^, which showed preferential labelling acidic residues (Fig. 1e and Supplementary Table 2) consistent with prior findings^49^. To both confirm the protein targets identified by SEE-CITE and assess how reliance on peptide-level identification impacts protein coverage for SEE-CITE, we next compared the proteins identified by SEE-CITE (based on **M2**-modified peptides; Fig. 1d) with those captured by **2a** using a protein-directed AfBPP proteomic workflow^50^ (Fig. 1f). Nearly 80% of the 1,098 total SEE-CITE captured proteins were also enriched in our protein-directed dataset, which corroborates the complementarity of both methods (Fig. 1g), and **2a** binding sites were identified for 32% of the **2a**-enriched proteins (Fig. 1g). Protein class analysis revealed a slight increase in enzymes and a marked decrease in transcription factors (TFs) enriched by scout probe **2a**, consistent with the general paucity of small-molecule binding pockets in TFs (Fig. 1h,i). The presence of **2a**-labelled residues proximal to known co-crystallized inhibitors, including in FKBP prolyl isomerase 1A and cathepsin-D (Extended Data Fig. 3h,i) confirmed that the SEE-CITE scout probe can identify known small-molecule binding sites.

### Enhancing PAL data analysis in FragPipe

One limitation of our initial, variable modification, MSFragger search (Fig. 1) is that, for peptide–spectrum matches (PSMs) lacking sufficient sequence coverage for a crosslinked modification to be placed on a single residue, the modification is placed on the first amino acid within a stretch of equally scoring sites. This limitation, which may reduce the accuracy of localization of crosslinking sites, is exemplified by the **2a**-modified peptide AYLESEVAISEELVQK from reticulon-4 (RTN4). For this peptide, the modification is placed on the first residue, alanine, despite there being no detectable shifted or unshifted b or y ions between the 1A and adjacent 2Y residue (Supplementary Fig. 2a).

**Fig. 2:**
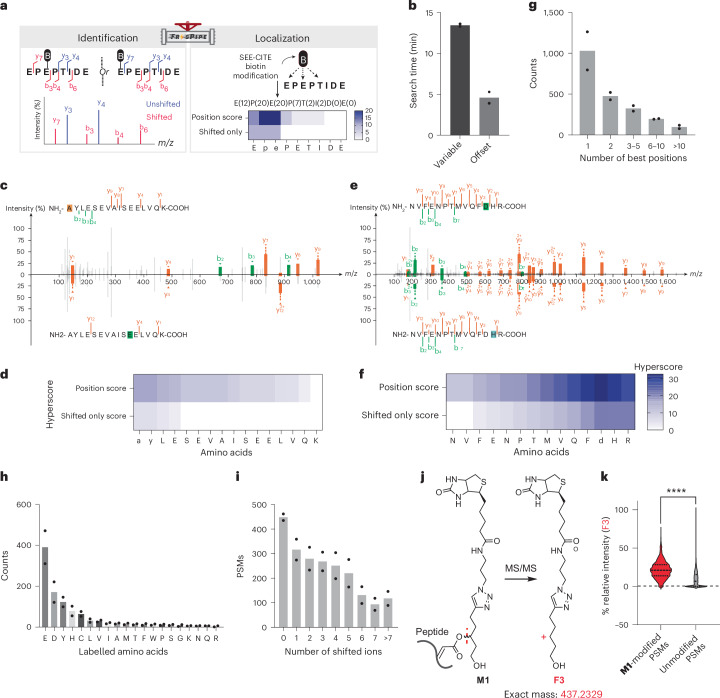
Establishing scores in MSFragger and FragPipe to assess localization of SEE-CITE modifications. **a**, Workflow shows how MSFragger, in mass offset search mode, calculates multiple scores assessing the number, positions, and quality of SEE-CITE modified (shifted) and unshifted fragment ions detected for PAL-modified PSMs. **b**, Comparison of search times using mass offset versus variable modification search mode of MSFragger (shown is total FragPipe run time). **c**, Example SEE-CITE-modified spectra for ayLESEVAISEELVQK from RTN4 comparing localization on 1A (top) to manual placement of the modification on 11E (bottom). **d**, The MSFragger hyperscore calculated using both shifted and unshifted ions (top) and only shifted ions (bottom) for peptide shown in **d**. **e**, Example SEE-CITE-modified spectra for NVFENPTMVQFdHR peptide from the COX15 subunit of cytochrome C oxidase comparing hyperscore with localization on 11D (top) to manual placement of the modification on 12H (bottom), with **f**, showing hyperscore calculated using both shifted and unshifted ions (top) and only shifted ions (bottom) for peptide show in **f**. **g**, Distribution of best positions with identical hyperscores for PSMs with SEE-CITE modifications. **h**, Distribution of labelled amino acids identified for PSMs with single best positions as identified in **h**. **i**, Histogram of number of shifted ions detected for PSMs with SEE-CITE modifications. **j**, Predicted fragmentation consistent with the absence of shifted ions. **k**, Relative intensity of signature fragment ion (*m*/*z* +437) for SEE-CITE-modified versus unmodified PSMs. The significance value (*P* < 0.000001) was determined by comparing all modified PSMs with unmodified PSMs. For **d** and **f**, lowercase letters show the positions with the best hyperscores. For **b**, **g**, **h** and **i**, mean ± standard deviation is shown for reanalysis of the duplicate experiments from Extended Data Fig. 3b, entry 6. All MS data can be found in Supplementary Table 4. Source data

**Extended Data Fig. 4:**
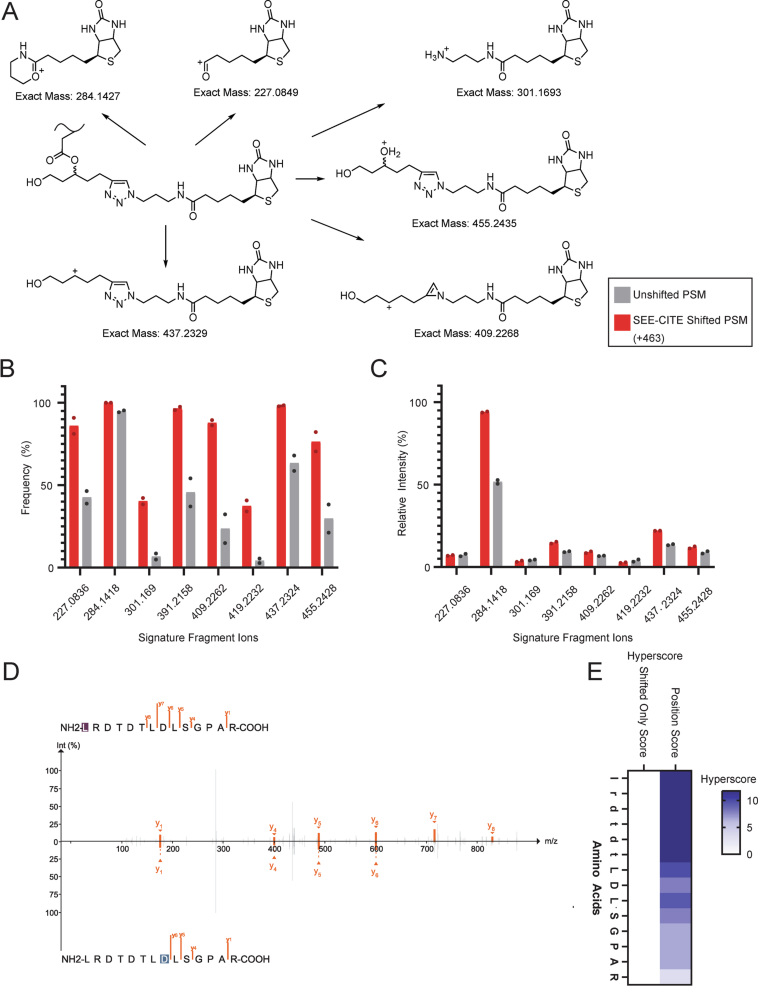
FragPipe diagnostic ion miningcoupled with MSFragger mass offset search identify signature fragment ions for the SEE-CITE modification. **A**, Shows proposed structures of signature fragment ions with exact masses corresponding to the m/z values identified by diagnostic ion mining for entry 6 from Fig. 2c. **B**, Frequency distribution and **C**, relative intensities of signature fragment ions. **D**, The presence of unshifted ions for the C-terminal half of the peptide enables partial localization of the SEE-CITE modification to the N-terminal ‘lrdtdt’ portion of the peptide, as illustrated by the lower spectra, where the modification was manually placed on the 8D residue (bottom) rather than the 1 L residue (top), which results in a decreased number of matched unshifted Y ions, with loss of the Y_7_ and Y_8_ fragments. **E**, shows hyperscores derived from unshifted ions for the lrdtdtLDLSGPAR peptide DHX16, for which no shifted ions were matched. Experiments were performed in duplicate. All data can be found in Supplementary Table 4. Source data

To address this problem, we established a tailored PAL-analysis workflow in FragPipe (labelled ‘PAL’ workflow in FragPipe 22 release; Supplementary Note 1) that builds on our mass offset search workflow^51^ and is generally compatible with PAL probes, including the SEE-CITE probes. This PAL workflow (Fig. 2a) introduces a set of MSFragger localization scores (hyperscores) tailored for more accurate modification localization together with customization of the MSBooster^52^ rescoring tool for PAL, by improving the use of modified peptide retention times. Reanalysis of datasets from Fig. 1 with this new mass offset workflow revealed a markedly reduced processing time and a slight (~10%) (Fig. 2b, Supplementary Fig. 2b and Supplementary Note 1). This scoring approach also accurately localized modifications as illustrated by case study peptides from RTN4 and COX5A (Fig. 2c–f). For both of these peptides, manual placement of modifications on lower-scoring residues using the PDV viewer^53^, corroborating the FragPipe-identified labelling site and confirming the likelihood of the assigned modification site (Supplementary Note 2). We next parsed our reprocessed data from Fig. 1d (Supplementary Fig. 2b,c, Supplementary Fig. 3a,b and Supplementary Note 3). We found that the labelling site could be localized to a single amino acid for 48% of PSMs (Fig. 2g), and that 55% of all localized residues are glutamate or aspartate residues and a further 25% of modifications localize to either tyrosine, histidine or cysteine (Fig. 2h). Notably this bias towards acidic and more nucleophilic residues is increased when compared with our initial analysis (Fig. 1e). Unexpectedly, for ~20% of all spectra, no shifted ions were observed (Fig. 2i). We hypothesized that loss of the complete PAL adduct to release **M1** could explain this observation (Fig. 2j); similar fragmentation has been previously reported for peptides modified by diazirine-based chemical crosslinkers^54^. This hypothesis was substantiated by diagnostic ion mining^29^, which identified the comparatively high intensity and specificity 437 *m*/*z* ion, which is consistent with loss of **M1** (Fig. 2k and Extended Data Fig. 4a–c). Importantly, using the hyperscore approach, for these spectra that lack shifted ions, the sequence region of likely localization can still be inferred from the scores of the unshifted ions, as illustrated for the example peptide from RNA helicase DHX16 (Extended Data Fig. 4d,e).

### Using SEE-CITE for advanced scout fragments and drugs

**Extended Data Fig. 5:**
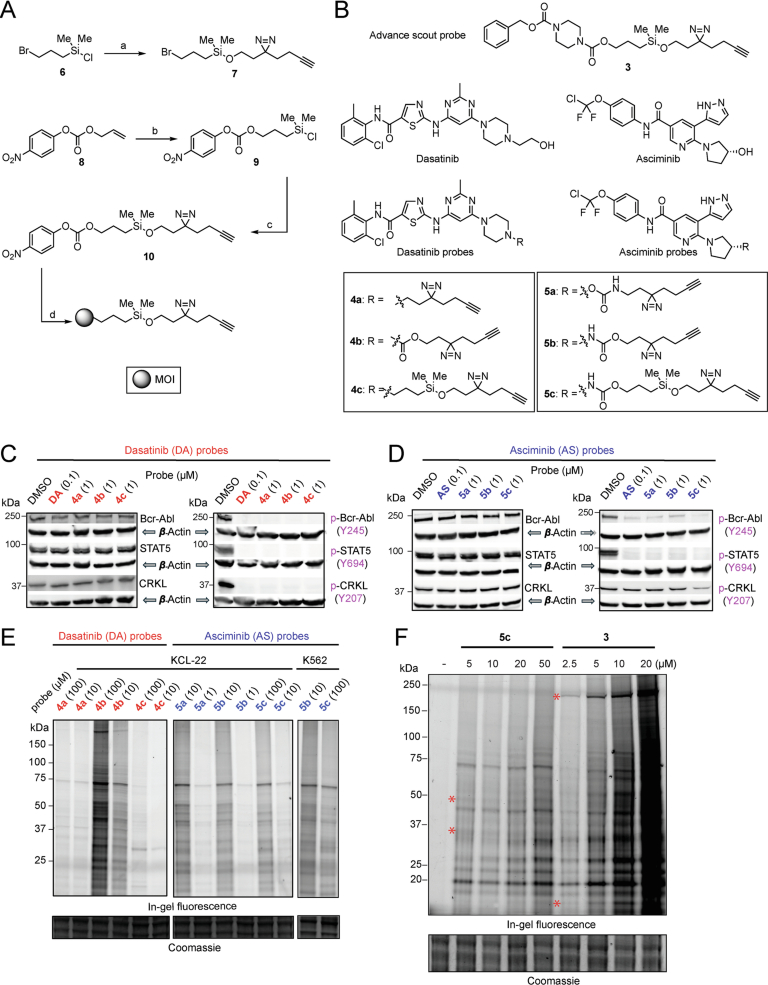
Synthesis and assessment of cell-based labeling and kinase inhibitory activity of SEE-CITE probes. **A**, Design of the dasatinib and asciminib photoaffinity probes. **B**, Synthesis of the silyl ether tags. Reagents and conditions: a. **1**, NEt_3_, CH_2_Cl_2_, 0 °C, 75%; b. chlorodimethylsilane, 1,5-cyclooctadiene, [Ir(cod)Cl]_2_(0.01 mol%), 75-80 °C, 96%; c. **1**, NEt_3_, CH_2_Cl_2_, 0 °C, 81%. **C,D**, Immunoblot analysis of KCL22 cells treated for 2 h with either dasatinib probes (**4a**-**4c**) and asciminib probes (**5a**-**5c**) at the indicated concentrations. **E,F**, Gel-based AfBPP analysis comparing PAL-labeling using various asciminib and dasatinib probes at the indicated concentrations for 1 h. Data presented are from n = 1 (**C**-**E**) and n = 2 (**F**) independent experiment(s).

We next focused on expanding the SEE-CITE methodology to more structurally complex MOIs. We first piloted our synthetic route on the more advanced scout probe **3** (Extended Data Fig. 5a,b). Gel-based analysis confirmed different protein labelling patterns for scout probes **2a** and **3**, consistent with each probe labelling distinct proteins (Supplementary Fig. 4a).

**Fig. 3:**
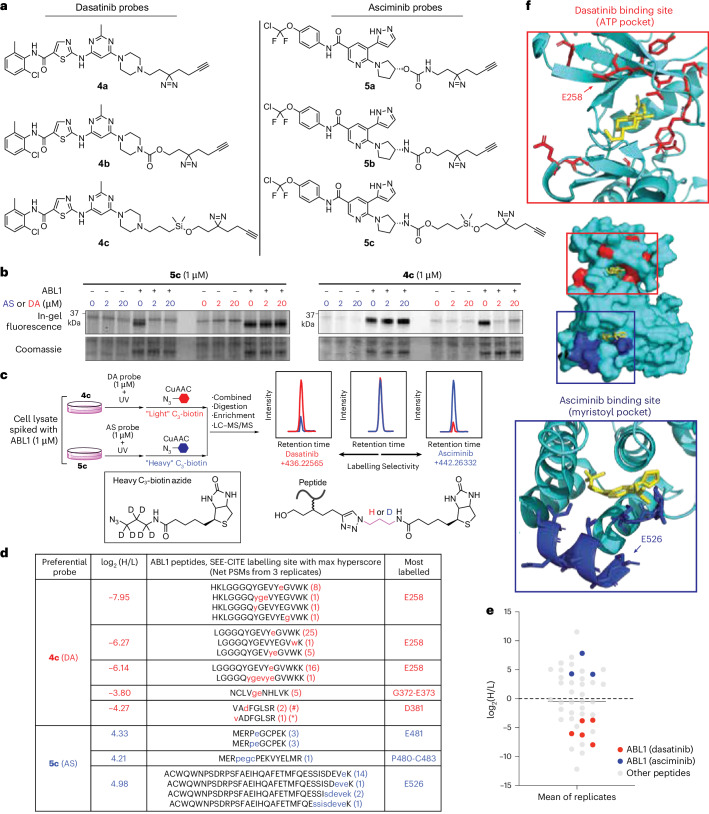
SEE-CITE mapping of ABL1 binding sites. **a**, Structures of dasatinib and asciminib probes. **b**, Competitive gel-based AfBPP analysis of recombinant ABL1 kinase domain (1 μM) in HEK293T lysates (0.5 mg ml^−1^) pretreated with dasatinib or asciminib (0.5 h) followed by **4c** or **5c** probe treatment (1 μM, 0.5 h). Representative data are presented for *n* = 3 independent experiments. **c**, Workflow for quantitative SEE-CITE comparing **4c** and **5c** labelling of ABL1 kinase domain (1 μM) in lysates treated with **4c** or **5c** (1 μM, 0.5 h). **d**, Mean log_2_(H/L) ratios generated via IonQuant analysis with FragPipe variable search workflow and with SEE-CITE-based sites of labelling (lowercase letters) using FragPipe mass offset search workflow. *, peptide identified using MSBooster and Percolator without deisotoping in MSFragger; #, peptide identified using PeptideProphet with de-isotoping in MSFragger. **e**, Comparison of log_2_(H/L) ratios of SEE-CITE-modified ABL1 peptides (red/blue) versus unmodified peptides (grey) from *n* = 3 biological replicates. **f**, Identified labelled sites for the ABL1 kinase domain by SEE-CITE probes **4c** and **5c** (PDB: 2GQG for ATP pocket and 5MO4 for myristoyl pocket, respectively). All MS data can be found in Supplementary Table 5. Source data

Alongside **3**, we selected dasatinib and asciminib as ideal MOIs for proof-of-concept SEE-CITE studies with biomedical relevance (Fig. 3a). Both are FDA-approved and highly efficacious drugs that treat CML by inhibiting the BCR-ABL1 oncogenic kinase, with distinct modes of action. Dasatinib is an ATP-competitive type I kinase inhibitor^55^, while asciminib is an allosteric inhibitor that functions through a Specifically Targeting the Abl Myristoyl Pocket (STAMP) mode of inhibition^31,32,56^. The off-targets of dasatinib have been widely characterized, including by chemoproteomics^22,57,58^, and include other Src family kinases. As asciminib only recently received FDA approval in 2021, its off-targets remain less characterized.

We synthesized probes based on known solvent-accessible positions and tractable synthetic routes (Fig. 3a, Extended Data Fig. 5a,b, Supplementary Schemes 2 and 3, Supplementary Fig. 5 and Supplementary Note 4). Both conventional PAL probes (**4a** and **4b** for dasatinib and **5a** and **5b** for asciminib) that lack the SEE-CITE group, to serve as benchmarking probes, and SEE-CITE probes (**4c** and **5c**) were obtained in moderate yield (Supplementary Schemes 2 and 3).

### SEE-CITE probes inhibit and label BCR-ABL1

We next confirmed that our probes retained inhibitory activity in cellulo. Immunoblot analysis of compound-treated KCL-22 CML cells revealed robust inhibition of BCR-ABL1 activity, with dasatinib **4a–c** and asciminib **5a–c** probes abolishing BCR-ABL1 autophosphorylation and STAT5 phosphorylation at Y694 (Extended Data Fig. 5c,d). The dasatinib probes also blocked phosphorylation of substrate CRKL at Y207, whereas the asciminib analogues did not, consistent with prior reports^31^.

Cell-based AfBPP labelling studies further confirmed photocrosslinking and indicated some probe-specific differences in overall labelling intensities and banding patterns (Extended Data Fig. 5e and Supplementary Note 5). Recombinant ABL1 was robustly labelled by parent PAL probes **4b** and **5b** and SEE-CITE probes **4c** and **5b**, with labelling fully off-competed by excess parent compound (Fig. 3b and Supplementary Fig. 4b,c). These findings align with specific PAL at binding sites occupied by parent inhibitors and, more broadly, indicate that the SEE-CITE modifications should be generally tolerated, although the bulkier modification may impact overall activity.

### Quantification of ABL1 binding site engagement by SEE-CITE

To quantify relative probe engagement to active versus allosteric (STAMP) binding sites, we subjected recombinant ABL1 to SEE-CITE analysis following the workflow shown in Fig. 3c with differentiation **4c** and **5c** using light- and heavy-biotin azide reagents^47^. The relative labelling individual ABL1 peptides by each compound was then determined using MS1 intensity ratios (Heavy/Light; Supplementary Table 5), with negative H/L ratios indicating preferential dasatinib binding and positive ratios favouring asciminib binding. Both the variable modification workflow (Supplementary Fig. 6a–c) and mass offset search workflow (Fig. 3d–f) accurately delineated each probe’s known binding site (Supplementary Fig. 6a–c, Fig. 3d–f and Supplementary Note 6). While some modified residues were located immediately adjacent to the probe binding sites, such as E421 (3.4 Å), other residues were slightly more distant, such as E253 (~10 Å). We expect that these differences in proximity stem from a confluence of factors, including dynamic binding poses, the relatively flexible and longer SEE-CITE linker, and possibly some unavoidable remaining ambiguity in the assigned modification sites. Despite these nuances, the ABL1 labelling study provides a clear case for accurate delineation of small-molecule binding sites and models how SEE-CITE can integrate into follow-up workflows to enable binding site identification after an initial target is nominated.

### Protein-directed AfBPP reveals candidate asciminib targets

**Fig. 4:**
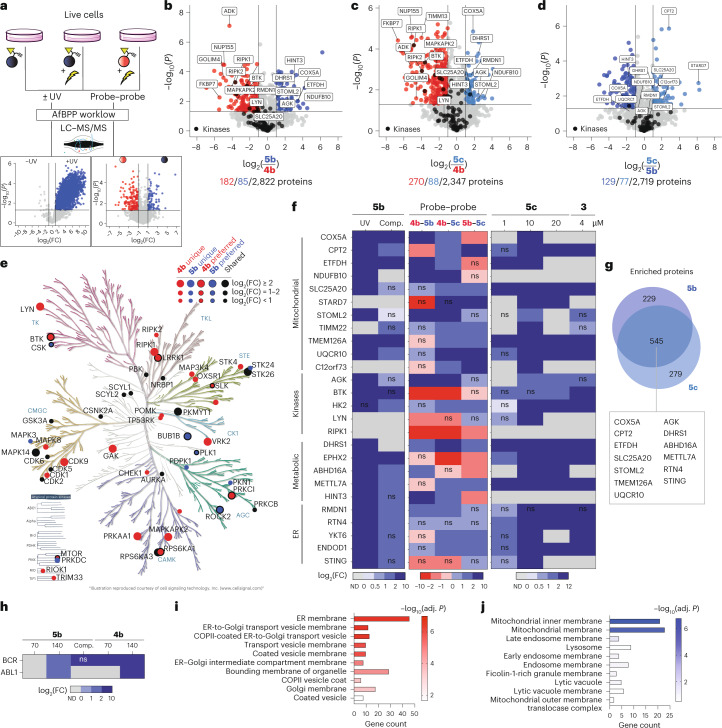
Interactome analysis of asciminib using protein-directed AfBPP. **a**, Workflow for protein-level directed AfBPP. **b**–**d**, Probe–probe AfBPP comparison of K562 cells treated for 1 h with **4b** (10 μM) versus **5b** (10 μM) (**b**), **4b** (10 μM) versus **5c** (100 μM) (**c**) and **5b** (10 μM) versus **5c** (100 μM) (**d**). The **5c** probe was screened at a higher concentration to match general protein labelling. Volcano plots display comparisons between enriched proteins from three groups with red representing **4b**-labelled proteins, darker blue representing **5b**-labelled proteins and lighter blue representing **5c**-labelled proteins (*n* = 3 biological replicates for each group per experiment). Black coloured dots represent labelled kinases. **e**, Kinome tree annotation^78^ for data in **b**. **f**, Heatmaps of high-value targets in **b** and in K562 cells labelled with **5b** (10 μM), **5c** (1, 10 and 20 μM), or **3** (4 μM) in a UV-dependent manner (1 h) or in a competitive manner with asciminib pretreatment (50 μM, 0.5 h) followed by 0.5-h-probe treatment. **g**, Overlap of proteins significantly enriched in a UV-dependent manner for **5b** (10 μM) and **5c** (100 μM) in K562 cells. **h**, Heatmap of BCR and ABL1 in KCL-22 cells labelled with **4b** (100 μM, 1 h) and **5b** (10 μM, 1 h) in a UV-dependent or in competitive manner with asciminib pretreatment (50 μM, 0.5 h). **i**,**j**, GO Cellular Component analysis for enriched proteins in **b** captured by **4b** (**i**) and **5b** in (**j**). Significance was calculated with Fisher’s exact test, and adjusted *P* values were derived using the Benjamini–Hochberg method. For probe–probe analyses in **b**–**d** and **f** and the UV-dependent labelling experiment in **f**, significant proteins were defined as log_2_(FC) >1.0 and *P* < 0.05 determined from two-tailed unpaired Student’s *t*-tests. For competitive analysis in **f**, significant proteins were defined as log_2_(FC) >0.5 and *P* < 0.05. Comp., competition; NS, not significant; ND, not detected; FC, fold change. All MS data can be found in Supplementary Table 6.

To identify proteins preferentially engaged by asciminib and to generate curated protein-level datasets to leverage for target prioritization during our planned subsequent SEE-CITE site-level labelling studies, we next turned to AfBPP proteomic analysis. We first generated probe–probe comparisons of **4b**, **5b** and **5c** (Fig. 4a–d and Supplementary Table 6). These comparisons revealed clearly distinct target profiles for each probe (Fig. 4b). Consistent with the active-site directed activity of dasatinib, we observed a clear trend towards increased capture of kinases by **4b** relative to both **5b** and **5c**, including previously reported off-targets of dasatinib such as BTK, CSK, LYN and RIPK2^57–59^ (Fig. 4b,c,e and Supplementary Tables 7 and 8). While most kinases favoured the dasatinib probe **4b**, a handful showed preferred labelling by one or more of the asciminib probes, including AGK, PKN1 and PDPK1 (Fig. 4e), which indicates that, while asciminib’s non-active site directed mode of action clearly minimizes kinase off-targets, asciminib still may bind to a handful of cellular kinases. Looking beyond kinases, many proteins (for example ADK, TIMM13, FKBP7 and Unc119b) showed favoured capture by **4b** when compared with both asciminib probes, indicating that probes **5b** and **5c** probably engage similar proteins.

For asciminib-specific binders, we were intrigued by the strong labelling of several mitochondrial proteins, including electron transfer protein ETFDH and the complex IV subunit COX5A, by both **5b** and **5c** relative to **4b** (Fig. 4b–d,f). While most of their targets were shared (Fig. 4g), we were intrigued by a handful of proteins that showed differential selectivity between **5b** and **5c**, including lipid transport protein STARD7 that favoured labelling by **4b** and **5c**, relative to **5b**, and mitochondrial-membrane carrier protein SLC25A20, which is a reported target of ingenol^17^ that favoured labelling by **5c** (Fig. 4f,g). Therefore, we conclude that a subset of proteins identified, such as COX5A, likely are asciminib specific binders, whereas others, such as STARD7 and SLC25A20, exhibit labelling suggestive of possibly multiple binding sites or preferential engagement to the SEE-CITE moiety.

Unexpectedly, while BCR was detected in our **5b** dataset and highly enriched by **4b** in our initial probe-probe datasets, ABL1 was not, which also highlighted a need to further improve our coverage. Therefore, we next acquired additional datasets modifying the gradient length. This improved coverage enabled us to detect ABL1 as enriched by both the asciminib and dasatinib probes corroborating the on-target activity of our probes (Fig. 4h).

**Extended Data Fig. 6:**
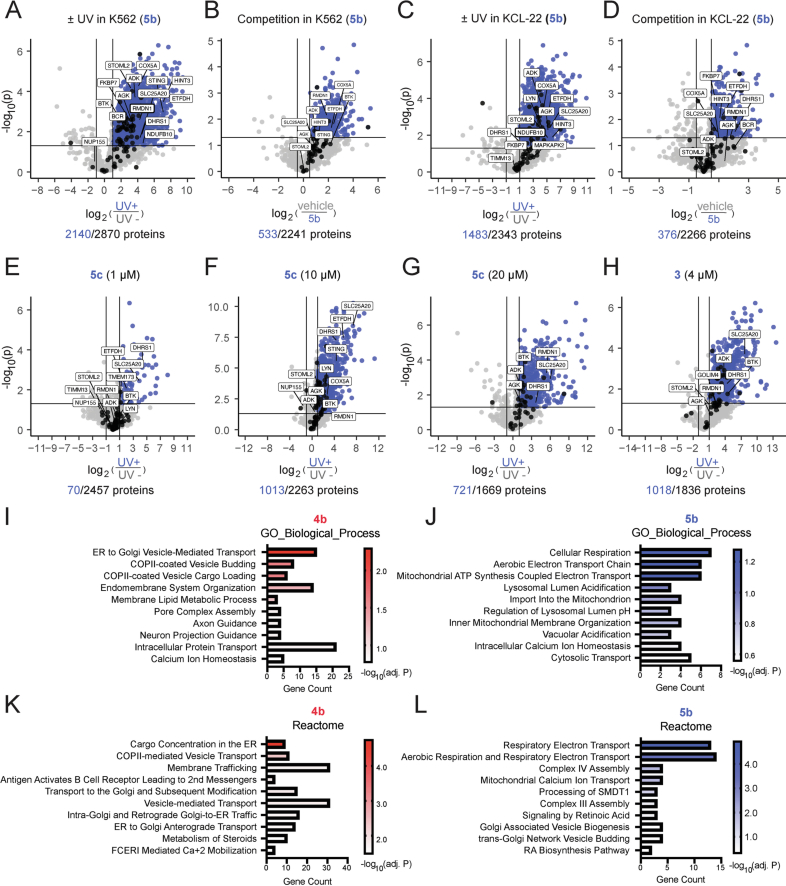
Interactome and pathway analyses with protein-directed AfBPP. **A-D**, Analyses of protein abundance changes in K562 cells (70-min gradient) for ‘**A**,**B**’ and in KCL22 cells for ‘**C**,**D**’ either treated with **5b** (10 μM, 1 h) in a UV-dependent ‘**A**,**C**’ or with **5b** (10 μM, 0.5 h) in a competitive manner ‘**B**,**D**’ including asciminib pretreatment (50 μM, 0.5 h). **E-H**, Analyses of protein abundance changes in K562 cells (70-min gradient) treated with **5c** (1, 10 or 20 μM, 1 h) in ‘**E**-**G**’ or **3** (4 μM, 1 h) in ‘**H**’ in a UV-dependent manner. All volcano plots display comparisons between enriched proteins from the treated groups indicated with blue colored dots being significantly enriched proteins and black colored dots being labeled kinases. For UV-dependent labeling experiments in ‘**A**’, ‘**C**’, **E**-**H**’, significant proteins were defined as log2(FC) > 1.0 and p < 0.05. For competitive analysis in ‘**B**’,‘**D**’, significant proteins were defined as log2(FC) > 0.5 and p < 0.05. P values are determined from two tailed unpaired student’s t-tests. **I**-**L**, GO Biological Process and Reactome analyses for enriched proteins in ‘Fig. 4b’ captured by **4b** in ‘**I** and **K**’ and **5b** in ‘**J** and **L**’. Pathway groups with an adjusted *P* value (adj. P) < 0.05 were considered significantly enriched over the background. Significance was calculated with Fisher’s exact test, and adjusted p values were derived using Benjamini-Hochberg method. All MS data can be found in Supplementary Table 6.

To further narrow the list of likely asciminib binders, we generated a suite of additional complementary datasets, including light-dependent and off-competition datasets in two cell lines, dose-dependent labelling and comparisons to scout probe **3**, with the latter aimed at delineating targets that favour the silyl ether linker (Fig. 4f,h and Extended Data Fig. 6a–h). Excess asciminib off-competed **5b** labelling for most of the previously noted candidate targets, corroborating high-occupancy labelling. Several proteins, including STARD7, STING and, most notably, COX5A, were enriched by **5c** and not by **3** (Fig. 4f and Extended Data Fig. 6e–h), providing further evidence of specificity. Some proteins, including the previously noted **2a**-labelled RTN4 and stimulator of interferon genes (STING), showed strong enrichment by all probes, albeit slightly favouring the asciminib probes. As this activity suggested the possibility that probe localization could contribute to differences in protein targets, we assessed Gene Ontology (GO) and Reactome enrichment for our probe–probe comparisons, which confirmed mitochondrial-preferred labelling for **5b** and endoplasmic reticulum (ER)-preferred labelling for **4b** (Fig. 4i,j and Extended Data Fig. 6i–l). Taken together, these AfBPP analyses nominate COX5A as a bona fide asciminib-specific binder and highlight some challenges for AfBPP-based target discovery, including the likelihood of multiple binding sites complicating target discovery.

### Proteome-wide quantitative SEE-CITE binding site analysis

**Fig. 5:**
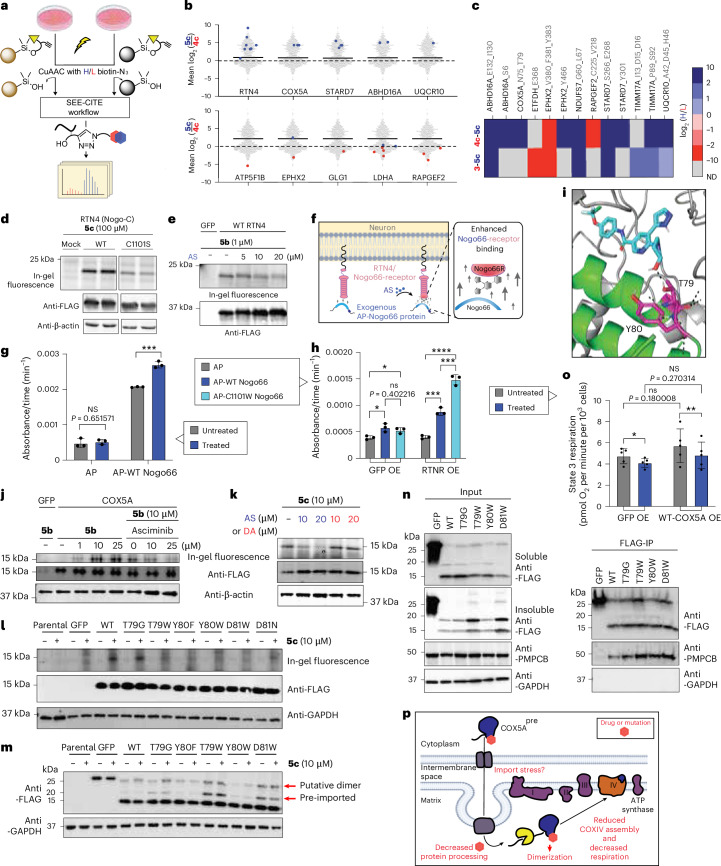
SEE-CITE and functional studies confirm activity and labelling sites for asciminib binders. **a**, General SEE-CITE-based AfBPP workflow. **b**, Scatter plots of peptides labelled by **4c** (100 μM, 1 h) and **5c** (100 μM, 1 h) in K562 cells. Data are average log_2_(H/L) values (*n* = 2 biological replicates) including singletons imputed using H/L ratio of 20. Coloured dots indicate probe-modified peptides for selected high-value targets. **c**, Heatmap comparing average log_2_(H/L) values for SEE-CITE-identified sites of labelling for selected targets in **b** and those in K562 cells labelled with **5c** (20 μM, 1 h) or **3** (4 μM, 1 h). **d**,**e**, In-gel fluorescence analyses of HEK293T cells overexpressing wild-type (WT) RTN4, mutant C1101S-RTN4 labelled with **5c** (100 μM, 1 h) (**d**) or in a competitive manner with asciminib (0.5 h) and **5b** (1 μM, 0.5 h) (**e**) with GFP-HEK293T cell line as a control. **f**, A schematic representation of NOGO66–RTNR interaction, assayed with AP fusion proteins. **g**,**h**, Quantification of Nogo66–RTNR interaction via AP binding assays in HEK293T cells stably overexpressing RTNR incubated with AP fusion proteins pre-treated with asciminib (10 μM, 15 min) (**g**) or overexpressing GFP or RTNR incubated with AP, wild-type (WT) Nogo66 or C1101W-Nogo66 proteins (**h**). **i**, Docking of asciminib into the SEE-CITE COX5A labelling region (T79-Y82). **j**–**l**, In-gel fluorescence analyses of HEK293T cells transiently overexpressing FLAG-tagged GFP, wild-type COX5A or mutant COX5A in a competitive manner with asciminib and/or dasatinib (1 h) and **5b** (**j**) or **5c** (**k**) (10 μM, 1 h) or in a UV-dependent manner with **5c** (10 μM, 1 h) (**l**). **m**, Immunoblot analysis of HEK293T cells transiently overexpressing FLAG-tagged GFP or COX5A (WT and mutants). **n**, FLAG immunoprecipitation (IP) with HEK293T transiently overexpressing FLAG-tagged GFP or COX5A (WT and mutants). **o**, State 3 respiration in plasma membrane-permeabilized HeLa cells stably overexpressing GFP–HA or COX5A–HA with or without asciminib treatment (10 μM, 4 h). Cells were offered pyruvate/malate as respiratory substrates. **p**, A schematic representation depicting effects of asciminib on COX5A and mitochondria functions. For **g**, **h** and **o**, data presented are mean ± s.d., with *P* values calculated using two-tailed unpaired and paired Student’s *t*-tests, respectively, using *n* = 3 biological replicates (**g** and **h**) and *n* = 6 biological replicates (**o**) (from left to right: *P* = 0.651571 and 0.000313 (**g**); *P* = 0.019546, 0.402216, 0.018749, 0.000398, 0.000959 and 0.000051 (**h**); *P* = 0.0461, 0.180008, 0.270314 and 0.007380 (**o**)) with *P < 0.05, **P < 0.01,***P < 0.001, and NS (not significant) *P* > 0.05. For **d**, **e** and **j**–**m**, data are presented for *n* = 3 independent experiments. NS, not significant; WT, wild type; OE, overexpression. All MS data can be found in Supplementary Table 9. Illustrations in **a, f** and **p** created in BioRender; Ngo, C. https://biorender.com/bxsja9h (2026). Source data

**Extended Data Fig. 7:**
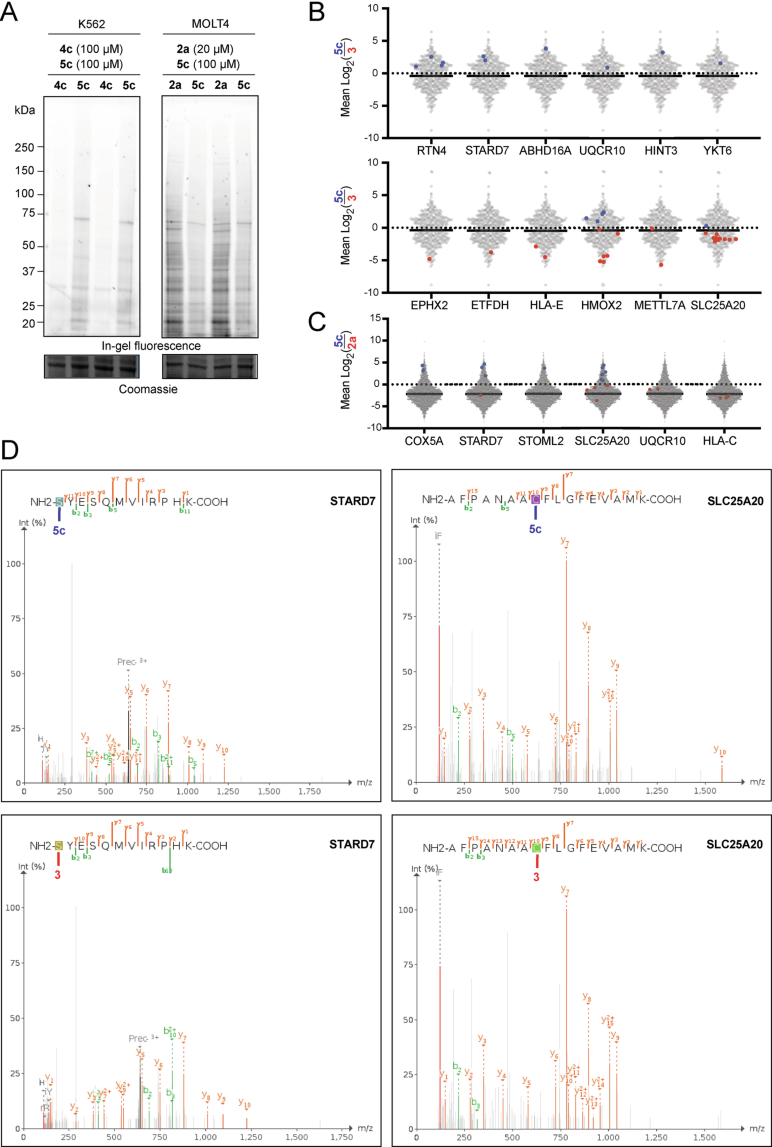
Establishing proteome-wide SEE-CITE. **A**, Gel-based AfBPP analysis of K562 and MOLT4 cells labeled by **4c**/**5c** or **2a**/**5c**, respectively, at the indicated concentrations for 1 h. Data presented are from n = 1 independent experiment. **B,C**, Measured SEE-CITE ratios for modified peptides for ‘B’ in K562 cells treated with **3** (4 μM) and **5c** (20 μM) for 1 h and ‘C’ MOLT4 cells treated with **2a** (20 μM) and **5c** (100 μM) for 1 hr. Data are average log2(H/L) values (n = 2 biological replicates) with singleton (heavy- or light-only modified peptides) set to log2(H/L) = 4.312. Colored dots indicate probe-modified peptides for selected high-value targets. **D**, representative spectra from datasets shown in ‘Extended Data Fig. 7B.’ Spectra generated in PDV. All MS data can be found in Supplementary Table 9.

**Extended Data Fig. 8:**
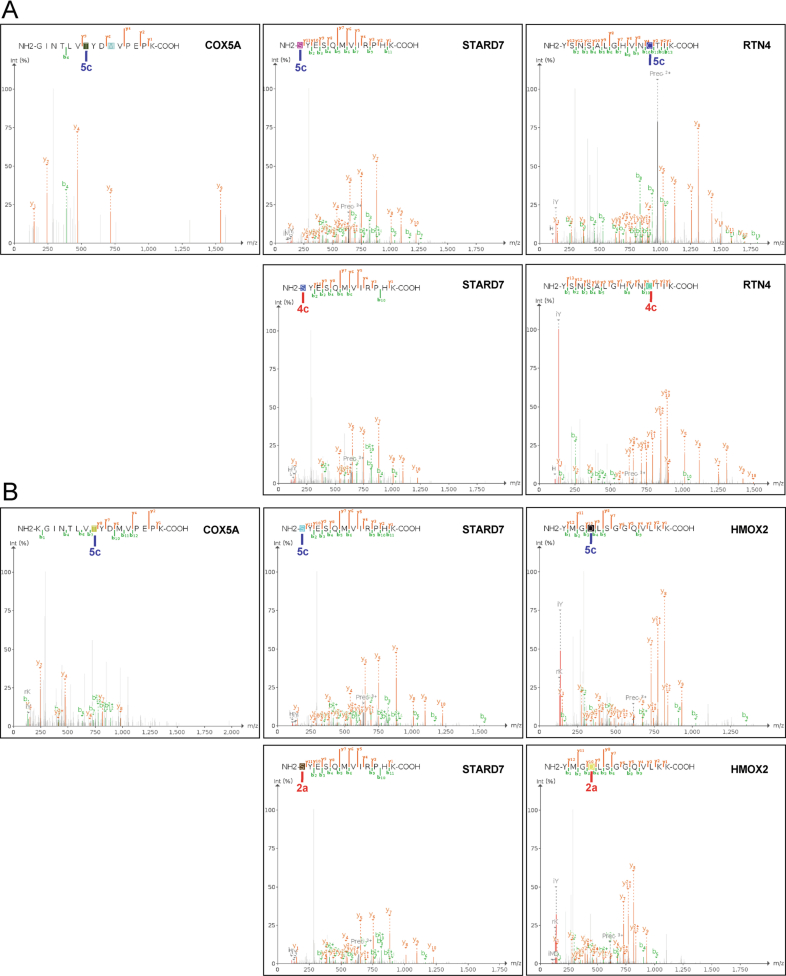
Examples of SEE-CITE modified spectra. **A**, representative spectra from datasets shown in ‘Fig. 5b,c’, **B**, representative spectra from datasets shown in ‘Extended Data Fig. 7C.’ Spectra generated in PDV. All MS data can be found in Supplementary Table 9.

We next extended SEE-CITE to de novo off-target binding site discovery. We chose to pursue four parallel lines of SEE-CITE inquiry: (1) analysis of endogenous proteins labelled in cells, (2) heterologous overexpression of targets identified by AfBPP, (3) mutational analysis to confirm binding sites and (4) functional studies. We generated a set of SEE-CITE probe–probe comparison datasets (**4c** versus **5c**, **5c** versus **2a**, and **5c** versus **3**; Fig. 5a–c, Extended Data Fig. 7a–c and Supplementary Table 9), and despite some differences in general labelling (Supplementary Note 7), we observed clear probe- and site-specific target engagement across all three probe–probe SEE-CITE comparisons (Fig. 5b,c and Extended Data Fig. 7b,c). For many SEE-CITE-labelled proteins, including COX5A, RTN4 and ABHD16A, a clear preference for labelling by **5c** was apparent. By contrast, other targets, such as HMOX2, EPHX2, STARD7 and SLC25A20, displayed several quantified labelling sites with distinct probe preferences, indicating the presence of distinct binding sites for each probe pair (Fig. 5b,c and Extended Data Fig. 7b–d). Manual inspection of labelled spectra confirmed the identified labelling regions (Extended Data Fig. 8). These examples provide initial evidence that, on a proteome-wide basis, similar to our labelling studies using recombinant ABL1, SEE-CITE discerns binding sites in a probe-dependent manner.

### Evaluating target engagement for STARD7

**Extended Data Fig. 9:**
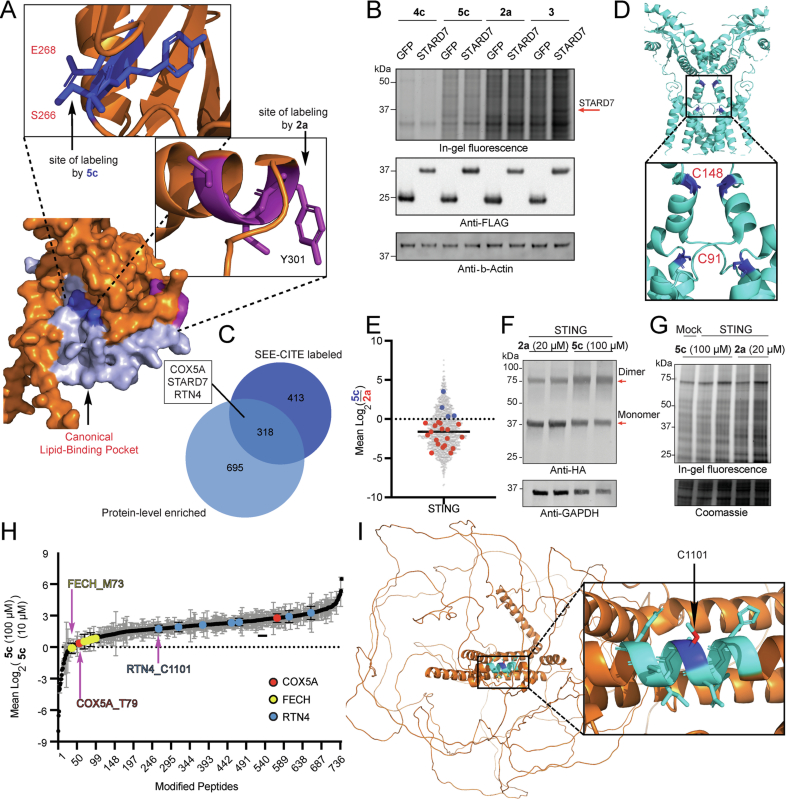
Confirming the labeling of STARD7 and STING. **A**, AlphaFold protein structure of mouse STARD7 highlighting sites of labeling (blue and purple) and the canonical lipid-binding pocket (light purple) (UniProt: Q66JV0, AF-Q66JV0-F1-v4). **B**, In-gel fluorescence of HEK293T cells transiently overexpressing FLAG-tagged GFP or STARD7 treated with various probes tested (10 μM, 1 h). Data presented are from n = 2 independent experiments. **C**, Overlapping SEE-CITE labeled proteins by **4c** (100 μM)/ **5c** (100 μM) in ‘Fig. 5b’, **3** (4 μM)/ **5c** (20 μM) in ‘Extended Data Fig. 7C’ and **5c** (10 μM)/ **5c** (100 μM) in ‘H’ with **5c**-enriched proteins from protein-directed AfBPP in ‘Extended Data Fig. 6F’ in K562 cells. Treatment time is 1 h. **D**, Mapping sites of labeling for STING by **5c** (PDB: 6NT5 for STING dimer). **E**, Scatter plots of STING-modified peptides in HEK293T cells transiently overexpressing STING treated with **2a** (20 μM, 1 h) or **5c** (100 μM, 1 h). Data are average log2(H/L) values (n = 2 biological replicates) including singletons imputed using H/L ratio of 20. Colored dots are shown for STING labeled peptides. **F**, Immunoblotting to validate of STING-HA expression as a dimer (~75 kDa) and a monomer (~37 kDa) in HEK293T cells treated with **2a** (20 μM, 1 h) or **5c** (100 μM, 1 h). **G**, In-gel fluorescence analysis of the same labeled proteome in ‘F’. The mock sample was prepared separately. **H**, Waterfall plot using average log_2_(H/L) values (n = 3 biological replicates) and the standard error of the mean (SEM) values for peptides modified by **5c** (10 μM or 100 μM, 1 h) in K562 cells excluding singletons. Colored dots are shown for COX5A (red)-, FECH (yellow)- and RTN4 (blue)- labeled peptides. **I**, AlphaFold protein structure of human RTN4 with probe-labeled peptide harboring C1101 residue highlighted (AlphaFold ID: AF-Q9NQC3-F1). For ‘**F**,**G**’, data presented are from n = 1 independent experiment. All MS data can be found in Supplementary Table 9. Source data

Inspired by the presence of two potential binding sites in STARD7 (Fig. 5b,c and Extended Data Figs. 7b–d, 8a,b and 9a) and its essential role in mitochondrial function and morphology^60,61^, which is in line with mitochondrial-preferred labelling by asciminib probes (Fig. 4i,j and Extended Data Fig. 6i–l), we chose STARD7 as an initial case study protein. We find that both **2a** and **3** show clear STARD7 labelling, while **5c** showed weaker, but still apparent, signal (Extended Data Fig. 9b). We suspect that the differential STARD7 labelling signal may reflect distinct binding sites engaged in a probe-specific manner, with **5c** engaging a reported hydrophobic lipid-binding pocket with dual specificity for phosphatidylcholine (PC) and ceramide^60,62^, whereas **2a** and **3** preferentially label a less-characterized pocket that is more solvent exposed and located distal to the canonical hydrophobic cage (Extended Data Fig. 9a).

### SEE-CITE is compatible with heterologous overexpression

While labelling sites were identified for 731 total **5c**-labelled proteins, some proteins that were captured by our protein-AfBPP were still not identified by SEE-CITE, perhaps due to lower protein expression, less favourable peptide sequences for detection, or other factors (Extended Data Fig. 9c). An example of such an ‘undetected’ protein is STING. As STING is known to harbour multiple small-molecule and lipid binding sites^63,64^, we opted to use STING as a test case for SEE-CITE analysis with heterologous overexpression. SEE-CITE identified two cysteine labelling sites in STING: C91, a known palmitoylation site, and C148, which plays a key role in inducing the STING polymerization^65^ (Extended Data Fig. 9d), with labelling favoured by **5c** (Extended Data Fig. 9d,e). Illustrating the added value of SEE-CITE, no signal was detectable for STING via gel-based analysis (Extended Data Fig. 9f,g). Taken together, our findings align with recent reports of comparatively promiscuous STING small-molecule binding and labelling by diazirine-containing compounds^14^.

### SEE-CITE captures proteins across the protein abundance spectrum

While SEE-CITE captured 49 total kinases, BCR-ABL was unexpectedly missing (Supplementary Table 9 and Supplementary Note 8). As we had been able to detect labelled ABL1 peptides with recombinant protein, we also considered whether protein abundance might contribute to the relative ease of SEE-CITE detection. Bulk proteomic analysis revealed that SEE-CITE is relatively insensitive to protein abundance, with only modest bias towards more abundant proteins (Supplementary Fig. 7a). Exemplary detected low-abundance proteins, such as STARD7, ARHGEF18 and NDUFA13 (Supplementary Table 9), were captured by SEE-CITE, whereas other medium abundance proteins, such as STING and ABL1, were not. Therefore, we expect that the absence of BCR-ABL peptides may stem from a confluence of factors, with modest expression contributing to some degree, alongside factors such as photocrosslinking efficiency and peptide chromatographic properties.

### Dose-dependent SEE-CITE identifies functional sites in RTN4

To further prioritize specific targets of **5c**, we generated dose-dependent SEE-CITE labelling analysis (Extended Data Fig. 9h), which revealed dose-dependent labelling for most proteins. One notable exception was FECH, for which all labelled peptides saturated at low probe concentration; this labelling is consistent with FECH functioning as a promiscuous diazirine binder^66,67^. This analysis also highlighted both RTN4 and COX5A as sites that favoured **5c** labelling at lower probe concentrations. For RTN4, the labelling was detected within the NOGO66 domain of RTN4 (C1101 and A1075-Y1076), which is known to bind to RTNR^68^. As RTN4 is predicted to be highly disordered by AlphaFold (Extended Data Fig. 9I), we turned again to gel-based labelling analysis to confirm the SEE-CITE proteomic and probe the likelihood of a small-molecule binding site. A single mutation (C1101S) attenuated probe labelling, consistent with this cysteine functioning as a primary probe binding site (Fig. 5d and Supplementary Fig. 7b,c). This labelling could be off-competed by 10 μM asciminib (Fig. 5e). Using an established secreted alkaline phosphatase (AP) receptor binding assay^69^ (Fig. 5f), we observed that both asciminib treatment and the bulky drug-binding mimetic C1101W point mutation s RTNR binding (Fig. 5f–h), indicating that compound binding probably stabilizes NOGO66-receptor binding. These findings align with the prior report^70^ that PC lipid binding promotes NOGO66 folding required to engage RTNR.

### SEE-CITE confirms COX5A as a functional target of asciminib

**Extended Data Fig. 10:**
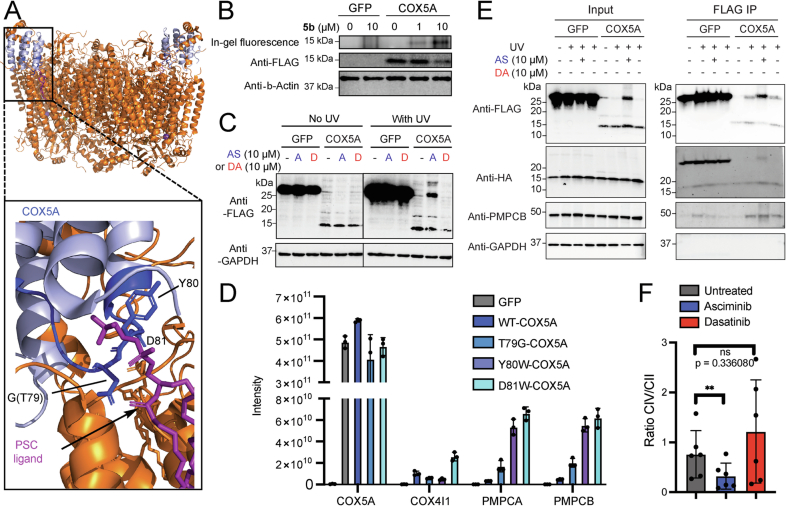
Characterization of COX5A as a bona fide off-target candidate of asciminib. **A**, Structural representation of COX5A-labeled sites (G(T)79, Y80 and D81) in close proximity with a defined lipid-binding pocket with phosphotidylsulfocholine (PSC) ligand (PDB: 1V54 for bovine heart cytochrome C oxidase structure). **B-C**, In-gel fluorescence ‘B’ and immunoblot analysis ‘C’ of HEK293T cells transiently overexpressing FLAG-tagged GFP or COX5A treated with **5b** (1 μM, 10 μM, 1 h) in ‘A’ or asciminib/ dasatinib (10 μM, 1 h) in ‘B’ in a UV-dependent manner. **D**, AP-MS analysis of HEK293T cells transiently overexpressing FLAG-tagged GFP, wildtype (WT) and mutant COX5A, showing protein intensities (n = 3 biological replicates). Data presented are mean ± SD. **E**, Co-immunoprecipitation of COX5A-HA overexpressing HEK293T stable cells that were transiently transfected with COX5A-FLAG, treated with asciminib or dasatinib (10 μM, 1 h). **F**, Mean ratios of assembled complex IV to Complex II assessed by Blue Native Polyacrylamide Gel Electrophoresis (BNGE) for HEK293T cells treated with asciminib or dasatinib (10 μM, 6 h). Quantitative assembly blots were shown in ‘Supplementary Fig. 7F’. Data presented are mean ± SD with significance values between experimental conditions calculated using paired student’s t-test (n = 6 biological replicates; from left to right, p = 0.008397 and 0.336080) with *p < 0.05, **p < 0.01,***p < 0.001, and NS (not significant) p > 0.05. For ‘**B**,**C**,**E**’, data presented are representative n = 3 (**B**,**C**) and n = 2 (**E**) independent experiments. All MS data can be found in Supplementary Table 9. Source data

Throughout our asciminib proteomic target discovery studies, the mitochondrial protein COX5A consistently stood out as a candidate target that exhibited preferential capture by our asciminib probes. The SEE-CITE-identified COX5A labelling region (T79-Y82) was located in a highly conserved region immediately adjacent to a known PC binding site. Docking of asciminib into this site showed a favourable binding pose (Fig. 5I and Extended Data Fig. 10a). Gel-based analysis confirmed COX5A labelling by asciminib, with clear dose dependence and specificity for asciminib over dasatinib (Fig. 5j,k and Extended Data Fig. 10b). Bulkier drug-like mutations at the identified binding site blocked probe binding (Fig. 5l). By contrast, the T79G mutation—glycine is conserved at this position for most species other than primates (Supplementary Fig. 7d)—afforded modestly increased labelling. Two of the bulkier mutations (T79W and D81W) appeared to promote drug-independent accumulation of the putative COX5a precursor protein retaining the signal peptide (Fig. 5m), suggestive of mutation-dependent mitochondrial import stress^71^. We also noted that both asciminib and these mutations caused the appearance of a 25-kDa FLAG-containing band, and that detection of this new protein species required UV irradiation (Fig. 5m and Extended Data Fig. 10c). As this species appeared specific to asciminib, we opted to investigate its nature further. We initially speculated that this band might match a ubiquitin conjugate or the COX4I1–COX5A intermediate, formed during assembly of complex IV. Immunoprecipitation studies ruled out these hypotheses (Supplementary Fig. 7e,f). Therefore, we subjected COX5A to affinity-purification (AP)-MS analysis, comparing the target profiles for wild-type and mutant COX5A, which revealed that the alpha and beta subunits of the mitochondrial-processing peptidase (PMPCA and PMPCB) showed mutation-dependent increased interactions with COX5A (Extended Data Fig. 10d). Immunoprecipitation analysis confirmed this interaction between COX5A and PMPCB, which was promoted by point mutants and also increased by asciminib (Fig. 5n and Extended Data Fig. 10d); notably, the two point mutants that promoted increased precursor COX5A were also found to accumulate in the detergent insoluble fraction, again suggesting challenges with import, processing or assembly. We hypothesized that COX5A might be forming homodimers in a drug-dependent manner, which was confirmed by coimmunoprecipitation (Extended Data Fig. 10e). We also considered the broader impacts of asciminib on mitochondrial function. We find that asciminib decreases complex IV assembly (Extended Data Fig. 10e and Supplementary Fig. 7g). We also measured rates of oxygen consumption in plasma membrane-permeabilized cells provided with pyruvate with malate as respiratory substrates. We observed that COX5A overexpression caused a trend towards increased rates of mitochondrial respiration and that asciminib induced a decrease in respiration (Fig. 5o). Therefore, we conclude that asciminib binding to COX5A can cause the formation of COX5A dimers, association with PMPCB, and retention of the precursor protein (Fig. 5p). This activity may also help rationalize some of the broader effects of asciminib on mitochondrial function, particularly the observed asciminib-induced decrease in respiration, which aligns with prior work^72^.

## Discussion

Application of SEE-CITE PAL chemoproteomic platform to scout probes and FDA-approved BCR-ABL kinase inhibitors dasatinib and asciminib revealed both known and previously unreported targets, including functional sites in RTN4 and COX5A. Furthermore, we expect that our discovery that COX5A interacts with the mitochondrial processing peptidase (MPP) may assign MPP as the protease responsible for cleaving the COX5A signal peptide. While all of our data indicate that COX5A is a bona fide target of asciminib, the potency of this interaction is still modest with engagement primarily at low micromolar drug concentrations; these concentrations align with the reported *C*_max_ value of asciminib but exceed that required for on target BCR-ABL inhibition^73^. As the asciminib binding sites in BCR-ABL, COX5A and RTN4 are all known to bind to lipids, we also expect that our work may highlight other potentially druggable lipid binding pockets and could indicate that asciminib may be a privileged scaffold for engaging these sites. The presence of many targets in our datasets with multiple small-molecule binding sites also generally indicates how valuable site-level analysis, generated by methods such as SEE-CITE, will be for future studies, as these targets may fail to meet prioritization criteria in protein-centric analyses. Enabled by our mass offset workflow and localization scores that enhanced the accuracy and confidence in modification localization while simultaneously reducing search speed, we find that diazirine-enabled photocrosslinking occurs preferentially at a subset of amino acids within the proteome, which could result in some biased labelling. Reactive cysteines, such as C1101 in RTN4^74,75^, appear to favour labelling, which may complicate clearly delineating reactivity- from binding-driven interactions. For RTN4, our data suggest that the compound interaction is, at least in part, driven by binding activity; however, it remains to be seen whether this will generalize to other PAL-labelled cysteines. It is also important to note that absence of detection by PAL proteomics may not mean that a protein is not labelled by an indicated probe, as shown by our gel-based analysis of STARD7. As shown by our BCR-ABL1 case study, detection of some known labelling sites may remain challenging. Increased input proteome and higher probe concentrations can in part address these limitations. However, for the latter, this strategy comes at the expense of an increased number of compound binders, many of which are probably low-occupancy labelling events. As illustrated by the recombinant ABL1 protein labelling, a more straightforward approach is to subject an already prioritized candidate target to SEE-CITE labelling to pinpoint likely binding sites.

Looking to the future, we expect that multiplexed comparisons of relative labelling for many SEE-CITE probes should readily reveal clear structure–activity relationships across larger compound libraries. These studies will additionally benefit from targeted proteomic approaches, alternative sequence specific proteases and enrichment-based multiplexing reagents^34,76,77^. We also foresee widespread utility for SEE-CITE in capturing distinct drug binding sites and, more broadly, for interaction site mapping for larger and highly gas-phase labile molecules, including lipids such as cholesterol, glycans, and even protein–protein interactions, using unnatural amino acids incorporated through genetic code expansion.

## Methods

### Cell lines and culture conditions

Cell culture reagents including Dulbecco’s modified Eagle medium (DMEM), penicillin–streptomycin (Pen/Strep) and Dulbecco’s phosphate-buffered saline were purchased from Fisher Scientific. Fetal bovine serum (FBS) was purchased from Avantor Seradigm (lot #214B17). All cell lines were obtained from ATCC and were maintained at a low passage number (<15 passages). K562 and KCL22 cells were cultured in RPMI supplemented with 10% FBS and 1% antibiotics (Pen/Strep, 100 U ml^−1^). HEK293T cells were maintained in DMEM supplemented with 10% FBS, 1% antibiotics (Pen/Strep, 100 U ml^−1^). All media were filtered (0.22 μm) before use. Cells were maintained in a humidified incubator at 37 °C with 5 % CO_2_. Cell lines were tested monthly for mycoplasma using the Mycoplasma Detection Kit (InvivoGen).

### Cloning of plasmids

List of plasmids with detailed information used in this study can be found in Supplementary Table 11. pDONR223 vector containing sequence for COX5A (plasmid #HsCD00042518) and pDONR221 vector containing sequence for STARD7 (plasmid #HsCD00719027) were obtained from DNASU and subcloned using GateWay cloning into C-terminal FLAG destination vector, pRK5-C-FLAG, which was a kind gift from T. Wucherpfennig. The Myc-DDK-tagged open reading frame clone of *Homo sapiens* reticulon 4 (RTN4), transcript variant 3, was obtained from Origene (RC221080) and also subcloned into pRK5-C-FLAG via GateWay cloning according to the manufacturer’s protocol.

### Generation of HEK293T and HeLa stable cell lines

For preparation of lentiviruses to generate HEK293T and HeLa cell lines stably expressing GFP, wild-type COX5A-HA and Y80W-COX5A-HA. HEK293T cells in 10-cm plates were transfected at ~80–90% confluency with lentiviral vector pTwist Lenti SFFV Puro containing wild-type COX5A-HA or mutant Y80W-COX5A HA (10 μg; Twist Biosciences) or FUGW containing GFP (10 μg; a kind gift from the Mikkola lab) with the lentiviral packaging plasmids pCMV-VSV-G (4 μg; Addgene #8454) and Δ8.9 (8 μg; a kind gift from the Divakaruni lab) and 66 μl of LipoFexin (Lamda Biotech, TS310) in Opti-MEM (Gibco) media for 48 h for lentiviral production according to the manufacturer’s protocol. For preparation of retroviruses to generate HEK293T and HeLa cell lines stably expressing GFP-His and RTNR-His, HEK-G3P cells in 10-cm plates were transfected at ~75% confluency with retroviral vector pCLHCX containing GFP-FLAG-His or RTNR-FLAG-His (1.5 μg) with the retroviral plasmid pVSVG (1.5 μg) and 20 μl of FUGENE HD transfection reagent (Promega, PRE2311) in Opti-MEM (500 μl; Gibco). After 24 h, the medium containing transfection reagent was replaced with antibiotic-free DMEM for another 24 h for retroviral production. The virus-containing medium was collected, and 3 ml of virus-containing medium with 8 μg ml^−1^ of polybrene was added to HEK293T and HeLat cells at ~40–50% confluency for overnight infection. After 24-h infection, the retrovirus was removed, and cells were incubated with new antibiotic-free media. Hygromycin at 400 μg ml^−1^ for HEK293T cells or at 200 μg ml^−1^ for HeLa cells was added for selection of transduced cells. Selection media was replaced every 24–48 h until the appearance of visible colonies of transduced cells. Cells were expanded to larger plates and cryogenically frozen in FBS containing 10% dimethyl sulfoxide (DMSO) for future use.

### Mutagenesis

Point mutations (C1101S RTN4-FLAG, C1101W AP-Nogo66-His and T79G-, T79W-, Y80F-, Y80W-, D81W- and D81N-COX5A) were created by PCR-based site-directed mutagenesis. All primers used to generate mutant constructs can be found in Supplementary Table 11. Mutant plasmids were then transformed into competent TOP10 cells. Colonies were selected and grown in SOC medium at 37 °C for 2–3 h. Successful mutagenesis was confirmed by sequencing.

### Transient transfections

HEK293T cells were plated into six-well or 10-cm plates 24-h before transfection and were transiently transfected at 70–80% confluency with the relevant plasmids. For transfection done in a six-well plate, plasmid (1.5 μg) and PEI MAX-Transfection Grade Linear Polyethylenimine Hydrochloride (MW 40,000) (Polysciences, 24765-1, 7.5 μl) were each diluted with OptiMEM (75 μl). After 5-min equilibration at room temperature, the two diluted mixtures were mixed gently and incubated for another 20 min at room temperature. The transfection cocktail was then added dropwise to the cells and incubated for 24 h using RTN4 plasmids or 48 h using COX5A and STARD7 plasmids. Transfection done in a 10-cm plate (from FLAG-immunoprecipitation experiments) followed the same transfection protocol using 5 μg of plasmid, 35 μl of PEI reagent and 300 μl of OptiMEM.

### Drug and/or probe treatment and UV irradiation for in situ PAL

All probes and drugs were made up as 1,000× stock solutions in DMSO. Adherent cells at 90–100% confluency (HEK293T cells) or 5.0 × 10^6^ suspension cells (K562/KCL-22 cells) were treated with various probes at the tested concentrations for 1 h for photoaffinity experiments with probe-treated samples only or pretreated with asciminib or dasatinib at the tested concentrations for 30 min followed by 30-min or 1-h probe treatment at detailed separately for competitive PAL experiments. Treatment was done in serum-free and antibiotic-free DMEM (for adherent cells) or RPMI (for suspension cells) media at 37 °C with 5 % CO_2_ for the indicated time. For vehicle/mock samples, pure DMSO was used for the treatment instead. Treated cells were subjected to UV irradiation on ice (plates with no lids) at 350 nm for 20 min.

### Cell collection, cell pellet storage, cell lysis and determination of protein concentration

Cells were washed with cold 1× phosphate-buffered saline (PBS), gently transferred into cold PBS and collected by centrifugation at 1,000*g* for 5 min. Cell pellets were then washed twice with cold PBS and lysed as detailed next or snap-frozen with liquid nitrogen, followed by −80 °C storage until usage.

For lysis method 1 (used in all gel-based AfBPP analyses with RTN4- and STING-overexpressing cells, protein-level and peptide-level SEE-CITE proteomics experiments excluding AP-MS, and FLAG immunoprecipitation excluding ubiquitin assay), cells were lysed in 0.3% CHAPS (3-[(3-cholamidopropyl) dimethylammonio]-1-propanesulfonate) in 1× PBS (pH 7.4) and incubated on ice for 30 min.

For lysis method 2 (used in AP-MS experiments), cells were lysed in 0.3% CHAPS in 20 mM HEPES (4-(2-hydroxyethyl)-1-piperazineethanesulfonic acid) on ice for 30 min.

For lysis method 3 (used in all gel-based experiments with COX5A-overexpressing cells), cells were lysed in 8 M urea in 1× PBS (pH 7.4) with four cycles of freeze–thaw using liquid nitrogen. For one co-immunoprecipitation experiment with HEK293T cells overexpressing both COX5A–HA (stable) and COX5A–FLAG (transient), the insoluble fractions were resolubilized with 8 M urea in 1× PBS (pH 7.4)

For lysis method 4 (used in competitive in-gel fluorescence for RTN4-overexpressing cells and gel-based AfBPP for STARD7-overexpressing cells), cells were lysed in 0.3% CHAPS in 1× PBS (pH 7.4) with four cycles of freeze–thaw using liquid nitrogen.

For lysis method 5 used in FLAG-immunoprecipitation ubiquitin assay, cells were lysed in 1× RIPA buffer for 30 min on ice.

All lysis buffers except 8 M urea included 5 mg ml^−1^ ethylenediaminetetraacetic acid (EDTA)-free protease inhibitor cocktail (Roche #11836170001). After lysis, cellular debris was clarified by centrifugation at 3,000*g* for 5–10 min, and the soluble fractions were transferred to fresh microcentrifuge tubes. Protein concentrations were determined using a Bio-Rad detergent-compatible protein assay kit (Bio-Rad Life Science #5000113 and #5000114) for lysis methods 1–4 and using Pierce BCA protein assay kit (Thermo Scientific #23225) for lysis method 5. Normalized samples were prepared at different concentrations for different experiments using either 1× PBS (pH 7.4) or the same lysis buffer.

### Click chemistry

Before click chemistry, a premixed copper-catalysed azide-alkyne cycloaddition (CuAAC) cocktail was freshly prepared at a 3:1:1:1 (v/v/v/v) ratio of 1.75 mM of tris[(1-benzyl-1H-1,2,3-triazol-4-yl)methyl]amine (TBTA) in ^*t*^BuOH/DMSO 4/1 per sample, 50 mM of CuSO_4_ in molecular biology (MB) water, 50 mM of tris-(2-carboxyethyl)phosphine hydrochloride (TCEP) in MB water to 1.25 mM of 5-TAMRA-azide (Vector Laboratories #CCT-AZ109-5) in DMSO or to 5 mM of a biotin-azide reagent in DMSO.

For gel-based AfBPP, normalized lysates (25 μl, 1 mg ml^−1^) were prepared with 1× PBS and mixed with 3 μl of a premixed CuAAC cocktail prepared with 5-TAMRA-azide. For protein-directed AfBPP proteomics (label-free quantification, LFQ) or peptide-directed SEE-CITE proteomics involving SP3 clean-up, normalized samples (200 μl, 1 mg ml^−1^ or 200 μl, 2 mg ml^−1^, respectively) were prepared in 1× PBS and mixed with 24 μl of a premixed CuAAC cocktail prepared with light biotin-azide.

For optimized peptide-directed SEE-CITE proteomics with chloroform/methanol precipitation, normalized lysates (1,600 μl, 2 mg ml^−1^) were clicked with 192 μl of a premixed CuAAC cocktail prepared with either a light biotin-azide or light biotin-azide sCIP reagent (NB3192). For optimized peptide-directed SEE-CITE-based isoTOP-ABPP with chloroform/methanol precipitation, a pair of normalized samples (800 μl, 2 mg ml^−1^) were clicked with heavy and light biotin-azide reagents (96 μl each).

The CuAAc reaction was done after 1-h incubation at room temperature in the dark. Post-clicked samples were subjected to sodium dodecyl sulfate–polyacrylamide gel electrophoresis (SDS–PAGE) analysis only, SDS–PAGE analysis followed by western blotting, or further proteomics samples preparation steps detailed below.

### SDS–PAGE analysis and imaging

One part of 4× loading dye (Bio-Rad #1610747) with 10% β-mercaptoethanol was added to three parts of samples (ready for SDS–PAGE analysis) to achieve 1× loading buffer. Samples, except for those clicked with 5-TAMRA azide in gel-based AfBPP experiments, were then subjected to 5-min heat denaturation at 95 °C. For gel-based AfBPP, SDS–PAGE analysis was done at 140 V using 4–12% Criterion XT Bis-Tris protein gel (Bio-Rad #3450124 or #3450124) or NUPAGE 10% Bis-Tris midi protein gel (Invitrogen #WBT01020BOX) with either 1× XT MOPS running buffer (Bio-Rad #1610788) or 1× NUPAGE MOPS SDS running buffer (Invitrogen #NP0001) or 1× NUPAGE MES SDS running buffer (Invitrogen #NP0002). For other experiments, SDS–PAGE analyses were done at 140 V using 4–20% Criterion TGX precast midi protein gel (Bio-Rad #5671094 or #5671095) with 1× Tris/glycine/SDS electrophoresis buffer (Bio-Rad #1610772EU). After SDS–PAGE analysis, the gel was imaged by a Bio-Rad ChemiDoc Imaging System to obtain a rhodamine image for gel-based AfBPP with a Bis-Tris gel or a stain-free loading control image for other TGX gels. After that, the gel was either stained by Coomassie InstantBlue for at least 2 h (only for gel-based AfBPP) or subjected to western blot analysis as described below.

### Western blots

Before western blot analysis, a rhodamine image (for in-gel fluorescence experiment) or a stain-free image (for any other experiments) was obtained by a Bio-Rad ChemiDoc Imaging System. Proteins were then transferred from the gel to a pre-equilibrated nitrocellulose membrane (Bio-Rad #1620112) or Immun-Blot polyvinylidene fluoride membrane (pre-activated with 200-proof ethanol) (Bio-Rad #162177) using a semi-dry transfer system (Bio-Rad Transblot) with a low or mixed molecular weight setting. The membrane was blocked with 5% (w/v) milk in 1× Tris-buffered saline (TBS) for 1 h at room temperature. After blocking, the membrane was incubated with one of the rabbit primary antibodies listed below at a ratio of 1:3,000 in 5% (w/v) milk in 1× TBS overnight at 4 °C, washed with 1× TBS for 10 min three times the following day and incubated with a secondary antibody, IRDye 800CW goat anti-rabbit secondary antibody (Li-Cor Biotechnology, 926-32211, #D50528-07), at a ratio of 1:5,000 in 5% (w/v) milk in 1× TBST (TBS with 0.1% Tween20) room temperature for 1 h. After secondary antibody incubation, the membrane was washed with 1× TBS for 10 min three times and imaged using a Bio-Rad ChemiDoc Imaging System to obtain western blot results. For loading control, the membrane underwent the similar western blot analysis as described above using mouse anti-β-actin antibody (Cell Signaling, #3700S, #21) or mouse GAPDH monoclonal antibody (Proteintech, #60004-1-Ig, #10080731) as a primary antibody at 1:3,000 dilution and IRDye 680RD donkey anti-mouse secondary antibody (Li-Cor Biotechnology, 926-68072, #D41217-05) as a secondary antibody at 1:5000 dilution. The membrane was again imaged to obtain an anti-β-actin or an anti-GAPDH blot as a loading control. All primary antibodies in the study, used at 1:3,000 dilution, include: DYKDDDDK (FLAG) (Cell Signaling Technology, #14793, #7), c-Abl (Cell Signaling, #2862S, #16), phospho-c-Abl (Y245) (Cell Signaling, #2861S, #9), STAT5 (D2O6Y) (Cell Signaling, #94205, #5), phospho-STAT5A (Y694) (ABclonal, #AP0758, #4000000176), CRKL (ABclonal, #A11735. #0030740301), phospho-CRKL (Y207) (ABclonal, #AP0824, #21156250301), COXIV (Proteintech, #11242-1-AP, #00163993), PMPCB (Proteintech, #16064-1-AP, #00040152), β-actin (8H10D10) (Cell Signaling, #3700S, #21) and GAPDH (Proteintech, #60004-1-Ig, #10080731).

#### General procedures for sample clean-up in proteomic sample preparation

##### SP3 clean-up for peptide-level SEE-CITE samples or protein-level AfBPP

In total, 20 or 10 μl of Sera-Mag Speed-Beads Carboxyl Magnetic Beads, hydrophobic (GE Healthcare #65152105050250) and 20 or 10 μl of Sera-Mag Speed- Beads Carboxyl Magnetic Beads, hydrophilic (GE Healthcare #45152105050250) were mixed and washed with water three times and resuspended in 40 or 20 μl of water for each SEE-CITE sample or protein-level AfBPP sample, respectively. The bead slurries (40 or 20 μl) were then transferred to post-clicked samples (SEE-CITE sample or protein-level AfBPP sample, respectively) for 10-min incubation with shaking (1,000 rpm) for 10 min at room temperature. Absolute ethanol (500 μl) (>60%) was added to each sample, and the samples were incubated with shaking (1,000 rpm) for 10 min at room temperature. On a magnetic rack, supernatant was aspirated off, and samples were washed three times with 80% ethanol in water (400 μl).

##### Chloroform/methanol (CHCl_3_/MeOH) precipitation for peptide-level SEE-CITE sample preparation

To each sample, cold MeOH (3× volume), cold CHCl_3_ (1× volume) and cold water (3× volume) were added in this order, followed by centrifugation for 10 min (3,800 g) at 4 °C. The top layer was aspirated off without disturbing the white protein disc followed by MeOH addition (3× volume). After another 10-min centrifugation (3,800*g*) at 4 °C, the supernatant was aspirated off to give protein pellets. The pellet was washed three times in cold MeOH (1× volume) with gentle sonication and centrifuged (1,800*g*) for 2 min to pellet protein. After each wash, the supernatant was removed to give only protein pellets.

##### Preparation of peptide-directed SEE-CITE samples with NeutrAvidin enrichment

The probe-labelled K562 or KCL-22 proteome was prepared as described in the ‘Drug and/or probe treatment and UV irradiation for in situ PAL’ section. Subsequently, to each post-clicked SEE-CITE sample involving SP3 clean-up was added 10% sodium dodecyl sulfate (SDS) (20 μl) (working [SDS] = 1%) and benzonase (0.5 μl) (Fisher Scientific #70-664-3) for 30-min incubation at 37 °C. The samples (200 μl, 2 mg ml^−1^) were subjected to SP3 clean-up described earlier, followed by resuspension in 2 M urea (200 μl) prepared in PBS with 0.5% SDS. Resuspended samples were reduced with dithiothreitol (DTT) (10 μl of 200 mM stock in water, final concentration 10 mM) at 65 °C for 15 min and alkylated with iodoacetamide (10 μl of 400 mM stock in water, final concentration 20 mM) for 30 min at 37 °C. After that, absolute ethanol (400 μl) was added to each sample for 5-min incubation with shaking (1,000 rpm) at room temperature. Beads were again washed three times with 80% ethanol in water (400 μl) and resuspended in 200 μl of 2 M urea in 1× PBS. Trypsin (3 μl, 1 mg ml^−1^) was added for overnight digestion at 37 °C with shaking (200 rpm). After digestion, acetonitrile (3.8 ml) was added to each sample for 10-min incubation with shaking (1,000 rpm) at room temperature. Beads were then washed with acetonitrile (1 ml) three times using a magnetic rack. Peptides were eluted from SP3 beads with 2% DMSO in water (100 μl) for 30 min at 37 °C with shaking (1,000 rpm). The elution was repeated, and elution fractions were combined and subjected to enrichment as detailed below.

To each post-clicked SEE-CITE sample involving CHCl_3_/MeOH precipitation detailed earlier was added 6 M urea (a half volume of the lysate; for example, 400 μl of 6 M urea for 800 μl of the lysate, 600 μl of 6 M urea for 1,200 μl of the lysate and 800 μl of 6 M urea for 1,600 μl of the lysate) for protein resuspension. Then, 200 mM DTT in MB water (20 μl for 800 μl, 30 μl for 1,200 μl, or 40 μl for 1,600 μl of lysate) was added to resuspended samples (final [DTT] = 10 mM) followed by 15-min incubation at 65 °C or 30-min incubation at 37 °C (for one optimization experiment only). Then, 400 mM of IAin MB water (20 μl for 800 μl/30 μl for 1,200 μl/40 μl for 1,600 μl of lysate) was added (final [IA] was 20 mM) followed by 30-min incubation at 37 °C, and 1× PBS was added to adjust [urea] = 2 M (760 μl for 800 μl of lysate/1,140 μl for 1,200 μl of lysate/1,520 μl for 1,600 μl of lysate) followed by trypsin addition (8 μl, 5 mg ml^−1^) for overnight digestion with shaking (200 rpm) at 37 °C. After digestion, 10% SDS was added to each peptide solution (for example, 270 μl for 1,600 μl lysate), and the samples were heated for 5 min at 60 °C to solubilize insoluble materials. Then, 1× PBS (~10 ml) was added to adjust [SDS] to 0.2% (urea concentration ~0.36 M). Enrichment is detailed below.

For each sample, 50 μl of NeutrAvidin Agarose resin slurry (Pierce #29200) was washed three times in 1× IAP buffer (50 mM MOPS pH 7.2, 10 mM sodium phosphate and 50 mM NaCl buffer), resuspended in 1× IAP buffer (500 μl) and added to the SP3-clean-up peptide solutions or CHCl_3_/MeOH-clean-up samples after adjusting [SDS] to 0.2%. Peptide enrichment was done with rotation for 2 h at room temperature. After incubation, beads were pelleted by centrifugation (1,800g, 2 min), washed three times with 1× PBS and three times with water (1 ml per wash), followed by peptide elution with 80% acetonitrile in water containing 0.1% trifluoroacetic acid (TFA) (80 μl) for 10 min at room temperature. The second elution was done at 72 °C. The beads were quickly washed with the same solvent (40 μl). Elution and wash fractions (200 μl) were combined and dried via SpeedVac. Dried peptides were reconstituted with 5% acetonitrile and 1% formic acid in MB water and analysed by LC–MS/MS.

##### Preparation of peptide-directed SEE-CITE samples using a biotin-sCIP reagent (NB3192)

After CuAAC labelling with light biotin-azide sCIP reagent (**NB3192**), samples underwent the exact same CHCl_3_/MeOH precipitation, resuspension, reduction, alkylation and digestion as described for post-clicked SEE-CITE samples involving CHCl_3_/MeOH precipitation earlier. For each digested sample (from 1,600 μl lysate, 2 mg ml^−1^), Pierce streptavidin agarose beads (50 μl) (Pierce #20353) were washed twice with 1× PBS, resuspended in 1× PBS (500 μl) and added to each sample. Peptide enrichment was done with rotation for 2 h at room temperature. After incubation, beads were pelleted by centrifugation (1,800*g*, 2 min) and washed three times with 1× PBS and three times with water (1 ml per wash) followed by a 30-min acidic peptide elution with 2% TFA (200 μl) to cleave off the DADPS linker at room temperature. Then, 80% acetonitrile (400 μl) in water was added to briefly wash beads and capture all eluted peptides. The elution and wash fractions (600 μl) were combined and dried via SpeedVac. Dried peptides were reconstituted with 5% acetonitrile and 1% formic acid in MB water and analysed by LC–MS/MS.

##### Preparation of protein-directed AfBPP samples with streptavidin enrichment

The probe-labelled K562 or KCL-22 proteome was prepared as described in the ‘Drug and/or probe treatment and UV irradiation for in situ PAL’ section and underwent the SP3 clean-up protocol detailed earlier.

Protein elution from SP3 beads was done twice with 0.2% SDS in 1× PBS (50 μl) via 30-min incubation with shaking (1,000 rpm) at room temperature. On a magnetic rack, the elution fractions were transferred to a new 1.5-ml Eppendorf tube. For each protein sample, Pierce streptavidin agarose beads (50 μl) (Pierce #20353) was washed twice with 1× PBS, resuspended in 1× PBS (500 μl) and added to each sample. Protein enrichment was done with rotation for 2 h at room temperature. After incubation, beads were pelleted by centrifugation (1,800*g*, 2 min) and washed three times with 1× PBS and three times with water (1 ml per wash). After washing, beads were resuspended in 6 M urea in 1× PBS (200 μl), incubated with 200 mM DTT in water (10 μl), final [DTT] = 10 mM) for 15 min at 65 °C followed by 30-min incubation with 400 mM iodoacetamide in water (10 μl, final concentration 20 mM) at 37 °C. Then, 400 μl PBS was added to dilute the urea concentration to ~2 M, followed by 2-min centrifugation at 1,800*g* and removal of the supernatant. The beads were resuspended in 2 M urea in PBS (200 μl), followed by addition of trypsin (3 μl, 1 mg ml^−1^) for overnight incubation with shaking (200 rpm) at 37 °C. After digestion, 10 μl of 10% TFA was added to each ~200-μl sample to achieve the final concentration of 0.5%. Samples were then cleaned up with Pierce C18 spin tips (Thermo Fisher #87784) according to the manufacturer’s protocol. Dried peptides were reconstituted with 5% acetonitrile and 1% formic acid in MB water and analysed by LC–MS/MS.

##### Preparation of AP-MS samples

HEK293T cells transiently overexpressing FLAG-tagged GFP, wild-type COX5A or mutant COX5A including T79G-, Y80W- and D81W-COX5A were lysed by the lysis method 2, described in the ‘Cell collection, cell pellet storage and cell lysis’ section. For each sample, anti-FLAG EZView resins (10 μl) (Sigma #F2426) were washed twice with a buffer A containing 20 mM HEPES, pH 7.40, 150 mM NaCl and resuspended in buffer A (50 μl). Normalized lysate samples (250 μl, 3.2 mg ml^−1^) were prepared and incubated with prewashed resins for 2 h at 4 °C. Resins were then spun down at 8,200*g* for 1 min, followed by removal of supernatant. Resins were washed three times with buffer A, followed by protein elution in 8 M urea (40 μl) freshly prepared in buffer A via 30-min incubation with shaking (400 rpm) at 37 °C. Resin was spun down, and the eluant was collected in a new microcentrifuge tube. Resins were incubated with an additional 35 μl of 8 M urea with shaking (400 rpm) at 37 °C for 15 min. All eluted fractions containing proteins were combined, reduced with 200 mM DTT (5 μl) for 15 min at 65 °C, alkylated with 400 mM iodoacetamide (5 μl) for 30 min at 37 °C. To the samples was added buffer A (220 μl) to adjust the final urea concentration to 2 M. Trypsin (4 μl, 1 mg ml^−1^) and 100 mM CaCl_2_ (13.92 μl, final concentration 4 mM) were added to digest proteins overnight with shaking (200 rpm) at 37 °C at 200 rpm. TFA (1.74 μl) was added to acidify the samples (final concentration 0.5%). Samples were then cleaned up with Pierce C18 spin tips (Thermo Fisher #87784) according to the manufacturer’s protocol. Dried peptides were reconstituted with 5% acetonitrile and 1% formic acid in MB water and analysed by LC–MS/MS.

##### LC–MS/MS analysis

Samples were analysed by LC–MS/MS using a Thermo Scientific Orbitrap Eclipse Tribrid mass spectrometer with Xcalibur (v4.6.67.17.) software. Peptides were fractionated online using an 18-cm-long, 100-μm-inner-diameter fused silica capillary, packed in-house with bulk C18 reversed-phase resin (particle size 1.9 μm, pore size 100 Å; Dr. Maisch GmbH). The 70-min or 140-min water acetonitrile gradient was delivered using a Thermo Scientific EASY-nLC 1200 system at different flow rates (buffer A: water with 3% DMSO and 0.1% formic acid and buffer B: 80% acetonitrile with 3% DMSO and 0.1% formic acid). The detailed gradient includes 0–5 min from 3% to 10% at 300 nl min^−1^, 5–64 min from 10% to 50% at 220 nl min^−1^, and 64–70 min from 50% to 95% at 250 nl min^−1^ buffer B in buffer A for 70-min gradient, or 0–6 min from 3% to 20% at 300 nl min^−1^, 6–130 min from 20% to 38% at 220 nl min^−1^, and 130–140 min from 38% to 95% at 250 nl min^−1^ buffer B in buffer A for 140-min gradient. Data were collected with charge exclusion (1, 8, >8). Data were acquired using a data-dependent acquisition method comprising a full MS1 scan (resolution 120,000), followed by sequential MS2 scans (resolution 15,000, 30,000 and 60,000) to utilize the remainder of the 3-s cycle time. Time between master scans was set to 1 s and 3 s for compound labelling datasets, validation datasets and fractionation datasets. High-energy collisional dissociation collision energy of MS2 fragmentation was 30%.

##### Data compilation and statistics

Raw MS data collected by LC–MS/MS or mzML files converted from raw MS data were searched with MSFragger and FragPipe computational platforms (v20.0, v21.2-build38 and v22.0)

For peptide-directed SEE-CITE quantitative analyses with variable modification search, isoTOP-ABPP workflow was used as the template, adjusted for the SEE-CITE modification masses. MS1 labelling quantification with IonQuant^79^ was enabled, with Light set as *+436.2256 and Heavy set as *442.2633. The MS1 intensity ratio of heavy and light labelled peptides were reported. This analysis used Fragpipe version 20.0, MSFragger version 3.8, IonQuant version 1.9.8 and Philosopher version 5.0.0.

For peptide-directed SEE-CITE quantitative analyses with mass offset search, custom mass offset search-based workflow was used. SEE-CITE modifications with Light set as *+436.2256 and Heavy set as *442.2633 were specified as mass offsets allowed on all amino acids. Met oxidation, N-term acetylation and cysteine alkylation are still specified as variable modifications. Further details for mass offset search are discussed in the Supplementary Information. This analysis used FragPipe version 21.2-build38 (now released in FragPipe 22 under the name ‘PAL’), MSFragger version 4.1-rc33, IonQuant version 1.10.23 and Philosopher version 5.1.1-RC13. In all variable modification and mass offset searches, precursor and fragment mass tolerance was set as 20 ppm. Missed cleavages were allowed up to 2. Peptide length was set to 7–50, and peptide mass range was set to 500–5,000.

For protein-level AfBPP analyses, LFQ with IonQuant featuring FDR-controlled match-between-runs (MBR)^79^ was used. Normalization was enabled only for probe–probe LFQ analyses, while competitive and/or UV-dependent LFQ experiments were searched without the normalization module, allowing accurate protein quantification for samples with large differences in abundance in these LFQ experiments. Identified proteins from the LFQ-MBR search output were filtered by Perseus 2.0.11 to retain only proteins identified in at least two replicates (for experiments with three replicates per condition) or four replicates (for experiments with six replicates per condition). Missing values were then imputed by Perseus on the basis of the normal distribution. Imputed proteins were processed with our custom R scripts via RStudio, version 2024.09.0+375, to generate volcano plots for visualization and classification of enriched (UP), not significant (NS) and not enriched (DOWN) with log_2_(FC) (where FC is fold change) and raw *P* values. For competitive analyses, significant proteins were defined as log_2_(FC) >0.5 and *P* < 0.05. For other probe–probe and UV-dependent labelling experiments, significant proteins were defined as log_2_(FC) >1.0 and *P* < 0.05. This analysis used FragPipe version 22.0, MSFragger version 4.1, IonQuant version 1.10.27, diaTracer version 1.1.3 and Philosopher version 5.1.1. Spectra were visualized in PDV^53^. Custom Python scripts were implemented to compile labelled peptide and protein datasets. Unique proteins and unique peptides were quantified for each dataset. Unique proteins were established on the basis of UniProt protein IDs. Unique peptides were found on the basis of sequences containing modified residues. Unique modified peptides were classified by an identifier consisting of a UniProt protein ID, the modified residue and the corresponding amino acid number (ProteinID_*#); residue numbers were found by aligning the peptide sequence to the corresponding UniProt protein sequence. When there are multiple modified residues in one peptide, all the modified residue numbers will be reported as ProteinID_*#_*#.

For other non-proteomics statistical analyses, statistical values including the exact *n*, statistical test and significance are reported in the figure legends. Statistical significance was defined as *P* < 0.05 and, unless indicated otherwise, determined by two-tailed paired and unpaired Student’s *t*-tests calculated by Prism, version 10.4.1 (532).

##### Pathway analysis

For Kyoto Encyclopedia of Genes and Genomes (KEGG) and GO analyses, the subset of proteins to be analysed was searched using the Enrichr algorithm^80–82^ and plotted using Prism, version 10.4. The entirety of the proteins in the dataset used the ‘background’ dataset for statistical comparison. Pathway groups with an adjusted *P* value <0.05 were considered significantly enriched over background. The first ten terms were shown in the histograms.

##### Gel-based AfBPP analyses of ABL1 kinase domain

Recombinant human c-ABL His-tag protein (1 μM) (R&D Systems, #11091-AL) was spiked into HEK293T cells (0.5 mg ml^−1^ in Fig. 3b or 1 mg ml^−1^ in Supplementary Fig. 4b,c). For Fig. 3b, competitive gel-based AfBPP analyses were done by pretreating ABL1 in lysate with asciminib or dasatinib (0, 2, 20 μM, 0.5 h) followed by probe treatment with **4c** or **5c** (1 μM, 0.5 h). For Supplementary Fig. 4b,c, ABL1 in lysates were either pretreated with asciminib or dasatinib (100 μM, 0.5 h), followed by probe treatment with **4b**, **5c**, **4c** or **5c** (10 μM, 0.5 h) (Suppplementary Fig. 4b) or simply treated with **4c** or **5c** (1, 10 μM) (Suppplementary Fig. 4c). Drug and probe stocks were prepared in DMSO as 100× stocks. Post-treated samples were UV-irradiated on ice at 350 nm for 20 min. All samples underwent the following protocols: click chemistry for gel-based AfBPP, SDS–PAGE analysis and imaging, and western blotting, as described earlier.

##### Purification of AP fusion proteins

HEK293T cells were transiently transfected with AP fusion proteins for 24 h. The transfection medium was replaced with serum-free DMEM, and the cells were left to incubate for 24 h at 37 °C to produce secreted AP fusion proteins. The media containing proteins were collected and concentrated gently at 2,000*g* at 4 °C with intervals of 5 min to prevent protein precipitation until having ~1 ml. NTA Ni resin (250 μl per protein) was equilibrated with 20 mM Tris–HCl pH 7.40, 150 mM NaCl. To 1 ml of AP fusion proteins, washed resin was added, and proteins were rotated with resin for 2 h at 4 °C. After 2-h incubation, protein–resin slurry was transferred to the column. Protein-bound resin was washed with ten bed volumes (~5 ml) of freshly prepared 20 mM Tris–HCl pH 7.40, 150 mM NaCl and 20 mM imidazole. AP fusion proteins were eluted with ten bed volumes (~5 ml) of freshly prepared 20 mM Tris–HCl pH 7.40, 150 mM NaCl and 200 mM imidazole. Eluted fractions were subject to buffer exchange with MWCO 10 kDa (Amicon) to remove high salt at 2,000*g* at 4 °C until reaching ~250 μl. Purified proteins were determined concentrations using a Bio-Rad detergent-compatible protein assay kit (Bio-Rad Life Science #5000113 and #5000114) and stored at −80 °C as 5-μl or 10-μl aliquots for future binding assays.

##### Nogo66–Nogo66R (RTN4–RTNR) AP binding assay

HEK293T or HeLa cells stably expressing GFP-FLAG-His or RTNR-FLAG-His were plated into six-well plates to reach 100% confluency before the assay. Experiments were conducted as previously described^69^. Cells were washed once with a cold HBAH buffer containing Hanks’ balanced salt solution (ThermoFisher #14025092), bovine serum albumin (0.5 mg ml^−1^) (Fisher Scientific #BP1600-100), 0.1% (w/v) NaN_3_, 20 mM HEPES, pH 7.45. AP fusion proteins were diluted in HBAH to reach 100 nM and added to the cells gently. Cells were incubated with protein at 4 °C for 3 h. After incubation, protein solution was removed, and cells were washed five times with cold HBAH with 5-min incubation in HBAH between each wash. Cells were lysed with 200 μl Triton–Tris buffer (1% (v/v) Triton X-100 and 10 mM Tris–HCl, pH 8.0) at room temperature. Lysates were collected into Eppendorf tubes and spun at 3,000*g* for 5 min at 4 °C to remove cell nuclei. Then, 150 μl lysates were transferred to new Eppendorf tubes and heated at 65 °C for 10 min to heat-inactivate endogenous AP. Per 100-μl lysate after heat inactivation, 100 μl of 4-nitrophenyl phosphate (p-NPP) prepared in buffer containing 20 mM homoarginine, 1 mM MgCl_2_ and 21% diethanolamine, pH 10.40, was added to reach 12 mM working concentration. Heat-stable AP activity was detected by measuring absorbance of samples at 405 nM at 37 °C for 3 h using a multimodal plate reader (BioTek Synergy H1).

##### Seahorse XF analysis

All respirometry was conducted in a Seahorse XF96 or XFe96 Analyzer (Agilent Technologies). All experiments were conducted at 37 °C and at pH 7.2. The placement of treatment groups on the XF plate was randomized across biological replicates as best as possible to avoid biased results.

Recombinant, mutant perfringolysin O (rPFO; commercially XF Plasma Membrane Permeabilizer [XF PMP, Agilent Technologies]) was used to selectively permeabilize the plasma membrane of cells. Experiments were conducted as previously described^83,84^. Immediately prior to the assay, cell medium was replaced with MAS buffer (70 mM sucrose, 220 mM mannitol, 10 mM KH_2_PO_4_, 5 mM MgCl_2_, 2 mM HEPES, 0.2% (w/v) Fraction V BSA and 1 mM 4-(2-hydroxyethyl)piperazine-1-ethane-sulfonic acid (EGTA); pH 7.2) containing 2 nM rPFO, 5 mM pyruvate with 1 mM malate and 4 mM ADP. The ADP-stimulated respiration rate (referred to as ‘state 3’ respiration) was measured, and background signal was measured after treatment with 0.2 μM rotenone with 1 μM antimycin A.

When normalizing respirometry experiments to cell number, cells were fixed immediately upon completion of the assay with 2% (w/v) formaldehyde for 20 min at room temperature and kept refrigerated between 1 and 14 days until assessment. On the day before cell counting, cells were stained with Hoechst (Thermo Fisher #33342) at 10 ng ml^−1^ overnight at room temperature. Cell counts were obtained using the Operetta High-Content Imaging System (PerkinElmer).

##### FLAG immunoprecipitation

For ubiquitin assay, HEK293T cells stably overexpressing COX5A–HA were transiently transfected with COX5A–FLAG for 48 h. All other FLAG-immunoprecipitation experiments required parental HEK293T cells transiently overexpressing FLAG-tagged GFP, wild-type COX5A and mutant COX5A (T79G-, T79W-, Y80W- and D81W-COX5A) before asciminib treatment. As annotated in detail per experiment, cells with 90–100% confluency in 10-cm plates were untreated or subjected to 1 h treatment with asciminib (10 μM) or dasatinib (10 μM) with or without 20-min UV irradiation at 350 nm. Treated cells from ubiquitin assay were lysed using the lysis method 5, and all other FLAG-immunoprecipitation-lysed cells by lysis method 1. Both lysis methods and the corresponding determination of protein concentrations were described in detail earlier. For each normalized lysate sample (350 μl, 3 mg ml^−1^) lysates, 10 μl of anti-FLAG EZView resins (Sigma #F2426) were washed with buffer B containing 50 mM Tris–HCl, pH 7.40, 150 mM NaCl and resuspended in buffer B (100 μl). Normalized samples were rotated with prewashed resins for 2 h at 4 °C, with 50 μl of lysate being saved as input samples. After incubation, resins were spun down at 8,200*g* for 1 min., followed by removal of supernatant, and washed three times with buffer B. Elution of immunoprecipitated proteins was done by adding 4× Laemmli loading dye containing no 10% β-mercaptoethanol (30 μl) to each sample and heating the samples for 5 min at 95 °C. To each input sample, 16 μl of 4× Laemmli sample buffer with 10% β-mercaptoethanol was added, and the mixture was boiled at 95 °C for 5 min. Eluted samples were subjected to SDS–PAGE and western blot analyses as described earlier in the ‘SDS–PAGE analysis and imaging’ and ‘Western blots’ sections.

##### BN-PAGE analysis to study OXPHOS complex assembly

Parental HEK293T cells at 90% confluency in 10-cm plates were treated with DMSO as vehicle, dasatinib (10 μM) or asciminib (10 μM) for 6 h at 37 °C and 5% CO_2_. Treated cells were collected and stored as cell pellets at −80 °C until usage. Experiments were conducted as previously described^85^. Cell pellets were thawed on ice, resuspended in PBS-Digitonin 25% (8 mg ml^−^^1^) (400 μl) and incubated for 10 min. Samples were centrifuged twice for 5 min at 10,000*g* at 4 °C. Pellets were resuspended in 1.5 M aminocaproic acid, 50 mM Bis-Tris/HCl pH 7.0 and 1% digitonin. Samples were centrifuged for 30 min at 20,000*g* at 4 °C. Supernatant was collected and resuspended with sample buffer prepared with 0.75 M aminocaproic acid, 50 mM Bis-Tris–HCl pH 7.0, 0.5 mM EDTA and 5% SERVA Blue G. Samples were loaded into native PAGE 3–12% (Invitrogen #BN1003BOX), run for 3 h at 150 V and underwent western blot analysis. Antibodies used in this experiment include COXIV (Proteintech, #11242-1-AP, #00110030) and SDHA (Invitrogen, #459200 and #YB3840708).

##### Cell line sources

All cell lines used were purchased from ATCC: HEK293T (ATCC, CRL-3216), HeLa (ATCC, CCL-2), K562 (ATCC, CCL-243), KCL22 (ATCC, CRL-3349) and MOLT4 (ATCC, CRL-1582).

##### Protein structural analysis

Depictions of protein structures obtained directly from the Protein Data Bank (PDB) were generated using the licensed PyMOL Molecular Graphics System, Version 2.5.5 Schrödinger, LLC.

### Reporting summary

Further information on research design is available in the Nature Portfolio Reporting Summary linked to this article.

## Online content

Any methods, additional references, Nature Portfolio reporting summaries, source data, extended data, supplementary information, acknowledgements, peer review information; details of author contributions and competing interests; and statements of data and code availability are available at 10.1038/s41557-026-02127-4.

## Supplementary information

Supplementary Information Supplementary Notes 1–8, Supplementary Figures 1–7, Supplementary Schemes 1–4, synthetic procedures, nuclear magnetic resonance spectra and Supplementary Tables 1, 3, 8 and 11.

Reporting Summary

Supplementary Table 2 Proteomics data for Fig. 1 and related extended and/or supplementary figures.

Supplementary Table 4 Proteomics data for Fig. 2 and related extended and/or supplementary figures.

Supplementary Table 5 Proteomics data for Fig. 3 and related extended and/or supplementary figures.

Supplementary Table 6 Proteomics data for Fig. 4 and related extended and/or supplementary figures.

Supplementary Table 7 Comparison of kinases labelled by asciminib and dasatinib probes.

Supplementary Table 9 Proteomics data for Fig. 5 and related extended and/or supplementary figures.

Supplementary Table 10 Complete list of raw proteomics datasets.

## Source data

Source Data Fig. 1 Statistical Source Data.

Source Data Fig. 2 Statistical Source Data.

Source Data Fig. 3 Statistical Source Data.

Source Data Fig. 5 Statistical Source Data.

Source Data Extended Data Fig. 3 Statistical Source Data.

Source Data Extended Data Fig. 4 Statistical Source Data.

Source Data Extended Data Fig. 9 Statistical Source Data.

Source Data Extended Data Fig. 10 Statistical Source Data.

Source Data Fig. 1 Unprocessed gels.

Source Data Fig. 3 Unprocessed gels.

Source Data Fig. 5 Unprocessed gels and western blots.

Source Data Extended Data Fig. 5 Unprocessed gels and western blots.

Source Data Extended Data Fig. 7 Unprocessed gels.

Source Data Extended Data Fig. 9 Unprocessed gels and western blots.

Source Data Extended Data Fig. 10 Unprocessed gels and western blots.

## Data Availability

The MS data have been deposited to the ProteomeXchange Consortium (http://proteomecentral.proteomexchange.org) via the Proteomics Identification Database (PRIDE) partner repository with the dataset identifiers PXD068136, PXD068139, PXD068140 and PXD070048. Source data are provided with this paper.

## Code availability

MSFragger can be downloaded as a single JAR binary file at https://msfragger.nesvilab.org/. FragPipe is available via GitHub at https://github.com/Nesvilab/FragPipe (ref. ^86^). Custom code for LFQ analysis, titled ‘LFQ-kinase_MBR_CN’, is available via GitHub at https://github.com/BackusLab/LFQ-kinase_MBR_CN.git (ref. ^87^).
