# Supplementary material for: Small-molecule binding-site discovery using silyl ether-enabled chemoproteomics
Source: Nat Chem. 2026 Apr 27;18(8):1431–42. doi: 10.1038/s41557-026-02127-4 (PMC13423832; doi:10.1038/s41557-026-02127-4)

nature chemistry

Supplementary information

https://doi.org/10.1038/s41557-026-02127-4

Small-molecule binding-site discovery using silyl ether-enabled chemoproteomics

In the format provided by the authors and unedited

Table of Contents

(A)	Supplementary Notes 1–8	2-5
(B)	Supplementary Figures and Schemes	5-15
(C)	Supplementary Tables	15-19
(D)	Synthetic Procedures	19-29
(E)	Docking Methods	29
(F)	NMR Spectra	30-66
(G)	Supplementary References	67-68
(H)	Source Data for Supplementary Figures	69-72

(A) Supplementary Notes

Supplementary Note 1: PAL-tailored mass offset search-based workflow with hyperscores. We implemented a PAL-tailored mass offset search-based workflow (released in FragPipe 22 under the name 'PAL'). In this workflow, the SEE-CITE modification masses were specified as mass offsets (allowed on any amino acid), whereas Met oxidation and N-term acetylation are still specified as variable modifications. Parameter optimization was disabled and the number of peaks to use in MSFragger was increased to 500, as these options were determined to provide better localization. Deisotoping in MSFragger was enabled (unless otherwise noted). MSBooster and Percolator were used for rescoring unless otherwise noted (in which case, PeptideProphet was used instead). Several additional modifications were made. MSBooster[1] was modified to model the retention times of unmodified and mass shifted peptides separately.

Importantly, MSFragger was extended to compute and report the following new scores to fully explore different types of fragments that can be observed in MS/MS spectra of labeled peptides:

1. "Score all unshifted": the hyperscore when matching only unmodified fragments. This score assumes that the modification is fully labile.

2. "Score best position": the hyperscore when putting the modification on the best site. This assumes that the modification is not labile.

3. "Score second best position": the hyperscore when putting the modification on the second-best site.

4. "Score shifted all positions": the score computed based on all possible shifted fragments. This score covers the case in which peptidoforms with modifications at different sites are co-fragmented.

5. "Score shifted best position": the score when putting the modification on the best site and matching only the modified fragments.

6. "Position scores": a string with peptide sequence and scores when putting the modification on the specific site.

7. "Shifted only position scores": similar to the "position scores" but only matching the modified fragments.

Hyperscores are reported for each modification position within the peptide sequence, computed using all ions, and separately using shifted and unshifted ions only—this latter feature is particularly beneficial for sequences with few shifted ions due, for example, to more labile modifications. MSFragger also calculates the number of matched ions corresponding to each of the above scores. These scores can be used to evaluate localization confidence and labile levels. If "score all unshifted" is higher than "score best position", then the modification is likely to be fully labile. If "score best position" is much higher than "score second best position", the modification can be confidently localized on a specified site. If "score shifted all position" is fairly high, the spectrum is highly multiplexed. There are peptides with modifications at different sites. "position scores" and "shifted only position scores" can be used to measure where the modification is localized.

Supplementary Note 2: Advantages of the hyperscoring approach. Illustrating the benefits of the hyperscoring approach, for the **2a**-modified peptide AYLESEVAISEELVQK RTN4 peptide, we find that our method localizes the region of the modification to residues 1A2Y (**Fig. 2C,D**), based on the combined weighted scores of the three total shifted b ions and five total unshifted y ions

that lack the crosslinked modification. If the modification is then manually placed on the 11E residue, which has a lower hyperscore, as visualized using the FragPipe integrated PDV viewer[2] shows, a decrease in overall matched ions is observed, consistent with this alternative residue as not a likely labeling site. An even more compelling example is the NVFENPTMVQFDHR peptide from the COX15 subunit of cytochrome C oxidase, for which complete sequence coverage is obtained and the MSFragger hyperscore clearly delineates the likely labeling site as 11D residue, with loss of the Y_2 ion if the modification is placed on 12H (**Fig. 2E,F**).

Supplementary Note 3. Assessing the impact of deisotoping on photocrosslinking localization. Manual inspection of spectra (**Supplementary Fig. 2C**) revealed several added complexities for achieving high confidence localization. For VAQLYADLDggfshaawlLPGWLPLPSFR peptide from lanosterol 14-alpha demethylase (CYP51A1), MSFragger placed the modification within the 10G-18L region. However, placing the modification on the either 4L or 5Y residue when viewing the spectra in PDV, allowed for an additional shifted b_5 and b_7 ions to be matched, with loss of the corresponding unshifted y_{20} ion. This alternative localization, while lower scoring, represents a plausible additional or alternative site for the SEE-CITE modification. Further illustrating the complexities of pinpointing the localization, we observed for AADKGVYIIgssgfdSIPADLGVIYTR peptide from saccharopine dehydrogenase-like oxidoreductase (SCCPDH) that placing the modification on the 15D residue using PDV gained matches for the b_{14} and y_{13} ions (**Supplementary Fig. 3A**). As Y_{13} was doubly charged and b_{14} triply charged, we posited that we could recover these matches by searching with MSFragger without spectral deisotoping. This hypothesis proved correct (**Supplementary Fig. 3B**), but came at the expense of a slight, but not significant ~5-10% loss in sequence coverage (**Supplementary Fig. 2B**). Thus, we opted to proceed with deisotoping (the default option in MSFragger) as it afforded increased coverage, despite a slight decrease in the ability to localize modifications on the peptide that resulted from fewer matches for multiply charged ions.

Supplementary Note 4. Synthesis of dasatinib and asciminib PAL probes. For dasatinib, we first synthesized both alkyl- and carbamate-linked conventional PAL probes **4a** and **4b** that lack the SEE-CITE group, to serve as benchmarking probes (**Fig. 3A** and **Supplementary Fig. 5**). Dasatinib SEE-CITE probe **4c** was similarly obtained by alkylation of the piperazine core with alkyl bromide silyl ether diazirine handle **7** (**Supplementary Scheme 2**). For asciminib, we initially pursued ether-linked probes (**Fig. 3A, Extended Data Fig. 5A,B,** and **Supplementary Scheme 3**). However, In contrast to our synthesis of the dasatinib probes the alkylation strategy failed to afford appreciable amounts of our desired probes. Therefore, we instead opted to install the diazirine handle via a carbamate linkage, which proceeded smoothly to furnish asciminib PAL probe **5a.** As the synthesis of the corresponding SEE-CITE probe was initially hampered by difficulties in obtaining the requisite amine-containing silyl ether diazirine intermediate, we revised our strategy, which yielded asciminib SEE-CITE probe **5c** with a reverse carbamate linkage (**Fig. 3A, Extended Data Fig. 5A,B,** and **Supplementary Scheme 3**).

Supplementary Note 5. Difference in labeling by the SEE-CITE and parent PAL probes. For the dasatinib analogues, the carbamate probe 4**b** showed increased overall gel-based labeling when compared to the alkyl probes **4b** and **4c,** consistent with the reduced charge improving cell penetration (**Extended Fig. 5E**). Probe **4b**, which features the carbamate linkage showed markedly increased labeling when compared with alkyl analog **4a** or **4c**. SEE-CITE asciminib probe **5c** showed similar banding patterns to **5a** and **5b** indicating similar target profiles, albeit at an increased compound dose required to achieve comparable labeling in both KCL-22 and K562 cells (**Extended Fig. 5E**). As we encountered unexpected synthetic challenges in isolating the carbamate dasatinib analogue (**Supplementary Scheme4**), we opted to also compare carbamate-linked scout probe **3** to **5c** to further bolster our SEE-CITE analysis of asciminib using

structurally matched compounds. Gel-based comparison of scout fragment **3** to **5c** revealed several distinct bands, indicating some probe-specific SAR (**Extended Fig. 5F**).

Supplementary Note 6. Search settings for Abl1 site of labeling. We subjected Abl recombinant protein spiked into cell lysates to SEE-CITE and compared the relative labeling by **4c** versus **5c** each at 1 μM. IonQuant MS1 ratios were quantified for 43 total modified peptides (**Supplementary Table 5**). We then compared the labeling sites identified using the variable modification workflow (**Supplementary Fig.6A-C**) to those generated using our new PAL mass offset based workflow (**Fig. 3D**). Our objectives were to assess both the performance of the new workflow and to confirm the SEE-CITE's fidelity for capturing known binding sites.

Our variable modification search analysis revealed eleven labeled sites within five peptide sequences that showed strongly preferential labeling by dasatinib probe **4c**, as indicated by the strongly negative ratios (**Fig. 3E**). Our offset search workflow initially identified four of the five labeled peptides, localizing the modifications to nearly identical labeling regions within the peptides, with E258 as the most frequently labeled residue. The new scores we implemented in MSFragger for the mass offset workflow highlight likely ambiguous labeling, reporting G372-E373 as labeled rather than only G372, which was identified as SEE-CITE modified by MSFragger in the variable modification search mode (**Supplementary Fig.6A**). By turning off deisotoping in MSFragger and using Percolator without MSBooster (or, alternatively, using PeptideProphet[3] for rescoring), we were able to recover the final labeled peptide, with modification likely occurring at D381, although V379 cannot be ruled out as an alternative labeling site (**Fig. 3D,E**, **Supplementary Fig.6B**). All of these labeled sites are located proximal to the ATP binding site (**Fig. 3F** and **Supplementary Fig.6C**, highlighted in red), consistent with dasatinib's active site-directed mode of inhibition. Similarly, we identified three residues with highly positive MS1 ratios as computed by IonQuant (**Fig. 3E**), indicative of asciminib preferential binding (**Supplementary Fig.6C**, P480/E481/E526). These same three sequences were also identified with the mass offset search method (**Fig. 3D**), with E481/P480-C483/E526 as the most labeled sites. Gratifyingly, all three of these sequences are located proximal to the allosteric myristoyl pocket engaged by asciminib (**Fig. 3F**). Thus, the highly precise compound-specific delineation of these known compound binding sites provides a compelling case for the use of SEE-CITE in proteome-wide binding site analysis.

In addition to this compelling data, while conducting these analyses, we did make several additional observations that apply more generally to diazirine site-of-labeling proteomics. Consistent with our amino acid analysis with our scout probe **(Fig. 1E** and **Fig. 2I**), we also observe a trend towards increased identification of acidic residues as the likely labeling sites in the mass offset search mode compared to the variable modification search (**Fig. 3D** and **Supplementary Fig.6A**). These findings hint that in complex biological mixtures alkyl diazirines likely show marked preference for acidic residues rather than reacting equally across all 20 amino acids. Additionally, for the mass offset searches, we did observe decreased coverage of some labeled peptides when MSFragger was coupled with rescoring using MSBooster and Percolator; we were able to recover these sequences using PeptideProphet instead. We found these modifications to be particularly relevant for datasets with large numbers of contaminating unmodified peptides, which we found to be the case for our ABL1 datasets that were prepared using the low 1 μM probe concentration.

Supplementary Note 7. Establishing probe-probe comparisons via SEE-CITE. To start our site-of-labeling campaign, we assessed relative probe labeling (**Extended Data Fig. 5F and Extended Data Fig. 7A**) and adjusted probe concentrations in an effort to account for the different general protein labeling activities (**3 = 2a > 5c > 4c**). We then generated three probe-probe SEE-

CITE comparison datasets (**4c b 5c**, **5c** vs **2a**, and **5c vs**; **Fig. 5A-C**, **Extended Data Fig. 7B,C**, and **Supplementary Table 9**). While our efforts to match labeling efficiency were successful for our **5c** vs **3** comparison, which showed a median ratio close to $Log_2(H/L) > 0$, we did note some differences in labeling for our **4c** vs **5c** and **5c** vs **2a** comparisons, which could be attributed to a combination of several factors, such as intramolecular quenching of diazirine and differences in cellular permeability and subcellular localization for each probe.

Supplementary Note 8. DFG motif is highly homologus in kinases, which complicates site-of labeling analysis. Analysis of the kinases captured by the proteome-wide SEE-CITE analysis (**Supplementary Table 9)** revealed one unexpected challenge with such kinase-directed site-of-labeling analysis, namely the high sequence conservation of some kinase active sites, as exemplified by the modified tryptic peptide (I(L)AD*FGLAR, with isoleucine and leucine being isobaric and therefore indistinguishable) from the kinase DFG loop, which is shared by 27 human protein kinases. For ABL1, the DFG motif sequence is only shared with ABL2 and, more broadly, most observed labeled peptides are unique, which indicates that, while SEE-CITE users should be aware of such non-unique sequences, most labeling sites should be unimpacted by this potential confounder.

(B) Supplementary Figures

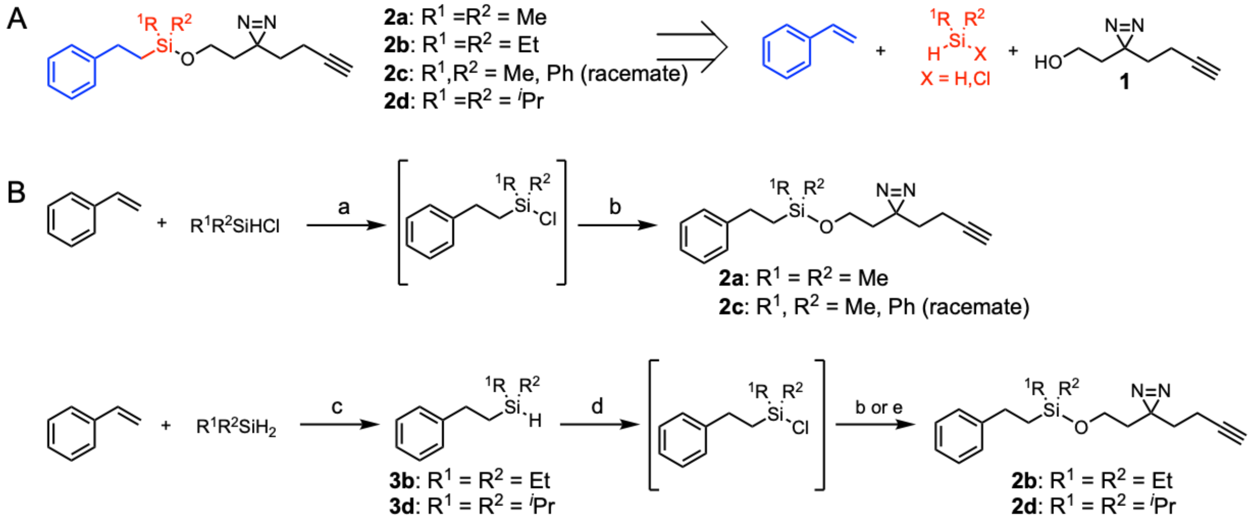

Supplementary Scheme1. **Design and synthesis of the prototype SEE-CITE probes 2a-d**. **A**, Design of the four prototype SEE-CITE probes from styrene. **B**, Reagents and conditions: a. Karstedt's catalyst, neat, 0 °C to rt; b. **1**, NEt_3, CH_2Cl_2, 0 °C , 41% (for **2a**, 2 steps from styrene), 63% (for **2b**, 2 steps from **3b**) 87% (for **2c**, 2 steps from styrene); c. $B(C_6F_5)_3$, CH_2Cl_2, rt - 40 °C , 86% (for **3b**), 60% (for **3d**); d. trichloroisocyanuric acid, CH_2Cl_2, 0 °C to rt; e. **1**, imidazole, CH_2Cl_2, 0 °C , 57% (for **2d**, 2 steps from **3d**). While synthesis of most of the scout probes proceeded smoothly with NEt_3 as base, for the bulkier compound **2d,** imidazole was requisite as base for high yield silyl ether formation.

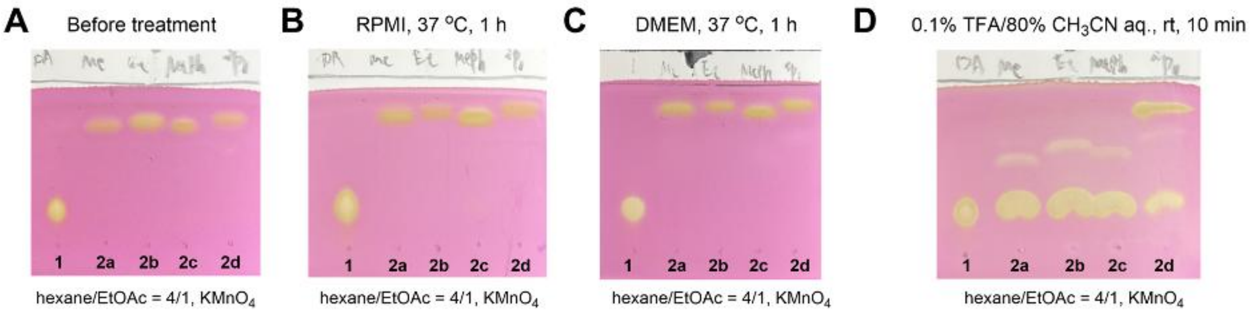

Supplementary Fig.1. **Stability of the prototype probes** - TLC analysis of the stability of the prototype probes **A**, prior to treatment under **B**,**C**, cellular experimental or **D**, elution from neutravidin condition. Each probe was put in an Eppendorf tube and was mixed with the corresponding medium (10 mM concentration) and subjected to the indicated temperature and time, with 1,000 rpm shaking for ‘B’ and ‘C’ **(**FBS- and antibiotics-free media**)**. After that, the mixture was partitioned with ether and water, then the organic layer was monitored by TLC with $KMnO_4$ staining reagent. All probes were stable enough under general cellular labeling conditions ‘B’ and ‘C’. All probes, except **2d,** were completely cleaved under the elution condition from neutravidin beads in ‘D**’**.

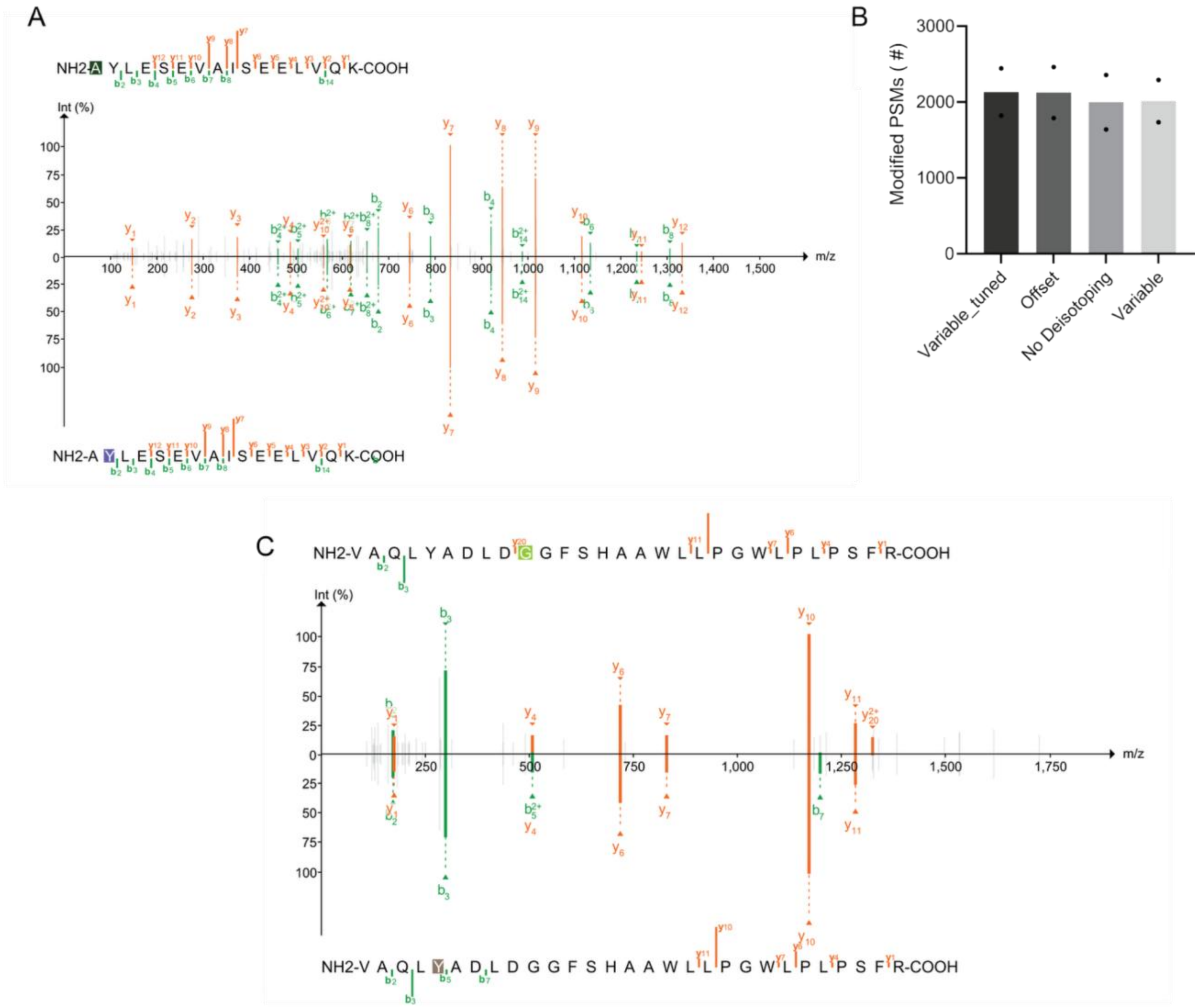

Supplementary Fig.2. Establishing and testing hyperscore analysis in FragPipe. **A**, Example MS/MS spectra generated with PDV[2] showing localization of SEE-CITE modification for AYLESEVAISEELVQK peptide from RTN4. Top spectra shows the 1A SEE-CITE (+436.22565) localization placed by MSFragger and bottom shows matches when the SEE-CITE modification is manually placed on the 2Y position using the PDV. **B**, Comparison of coverage, PSMs, obtained after re-searching datasets from **Extended Data Fig. 3** (entry 6, n=2 biological replicates), using the indicated MSFragger search modes. **C**, Example MS/MS spectra for VAQLYADLDggfshaawlLPGWLPLPSFR peptide from Lanosterol 14-alpha demethylase (CYP51A1) with MSFragger hyperscore indicating likely localization within the 'ggfshaawl' sequence (top). Alternative localization site on 4L or 5Y visualized in PDV (bottom). All data can be found in **Supplementary Table 4**.

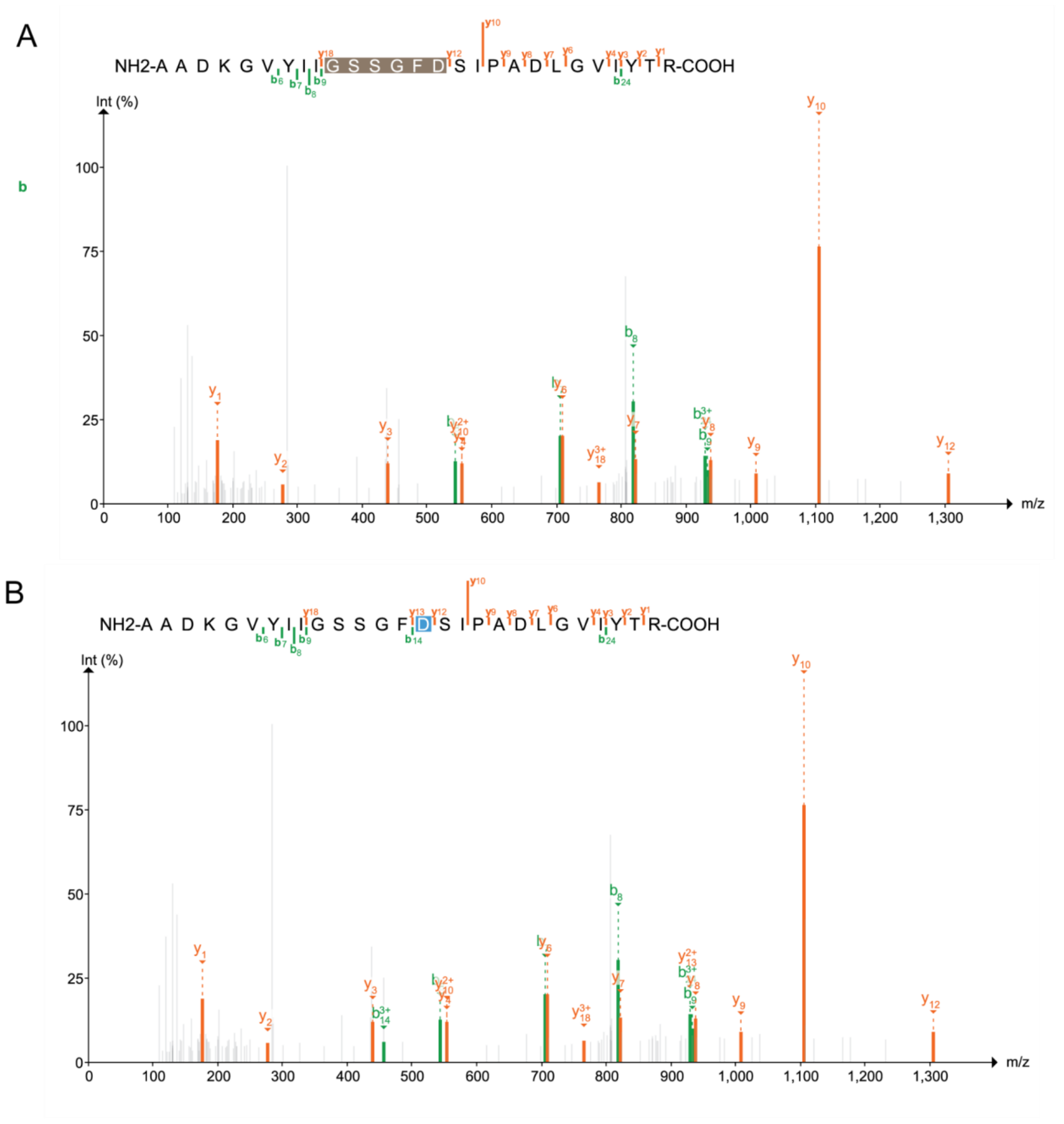

Supplementary Fig.3. Example MS/MS spectra for AADKGVYIIgssgfdSIPADLGVIYTR peptide from Saccharopine dehydrogenase-like oxidoreductase (SCCPDH) shows alternative localization site on 15D. **A**, shows localization to the gssgfd region and **B**, shows localization to the 15D residue identified with MSFragger search without deisotoping. All data can be found in **Supplementary Table 4**.

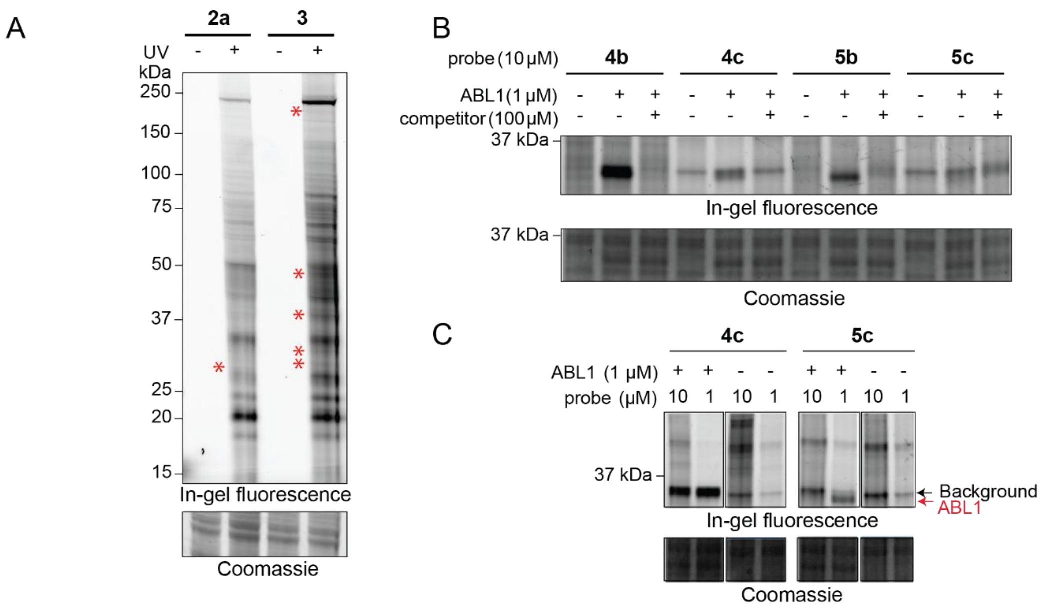

Supplementary Fig.4. Assessing cell-based labeling and kinases inhibitory activity for SEE-CITE probes A, Gel-based A*f*BPP of HEK293T cells treated with scout probes **2a** or **3** (20 μM, 1 h). **B,C**, Competitive gel-based A*f*BPP analysis comparing labeling of ABL1 kinase domain of by dasatinib probes **4b** or **4c** versus asciminib probes **5b** or **5c** (10 μM, 1 h) using recombinant ABL1 (1 μM) in lysates (1 mg/mL). For 'B' higher probe concentration results in labeling of a protein band co-migrating with Abl1 for **5c.** Lower probe concentration shown in 'C' shows maintained labeling for Abl1 and decreased labeling for the co-migrating band for **5c**, allowing the two bands to be resolved and confirming on-target labeling by **5c.** Data presented are from n=1 independent experiment for 'A' and n=2 independent experiments for 'B-C'.

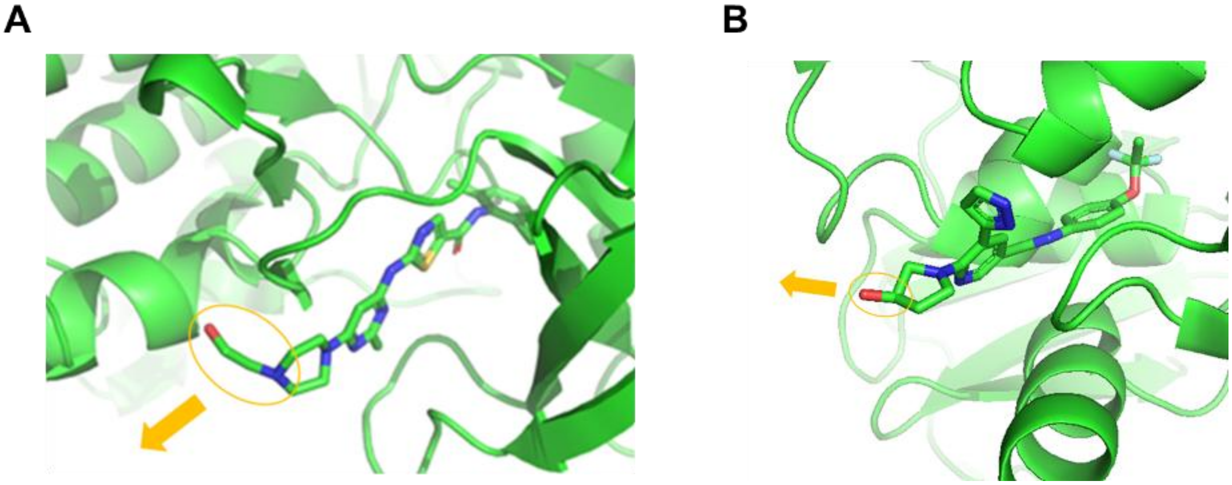

Supplementary Fig.5: X-ray Crystal Structure of kinase inhibitor bound ABL kinase domain. A, Dasatinib (BMS-354825) Bound to Activated ABL Kinase Domain (PDB: 2GQG). **B**, ABL1 kinase (T334I_D382N) in complex with asciminib and nilotinib (PDB: 5MO4). Yellow arrows show the vectors for installing diazirine linkers.

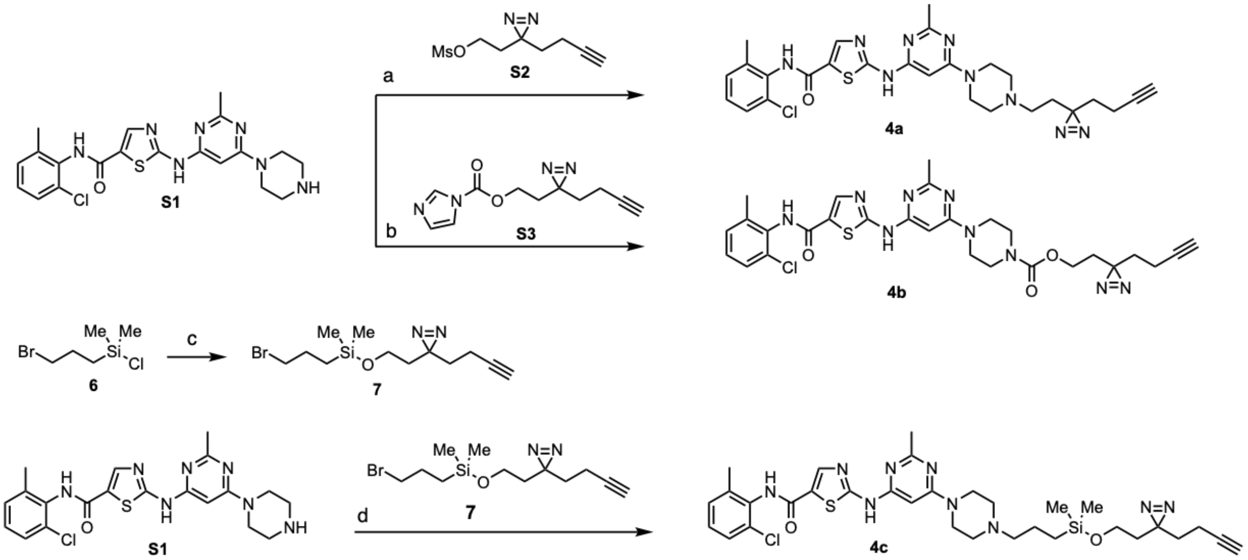

Supplementary Scheme2: Synthetic scheme of dasatinib probes. Reagents and conditions: a. DIPEA, DMF, 80°C, 35%; b. Compound **1**, DIPEA, DMF, 60°C, 28%; c. NEt_3, CH_2Cl_2, 0°C, 75%; d. NaI, DIPEA, DMF, 50°C, 47%.

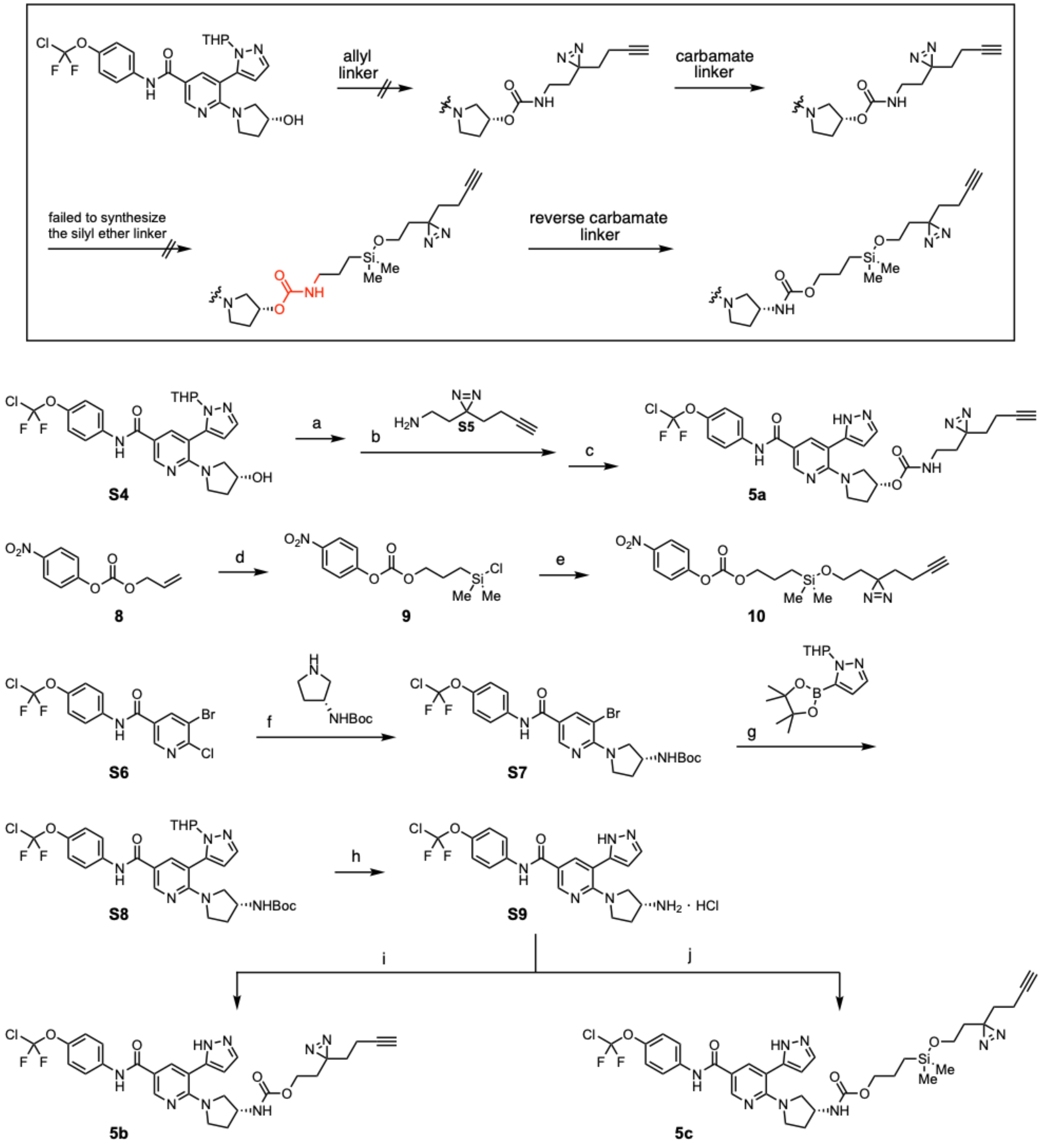

Supplementary Scheme3. **Synthetic scheme of asciminib probes**. Reagents and conditions: a. 4-nitrophenyl chloroformate, NEt3, CH2Cl2, 0°C, generated in situ for (b); b. NEt3, DMF, rt; c. TFA, CH2Cl2, rt, 74% for steps (b-c); d. [Ir(cod)Cl]2, 1,5-cyclooctadiene, chlorodimethylsilane, neat, 75-80°C, 96%; e. Compound 1, NEt3, CH2Cl2, 0°C, 81%; f. DIPEA, DMSO, 120°C, 98%; g. Pd(PPh3)4 (5 mol%) , K3PO4, toluene, 110°C, 51%; h. 4N hydrochloric acid/1,4-dioxane, CH2Cl2, rt, 99%; i. Compound S3, DBU, DMF, rt, 49%; j. Compound 10, DMAP, DIPEA, CH2Cl2, rt, 31%.

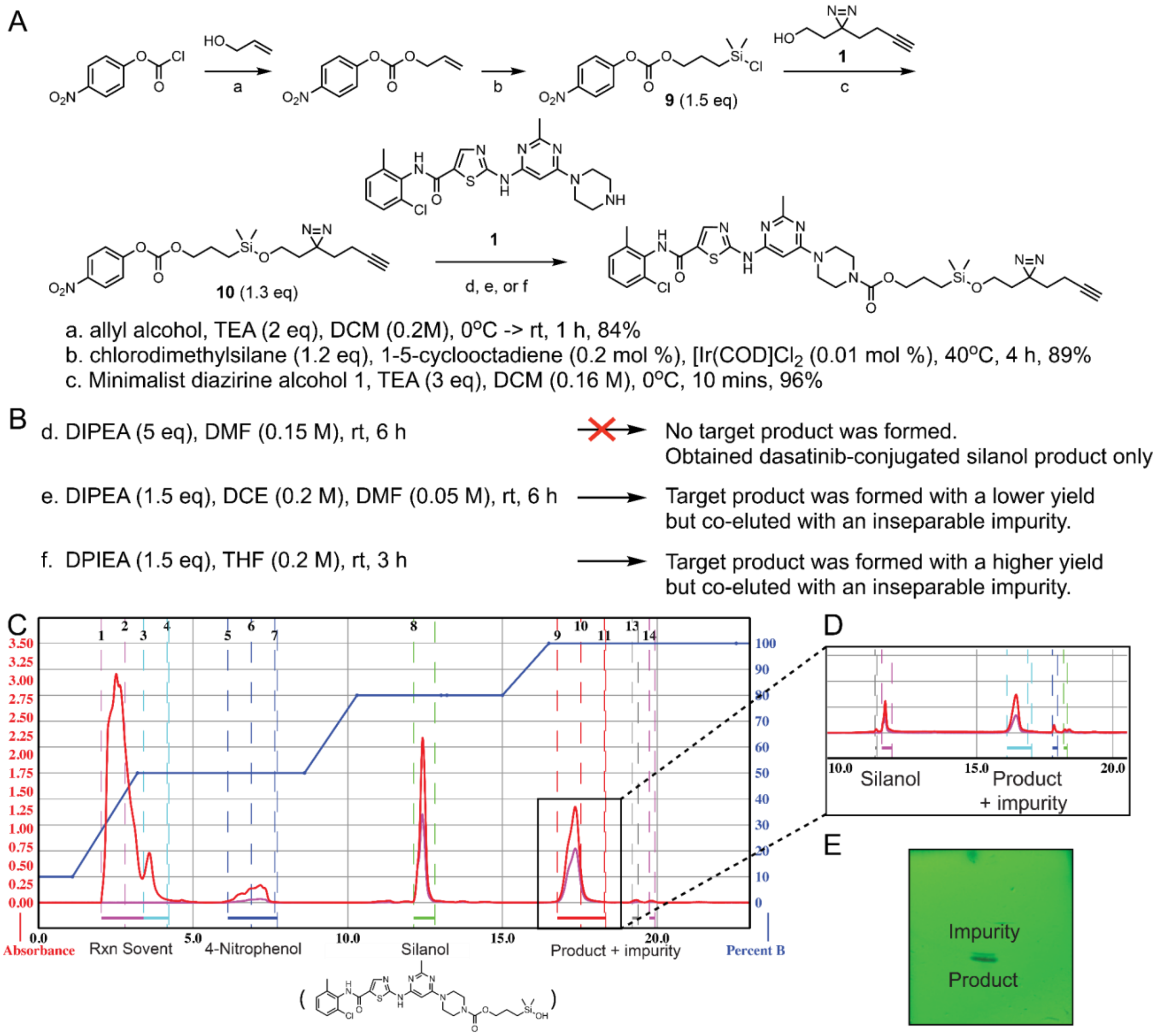

Supplementary Scheme4. **Synthetic and purification efforts toward carbamate dasatinib probe. A**, Scheme of the synthetic route. **B**, Reaction conditions attempted for generating carbamate dasatinib probe. **C**, Preparative High-Performance Liquid Chromatography (Prep-HPLC) trace shows co-elution of the target dasatinib probe and an inseparable impurity at 100% B-acetonitrile, confirmed by ^{1}H-NMR. **D**, Prep-HPLC analysis of the collected product-impurity mixture yielded the cleaved silanol (structure shown) and once again the product-impurity mixture. The red trace is for absorbance at 214 nm, and the purple trace is for absorbance at 254 nm. **E**, Subjecting the product-impurity mixture to manual column chromatography also failed to isolate carbamate dasatinib probe from the impurity due to their highly similar Rf values in various solvent pairs (dichoromethane (DCM)/5-10% methanol (MeOH) and hexane/ethyl acetate (in varying volumetric proportions)), depicted here by the Thin Layer Chromatography analysis in DCM/ 10% MeOH.

A

Log$_2$(H/L)	Preferential Probe	ABL1 Peptides (Total Spectral Count from 3 Replicates)	Most Frequently Modified Residue
-7.95	**4c** (DA)	HKLGGGQY(2)GEVYE(7)G(1)VWK	E258
-6.27	**4c** (DA)	LGGGQYGEVY(6)E(23)GVWK	E258
-6.04	**4c** (DA)	LGGGQYG(1)EVY(6)E(15)GVWKK	E258
-3.8	**4c** (DA)	NC[57.0215]LVG(5)ENHLVK	G372
-3.71	**4c** (DA)	V(2)AD(3)FGLSR	D381
4.29	**5c** (AS)	MERP(1)E(3)GC[57.0215]PEK	E481
4.21	**5c** (AS)	MERP(1)EGC[57.0215]PEKVYELMR	P480
7.83	**5c** (AS)	AC[57.0215]WQWNPSDRPSFAEIHQAFETMFQESSISDE(9)VEK	E526

B

Custom Mass Offset Workflow	ABL1-modified Peptides by 4c (Net PSMs from 3 Replicates)	Maximum Hyperscores for Identified PSMs	Most Frequently Modified Residue
Percolator with no deisotope	vADFGLSR(1)	18.76	V379
Peptide Prophet with no MSBooster	VAdFGLSR(2)	15.23; 16.92	D381

C

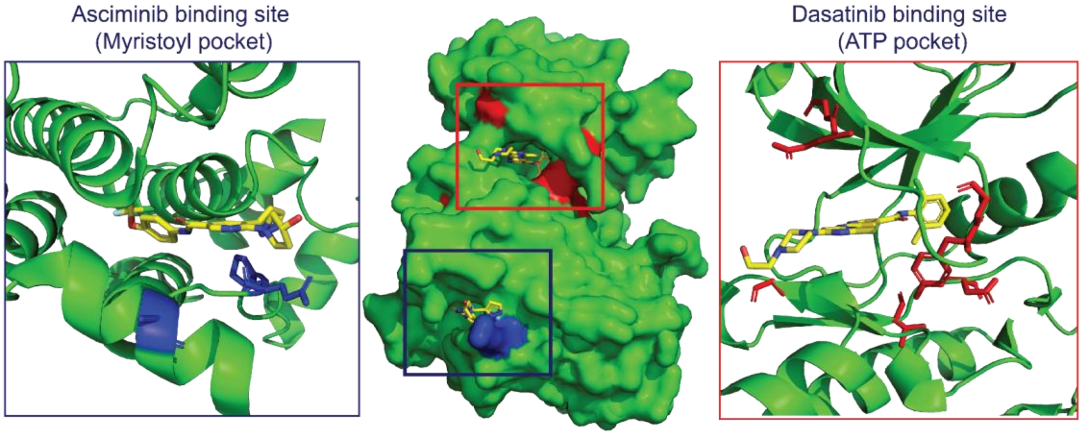

Supplementary Fig.6. **Quantitative comparison of labeling sites of ABL1 identified by MSFragger variable modification search using SEE-CITE probes 4c (dasatinib, light biotin-azide, red) and 5c (asciminib, heavy biotin-azide, red). A**, Summary of labeling sites identified for the indicated peptides, together with the log2 (H/L) ratios. If the log2(H/L) value was positive, the number of times 442.26332 modifications by **5c** was counted for each amino acid residue. Similarly, if the log2(H/L) was <0, the number of times 436.22566 modifications by **4c** was counted for each amino acid residue. Log2(H/L) values were calculated as the average of 3 replicates excluding singleton (heavy- or light-only labeled peptides). **B**, Comparison of localization obtained using MSFragger mass offset workflow in FragPipe with either MSFragger (without deisotoping) and Percolator, or MSFragger (with deisotoping) and PeptideProphet. **C**, Structures of ABL1 kinase domain co-crystallized with each inhibitor (PDB: 2GQG for ATP pocket and 5MO4 for myristoyl pocket, respectively) and with the colored amino acids corresponding to the SEE-CITE labeled residues with colors corresponding to the labeling indicated in ‘A’. All MS data can be found in **Supplementary Table 5**.

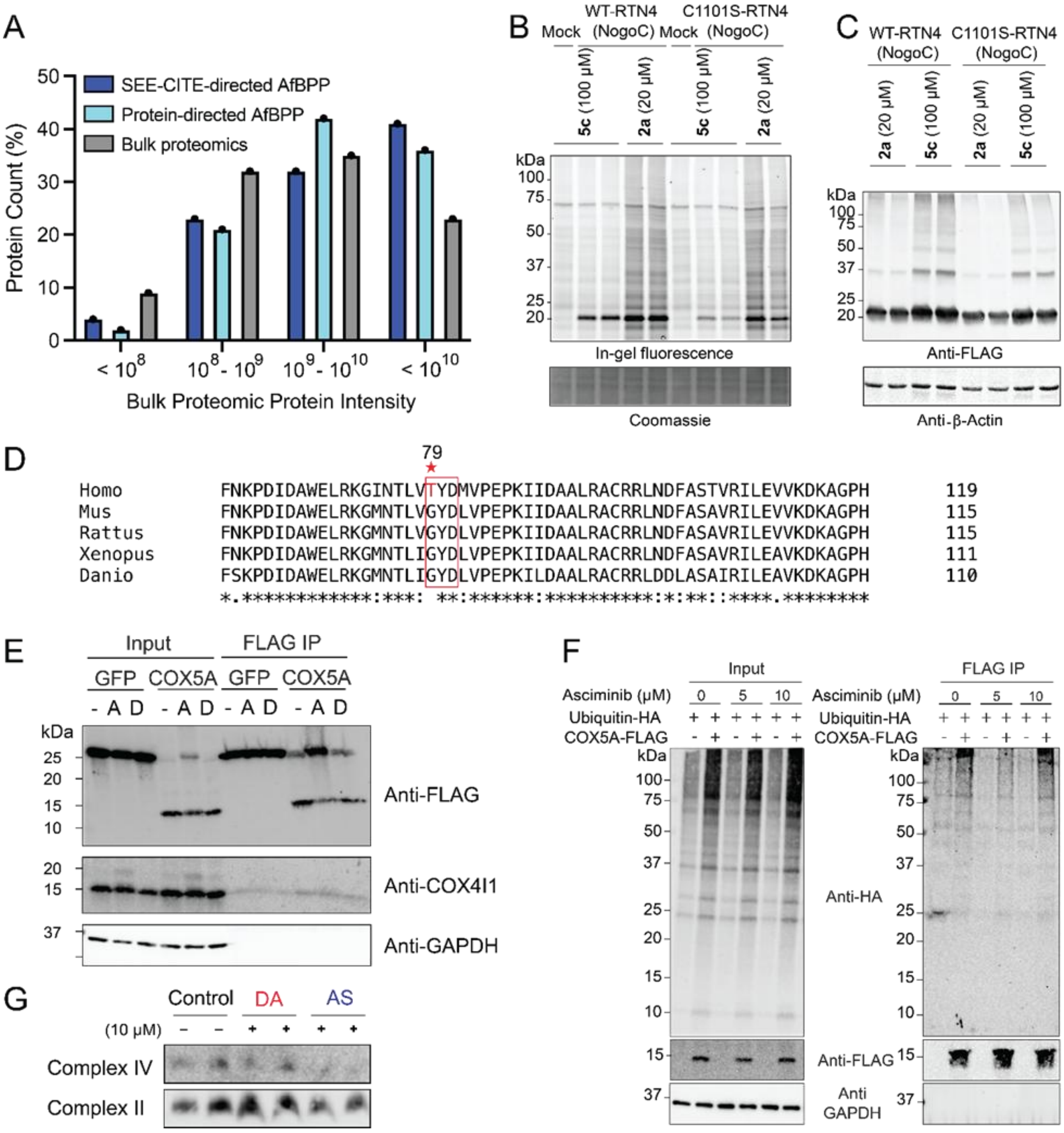

Supplementary Fig.7. **A**, Distribution of proteins identified by each method binned by bulk protein intensity (n=3 biological replicates). SEE-CITE datasets used from Figure 5B,C, Extended Fig. 7, Extended Fig. 8 and Extended Fig. 9 and protein-directed AfBPP with **5c** (10 μM 1h) from Figure 4F and Extended Fig. 6F against bulk proteomics in K562 cells. **B**, Gel-based AfBPP analysis of HEK293T cells transiently overexpressing WT or C1101S treated with **2a** (20 μM, 1 h) and **5c** (100 μM, 1 h). **C**, Immunoblotting to validate the expression of FLAG-tagged WT- or C1101S-RTN4 in HEK293T cells. **D**, Protein sequence alignment of COX5A across species (Clustal Omega[4]). **E**, FLAG-immunoprecipitation of HEK293T cells transiently overexpressing COX5A-FLAG treated with vehicle (-), asciminib (A) or dasatinib (D) (10 μM, 1 h). **F,**) Co-

immunoprecipitation of COX5A-HA overexpressing HEK293T stable cells which were transiently transfected with COX5A-FLAG, treated with asciminib (5 μM, 10 μM, 1h). **G**, Representative immunoblot (n=2) for BNGE in 'Extended Fig. 10F'. Data presented are from n=1 independent experiment for 'B-C', n=2 independent experiments for 'E,G', and n=3 independent experiments for 'F'.

(C) Supplementary Tables

Supplementary Table 1. Stability of the prototype probes under neutravidin enrichment conditions.

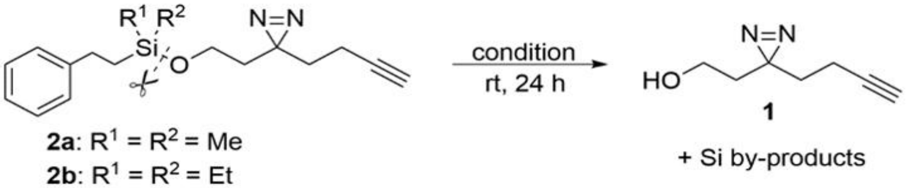

2a: $R^1 = R^2$ = Me
2b: $R^1 = R^2$ = Et
2c: R^1, R^2 = Me, Ph (racemate)

Entry	Probe	Condition	Cleavage was observed?
1	**2a**	MeOH/sat.NaHCO$_3$ aq.	Yes
2	**2b**	↓	Yes
3	**2c**	↓	Yes
4	**2a**	PBS	No
5	**2b**	↓	No
6	**2c**	↓	No
7	**2a**	1% SDS in PBS	Yes
8	**2b**	↓	Yes[a]
9	**2c**	↓	Yes[a]
10	**2a**	6M urea in PBS	No
11	**2b**	↓	No
12	**2c**	↓	No

a. A tiny amount of cleavage

Supplementary Table 3. Solvents and gradients used for LC-MS/MS data acquisition.

Parameter	Condition	
Column	100 μM ID fused silica capillary packed in-house with bulk C18 reversed phase resin (particle size, 1.9 μm; pore size, 100 Å; Dr. Maisch GmbH)	
Mobile phase	Buffer A: water with 3% DMSO and 0.1% formic acid<br>Buffer B: 80% acetonitrile with 3% DMSO and 0.1% formic acid	
Gradient and flow rate	For 70 min<br>0 - 5 min, 3- 20% B, 300 nL/min<br>5 - 64 min, 20 - 55% B, 220 nL/min<br>64 - 66 min, 55 - 95% B, 250 nL/min<br>66 - 70 min, 95% B, 250 nL/min	For 140 min<br>0 - 6 min, 3 - 20% B, 300 nL/min<br>6 - 130 min, 20 - 38% B, 220 nL/min<br>130 - 140 min, 38 - 95% B, 250nL/min
Run time	70 minutes or 140 minutes	
Injection volume	5 uL	

Supplementary Table 8. **A shortlist of high-affinity kinase off-target candidates of dasatinib.** Selected kinases for comparison with our study were previously identified across multiple biological contexts including recombinant kinases from Study 1[5] (with Kd < 100 nM), K562 lysates from Study 3 ([6,7]), and intact Jurkat cells from Study 2 [6,7]. Kinases from our study were enriched by **4b** 100 μM in KCL22 cells and 10 μM in K562 cells) in UV-dependent protein-directed A*f*BPP analyses.

		Study 1	Study 2	Study 3	Current Study					
Uniprot ID	Gene	Kinase Proteins	Jurkat Cells	K562 Lysates	4b (10 μM) K562 cells	Log2 (FC)	Raw P Value	4b (100 μM) KCL-22 Cells	Log2 (FC)	Raw P Value
P00519	**ABL1**	Yes	Yes	Yes				Yes	5.37423833	5.93E-04
Q06187	**BTK**	Yes		Yes	Yes	3.6694444	7.94E-04	Yes	4.71293767	2.23E-04
P41240	**CSK**	Yes	Yes	Yes				Yes	3.69989332	2.28E-05
O14976	**GAK**	Yes	Yes	Yes	Yes	3.04573631	2.75E-02	Yes	5.38797951	5.28E-03
P07948	**LYN**	Yes		Yes	Yes	2.15881983	3.29E-05	Yes	3.39562988	3.94E-04
Q16539	**MAPK14**	Yes	Yes	Yes	Yes	3.23977534	3.98E-04	Yes	3.30319722	2.99E-06
O43353	**RIPK2**	Yes		Yes				Yes	6.9525617	1.81E-02

Supplementary Table 11. Plasmids and primers

Plasmid #	Plasmid	Mutation	Plasmid Backbone
1	RTN4-FLAG	N/A	pRK5-C-FLAG
2	RTN4-FLAG	C1101S	pRK5-C-FLAG
3	STING	N/A	pcDNA3.1

4	COX5A-FLAG	N/A	pRK5-C-FLAG
5	COX5A-FLAG	T79G	pRK5-C-FLAG
6	COX5A-FLAG	Y80F	pRK5-C-FLAG
7	COX5A-FLAG	D81N	pRK5-C-FLAG
8	COX5A-FLAG	T79W	pRK5-C-FLAG
9	COX5A-FLAG	Y80W	pRK5-C-FLAG
10	COX5A-FLAG	D81W	pRK5-C-FLAG
11	COX5A-HA	N/A	pTwist Lenti SFFV Puro
12	Ubiquitin-HA	N/A	pRK5-HA
13	AP-His	N/A	pAPTag-5
14	AP-Nogo66-His	N/A	pAPTag-5
15	AP-Nogo66-His	C1101W	pAPTag-5
16	GFP	N/A	FUGW
17	Delta 8.9	N/A	Delta 8.9
18	VSVG	N/A	VSVG
19	STARD7	N/A	pRK5-C-FLAG
20	RTNR	N/A	Modified pCLNCX [8]
Primer #	Gene	Mutation	Primer Sequence
1	RTN4	C1101S	Forward: GTAATTCTGCTCTTGGTCATGTGAACTCCACGATAAAGGAACTCAGGCGCCTCTTC<br><br>Reverse: GAAGAGGCGCCTGAGTTCCTTTATCGTGGAGTTCACATGACCAAGAGCAGAATTAC

2	AP-Nogo66 (RTN4)	C1101W	Forward: CTGCTCTTGGTCATGTGAACTGGACGATAAAGG<br><br>Reverse: GGCGCCTGAGTTCCTTTATCGTCCAGTTCACATGAC
3	COX5A	T79G	Forward: GGGATAAACACACTTGTTGGCTATGATATGGTTCC<br><br>Reverse: GGGCTCTGGAACCATATCATAGCCAACAAGTGTG
4	COX5A	Y80F	Forward: GGGATAAACACACTTGTTACCTTTGATATGGTTCCAG<br><br>Reverse: GATTTTGGGCTCTGGAACCATATCAAAGGTAACAAGTG
5	COX5A	D81N	Forward: GTTACCTATAATATGGTTCCAGAGCCCAAAATC<br><br>Reverse: GCTCTGGAACCATATTATAGGTAACAAGTGTGTTTATC
6	COX5A	T79W	Forward: GGGATAAACACACTTGTTTGGTATGATATGGTTCC<br><br>Reverse: GGGCTCTGGAACCATATCATACCAAACAAGTGTG

7	COX5A	Y80W	Forward:<br>GGGATAAACACACTTGTTACCTGGGATATGGTTCC<br><br>Reverse:<br>GGGCTCTGGAACCATATCCCAGGTAACAAGTGTG
8	COX5A	D81W	Forward:<br>CACACTTGTTACCTATTGGATGGTTCCAGAGCCC<br><br>Reverse:<br>CAATGATTTTGGGCTCTGGAACCATCCCATAGGTAAC

(D) Synthetic Procedures

General Synthetic Procedures. All reactions were performed in oven dried glassware under an inert atmosphere of dry N_2 unless stated otherwise. Silica gel P60 (SiliCycle) was used for column chromatography and SiliCycle 60 F254 silica gel (precoated sheets, 0.25 mm thick) was used for analytical thin layer chromatography. Plates were visualized by fluorescence quenching under UV light or by staining reagents. Other reagents were purchased from Sigma-Aldrich (St. Louis, MO), Alfa Aesar (Ward Hill, MA), EMD Millipore (Billerica, MA), Fisher Scientific (Hampton, NH), Oakwood Chemical (West Columbia, SC), Combi-blocks (San Diego, CA), Click Chemistry Tools (Scottsdale, AZ) and Cayman Chemical (Ann Arbor, MI) and used without further purification. ^{1}H NMR and ^{13}C NMR spectra for characterization of new compounds and monitoring reactions were collected in $CDCl_3$, DMSO-d_6, or CD_3OD (Cambridge Isotope Laboratories, Cambridge, MA) on a Bruker AV300, AV400, AV500 and NEO600 MHz spectrometer in the Department of Chemistry & Biochemistry at University of California, Los Angeles. All chemical shifts are reported in the standard notation of parts per million using the peak of residual proton signals of the deuterated solvent as an internal reference. Coupling constant units are in Hertz (Hz). Splitting patterns are indicated as follows: br, broad; s, singlet; d, doublet; t, triplet; q, quartet; m, multiplet; dd, doublet of doublets; dt, doublet of triplets. Low-resolution mass spectrometry was performed on an Agilent Technologies InfinitiyLab LC/MSD single quadrupole LC/MS (ESI source). High-resolution mass spectroscopy was performed on Waters LCT Premier TOF LC/MS with ACQUITY UPLC in the Department of Chemistry & Biochemistry at University of California, Los Angeles or the University of California, Irvine Mass Spectrometry Facility. Data were collected on a Waters LCT Premier by flow injection analysis (FIA) in methanol and data analyzed with MassLynx v. 4.1 software. In ESI+ mode, analyte m/z ions (M+H)+ or (M+Na)+ were validated to less than ± 5 ppm relative to the nearest sodiated polyethylene glycol (CAS: 25322-68-3, av. Mwt 400)) or sodiated

methoxypolyethyleneglycol (CAS: 990-74-4, av. Mwt 350) calibrant peak lockmass. In ESI- mode, analyte m/z ions (M-H)- were validated relative to the nearest calibrant peak from the sodium formate cluster series (m/z 100-2000).

Synthesis of prototype SEE-CITE probes

Minimalist diazirine tag **1** was synthesized according to literature procedure[9].

3-(but-3-yn-1-yl)-3-(2-{[dimethyl(2-phenylethyl)silyl]oxy}ethyl)-3*H*-diazirine (2a)

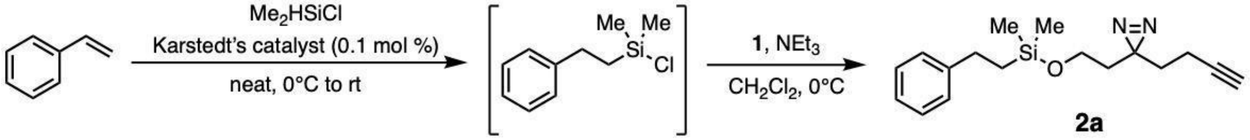

2a

To a stirred mixture of styrene (62.4 μL, 543 μmol) and chlorodimethylsilane (67.0 μL, 615 μmol) was added Platinum(0)-1,3-divinyl-1,1,3,3-tetramethyldisiloxane complex solution (Karstedt's catalyst, 0.05 M, 7.24 μL, 0.362 μmol) at 0 ℃ and the resulting mixture was stirred at room temperature under N_2 atmosphere overnight. The reaction mixture was cooled to 0 °C and then **1** (50.0 mg, 362 μmol) in dry CH_2Cl_2 (1.00 mL) and triethylamine (101 μL, 724 μmol) were added in this order and the resulting mixture was stirred for 30 minutes under N_2 atmosphere. Water was added and the resulting mixture was extracted with CH_2Cl_2 and the combined organic layers were washed with brine, dried over anhydrous Na_2SO_4. After filtration and evaporation of the organic solvents, the crude mixture was purified by silica gel column chromatography (hexane/EtOAc = 98/2) to afford **2a** (45 mg, 150 μmol, 41%) as colorless oil.

Colorless oil; ^{1}H NMR ($CDCl_3$) δ 7.31-7.25 (m, 2H), 7.23-7.14 (m, 3H), 3.45 (t, *J* = 6.4 Hz, 2H), 2.72-2.63 (2H, m), 2.08-1.99 (2H, m), 1.96 (t, *J* = 2.8 Hz, 1H) 1.67 (t, *J* = 8.0 Hz, 2H), 1.61 (t, *J* = 6.4 Hz, 2H), 1.00-0.95 (2H, m), 0.12 (s, 6H); ^{13}C NMR ($CDCl_3$) δ 144.75, 128.32, 127.79, 125.59, 82.89, 68.99, 57.20, 35.91, 32.90, 29.19, 26.80, 18.18, 13.30, -2.27.; HRMS (ESI) Anal. calcd. for $C_{17}H_{24}N_2OSiNa$ *m/z* 323.1555 $[M+Na]^+$, found 323.1570.

3-(but-3-yn-1-yl)-3-(2-{[methyl(phenyl)(2-phenylethyl)silyl]oxy}ethyl)-3*H*-diazirine (2c)

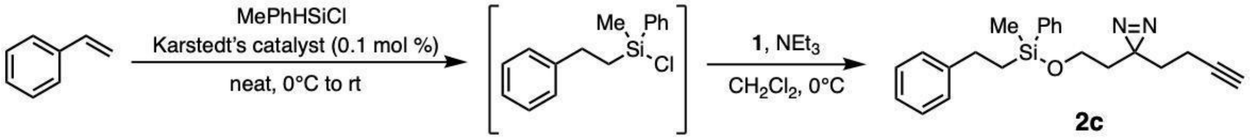

2c

To a stirred mixture of styrene (62.4 μL, 543 μmol) and chloromethylphenylsilane (92.4 μL, 615 μmol) was added Platinum(0)-1,3-divinyl-1,1,3,3-tetramethyldisiloxane complex solution (Karstedt's catalyst, 0.05 M, 7.24 μL, 0.362 μmol) at 0 °C and the resulting mixture was stirred at room temperature under N_2 atmosphere overnight. The reaction mixture was cooled to 0 °C and then **1** (50.0 mg, 362 μmol) in dry CH_2Cl_2 (1.00 mL) and triethylamine (101 μL, 724 μmol) were added in this order and the resulting mixture was stirred for 30 minutes under N_2 atmosphere. Water was added and the resulting mixture was extracted with CH_2Cl_2 and the combined organic layers were washed with brine, dried over anhydrous Na_2SO_4. After filtration and evaporation of the organic solvents, the crude mixture was purified by silica gel column chromatography (hexane/EtOAc = 98/2) to **2c** (115 mg, 317 μmol, 87 %) as colorless oil.

Colorless oil; ^{1}H NMR ($CDCl_3$) δ 7.62-7.56 (m, 2H), 7.43-7.37 (m, 3H), 7.30-7.24 (m, 2H), 7.22-7.14 (m, 3H), 3.51-3.45 (m, 2H), 2.72 (t, J = 8.4 Hz, 2H), 2.04-1.99 (m, 2H), 1.95 (t, J = 2.8 Hz, 1H), 1.66 (t, J = 7.6 Hz, 2H), 1.63-1.58 (m, 2H), 1.31-1.16 (m, 2H), 0.40 (s, 3H); ^{13}C NMR ($CDCl_3$) δ 144.59, 136.30, 133.67, 129.82, 128.31, 127.96, 127.79, 125.62, 82.86, 69.00, 57.71, 35.84, 32.78, 29.04, 26.77, 17.03, 13.29, -4.00; HRMS (ESI) Anal. calcd. for $C_{22}H_{26}N_2OSiNa$ *m/z*385.1712 [M+Na]$^+$, found 385.1694.

Diethyl(2-phenylethyl)silane (3b)

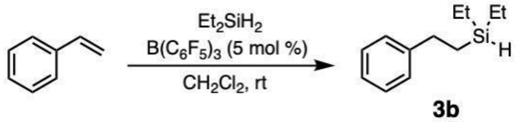

3b

To a stirred solution of tris(perfluorophenyl)borane (123 mg, 240 µmol) in anhydrous CH_2Cl_2 (5.00 mL) was added diethylsilane (933 µL, 7.20 mmol), followed by addition of styrene (550 µL, 4.80 mmol). The reaction mixture was stirred at room temperature for 3 hours. The reaction mixture was diluted with hexane and the resulting mixture was filtered through a short pad of silica gel. After washing with hexane, the filtrate was concentrated under reduced pressure. The residue obtained was purified by silica gel column chromatography (hexane = 100%) to afford **3b** (798 mg, 4.15 mmol, 86 %) as colorless oil.

Colorless oil; ^{1}H NMR ($CDCl_3$) δ 7.32-7.26 (m, 2H), 7.24-7.15 (m, 3H), 3.75-3.69 (m, 1H), 2.73-2.65 (m, 2H), 1.05-0.95 (m, 8H), 0.67-0.59 (m, 4H); ^{13}C NMR ($CDCl_3$) δ 144.97, 128.29, 127.78, 125.57, 30.76, 12.76, 8.15, 2.72; HRMS (ESI) Anal. calcd. for $C_{12}H_{19}Si$ *m/z* 191.1256 [M-H]$^-$, found 191.1248.

(2-phenylethyl)[di(propan-2-yl)]silane (3d)

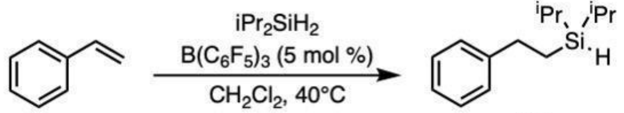

3d

To a stirred solution of Tris(pentafluorophenyl)boron (123 mg, 240 µmol) in anhydrous CH_2Cl_2 (5.00 mL) was added diisopropylsilane (1.18 mL, 7.20 mmol), followed by addition of styrene (550 µL, 4.80 mmol) and then the reaction mixture was stirred at 40 °C for 18 hours. After dilution with hexane, the resulting mixture was filtered through a short pad of silica gel, washing with hexane, the filtrate was concentrated under reduced pressure. The residue obtained was purified by silica gel column chromatography (hexane = 100%) to afford **3d** (632 mg, 2.87 mmol, 60%) as colorless oil.

Colorless oil; ^{1}H NMR ($CDCl_3$) δ 7.32-7.26 (m, 2H), 7.24-7.15 (m, 3H), 3.53-3.49 (m, 1H), 2.75-2.67 (m, 2H), 1.13-0.95 (m, 16H); ^{13}C NMR ($CDCl_3$) [observed peaks] δ; 145.23, 128.32, 127.72, 125.58, 31.47, 19.07, 18.74, 10.76, 10.55; HRMS (ESI) Anal. calcd. for $C_{14}H_{23}Si$ *m/z* 219.1569 [M-H]$^-$, found 219.1559.

3-(but-3-yn-1-yl)-3-(2-{[diethyl(2-phenylethyl)silyl]oxy}ethyl)-3*H*-diazirine (2b)

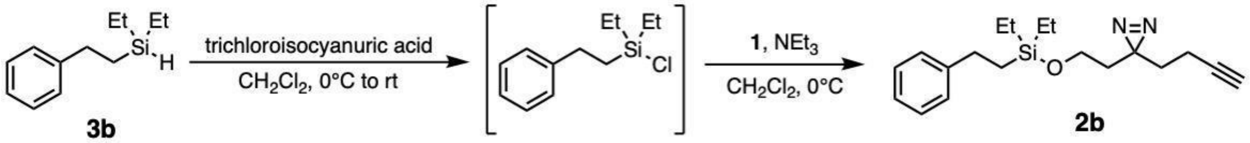

A solution of **3b** (83.5 mg, 434 µmol) in anhydrous CH_2Cl_2 (2.00 mL) was cooled to 0 °C, then trichloroisocyanuric acid (33.6 mg, 145 µmol) was carefully added and then warmed to room temperature and stirred for 1.5 hours under the N_2atmosphere. The insoluble materials were removed by filtration and washed with hexane, then the filtrate was concentrated in vacuo to afford the crude chlorosilane. **1** (40.0 mg, 289 µmol) and this crude chlorosilane were dissolved in anhydrous CH_2Cl_2 (3 mL) and cooled to 0 °C. Triethylamine (121 µL, 868 µmol) was added and the resulting mixture was stirred for 30 minutes under N_2 atmosphere. Water was added and the resulting biphasic mixture was extracted with CH_2Cl_2 and the combined organic layers were washed with brine and then dried over anhydrous Na_2SO_4. After filtration and evaporation, the residue was purified by silica gel column chromatography (hexane/EtOAc = 98/2) to afford **2b** (60 mg, 180 µmol, 62 %) as colorless oil.

Colorless oil; ^{1}H NMR ($CDCl_3$) δ 7.31-7.26 (m, 2H), 7.24-7.14 (m, 3H), 3.49 (t, *J* = 6.4 Hz, 2H), 2.72-2.65 (m, 2H), 2.07-2.00 (m, 2H), 1.96 (t, *J* = 2.8 Hz, 1H), 1.68 (t, *J* = 8.0 Hz, 2H), 1.60 (t, *J* = 6.4 Hz, 2H), 1.02-0.95 (m, 8H), 0.64 (q, J = 8.0 Hz, 4H); ^{13}C NMR ($CDCl_3$) δ 145.01, 128.33, 127.74, 125.59, 82.89, 68.97, 57.50, 36.07, 32.91, 29.19, 26.84, 14.83, 13.31, 6.73, 4.70; HRMS (ESI) Anal. calcd. for $C_{19}H_{28}N_2OSiNa$ *m/z* 351.1869 $[M+Na]^+$, found 351.1883.

3-(but-3-yn-1-yl)-3-[2-({(2-phenylethyl)[di(propan-2-yl)]silyl}oxy)ethyl]-3*H*-diazirine (2d)

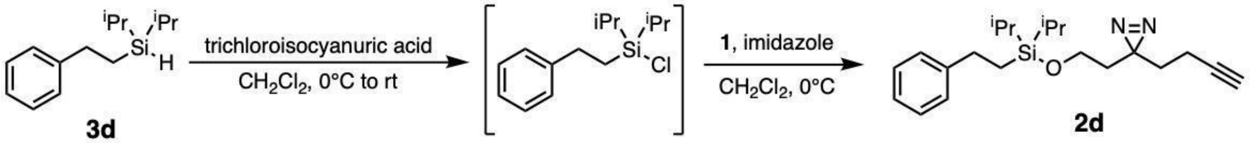

A solution of **3d** (128 mg, 579 µmol) in anhydrous CH_2Cl_2 (2.00 mL) was cooled to 0 °C, then trichloroisocyanuric acid (45.1 mg, 194 µmol) was carefully added and then warmed to room temperature and stirred for 1.5 hours under N_2atmosphere. The insoluble materials were removed by filtration and washed with hexane, then the filtrate was concentrated in vacuo to afford the crude chlorosilane. **1** (40.0 mg, 289 µmol) and imidazole (78.8 mg, 1.16 mmol) were dissolved in anhydrous CH_2Cl_2 (1.50 mL) and cooled to 0 °C, then the crude chlorosilane in anhydrous CH_2Cl_2 (1.50 mL) was added and the resulting mixture was stirred for 30 minutes under N_2 atmosphere. Water was added and the resulting biphasic mixture was extracted with CH_2Cl_2 and the combined organic layers were washed with brine and then dried over anhydrous Na_2SO_4. After filtration and evaporation, the residue was purified by silica gel column chromatography (hexane/EtOAc = 100/0 to 98/2) to afford **2d** (58 mg, 160 µmol, 57 %) as colorless oil.

Colorless oil; 1H NMR (CDCl3) δ 7.32-7.26 (m, 2H), 7.24-7.15 (m, 3H), 3.57 (t, *J* = 6.4 Hz, 2H), 2.74-2.68 (m, 2H), 2.04 (td, *J* = 7.6, 2.8 Hz, 2H), 1.96 (t, *J* = 2.8 Hz, 1H), 1.70 (t, *J* = 7.6 Hz, 2H), 1.61 (t, *J* = 6.4 Hz, 2H), 1.10-0.98 (m, 16H); ^{13}C NMR ($CDCl_3$) δ 145.32, 128.38, 127.65, 125.60, 82.89, 68.97, 58.11, 36.21, 32.88, 29.53, 26.90, 17.64, 13.32, 12.91, 12.43; HRMS (ESI) Anal. calcd. for $C_{21}H_{32}N_2OSiNa$ *m/z* 379.2182 $[M+Na]^+$, found 379.2169.

1-benzyl 4-(3-((2-(3-(but-3-yn-1-yl)-3H-diazirin-3-yl)ethoxy)dimethylsilyl)propyl) piperazine-1,4-dicarboxylate (3)

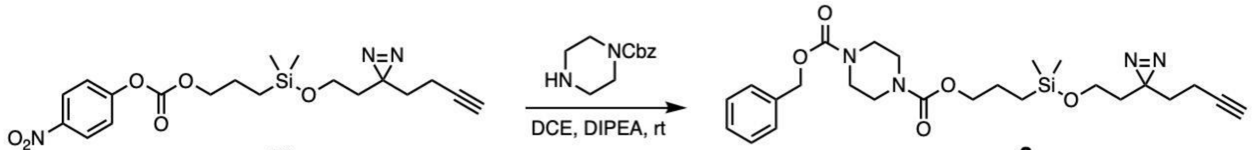

To a stirred mixture of **10** (46 mg, 110 µmol) and benzyl piperazine-1-carboxylate (36mg, 32 µL, 160 µmol) in anhydrous 1,2-Dichloroethane (1.10 mL) was added DIPEA (21 mg, 29 µL, 160 µmol). The resulting mixture was stirred at room temperature for 5 hours. The reaction mixture was then concentrated under reduced pressure and directly subjected to purification by preparatory-HPLC to afford **3** (20 mg, 40 µmol, 37%) as a brown oil.

Brown oil; ^{1}H NMR (300 MHz, $CDCl_3$) δ 7.38 – 7.32 (m, 5H), 5.15 (s, 2H), 4.06 (t, *J* = 7.0 Hz, 2H), 3.52 – 3.44 (m, 8H), 3.42 (t, *J* = 6.3 Hz, 2H), 2.06 – 1.98 (m, 2H), 1.96 (t, *J* = 2.7 Hz, 1H), 1.69 – 1.58 (m, 6H), 0.63 – 0.54 (m, 2H), 0.12 (s, 6H); ^{13}C NMR (126 MHz, $CDCl_3$) δ 155.57, 155.33, 136.56, 128.68, 128.30, 128.14, 82.98, 69.16, 68.15, 67.52, 57.32, 43.66 (broad), 35.96, 33.00, 26.90, 22.97, 13.41, 12.19, -2.21; HRMS (ESI) Anal. calcd. for $C_{25}H_{36}N_4O_5SiNa$ *m/z* 523.2347 $[M+Na]^+$, found 523.2355.

2-{[6-(4-{2-[3-(but-3-yn-1-yl)-3*H*-diazirin-3-yl]ethyl}piperazin-1-yl)-2-methylpyrimidin-4-yl]amino}-*N*-(2-chloro-6-methylphenyl)-1,3-thiazole-5-carboxamide (4a)

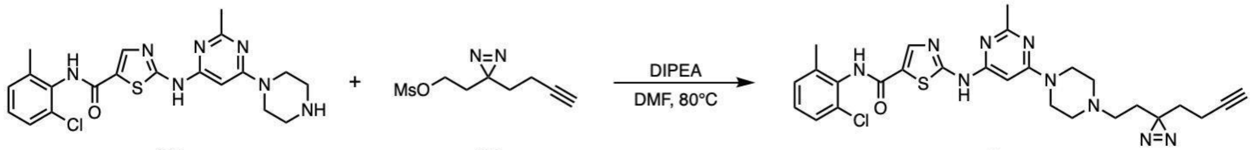

S1 (100.0 mg, 225.3 µmol)[10], **S2** (58.5 mg, 270.3 µmol)[11] and DIPEA (78.5 µL, 450.5 µmol) in anhydrous DMF (1.00 mL) were stirred at 80 °C under N_2 for 22 hours. After cooling to room temperature, water was added and the precipitate was filtered and washed with CH_2Cl_2 and dried in vacuo to afford **4a** (44 mg, 79 µmol, 35%) as colorless solid.

Colorless solid; ^{1}H NMR (DMSO-d$_6$) δ 11.37 (brs, 1H), 9.86 (s, 1H), 8.21 (s, 1H), 7.39 (dd, *J* = 7.6, 2.0 Hz, 1H), 7.30-7.21 (m, 2H), 6.04 (s, 1H), 3.54-3.45 (m, 4H), 2.83 (t, *J* = 2.8 Hz, 1H), 2.43-2.34 (m, 7H), 2.23 (s, 3H), 2.15 (t, *J* = 7.2 Hz, 2H), 2.02 (td, *J* = 7.6, 2.8 Hz, 2H), 1.62-1.54 (m, 4H); ^{13}C NMR (DMSO-d$_6$) δ 165.18, 162.55, 162.34, 159.91, 156.95, 140.83, 138.82, 133.52, 132.43, 129.03, 128.18, 127.01, 125.71, 83.29, 82.63, 71.75, 52.05, 43.56, 31.72, 29.41, 27.65, 25.57, 18.30, 12.69; HRMS (ESI) Anal. calcd. for $C_{27}H_{31}ClN_9OS$ *m/z* 564.2055 $[M+H]^+$, found 564.2040.

2-[3-(but-3-yn-1-yl)-3*H*-diazirin-3-yl]ethyl 4-[6-({5-[(2-chloro-6-methylphenyl)carbamoyl]-1,3-thiazol-2-yl}amino)-2-methylpyrimidin-4-yl]piperazine-1-carboxylate (4b)

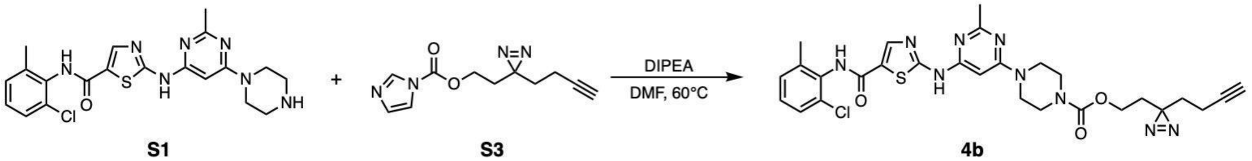

S1 **S3** **4b**

To a stirred solution of **S3** (80 mg, 0.34 mmol)[12] in anhydrous DMF (2 mL) was added **S1** (100 mg, 225 μmol)[10] and DIPEA (118 μL, 676 μmol) and then the resulting mixture was stirred at 60 ^{o}C under N_2 atmosphere for 19 hours. The reaction mixture was diluted with EtOAc and washed with water three times, brine, and then dried over anhydrous Na_2SO_4. After filtration and evaporation, the residue was purified by silica gel column chromatography (CH_2Cl_2/MeOH = 95/5), then the second purification (EtOAc/MeOH = 98/2) was conducted to afford **4b**(38.4 mg, 63.1 μmol, 28%) as colorless solid.

Colorless solid; ^{1}H NMR (DMSO-d$_6$) δ 11.50 (brs, 1H), 9.87 (s, 1H), 8.21 (s, 1H), 7.39 (dd, *J* = 8.0, 2.0 Hz, 1H), 7.30-7.21 (m, 2H), 6.07 (s, 1H), 3.92 (t, 6.0 Hz, 2H), 3.63-3.39 (m, 8H), 2.84 (t, *J* = 2.8 Hz, 1H), 2.41 (s, 3H), 2.23 (s, 3H), 2.01 (td, *J* = 7.2, 2.8 Hz, 2H), 1.74 (t, *J* = 6.0 Hz, 2H), 1.62 (t, *J* = 7.2 Hz, 2H); ^{13}C NMR (DMSO-d$_6$) δ 165.22, 162.51, 162.28, 159.90, 157.00, 154.25, 140.82, 138.81, 133.50, 132.43, 129.02, 128.17, 127.00, 125.77, 83.13, 82.84, 71.81, 60.01, 43.17, 31.69, 31.44, 26.87, 25.57, 18.30, 12.61; HRMS (ESI) Anal. calcd. for $C_{28}H_{31}ClN_9O_3S$ *m/z* 608.1954 $[M+H]^+$, found 608.1984.

3-(2-{[(3-bromopropyl)(dimethyl)silyl]oxy}ethyl)-3-(but-3-yn-1-yl)-3*H*-diazirine (7)

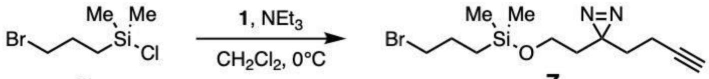

6 **7**

A mixture of **1** (100 mg, 724 μmol) and triethylamine (303 μL, 2.17 mmol) in anhydrous CH_2Cl_2 (5 mL) was cooled to 0 °C then **6** (234 mg, 1.09 mmol) in anhydrous CH_2Cl (2 mL) was added and the resulting mixture was stirred at the same temperature for 10 min. Water was added and the resulting mixture was extracted with CH_2Cl_2 and the combined organic layers were washed with brine and then dried over anhydrous Na_2SO_4. After filtration and evaporation, the residue was purified by (hexane/EtOAc = 98/2) to afford **7** (172.4 mg, 543.3 μmol, 75 %) as pale yellow oil.

Pale yellow oil; ^{1}H NMR ($CDCl_3$) δ 3.46-3.39 (m, 4H), 2.06-2.00 (m, 2H), 1.98 (t, *J* = 2.8 Hz, 1H), 1.95-1.85 (m, 2H), 1.66 (t, *J* = 7.6 Hz, 2H), 1.62 (t, *J* = 6.4 Hz, 2H), 0.75-0.68 (m, 2H), 0.12 (s, 6H); ^{13}C NMR ($CDCl_3$) δ 82.84, 69.00, 57.21, 36.88, 35.83, 32.86, 27.09, 26.74, 15.41, 13.27, -2.32; HRMS (ESI) expected m/z was not observed.

2-{[6-(4-{3-[{2-[3-(but-3-yn-1-yl)-3*H*-diazirin-3-yl]ethoxy}(dimethyl)silyl]propyl}piperazin-1-yl)-2-methylpyrimidin-4-yl]amino}-*N*-(2-chloro-6-methylphenyl)-1,3-thiazole-5-carboxamide (4c)

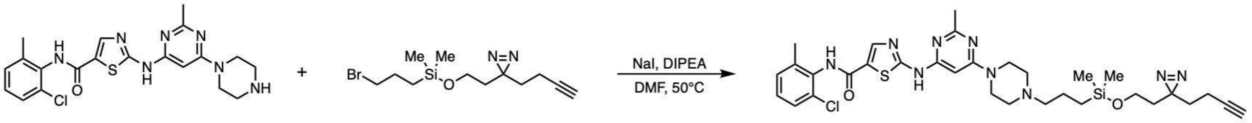

S1 **7** **4c**

To a stirred mixture of **S1** (80.0 mg, 180 μmol)[10] and **7** (85.8 mg, 270 μmol) in anhydrous DMF (1 mL) was added DIPEA (94.2 μL, 541 μmol) and sodium iodide (40.5 mg, 270 μmol) and then the resulting solution was stirred at 50 °C under N_2 atmosphere for 2.5 hours. The reaction temperature was cooled to room temperature and then the mixture was diluted with EtOAc and washed with water three times, brine and dried over anhydrous Na_2SO_4. After filtration and

evaporation, the residue was purified by silica gel column chromatography (CH_2Cl_2/MeOH = 95/5) to afford **4c** (57.5 mg, 84.5 μmol, 47%) as colorless solid.

Colorless solid; ^1H NMR (DMSO-d_6) δ 11.45 (s, 1H), 9.86 (s, 1H), 8.20 (s, 1H), 7.39 (dd, *J* = 7.6, 2.0 Hz, 1H), 7.31-7.20 (m, 2H), 6.04 (s, 1H), 3.56-3.44 (m, 4H), 3.40 (t, *J* = 6.0 Hz, 2H), 2.81 (t, *J* = 2.8 Hz, 1H), 2.45-2.34 (m, 4H), 2.39 (s, 3H), 2.33-2.25 (m, 2H), 2.23 (s, 3H), 2.00 (td, *J* = 7.6, 2.8 Hz, 2H), 1.62-1.41 (m, 6H), 0.60-0.49 (m, 2H), 0.07 (s, 6H); ^{13}C NMR (DMSO-d_6) [observed peaks] δ 165.15, 162.56, 162.36, 159.91, 156.93, 140.81, 138.81, 133.52, 132.43, 129.01, 128.15, 126.99, 125.69, 83.21, 82.60, 71.65, 60.98, 56.81, 52.30, 43.60, 35.06, 32.07, 27.10, 25.57, 20.02, 18.30, 13.27, 12.66, -2.19; HRMS (ESI) Anal. calcd. for $C_{32}H_{43}ClN_9O_2SSi$ *m/z* 680.2713 $[M+H]^+$, found 680.2745.

(3*R*)-1-[5-({4-[chloro(difluoro)methoxy]phenyl}carbamoyl)-3-(1*H*-pyrazol-5-yl)pyridin-2-yl]pyrrolidin-3-yl {2-[3-(but-3-yn-1-yl)-3*H*-diazirin-3-yl]ethyl}carbamate (5a)

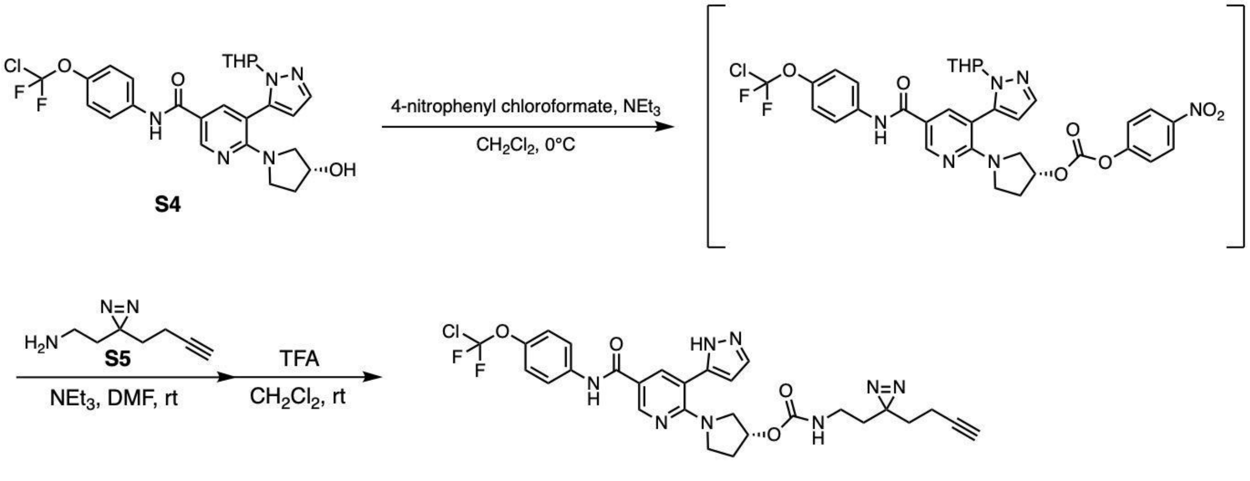

To a stirred solution of **S4** (70.0 mg, 131 μmol)[13] in anhydrous CH_2Cl_2 (2 mL) was added triethylamine (54.8 μL, 393 μmol) and 4-nitrophenyl chloroformate (39.6 mg, 197 μmol) at 0 °C and the resulting mixture was stirred at room temperature under N_2 atmosphere for 68 hours. Additional 4-nitrophenyl chloroformate (39.6 mg, 197 μmol) and triethylamine (54.8 μL, 3.00 Eq, 393 μmol) were added at 0 °C and the resulting mixture was stirred at room temperature for 27 hours. The reaction mixture was diluted with CH_2Cl_2 and washed with brine, then dried over anhydrous Na_2SO_4. After filtration and evaporation, the residue was purified by silica gel column chromatography (hexane/EtOAc = 1/1, Rf = 0.35) to afford the carbonate intermediate (60.9 mg, 87.1 μmol, 67 %) as a colorless solid. This intermediate was dissolved in anhydrous DMF (1 mL) and then **S5** (14.1 mg, 103 μmol)[9] and triethylamine (35.9 μL, 257 μmol) were added. After stirring at room temperature under N_2 atmosphere for 3.5 hours, the reaction mixture was diluted with EtOAc and washed with saturated $NaHCO_3$ aq., water, 1N HCl aq., water, saturated $NaHCO_3$ aq. and brine successively, then dried over anhydrous Na_2SO_4. After filtration and evaporation, the crude THP-protected carbamate was obtained. To a stirred solution of the crude carbamate in CH_2Cl_2 (1 mL) was added TFA (1 mL) and the resulting mixture was stirred at room temperature for 3 hours. The reaction mixture was neutralized by pouring into saturated $NaHCO_3$ aq. and extracted CH_2Cl_2. The combined organic layers were washed with saturated $NaHCO_3$ aq., brine, dried over anhydrous Na_2SO_4. After filtration and evaporation, the residue was purified by silica gel column chromatography (CH_2Cl_2/MeOH = 92/8) to afford **5a** (38.9 mg, 63.5 μmol, 74% in 2 steps) as colorless solid.

Colorless solid; ^{1}H NMR (CD_3OD) δ 8.82-8.68 (m, 1H), 8.13-8.04 (m, 1H), 7.84-7.65 (m, 3H), 7.32-7.21 (m, 2H), 6.52-6.42 (m, 1H), 5.21-5.08 (m, 1H), 3.63-3.17 (m, 4H), 2.97 (t, *J* = 4.8 Hz, 2H), 2.28-2.21 (m, 1H), 2.14-1.90 (m, 4H), 1.65-1.44 (m, 4H); ^{13}C NMR (DMSO-d_6, observed peaks) δ 164.05, 156.94, 155.45, 147.55, 144.78, 138.95, 138.62, 128.99, 125.00 (t, *J* = 285.3 Hz), 121.77, 121.40, 117.89, 113.72, 105.98, 83.11, 72.89, 71.74, 54.88, 46.83, 35.26, 32.16, 31.23, 30.50, 27.06, 12.64; HRMS (ESI) Anal. calcd. for $C_{28}H_{28}ClF_2N_8O_4$ *m/z* 613.1885 $[M+H]^+$, found 613.1883.

3-[chloro(dimethyl)silyl]propyl 4-nitrophenyl carbonate (9)

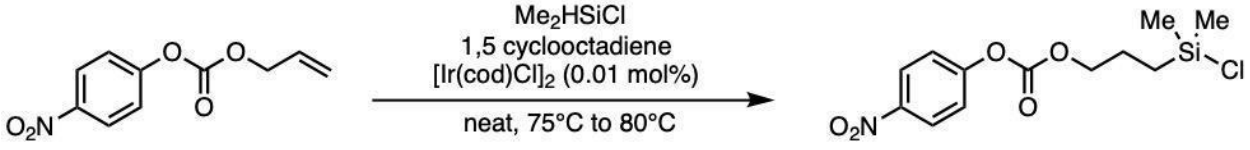

A mixture of **8** (1.50 g, 6.72 mmol), $[Ir(cod)Cl]_2$ (451 µg, 0.672 µmol) and 1,5-cyclooctadiene (16.5 µL, 134 µmol) was stirred at 75 °C until the SM melted down. After that, chlorodimethylsilane (1.1 mL, 10.1 mmol) was slowly added to the mixture for a period of 15 min at 75 ℃. After the completion of the addition, the mixture was heated at 80 °C for 1 h. After cooling to room temperature, the mixture was concentrated under reduced pressure to give crude **9** (2.05 g, 6.46 mmol, 96 %) as light brown oil, which was analytically pure enough and used in the next step without purification.

Light brown oil; ^{1}H NMR ($CDCl_3$) δ 8.28-8.22 (m, 2H), 7.39-7.34 (m, 2H), 4.28 (t, *J* = 6.8 Hz), 1.93-1.82 (m, 2H), 0.95-0.86 (m, 2H), 0.44 (s, 6H); ^{13}C NMR ($CDCl_3$) δ 155.48, 152.41, 145.29, 125.20, 121.71, 71.02, 22.30, 14.69, 1.44.

3-[{2-[3-(but-3-yn-1-yl)-3*H*-diazirin-3-yl]ethoxy}(dimethyl)silyl]propyl 4-nitrophenyl carbonate (10)

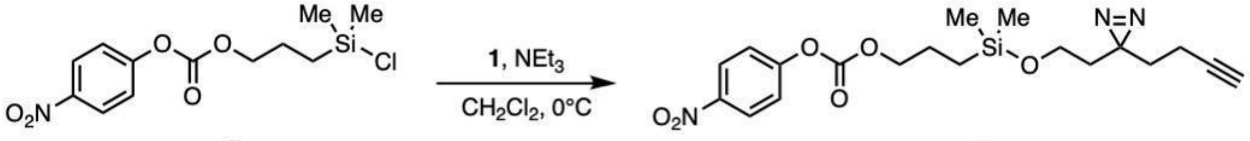

To a stirred mixture of **1** (100 mg, 724 µmol) and triethylamine (303 µL, 2.17 mmol) in anhydrous CH_2Cl_2 (5 mL) at 0 ℃ was added **9** (345 mg, 1.09 mmol) in anhydrous CH_2Cl_2 (2 mL) dropwise and the resulting mixture was stirred at the same temperature for 10 minutes. Water was added and the resulting mixture was extracted with CH_2Cl_2 and the combined organic layers were washed with brine and then dried over anhydrous Na_2SO_4. After filtration and evaporation under reduced pressure, the residue was purified by silica gel column chromatography (hexane/EtOAc = 9/1) to afford **10**(245.8 mg, 586 µmol 81 %) as pale yellow oil.

Pale yellow oil; ^{1}H NMR ($CDCl_3$) δ 8.31-8.24 (m, 2H), 7.42-7.35 (m, 2H), 4.27 (t, *J* = 9.2 Hz, 2H), 3.45 (t, *J* = 8.4 Hz, 2H), 2.06-1.99 (m, 2H), 1.97 (t, *J* = 3.6 Hz, 1H), 1.88-1.74 (m, 2H), 1.70-1.58

(m, 4H), 0.71-0.61 (m, 2H), 0.14 (s, 6H); ^{13}C NMR ($CDCl_3$) δ 155.58, 152.51, 145.33, 125.27, 121.78, 82.83, 71.66, 69.02, 57.21, 35.77, 32.85, 26.76, 22.43, 13.26, 11.92, -2.38; HRMS (ESI) Anal. calcd. for $C_{19}H_{25}N_3O_6SiNa$ *m/z* 442.1410 $[M+Na]^+$, found 442.1398.

tert-butyl {(3*R*)-1-[3-bromo-5-({4-[chloro(difluoro)methoxy]phenyl}carbamoyl)pyridin-2-yl]pyrrolidin-3-yl}carbamate (S7)

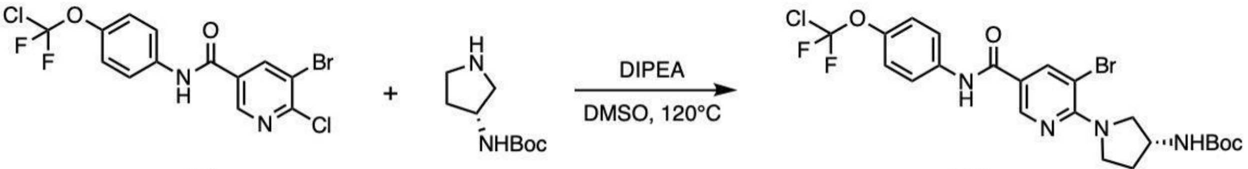

S6 **S7**

A mixture of **S6**[13] (2.59 g, 6.29 mmol), (*R*)-(+)-3-[*N*-(*tert*-butyloxycarbonyl)amino]pyrrolidine (1.40 g, 7.54 mmol), DIPEA (3.28 mL, 18.86 mmol) in anhydrous DMSO (12 mL) was stirred at 120 °C for 1 hour. After cooling to room temperature, the reaction mixture was diluted with EtOAc and washed with water, 1N HCl aq., sat. $NaHCO_3$ aq., water and brine successively, then dried over anhydrous Na_2SO_4. After filtration and concentration under reduced pressure, **S7** (3.46 g, 6.15 mmol, 98 %) was obtained as brown solid.

Brown solid; ^1H NMR (DMSO-d_6) δ 10.23 (s, 1H), 8.68 (d, *J* = 2.0 Hz, 1H), 8.34 (d, *J* = 2.0 Hz, 1H), 7.88-7.82 (m, 2H), 7.36-7.30 (m, 2H), 7.21 (d, *J* = 6.8 Hz, 1H), 4.09-3.98 (m, 1H), 3.92-3.78 (m, 2H), 3.76-3.67 (m, 1H), 3.58 (dd, *J* = 10.8, 4.8 Hz, 1H), 2.11-1.98 (m, 1H), 1.91-1.78 (m, 1H), 1.38 (s, 9H); ^{13}C NMR (DMSO-d_6) δ 162.81, 156.01, 155.26, 146.55, 144.92, 141.57, 138.36, 124.98 (t, *J* = 285 Hz), 121.79, 121.42, 120.29, 101.53, 77.89, 55.36, 49.89, 48.23, 30.43, 28.21; HRMS (ESI) Anal. calcd. for $C_{22}H_{25}BrClF_2N_4O_4$ *m/z* 563.0690 $[M+H]^+$, found 563.0692.

tert-butyl [(3*R*)-1-{5-({4-[chloro(difluoro)methoxy]phenyl}carbamoyl)-3-[1-(tetrahydro-2*H*-pyran-2-yl)-1*H*-pyrazol-5-yl]pyridin-2-yl}pyrrolidin-3-yl]carbamate (S8)

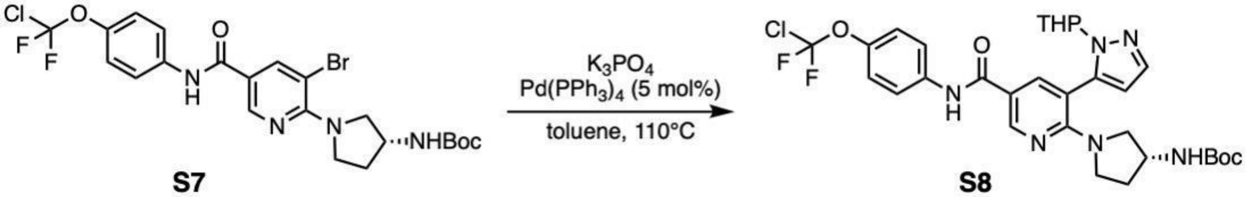

S7 **S8**

A mixture of **S7** (600 mg, 1.07 mmol), 1-(tetrahydro-2*H*-pyran-2-yl)-5-(4,4,5,5-tetramethyl-1,3,2-dioxaborolan-2-yl)-1*H*-pyrazole (386 mg, 1.39 mmol), K_3PO_4 (680 mg, 3.20 mmol) in toluene (5 mL) was degassed by sonication for a while. After that, $Pd(PPh_3)_4$ (61.7 mg, 53.4 μmol) was added and the resulting mixture was stirred at 110 °C under N_2 atmosphere for 16 hours. After cooling to room temperature, the reaction mixture was diluted with EtOAc and washed with water, brine, and dried over anhydrous Na_2SO_4. After filtration and evaporation, the residue was purified by silica gel column chromatography (CH_2Cl_2/EtOAc = 3/1) twice to afford **S8** (345.1 mg, 545.1 μmol, 51 %) as pale yellow amorphous.

Pale yellow solid; ^{1}H NMR (CD_3OD) δ 8.81 (d, J = 2.4 Hz, 1H), 8.11-7.90 (m, 1H), 7.81-7.72 (m, 2H), 7.65 (d, J = 2.4 Hz, 1H), 7.30-7.21 (m, 2H), 6.47-6.41 (m, 1H), 5.14-4.97 (m, 1H), 4.12-3.87 (m, 2H), 3.58-3.03 (m, 5H), 2.45-2.22 (m, 1H), 2.10-1.93 (m, 2H), 1.91-1.71 (m, 2H), 1.70-1.55 (m, 2H), 1.55-1.48 (m, 1H), 1.47-1.35 (m, 9H); ^{13}C NMR (CD_3OD, obscure spectrum, observed peaks) 166.57, 158.88, 157.93, 150.32, 147.58, 142.59, 142.53, 141.58, 140.66, 139.15, 126.77 (t, J = 285 Hz), 123.28, 123.01, 119.20, 109.89, 86.17, 86.08, 80.29, 69.22, 55.49, 51.32, 48.04, 31.70, 28.72, 26.01, 23.88, 23.82; HRMS (ESI) Anal. calcd. for $C_{30}H_{36}ClF_2N_6O_5$ m/z 633.2398 [M+H]$^+$, found 633.2416.

6-[(3*R*)-3-aminopyrrolidin-1-yl]-*N*-{4-[chloro(difluoro)methoxy]phenyl}-5-(1*H*-pyrazol-5-yl)pyridine-3-carboxamide hydrochloride (1:1) (S9)

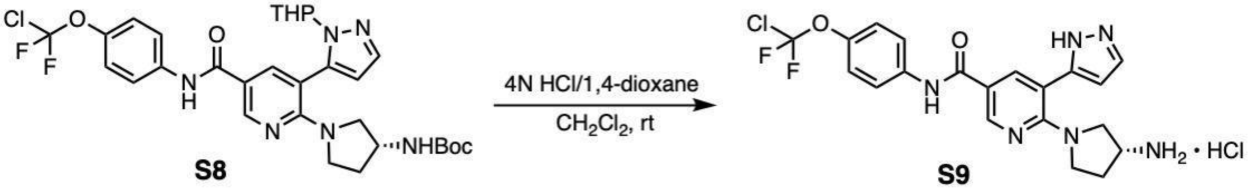

To a solution of **S8** (561 mg, 886 μmol) in CH_2Cl_2 (5 mL) was added 4N hydrochloric acid/1,4-dioxane (5 mL) and the resulting mixture was stirred at room temperature for 1.5 hours. After concentration under reduced pressure, the residue was triturated with ether and the solid was collected by filtration and washed with ether then dried in vacuo to afford **S9**(428 mg, 883 μmol, quant.) as a colorless solid.

Colorless solid; ^{1}H NMR (CD_3OD) δ 8.72 (d, J = 2.4 Hz, 1H), 8.55 (d, J = 2.4 Hz, 1H), 7.92 (d, J = 2.4 Hz, 1H), 7.85-7.80 (m, 2H), 7.33-7.27 (m, 2H), 6.73 (d, J = 2.4 Hz, 1H), 4.08-3.96 (m, 1H), 3.86-3.60 (m, 3H), 3.52-3.38 (m, 2H), 3.46 (dd, J = 11.6, 4.0 Hz, 1H), 2.56-2.39 (m, 1H), 2.32-2.17 (m, 1H); ^{13}C NMR (CD_3OD) δ 162.92, 151.99, 147.94, 146.90, 145.85, 139.38, 138.56, 132.21, 126.72 (t, J = 285 Hz), 123.37, 123.11, 121.46, 120.92, 108.73, 55.24, 51.25, 49.86, 29.78; HRMS (ESI) Anal. calcd. for $C_{20}H_{20}ClF_2N_6O_2$ m/z 449.1299 [M+H]$^+$, found 449.1308.

2-[3-(but-3-yn-1-yl)-3*H*-diazirin-3-yl]ethyl {(3*R*)-1-[5-({4-[chloro(difluoro)methoxy]phenyl}carbamoyl)-3-(1*H*-pyrazol-5-yl)pyridin-2-yl]pyrrolidin-3-yl}carbamate (5b)

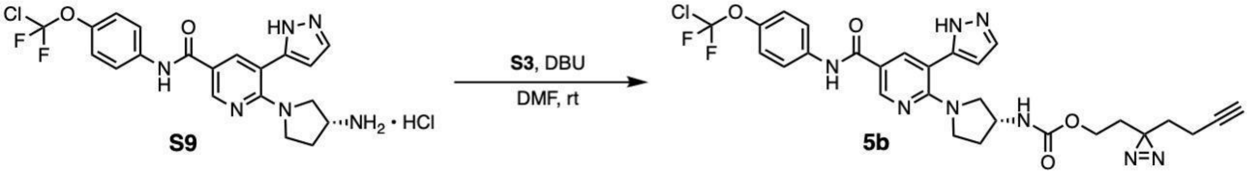

A mixture of **S9** (120 mg, 247 μmol) and **S3** (68.9 mg, 297 μmol) in anhydrous DMF (2 mL) was added DBU (112 μL, 742 μmol) and the resulting mixture was stirred at room temperature for 28 hours. The reaction mixture was diluted with EtOAc and washed with water, 1N HCl aq., sat. $NaHCO_3$ aq., brine, then dried over anhydrous Na_2SO_4. After filtration and evaporation, the residue was purified by silica gel column chromatography (CH_2Cl_2/MeOH = 95/5) to afford **5b** (32.9 mg, 53.7 μmol, 49 %) as a colorless solid.

Colorless solid; ^{1}H NMR (CD_3OD) δ 8.70 (d, J = 2.4 Hz, 1H), 8.25-8.18 (m, 1H), 7.82-7.71 (m, 3H), 7.31-7.23 (m, 2H), 6.58-6.48 (m, 1H), 4.24-4.09 (m, 1H), 3.93 (t, J = 6.0 Hz, 2H), 3.66-3.16 (m, 4H), 2.26 (t, J = 2.8 Hz, 1H), 2.18-2.07 (m, 1H), 2.02 (td, J = 7.6, 2.8 Hz, 2H), 1.98-1.89 (m, 1H), 1.71 (t, J = 6.0 Hz, 2H), 1.63 (t, J = 7.6 Hz, 2H); ^{13}C NMR (DMSO-d_6) [observed peaks] δ 164.01, 156.94, 155.56, 147.55, 144.79, 138.99, 138.64, 127.30, 125.02 (t, J = 285 Hz), 122.74,

121.78, 121.41, 117.80, 106.12, 83.15, 71.80, 58.69, 54.19, 50.07, 47.17, 31.82, 31.51, 30.26, 26.78, 12.65; HRMS (ESI) Anal. calcd. for $C_{28}H_{28}ClF_2N_8O_4$ *m/z* 613.1885 [M+H]$^+$, found 613.1886.

3-[{2-[3-(but-3-yn-1-yl)-3*H*-diazirin-3-yl]ethoxy}(dimethyl)silyl]propyl {(3*R*)-1-[5-({4-[chloro(difluoro)methoxy]phenyl}carbamoyl)-3-(1H-pyrazol-5-yl)pyridin-2-yl]pyrrolidin-3-yl}carbamate (5c)

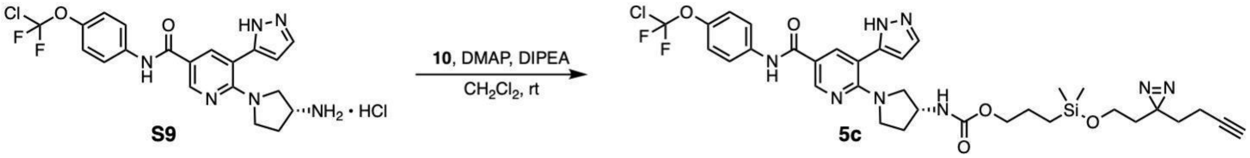

To a stirred mixture of **S9** (150 mg, 309 μmol), DMAP (37.8 mg, 309 μmol) and DIPEA (108 μL, 618 μmol) in anhydrous CH_2Cl_2 (2 mL) was added **10** (169 mg, 402 μmol) in anhydrous CH_2Cl_2 (2 mL) then the resulting mixture was stirred at room temperature for 1 hour. The reaction mixture was diluted with CH_2Cl_2 and washed with water, brine and dried over anhydrous Na_2SO_4. After filtration and evaporation, the residue was purified by silica gel column chromatography (CH_2Cl_2/MeOH = 96/4) to afford **5c** (68.9 mg, 94.5 μmol, 31 %) as a colorless solid.

Colorless solid; ^{1}H NMR (CD_3OD) δ 8.81-8.68 (m, 1H), 8.12-8.04 (m, 1H), 7.89-7.68 (m, 3H), 7.26 (d, *J* = 8.8 Hz, 2H), 6.54-6.43 (m, 1H), 4.17-4.06 (m, 1H), 4.06-3.91 (m, 2H), 3.59-3.05 (m, 6H), 2.24 (t, *J*= 2.8 Hz, 1H), 2.13-1.96 (m, 3H), 1.93-1.78 (m, 1H), 1.74-1.50 (m, 6H), 0.69-0.53 (m, 2H), 0.14 (s, 6H); ^{13}C NMR (DMSO-d_6) δ 164.14, 157.13, 155.94, 149.00, 147.41, 144.75, 138.78, 138.67, 128.86, 125.00 (t, *J* = 285 Hz), 121.76, 121.39, 117.76, 113.72, 105.94, 83.21, 71.66, 66.05, 56.84, 54.35, 49.98, 47.20, 34.99, 31.99, 30.28, 27.07, 22.54, 12.63, 11.61, -2.31; HRMS (ESI) Anal. calcd. for $C_{33}H_{40}ClF_2N_8O_5Si$ *m/z* 729.2542 [M+H]$^+$, found 729.2548.

(E) Rigid Receptor Docking Methods

The initial asciminib structure used to predict binding poses was generated and optimized in Gaussview using the Universal Force Field. The optimized structure was then converted to a pdbqt file format and only polar hydrogens were included using AutoDockTools (version 1.5.7). The receptor (PDB: 2DYR) was abridged to its monomeric form, and mutated and locally optimized *in silico* (Chain E G79T) in ChimeraX 1.8 using the Dunbrack rotamer library. The box size was set to 37Å x 37Å x 37Å (spacing of .308 Å) centered around the two preferentially labeled residues by compound **5c** (**Fig. 5l**). Binding energies were calculated using the Lamarckian Genetic Algorithm 6.0.16 and empirical free-energy scoring function in Autodock 4.2, with the highest scoring poses chosen for analysis.

(F) NMR Spectra

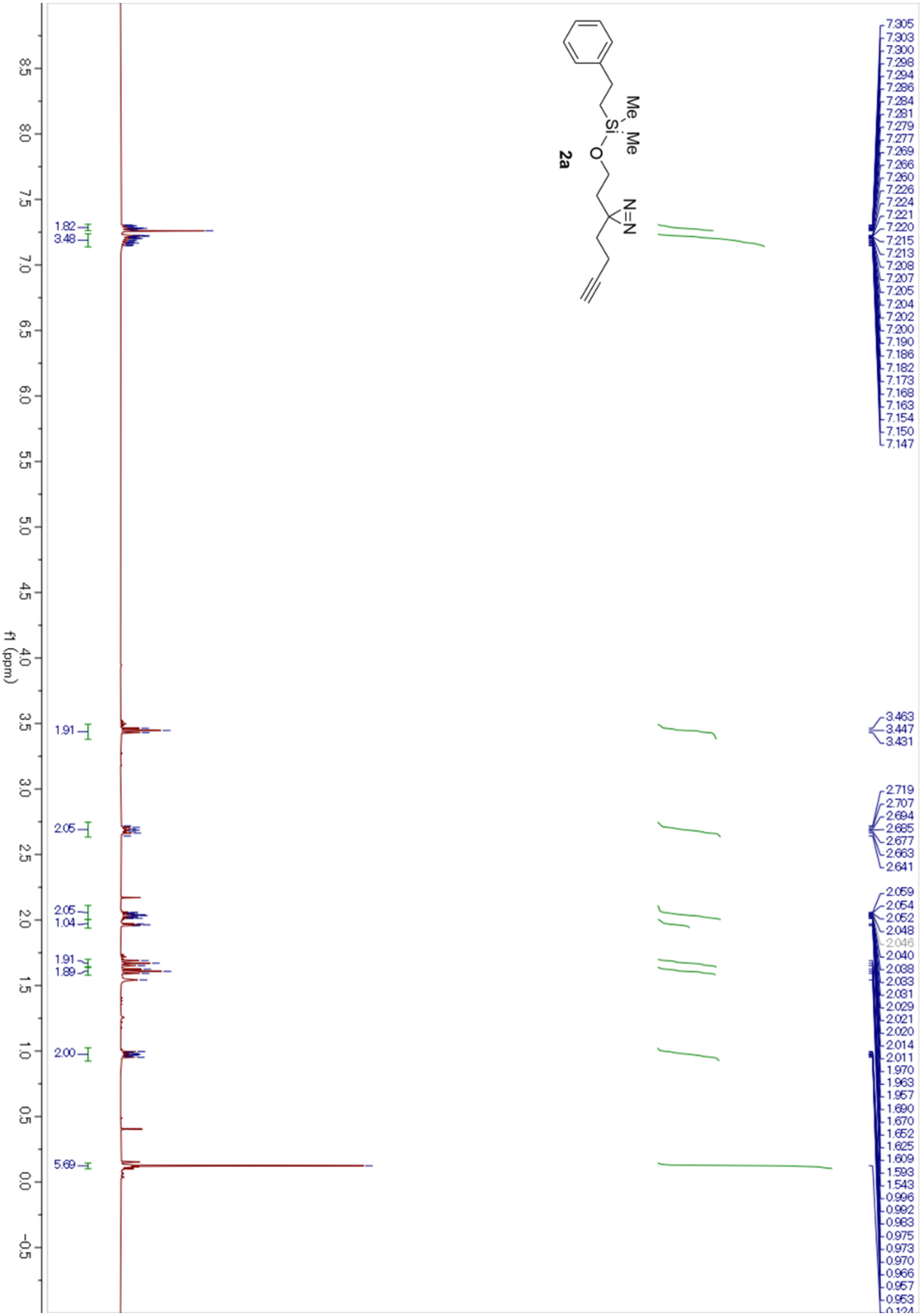
2a
Me
Me
Si
O
N=N
7.305
7.303
7.300
7.298
7.294
7.286
7.284
7.281
7.279
7.277
7.269
7.266
7.260
7.226
7.224
7.221
7.220
7.215
7.213
7.208
7.207
7.205
7.204
7.202
7.200
7.190
7.186
7.182
7.173
7.168
7.163
7.154
7.150
7.147
3.463
3.447
3.431
2.719
2.707
2.694
2.685
2.677
2.663
2.641
2.059
2.054
2.052
2.048
2.046
2.040
2.038
2.033
2.031
2.029
2.021
2.020
2.014
2.011
1.970
1.963
1.957
1.690
1.670
1.652
1.625
1.609
1.593
1.543
0.996
0.992
0.983
0.975
0.973
0.970
0.966
0.957
0.953
1.82
3.48
1.91
2.05
2.05
1.04
1.91
1.89
2.00
5.69
f1 (ppm)

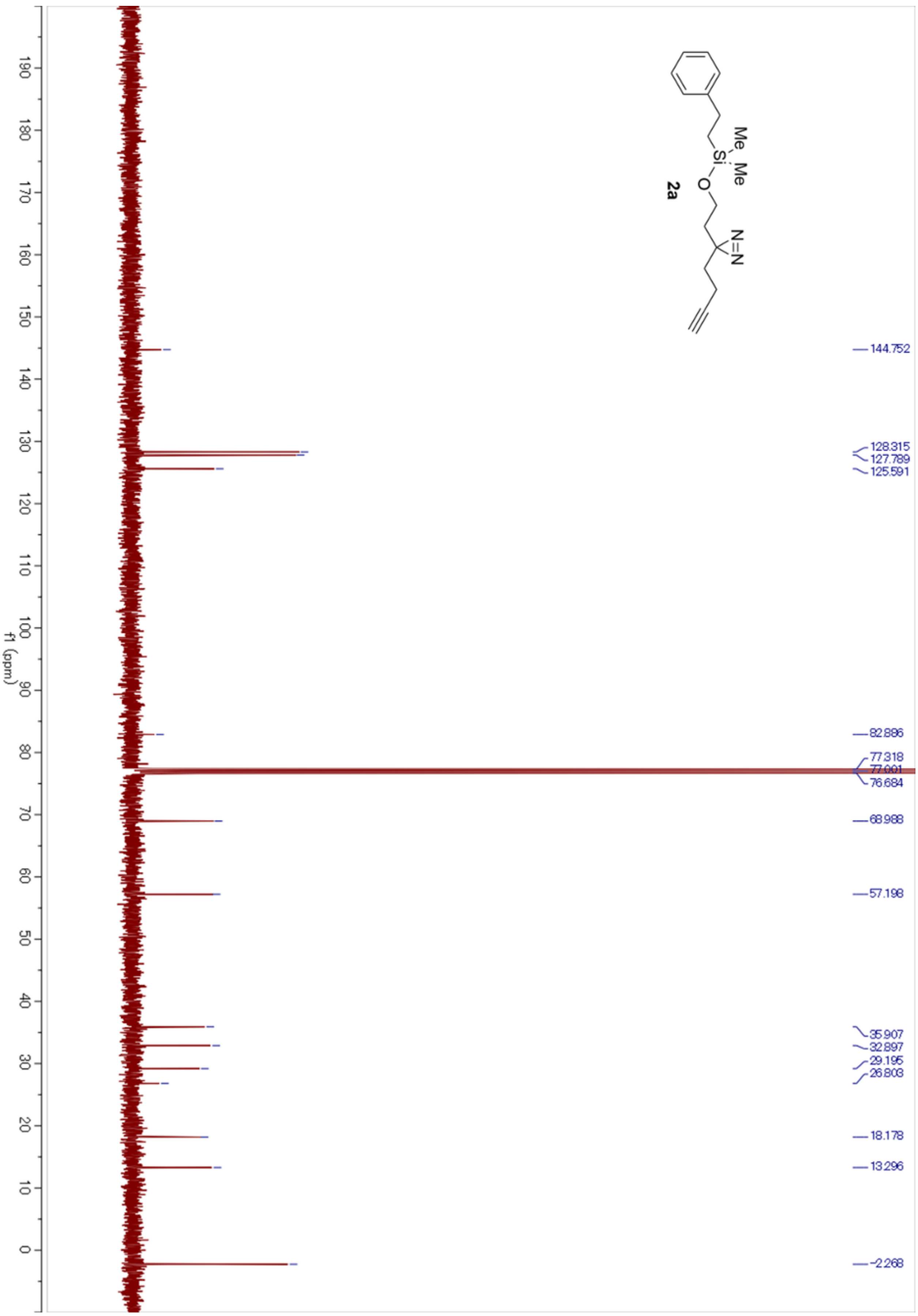
Me
Me
Si
O
N=N
2a
144.752
128.315
127.789
125.591
82.886
77.318
77.001
76.684
68.988
57.198
35.907
32.897
29.195
26.803
18.178
13.296
-2.268
f1 (ppm)
190
180
170
160
150
140
130
120
110
100
90
80
70
60
50
40
30
20
10
0

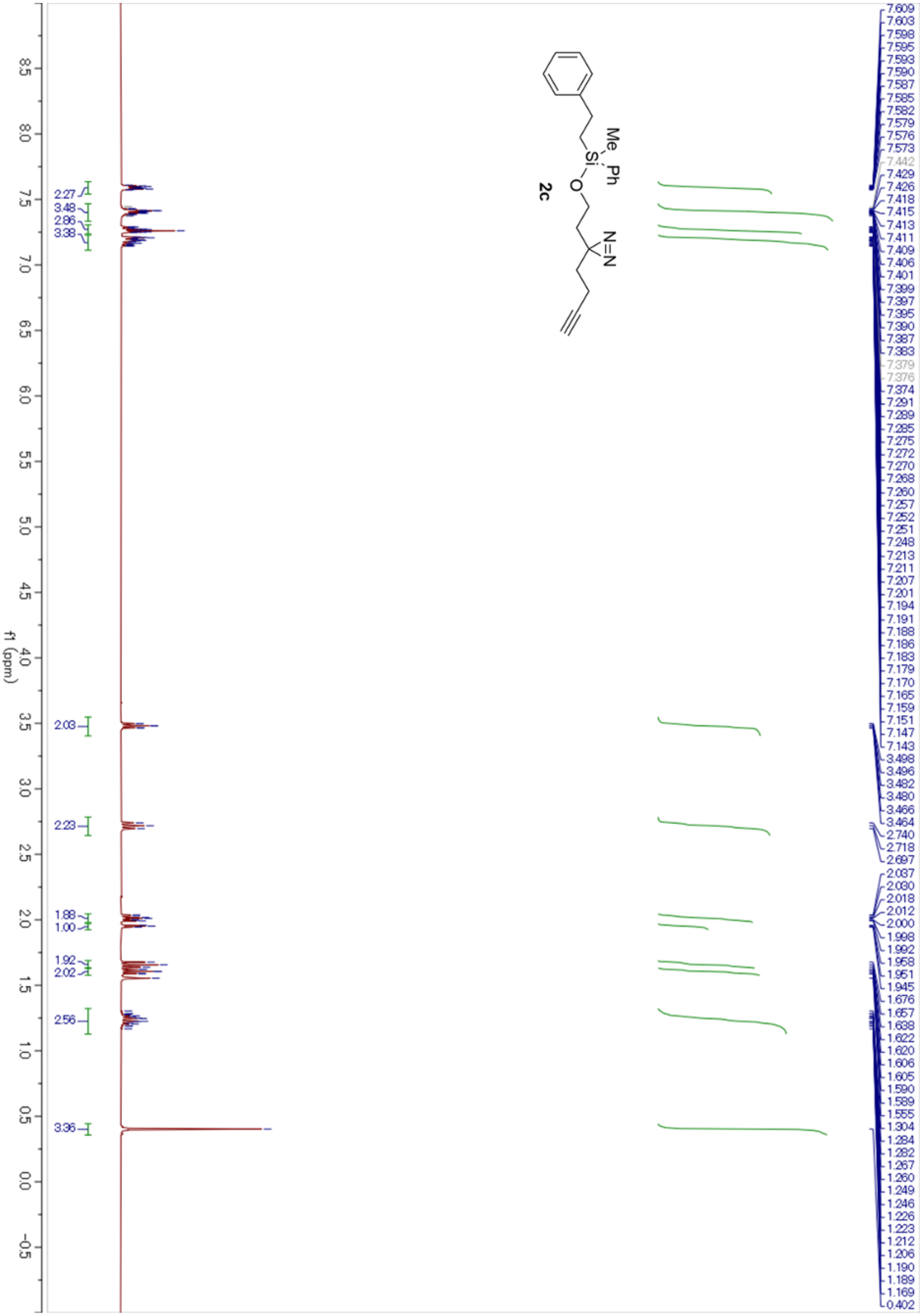
2c
f1 (ppm)

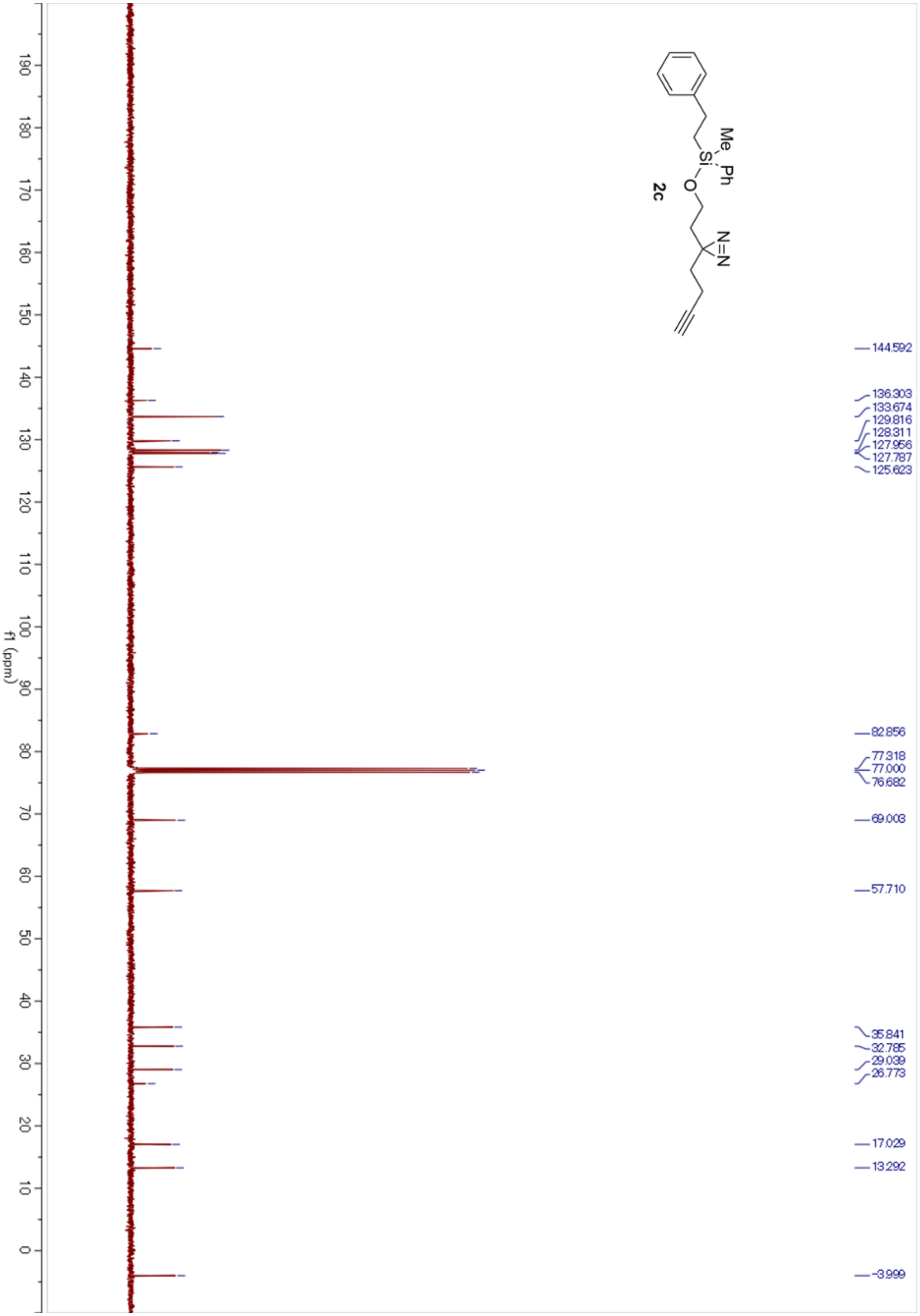
Me
Ph
Si
O
N=N
2c
144.592
136.303
133.674
129.816
128.311
127.956
127.787
125.623
82.856
77.318
77.000
76.682
69.003
57.710
35.841
32.785
29.039
26.773
17.029
13.292
-3.999
f1 (ppm)

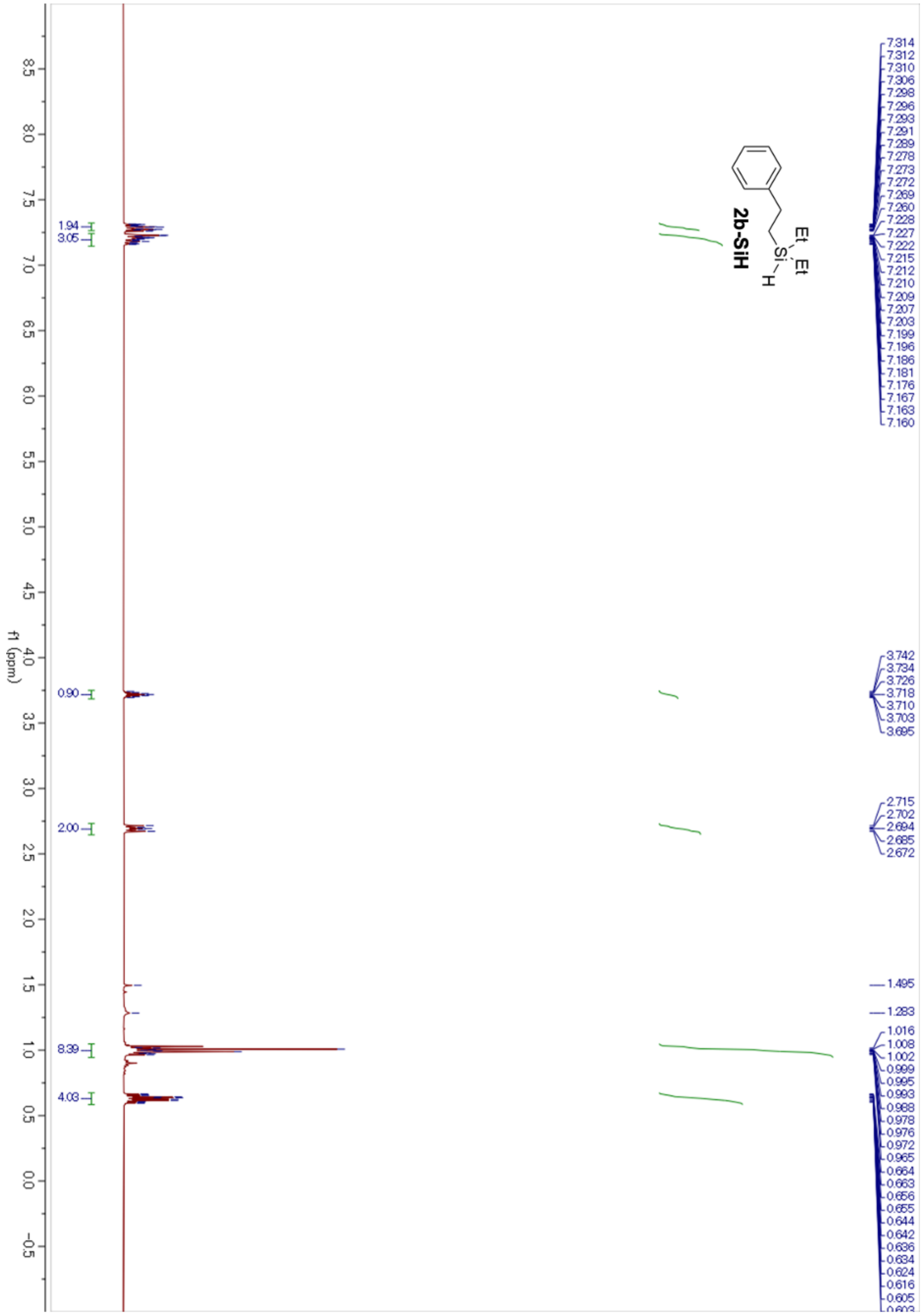

2b-SiH
Et
Et
Si
H
7.314
7.312
7.310
7.306
7.298
7.296
7.293
7.291
7.289
7.278
7.273
7.272
7.269
7.260
7.228
7.227
7.222
7.215
7.212
7.210
7.209
7.207
7.203
7.199
7.196
7.186
7.181
7.176
7.167
7.163
7.160
3.742
3.734
3.726
3.718
3.710
3.703
3.695
2.715
2.702
2.694
2.685
2.672
1.495
1.283
1.016
1.008
1.002
0.999
0.995
0.993
0.988
0.978
0.976
0.972
0.965
0.664
0.663
0.656
0.655
0.644
0.642
0.636
0.634
0.624
0.616
0.605
1.94
3.05
0.90
2.00
8.39
4.03
f1 (ppm)

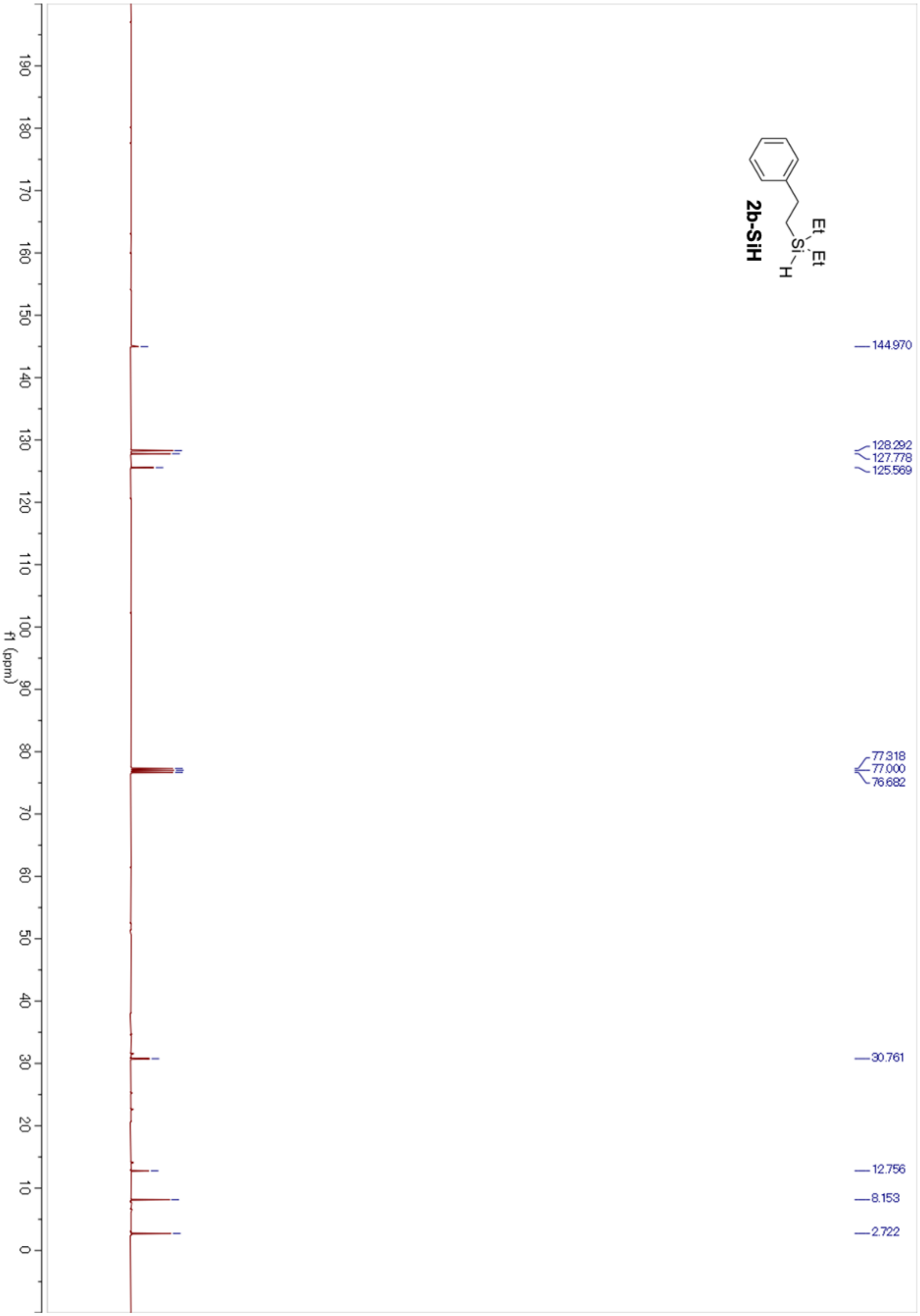
Et
Et
Si
H
2b-SiH
144.970
128.292
127.778
125.569
77.318
77.000
76.682
30.761
12.756
8.153
2.722
f1 (ppm)
190
180
170
160
150
140
130
120
110
100
90
80
70
60
50
40
30
20
10
0

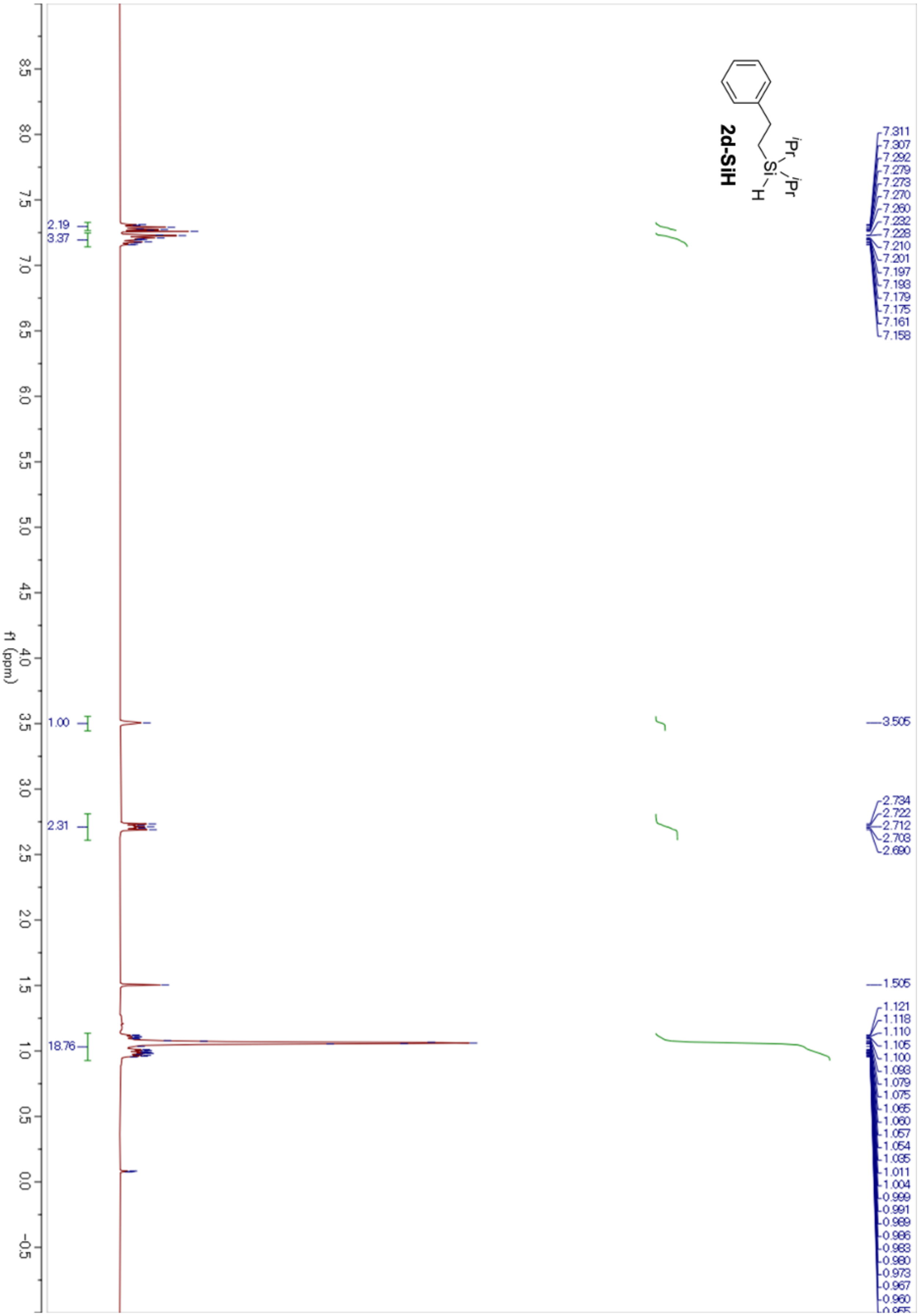
2d-SiH
f1 (ppm)

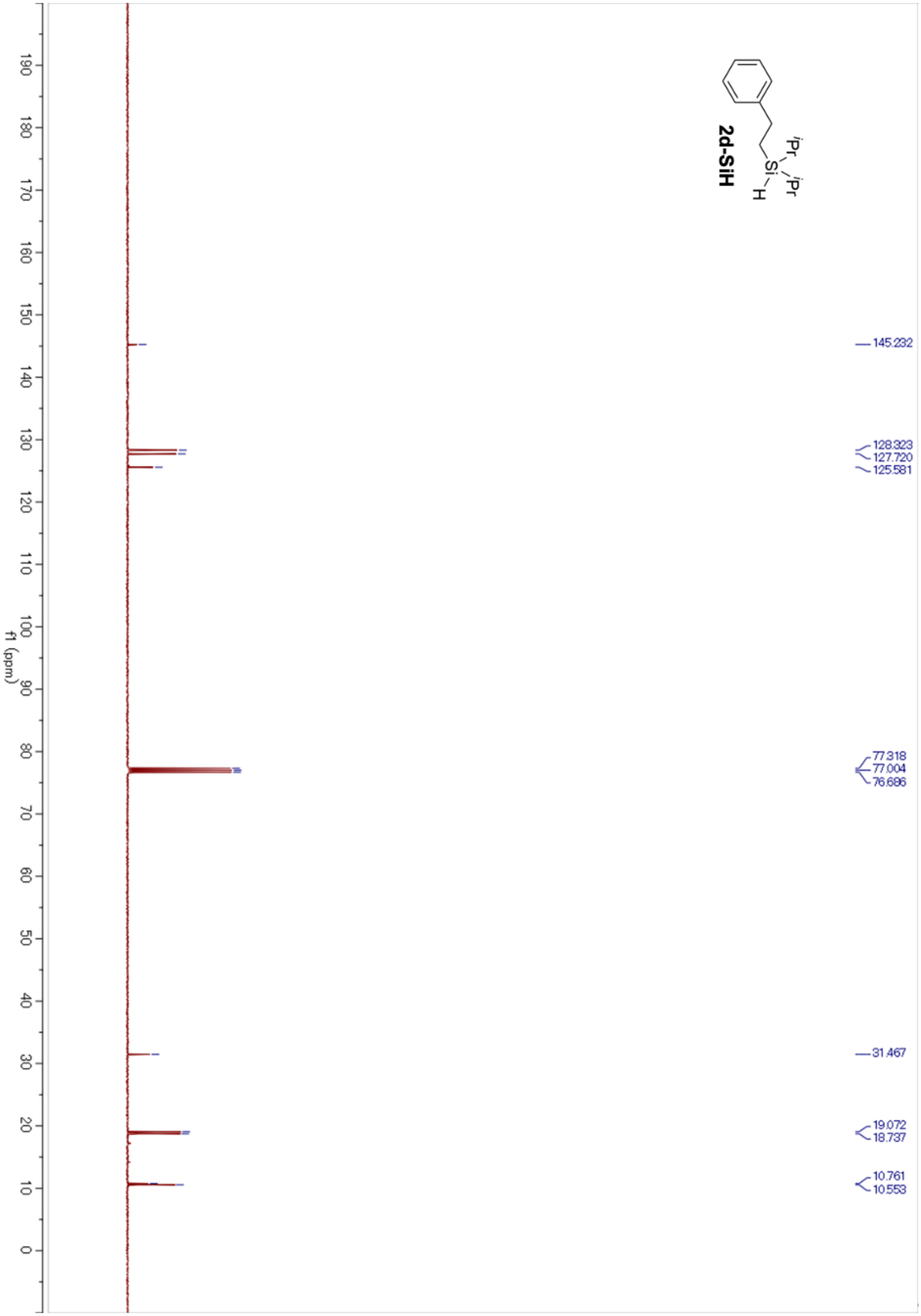

2d-SiH
iPr
iPr
Si
H
145.232
128.323
127.720
125.581
77.318
77.004
76.686
31.467
19.072
18.737
10.761
10.553
f1 (ppm)

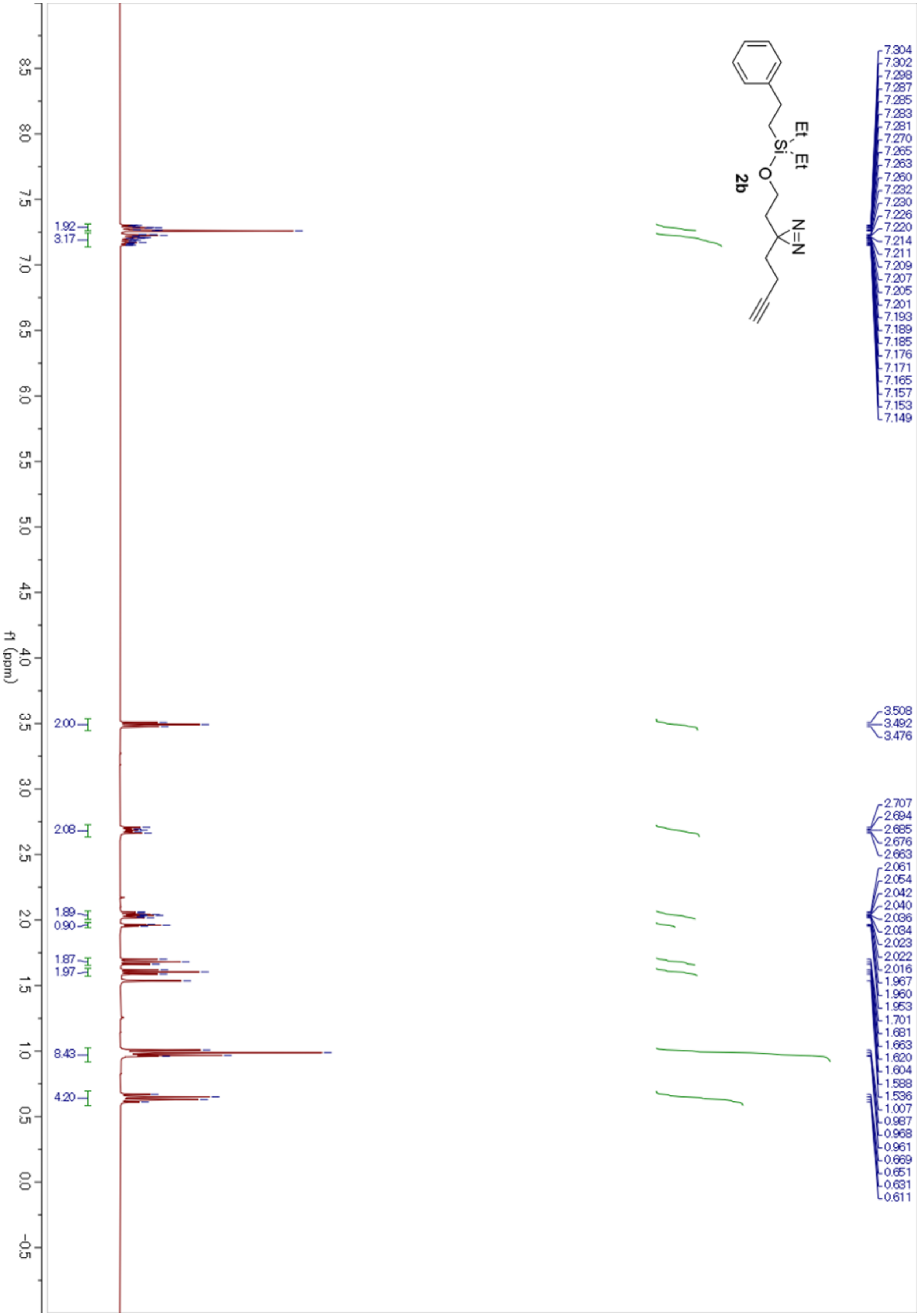

2b
Et
Et
Si
O
N=N
f1 (ppm)
8.5
8.0
7.5
7.0
6.5
6.0
5.5
5.0
4.5
4.0
3.5
3.0
2.5
2.0
1.5
1.0
0.5
0.0
-0.5
1.92
3.17
2.00
2.08
1.89
0.90
1.87
1.97
8.43
4.20
7.304
7.302
7.298
7.287
7.285
7.283
7.281
7.270
7.265
7.263
7.260
7.232
7.230
7.226
7.220
7.214
7.211
7.209
7.207
7.205
7.201
7.193
7.189
7.185
7.176
7.171
7.165
7.157
7.153
7.149
3.508
3.492
3.476
2.707
2.694
2.685
2.676
2.663
2.061
2.054
2.042
2.040
2.036
2.034
2.023
2.022
2.016
1.967
1.960
1.953
1.701
1.681
1.663
1.620
1.604
1.588
1.536
1.007
0.987
0.968
0.961
0.669
0.651
0.631
0.611

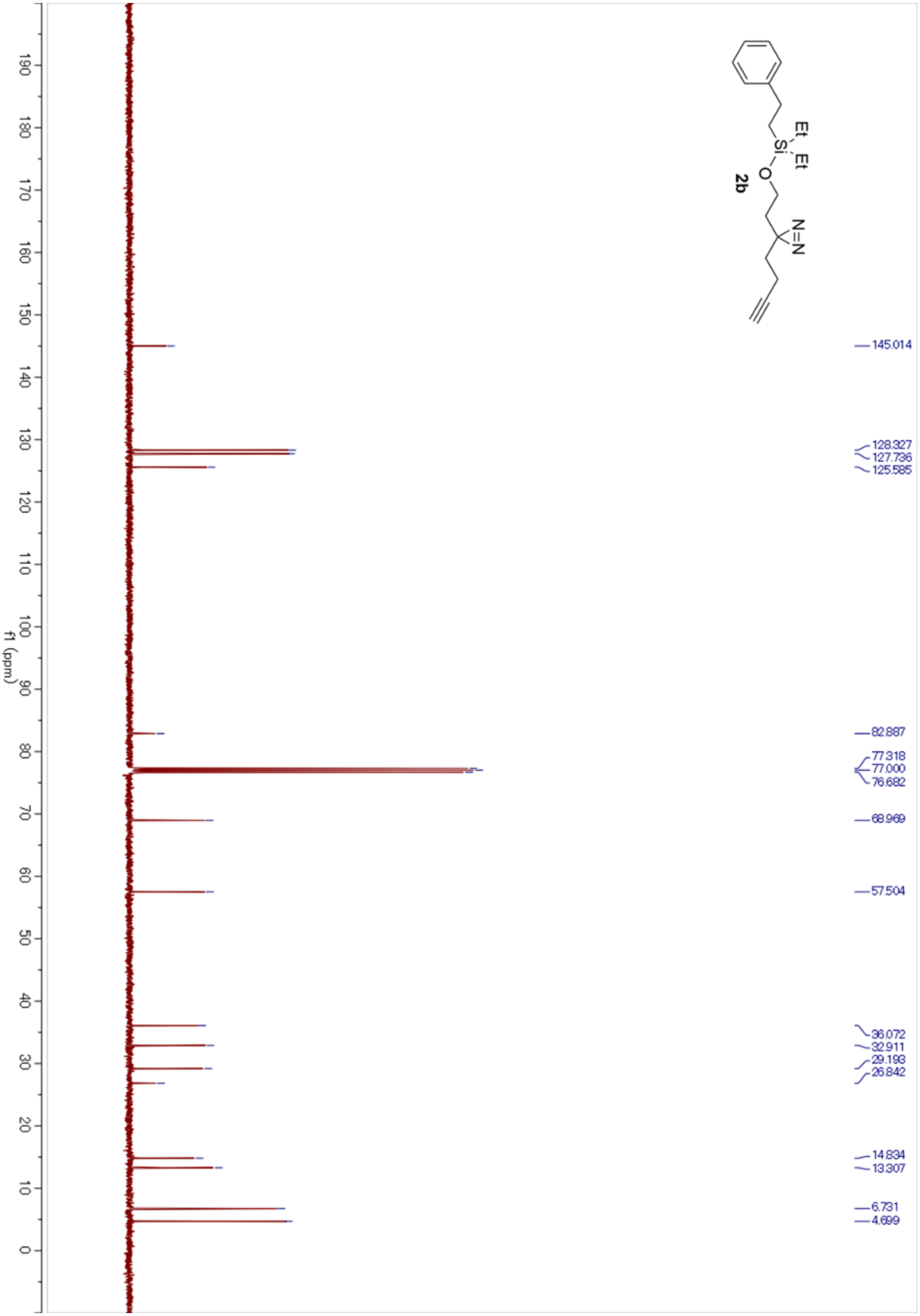
2b
Et
Et
Si
O
N=N
145.014
128.327
127.736
125.585
82.887
77.318
77.000
76.682
68.969
57.504
36.072
32.911
29.193
26.842
14.834
13.307
6.731
4.699
f1 (ppm)
190
180
170
160
150
140
130
120
110
100
90
80
70
60
50
40
30
20
10
0

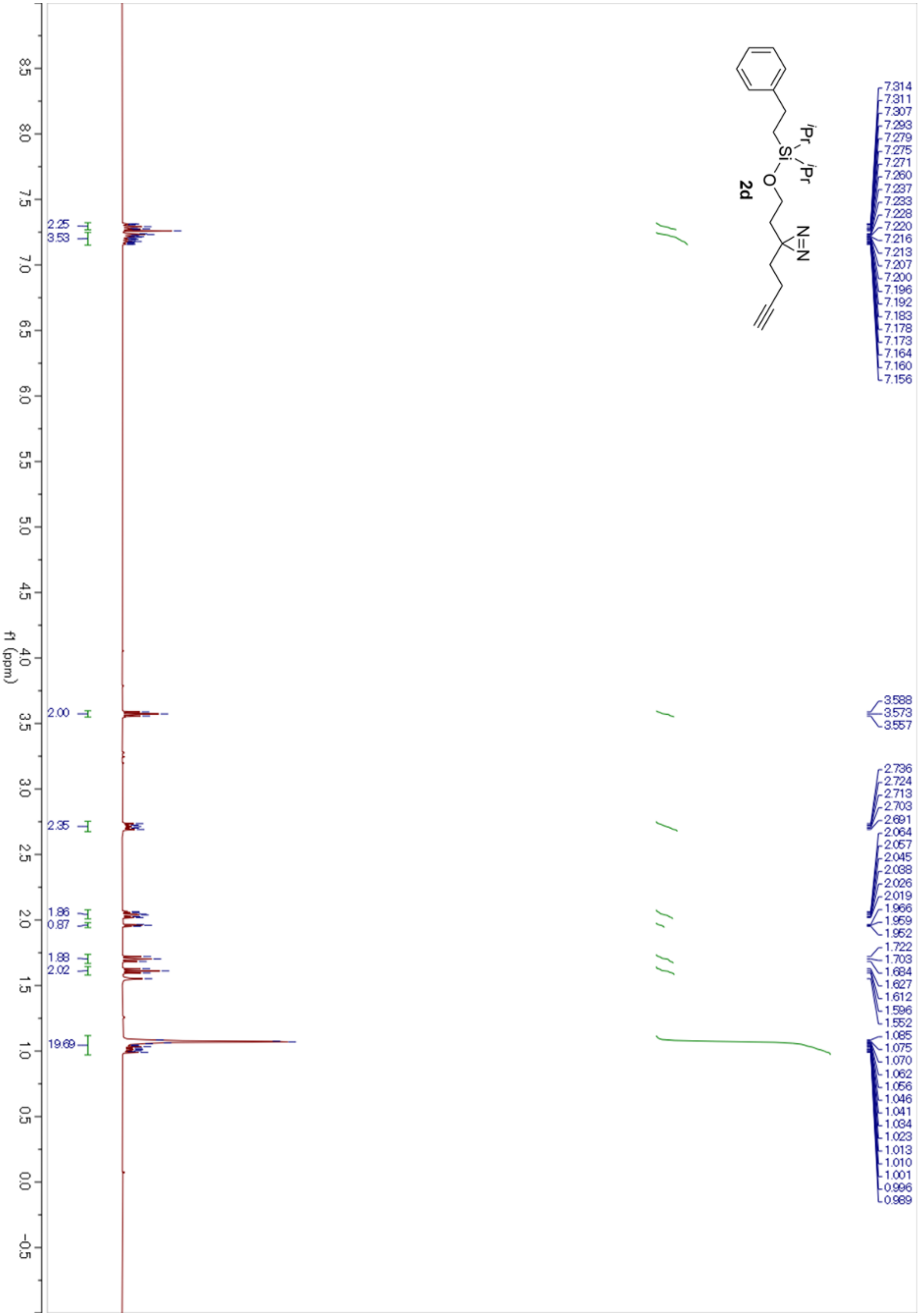

iPr
iPr
Si
O
N=N
2d
7.314
7.311
7.307
7.293
7.279
7.275
7.271
7.260
7.237
7.233
7.228
7.220
7.216
7.213
7.207
7.200
7.196
7.192
7.183
7.178
7.173
7.164
7.160
7.156
3.588
3.573
3.557
2.736
2.724
2.713
2.703
2.691
2.064
2.057
2.045
2.038
2.026
2.019
1.966
1.959
1.952
1.722
1.703
1.684
1.627
1.612
1.596
1.552
1.085
1.075
1.070
1.062
1.056
1.046
1.041
1.034
1.023
1.013
1.010
1.001
0.996
0.989
2.25
3.53
2.00
2.35
1.86
0.87
1.88
2.02
19.69
8.5
8.0
7.5
7.0
6.5
6.0
5.5
5.0
4.5
4.0
3.5
3.0
2.5
2.0
1.5
1.0
0.5
0.0
-0.5
f1 (ppm)

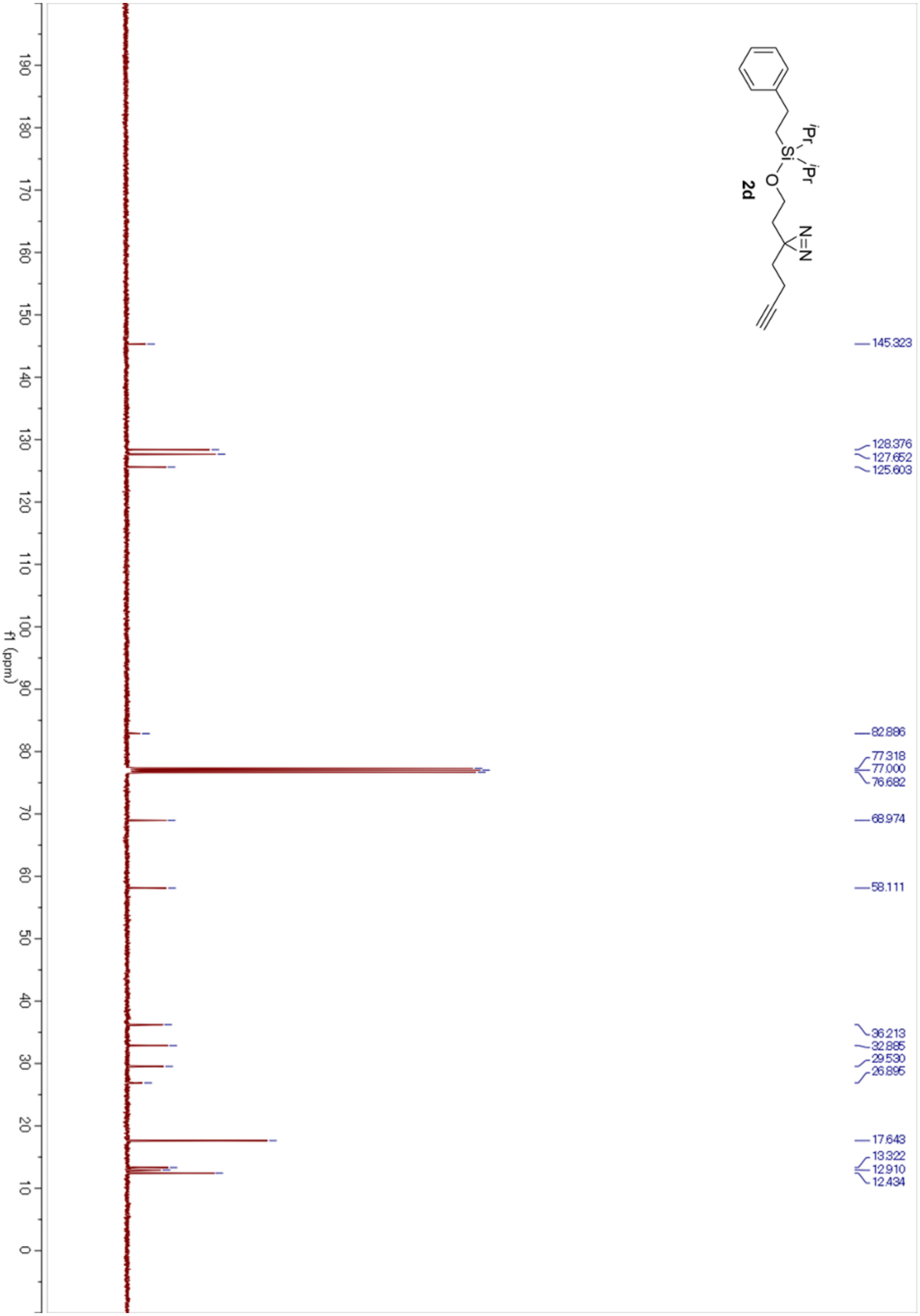

2d
iPr
iPr
Si
O
N=N
145.323
128.376
127.652
125.603
82.886
77.318
77.000
76.682
68.974
58.111
36.213
32.885
29.530
26.895
17.643
13.322
12.910
12.434
f1 (ppm)
190
180
170
160
150
140
130
120
110
100
90
80
70
60
50
40
30
20
10
0

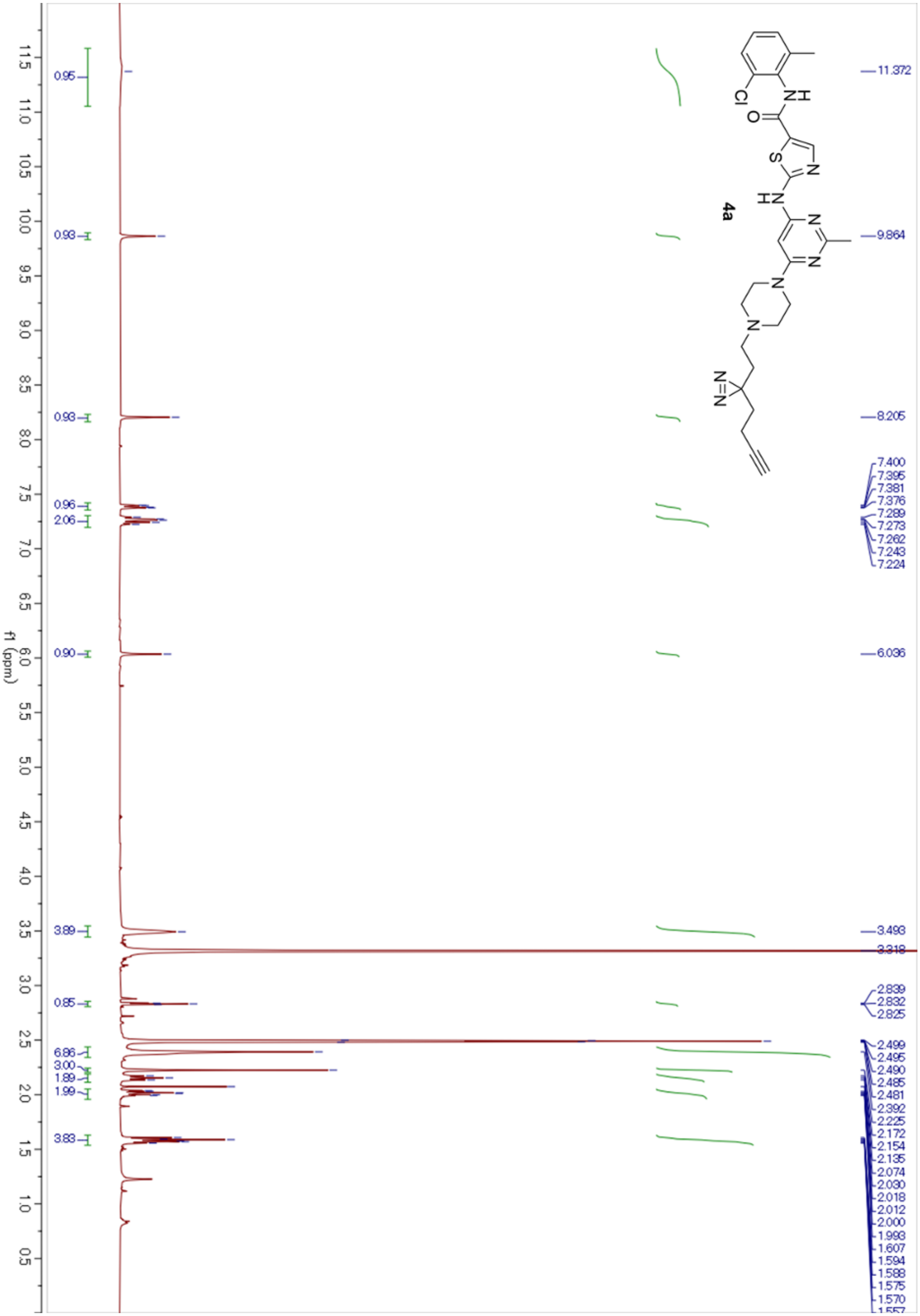
4a
f1 (ppm)
11.5
11.0
10.5
10.0
9.5
9.0
8.5
8.0
7.5
7.0
6.5
6.0
5.5
5.0
4.5
4.0
3.5
3.0
2.5
2.0
1.5
1.0
0.5
0.95
0.93
0.93
0.96
2.06
0.90
3.89
0.85
6.86
3.00
1.89
1.99
3.83
11.372
9.864
8.205
7.400
7.395
7.381
7.376
7.289
7.273
7.262
7.243
7.224
6.036
3.493
3.318
2.839
2.832
2.825
2.499
2.495
2.490
2.485
2.481
2.392
2.225
2.172
2.154
2.135
2.074
2.030
2.018
2.012
2.000
1.993
1.607
1.594
1.588
1.575
1.570

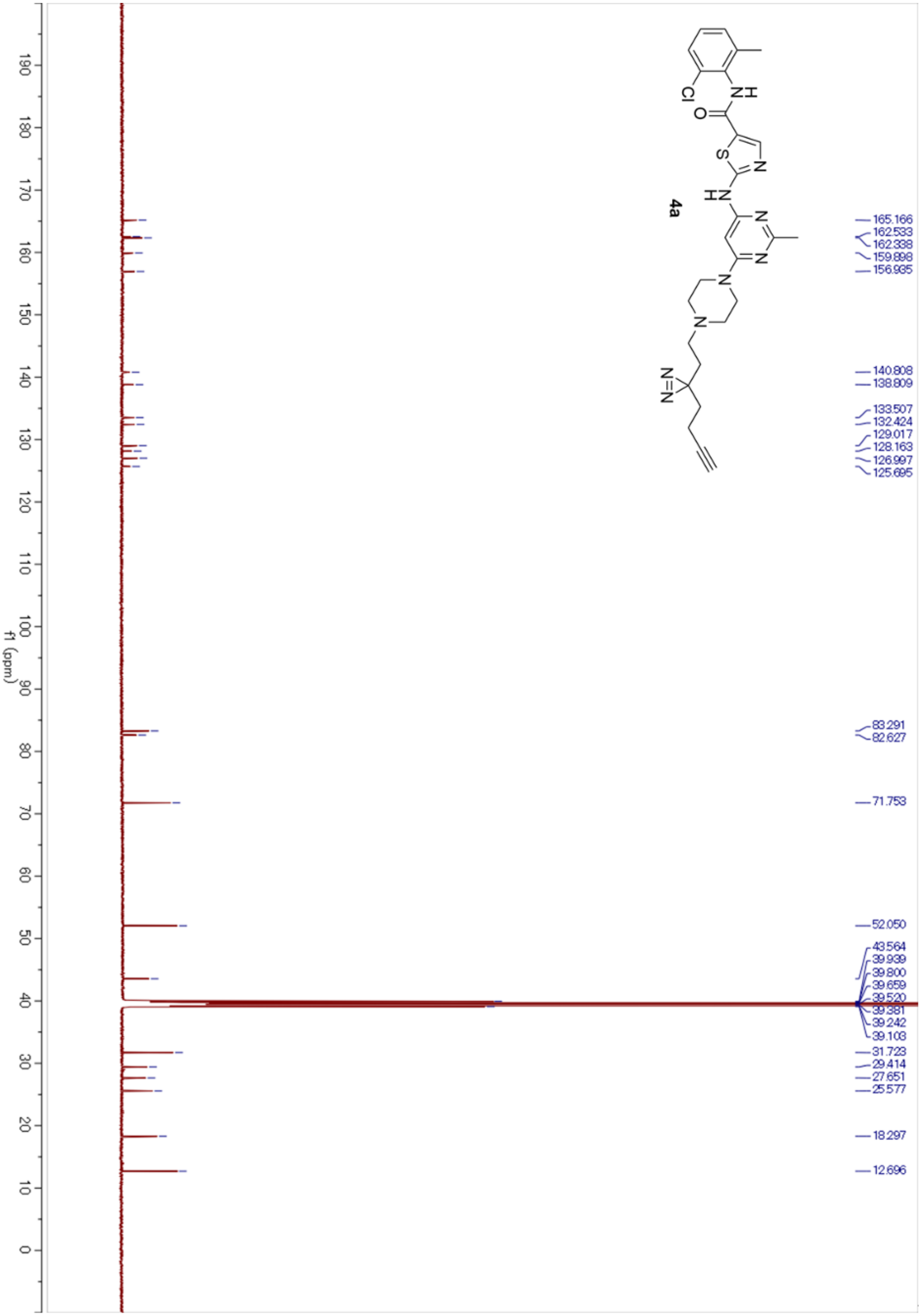
4a
165.166
162.533
162.338
159.898
156.935
140.808
138.809
133.507
132.424
129.017
128.163
126.997
125.695
83.291
82.627
71.753
52.050
43.564
39.939
39.800
39.659
39.520
39.381
39.242
39.103
31.723
29.414
27.651
25.577
18.297
12.696
f1 (ppm)

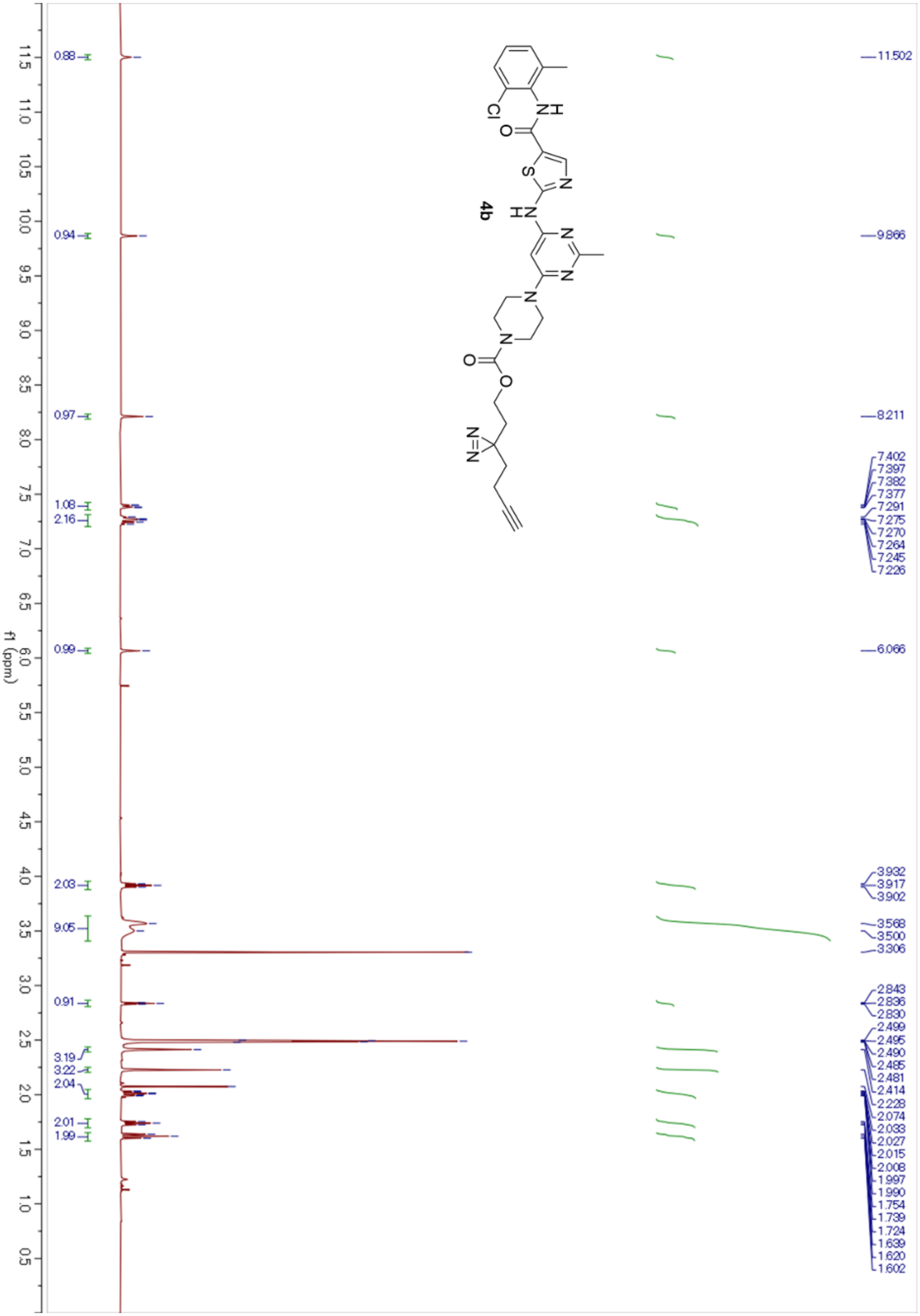
4b
f1 (ppm)

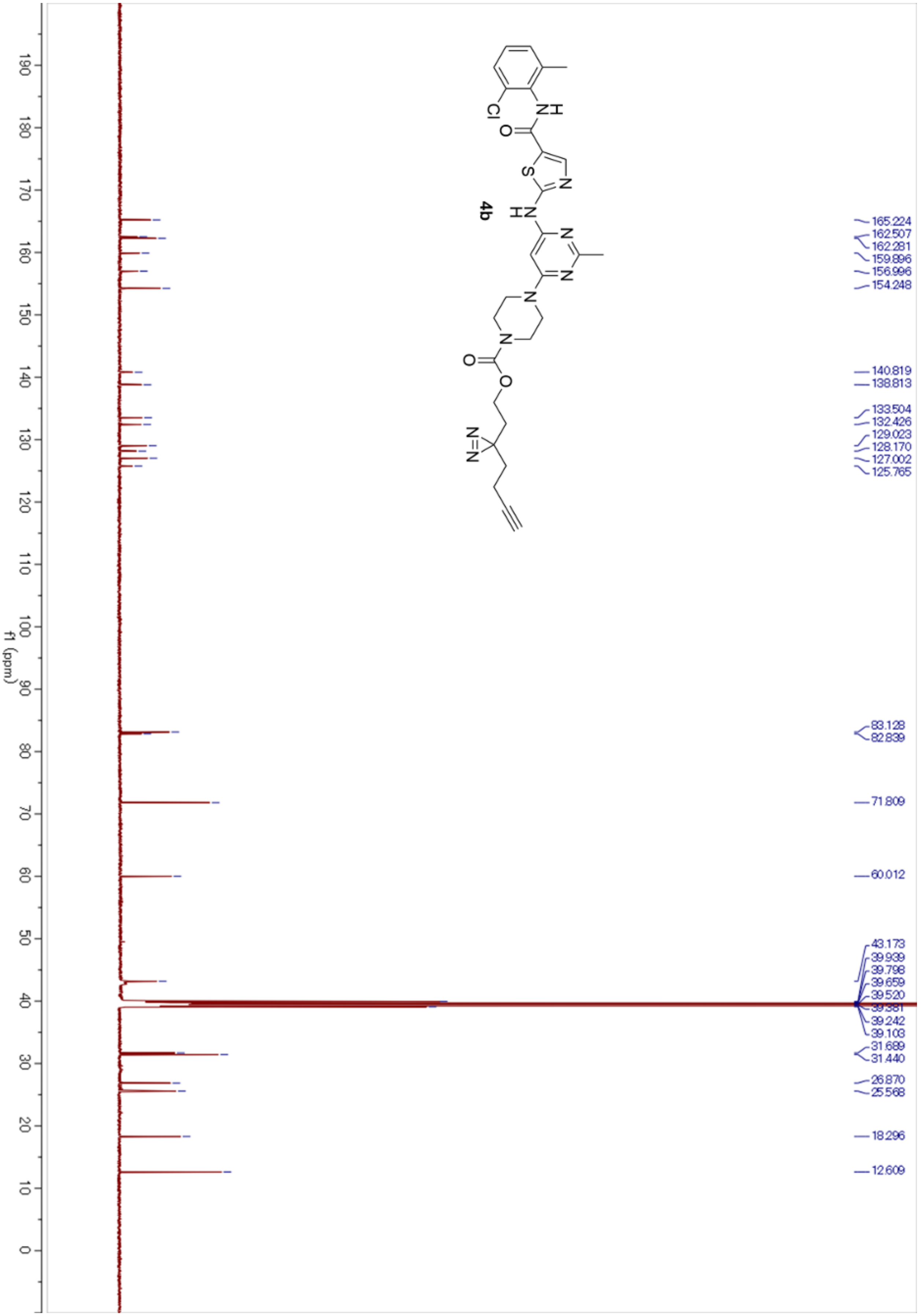
4b
165.224
162.507
162.281
159.896
156.996
154.248
140.819
138.813
133.504
132.426
129.023
128.170
127.002
125.765
83.128
82.839
71.809
60.012
43.173
39.939
39.798
39.659
39.520
39.381
39.242
39.103
31.689
31.440
26.870
25.568
18.296
12.609
f1 (ppm)

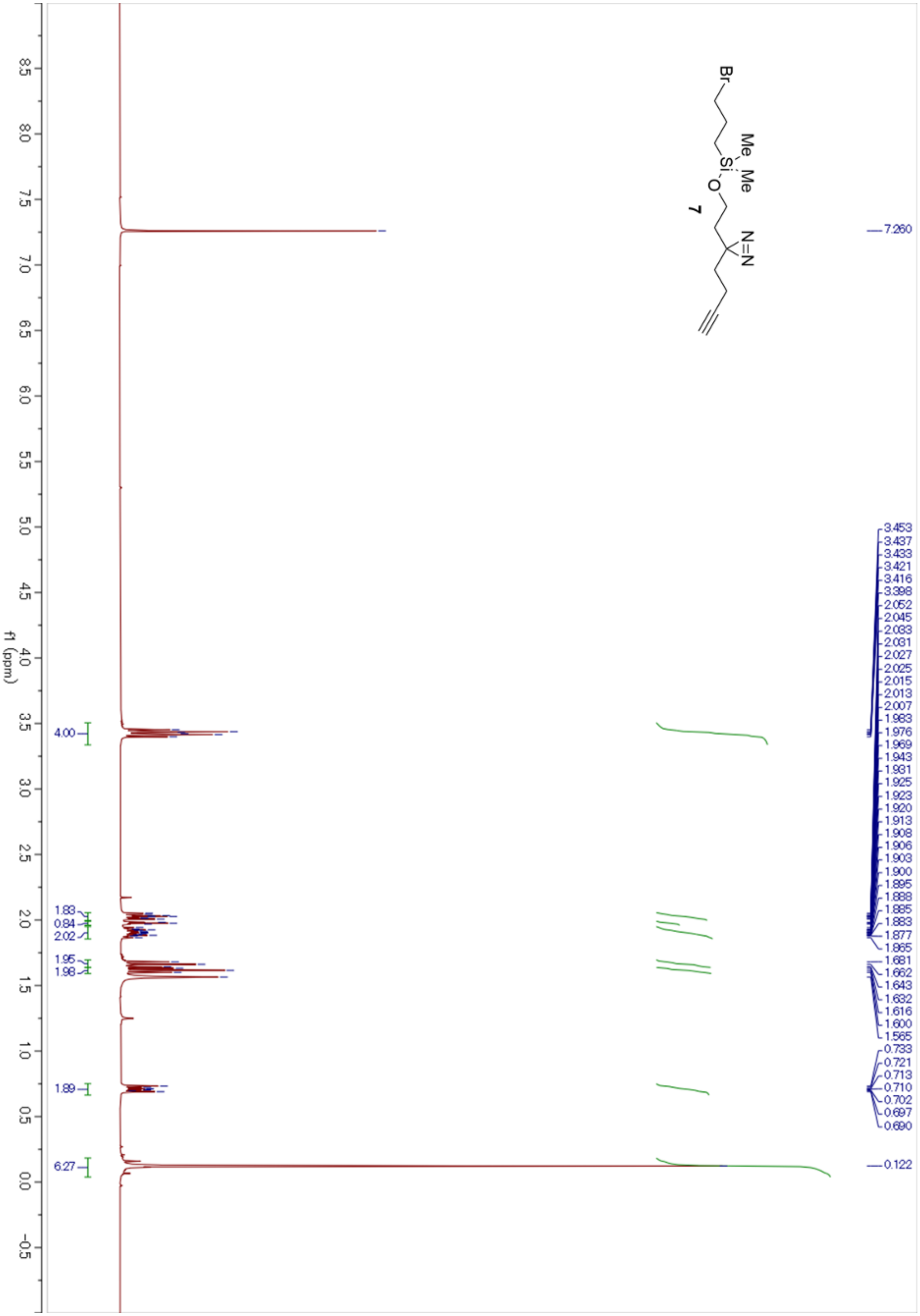
Br
Me Me
Si
O
N=N
7
7.260
3.453
3.437
3.433
3.421
3.416
3.398
2.052
2.045
2.033
2.031
2.027
2.025
2.015
2.013
2.007
1.983
1.976
1.969
1.943
1.931
1.925
1.923
1.920
1.913
1.908
1.906
1.903
1.900
1.895
1.888
1.885
1.883
1.877
1.865
1.681
1.662
1.643
1.632
1.616
1.600
1.565
0.733
0.721
0.713
0.710
0.702
0.697
0.690
0.122
4.00
1.83
0.84
2.02
1.95
1.98
1.89
6.27
f1 (ppm)

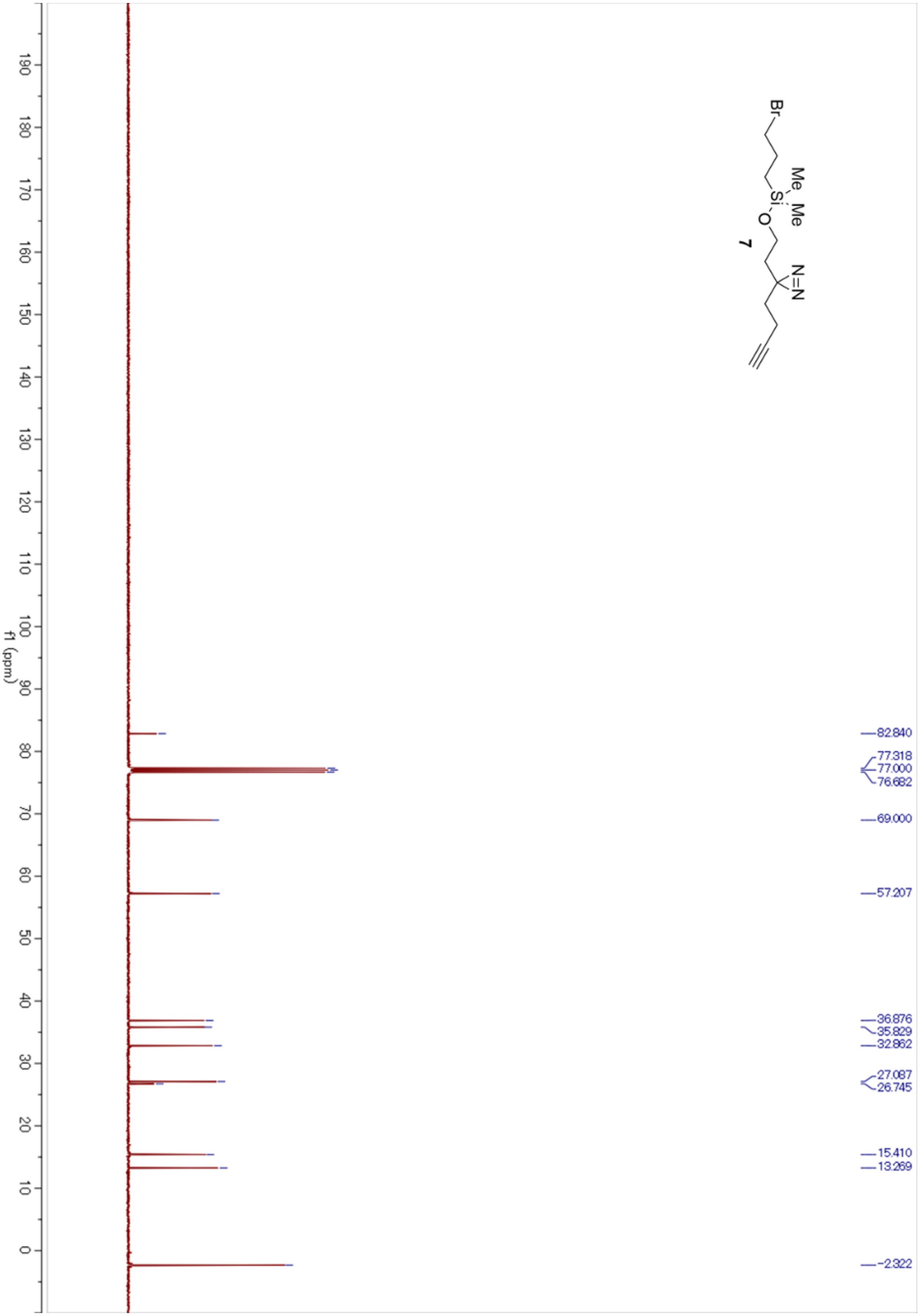
Br
Me
Me
Si
O
N=N
7
f1 (ppm)
82.840
77.318
77.000
76.682
69.000
57.207
36.876
35.829
32.862
27.087
26.745
15.410
13.269
-2.322

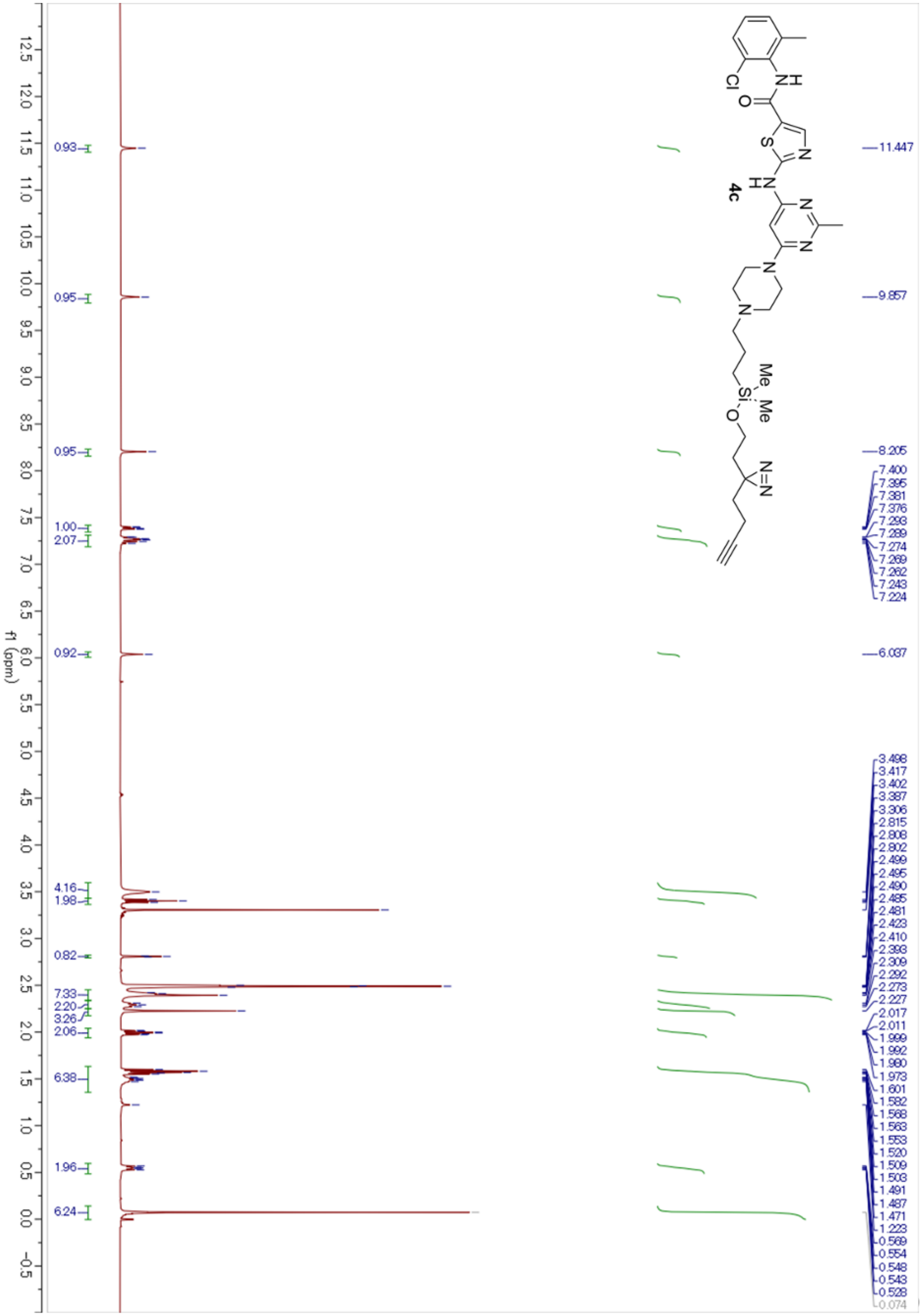
4c
Me Me
Si
O
N=N
H
N
O
Cl
S
N
N
H
11.447
9.857
8.205
7.400
7.395
7.381
7.376
7.293
7.289
7.274
7.269
7.262
7.243
7.224
6.037
3.498
3.417
3.402
3.387
3.306
2.815
2.808
2.802
2.499
2.495
2.490
2.485
2.481
2.423
2.410
2.393
2.309
2.292
2.273
2.227
2.017
2.011
1.999
1.992
1.980
1.973
1.601
1.582
1.568
1.563
1.553
1.520
1.509
1.503
1.491
1.487
1.471
1.223
0.569
0.554
0.548
0.543
0.528
0.074
0.93
0.95
0.95
1.00
2.07
0.92
4.16
1.98
0.82
7.33
2.20
3.26
2.06
6.38
1.96
6.24
f1 (ppm)

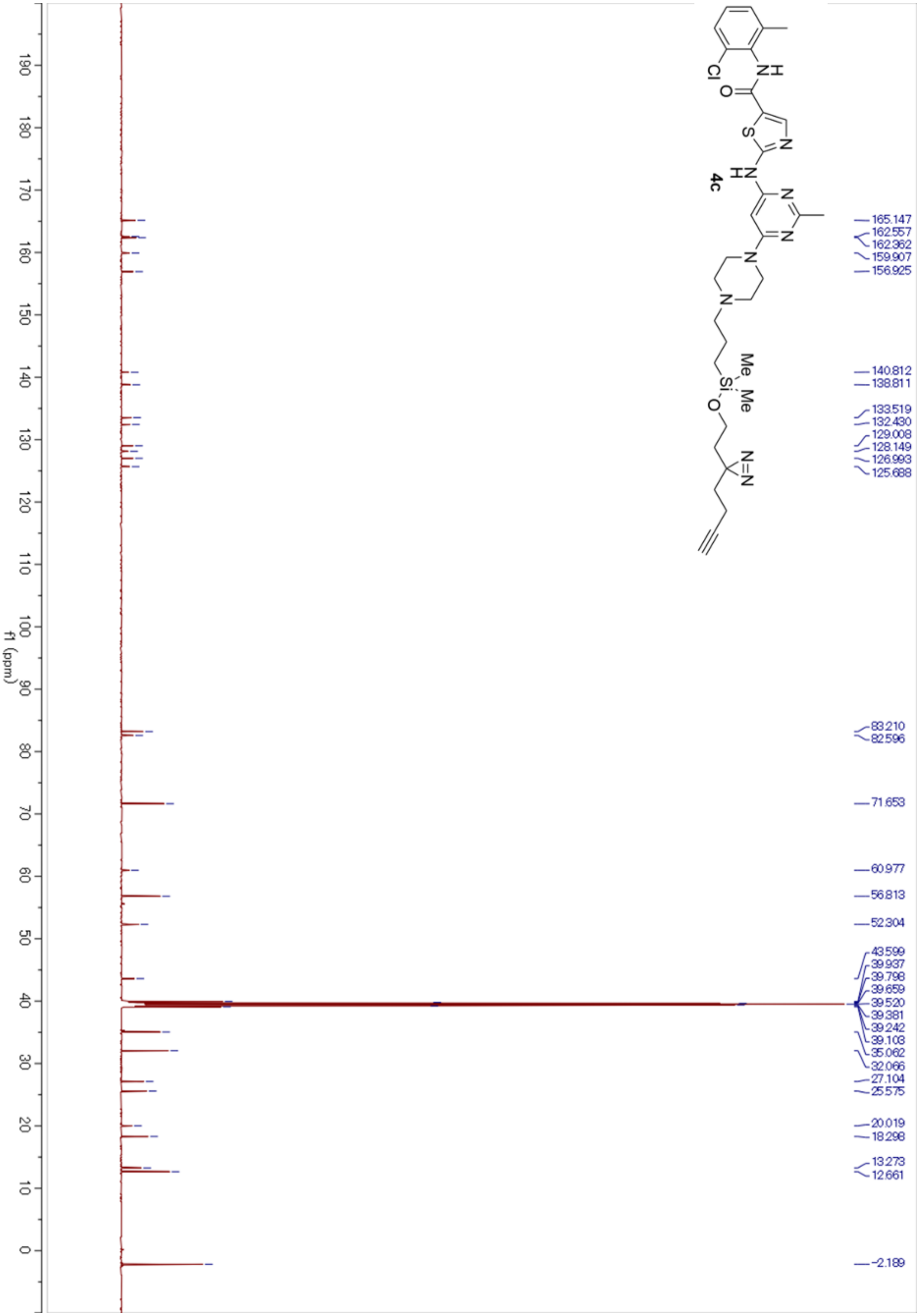
4c
165.147
162.557
162.362
159.907
156.925
140.812
138.811
133.519
132.430
129.008
128.149
126.993
125.688
83.210
82.596
71.653
60.977
56.813
52.304
43.599
39.937
39.798
39.659
39.520
39.381
39.242
39.103
35.062
32.066
27.104
25.575
20.019
18.298
13.273
12.661
-2.189
f1 (ppm)

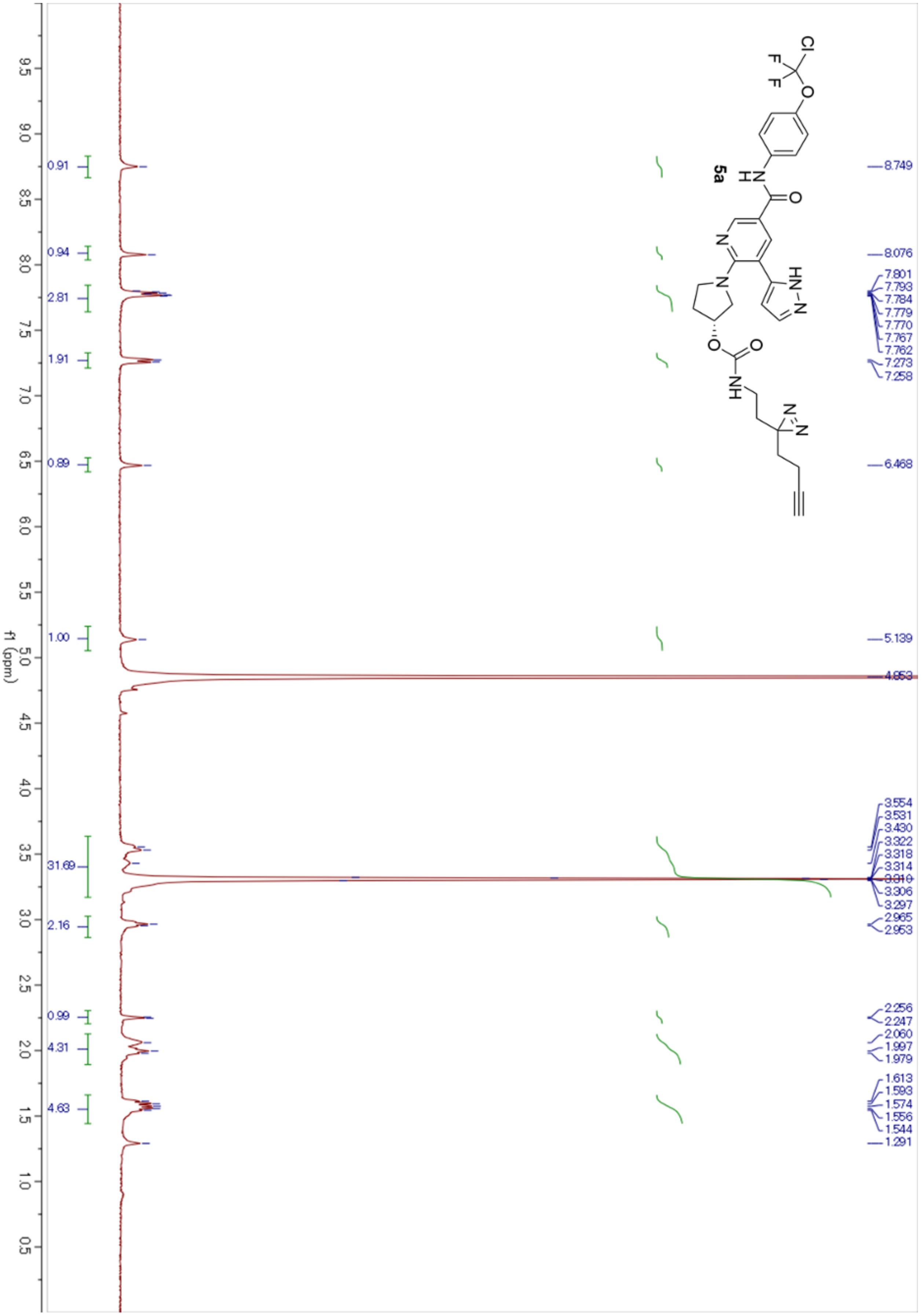
5a
f1 (ppm)
8.749
8.076
7.801
7.793
7.784
7.779
7.770
7.767
7.762
7.273
7.258
6.468
5.139
4.853
3.554
3.531
3.430
3.322
3.318
3.314
3.310
3.306
3.297
2.965
2.953
2.256
2.247
2.060
1.997
1.979
1.613
1.593
1.574
1.556
1.544
1.291
0.91
0.94
2.81
1.91
0.89
1.00
31.69
2.16
0.99
4.31
4.63

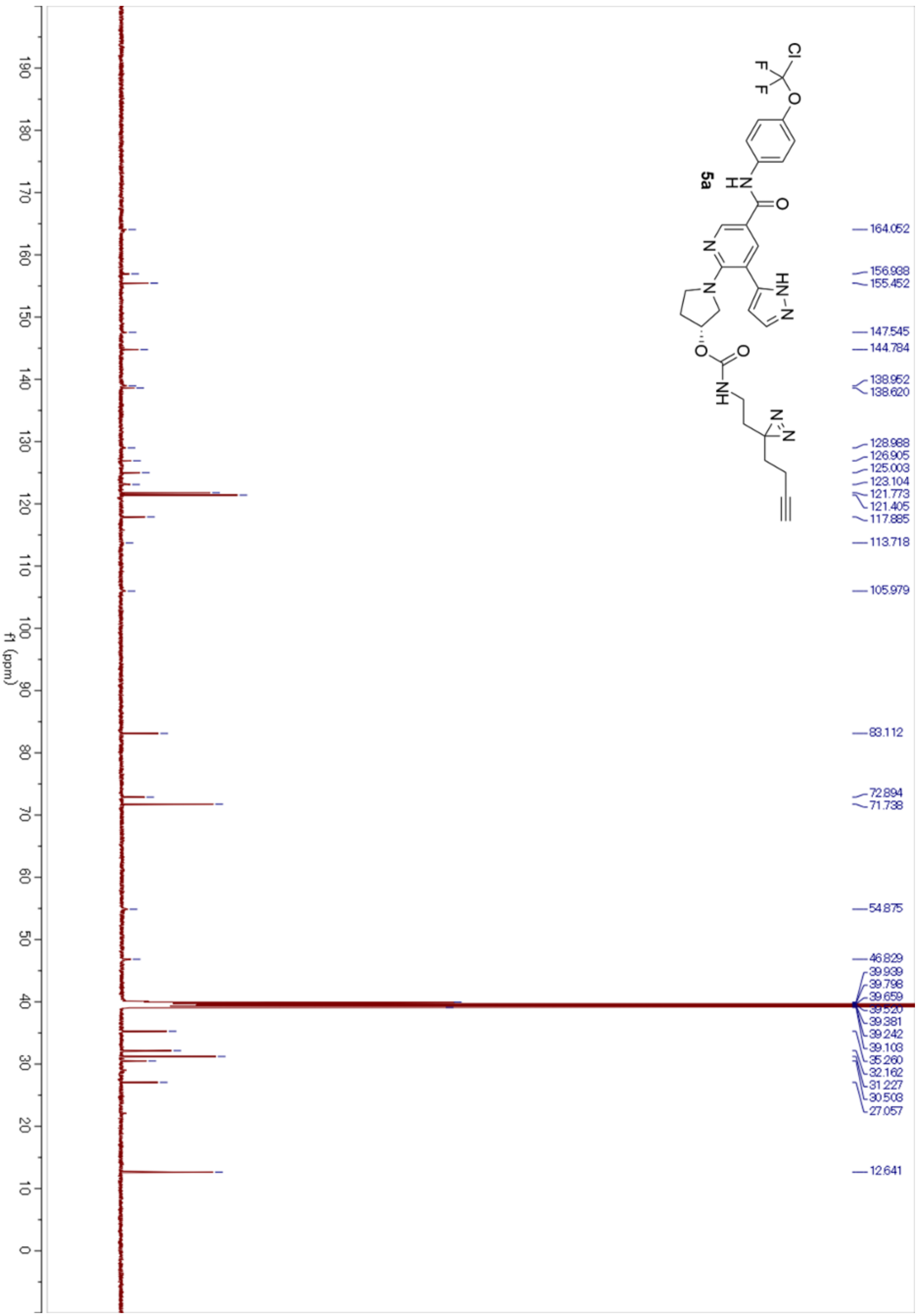
5a
164.052
156.938
155.452
147.545
144.784
138.952
138.620
128.968
126.905
125.003
123.104
121.773
121.405
117.885
113.718
105.979
83.112
72.894
71.738
54.875
46.829
39.939
39.798
39.659
39.520
39.381
39.242
39.103
35.260
32.162
31.227
30.503
27.057
12.641
f1 (ppm)

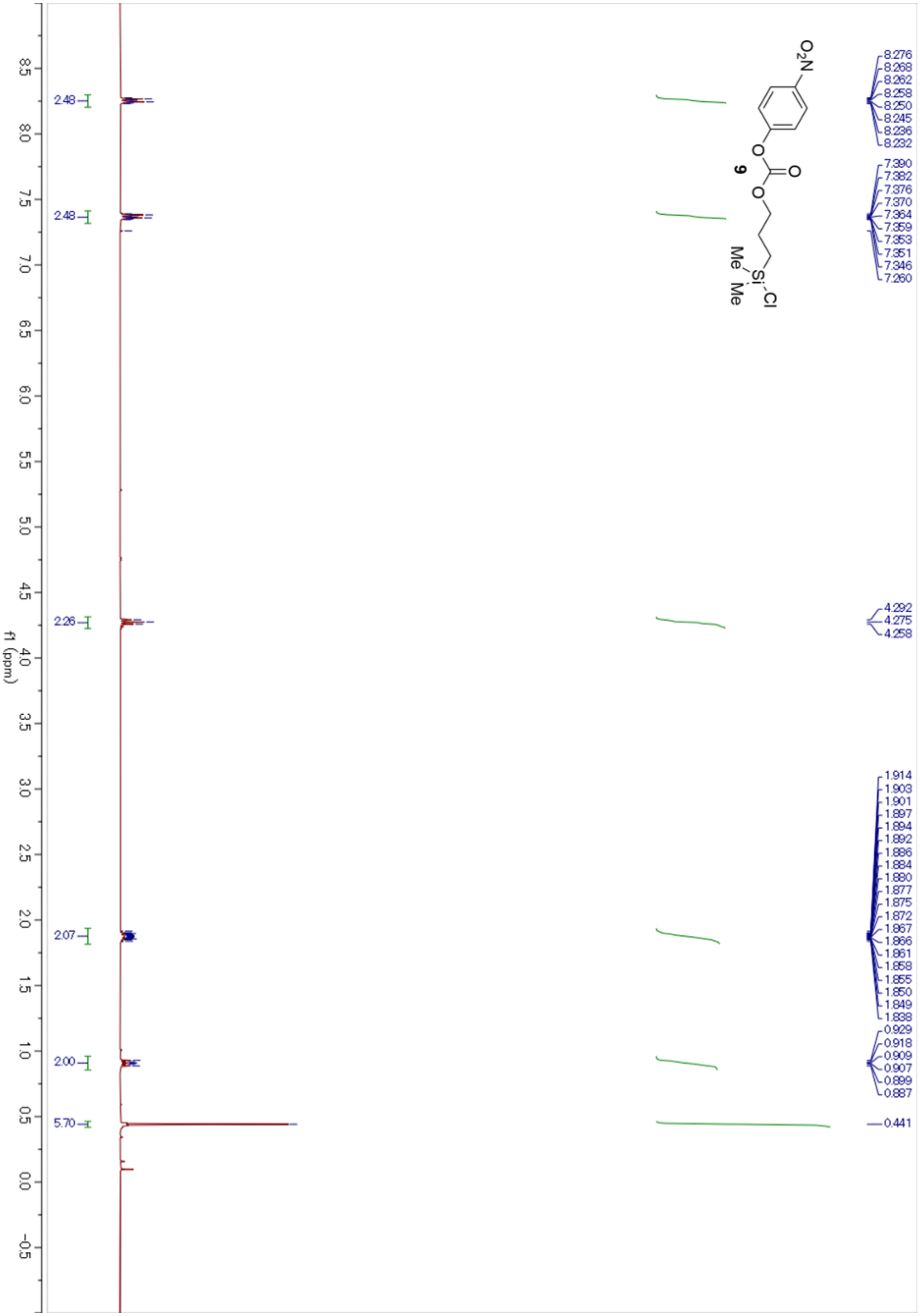
O2N
9
Si
Cl
Me
Me
8.276
8.268
8.262
8.258
8.250
8.245
8.236
8.232
7.390
7.382
7.376
7.370
7.364
7.359
7.353
7.351
7.346
7.260
4.292
4.275
4.258
1.914
1.903
1.901
1.897
1.894
1.892
1.886
1.884
1.880
1.877
1.875
1.872
1.867
1.866
1.861
1.858
1.855
1.850
1.849
1.838
0.929
0.918
0.909
0.907
0.899
0.887
0.441
2.48
2.48
2.26
2.07
2.00
5.70
f1 (ppm)

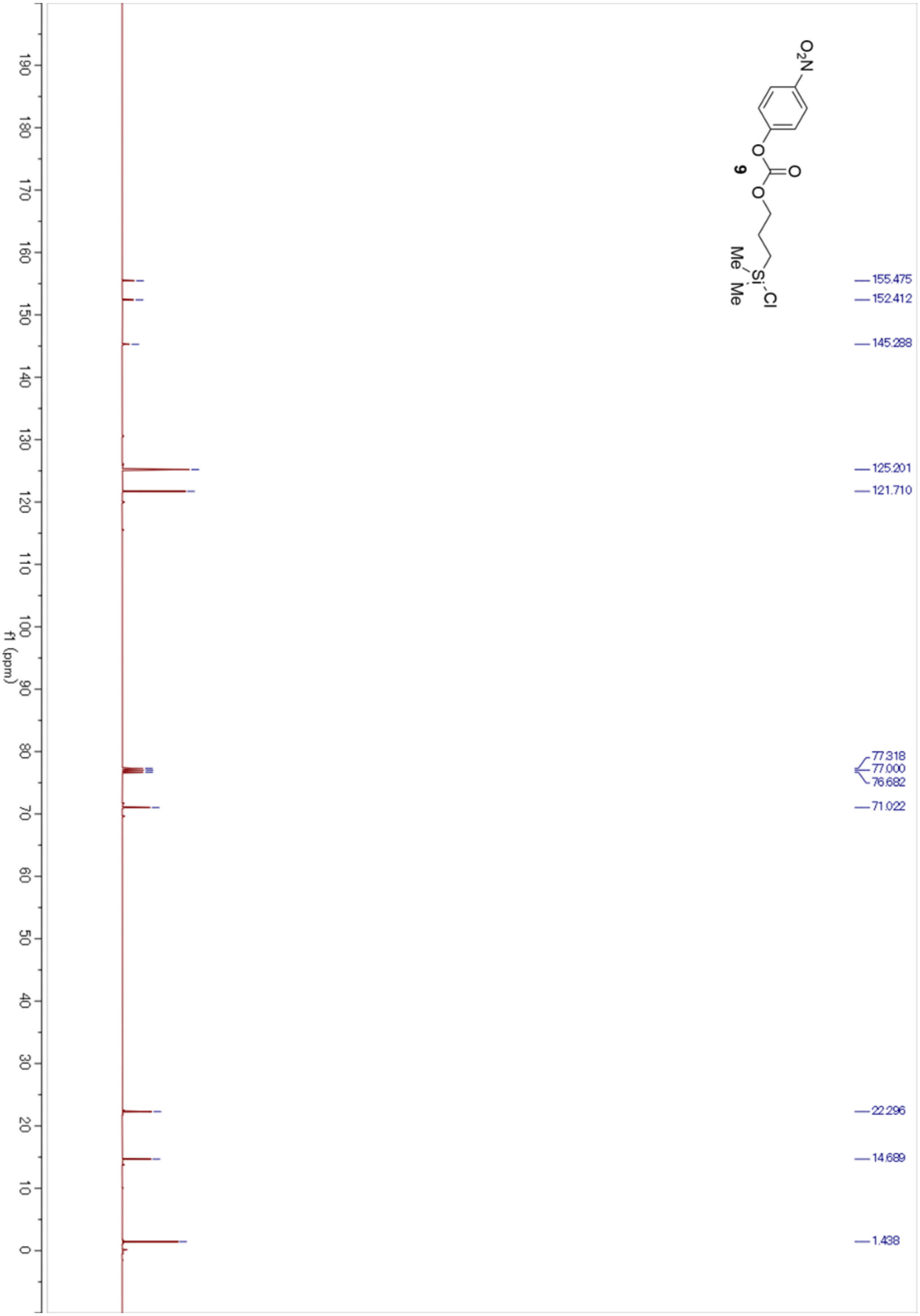

O2N
9
Me
Si
Cl
Me
155.475
152.412
145.288
125.201
121.710
77.318
77.000
76.682
71.022
22.296
14.689
1.438
f1 (ppm)
190
180
170
160
150
140
130
120
110
100
90
80
70
60
50
40
30
20
10
0

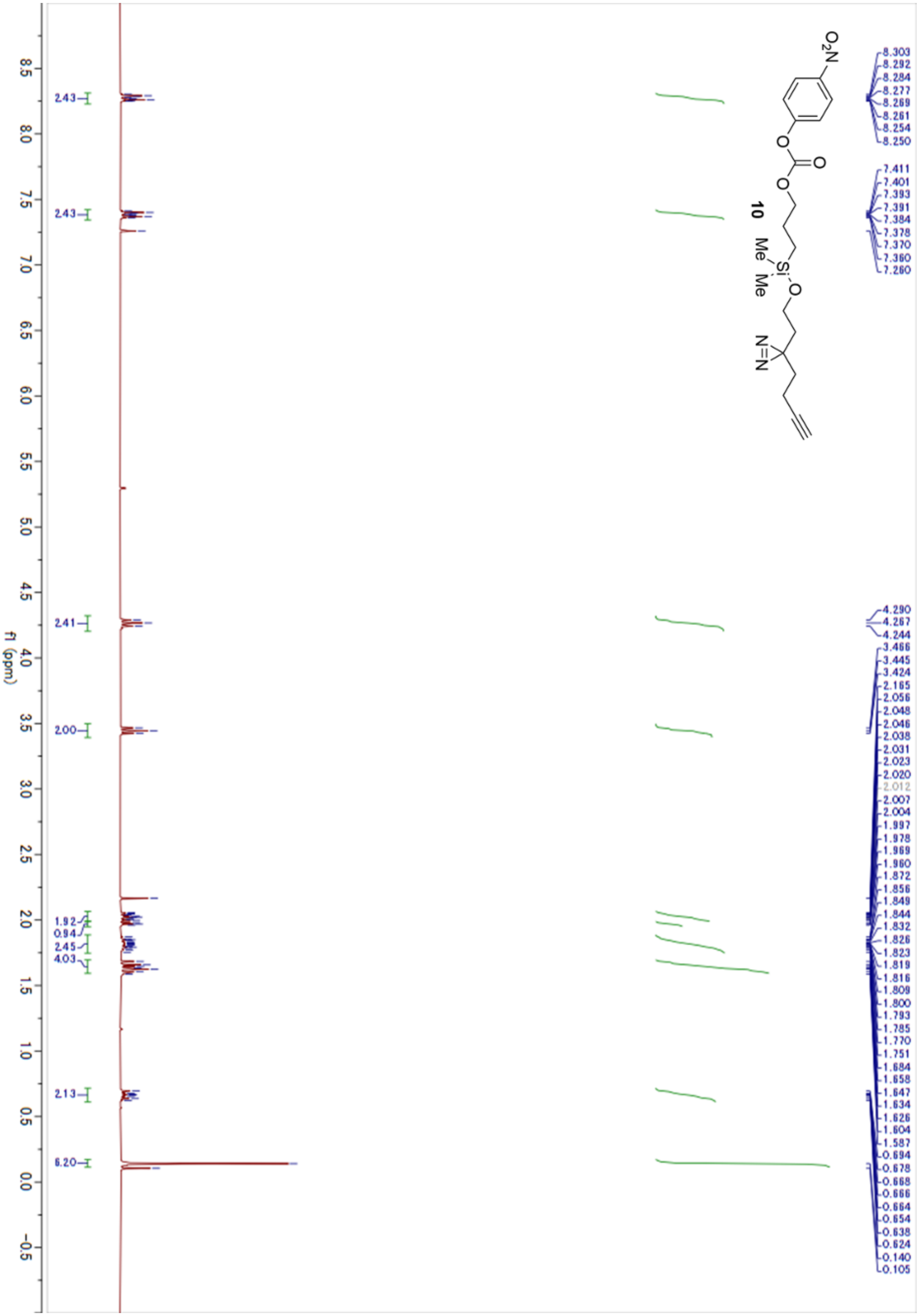
10
f1 (ppm)
8.303 8.292 8.284 8.277 8.269 8.261 8.254 8.250
7.411 7.401 7.393 7.391 7.384 7.378 7.370 7.360 7.260
4.290 4.267 4.244
3.466 3.445 3.424
2.165 2.056 2.048 2.046 2.038 2.031 2.023 2.020 2.012 2.007 2.004 1.997 1.978 1.969 1.960
1.872 1.856 1.849 1.844 1.832 1.826 1.823 1.819 1.816 1.809 1.800 1.793 1.785 1.770 1.751 1.684 1.658 1.647 1.634 1.626 1.604 1.587
0.694 0.678 0.668 0.666 0.664 0.654 0.638 0.624 0.140 0.105
2.43 2.43 2.41 2.00 1.92 0.94 2.45 4.03 2.13 6.20

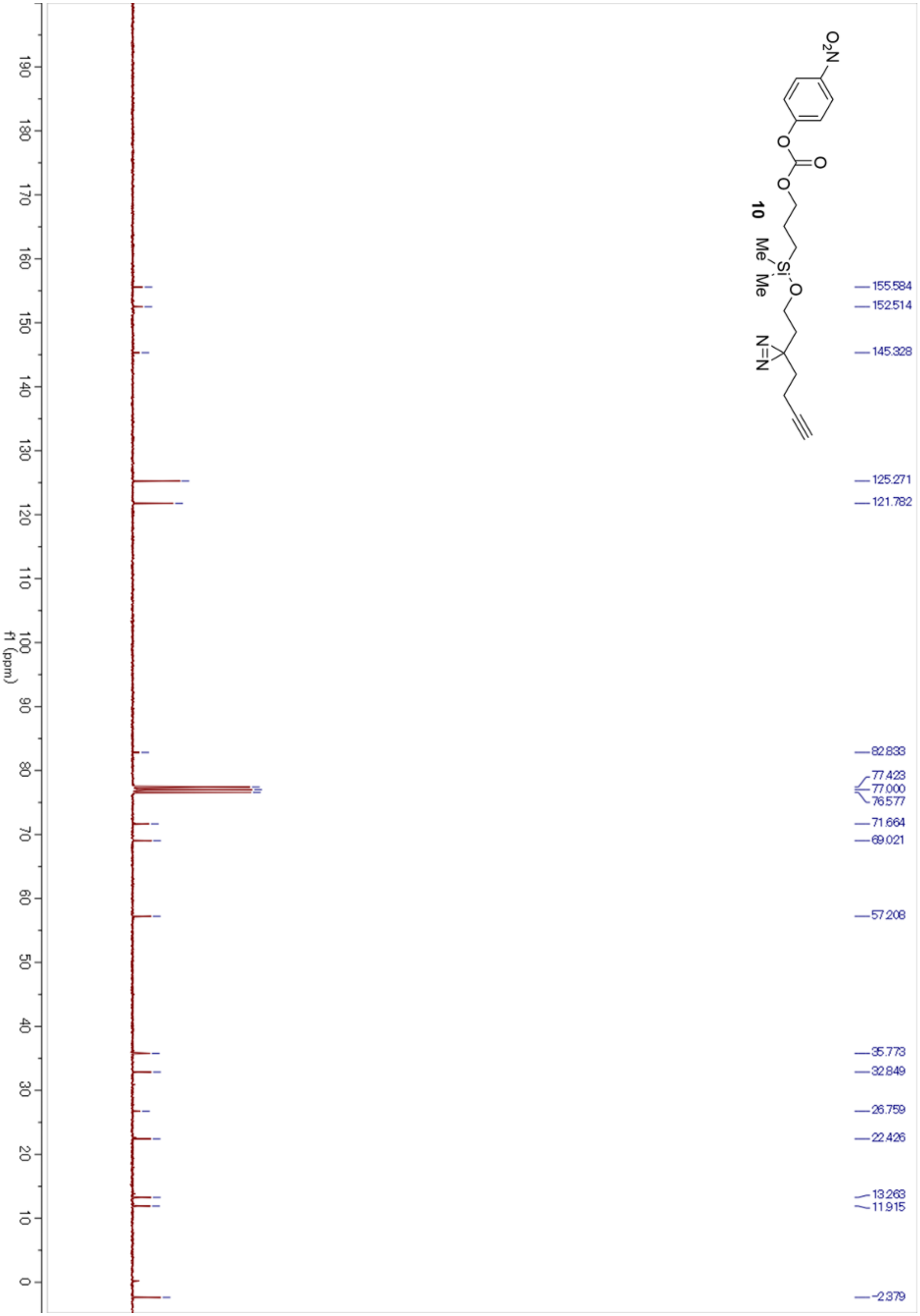
10
155.584
152.514
145.328
125.271
121.782
82.833
77.423
77.000
76.577
71.664
69.021
57.208
35.773
32.849
26.759
22.426
13.263
11.915
-2.379
f1 (ppm)

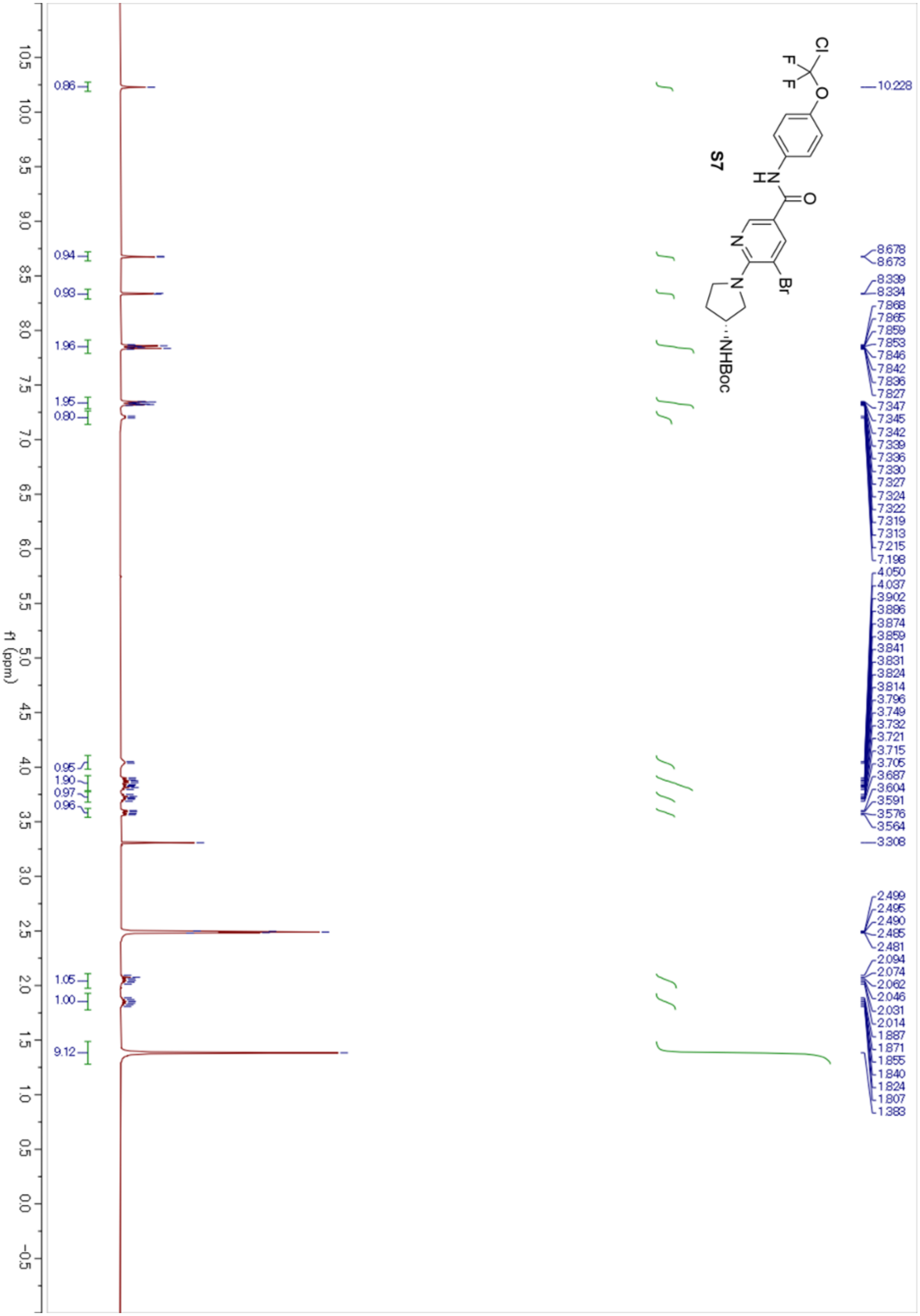
S7
NHBoc
Br
Cl
F
F
O
N
H
f1 (ppm)
10.228
8.678
8.673
8.339
8.334
7.868
7.865
7.859
7.853
7.846
7.842
7.836
7.827
7.347
7.345
7.342
7.339
7.336
7.330
7.327
7.324
7.322
7.319
7.313
7.215
7.198
4.050
4.037
3.902
3.886
3.874
3.859
3.841
3.831
3.824
3.814
3.796
3.749
3.732
3.721
3.715
3.705
3.687
3.604
3.591
3.576
3.564
3.308
2.499
2.495
2.490
2.485
2.481
2.094
2.074
2.062
2.046
2.031
2.014
1.887
1.871
1.855
1.840
1.824
1.807
1.383
0.86
0.94
0.93
1.96
1.95
0.80
0.95
1.90
0.97
0.96
1.05
1.00
9.12

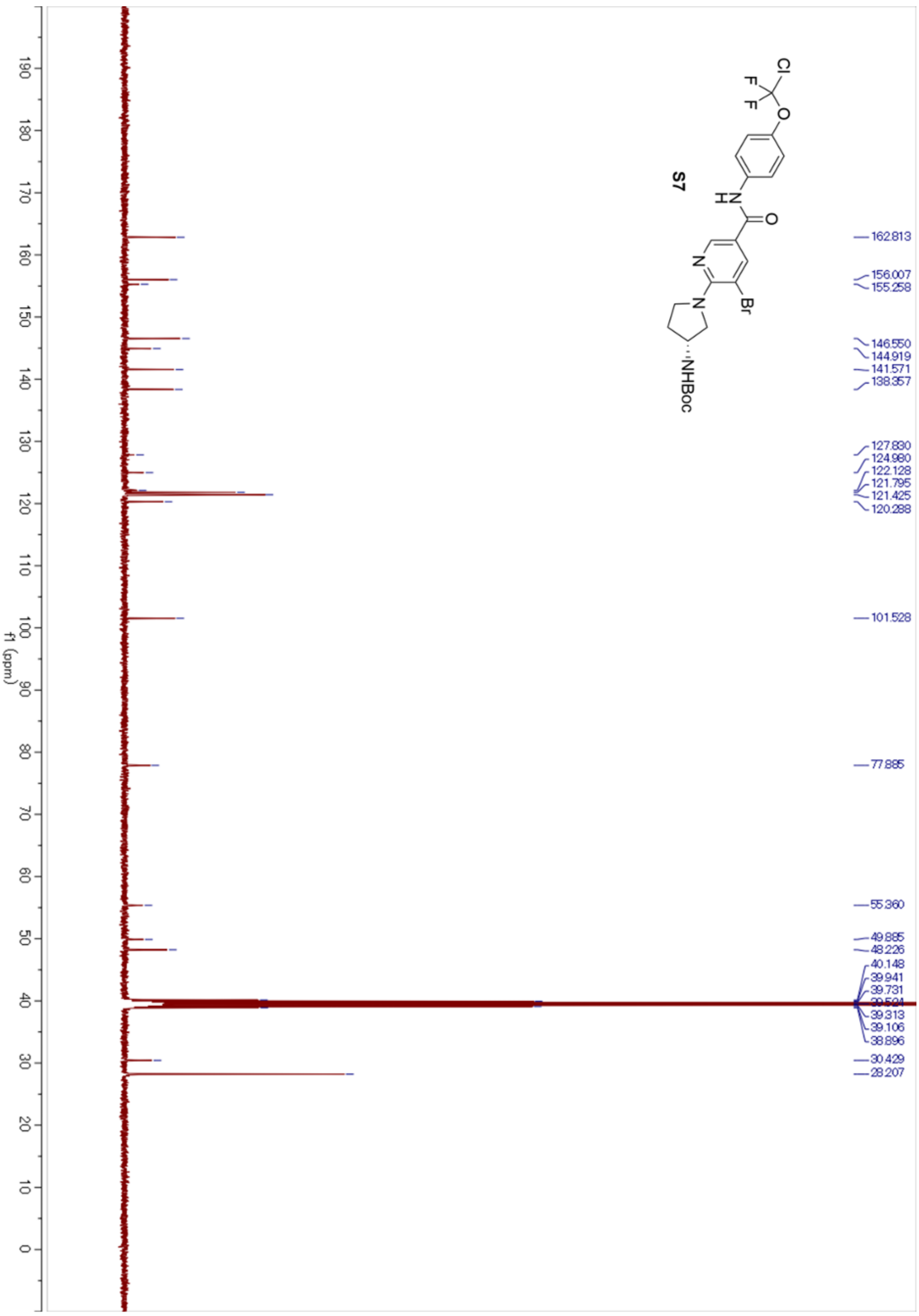

S7
Cl
F
F
O
N
H
O
N
N
Br
NHBoc
162.813
156.007
155.258
146.550
144.919
141.571
138.357
127.830
124.980
122.128
121.795
121.425
120.288
101.528
77.885
55.360
49.885
48.226
40.148
39.941
39.731
39.313
39.106
38.896
30.429
28.207
f1 (ppm)

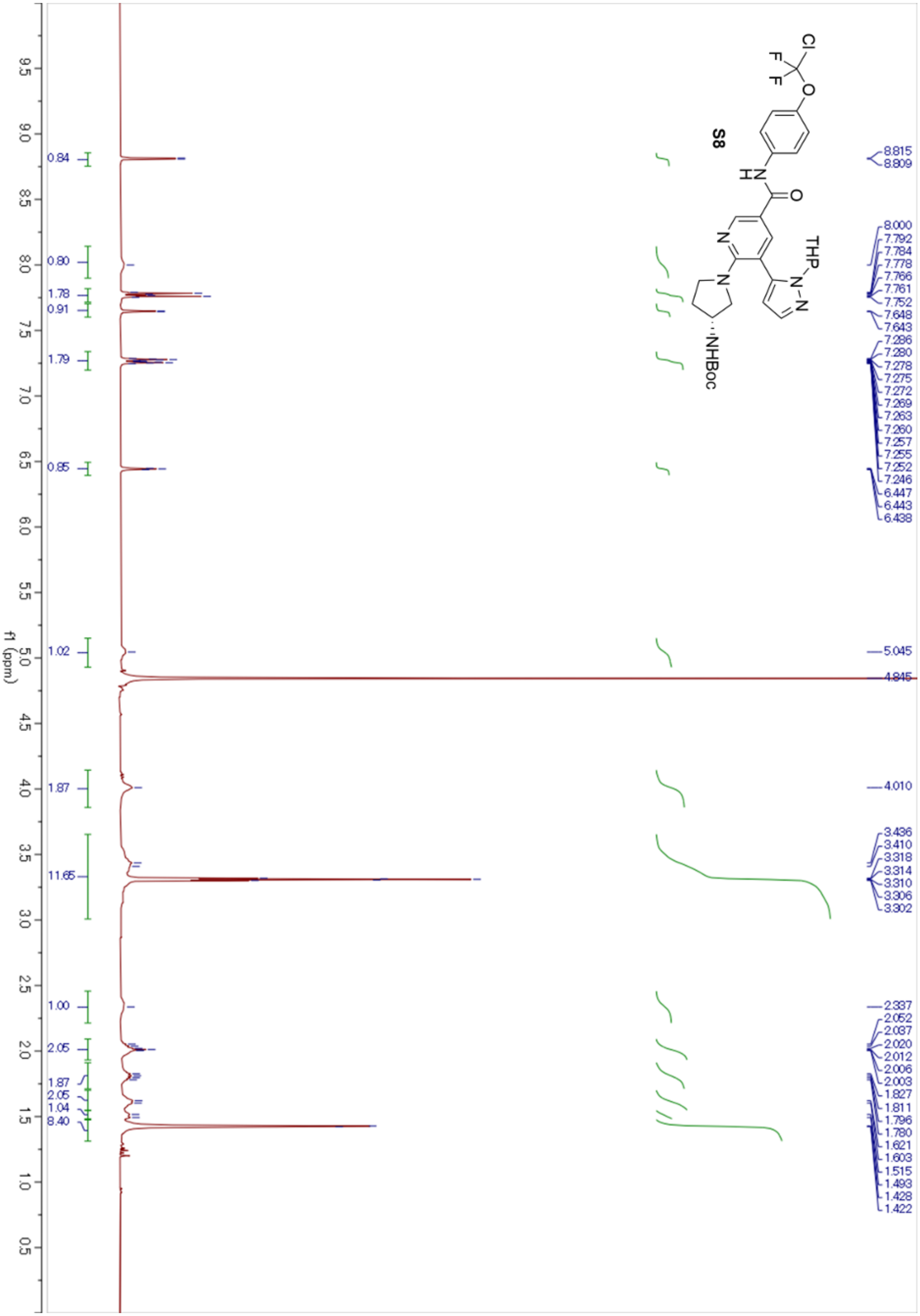

S8
THP
NHBoc
8.815
8.809
8.000
7.792
7.784
7.778
7.766
7.761
7.752
7.648
7.643
7.286
7.280
7.278
7.275
7.272
7.269
7.263
7.260
7.257
7.255
7.252
7.246
6.447
6.443
6.438
5.045
4.845
4.010
3.436
3.410
3.318
3.314
3.310
3.306
3.302
2.337
2.052
2.037
2.020
2.012
2.006
2.003
1.827
1.811
1.796
1.780
1.621
1.603
1.515
1.493
1.428
1.422
0.84
0.80
1.78
0.91
1.79
0.85
1.02
1.87
11.65
1.00
2.05
1.87
2.05
1.04
8.40
f1 (ppm)

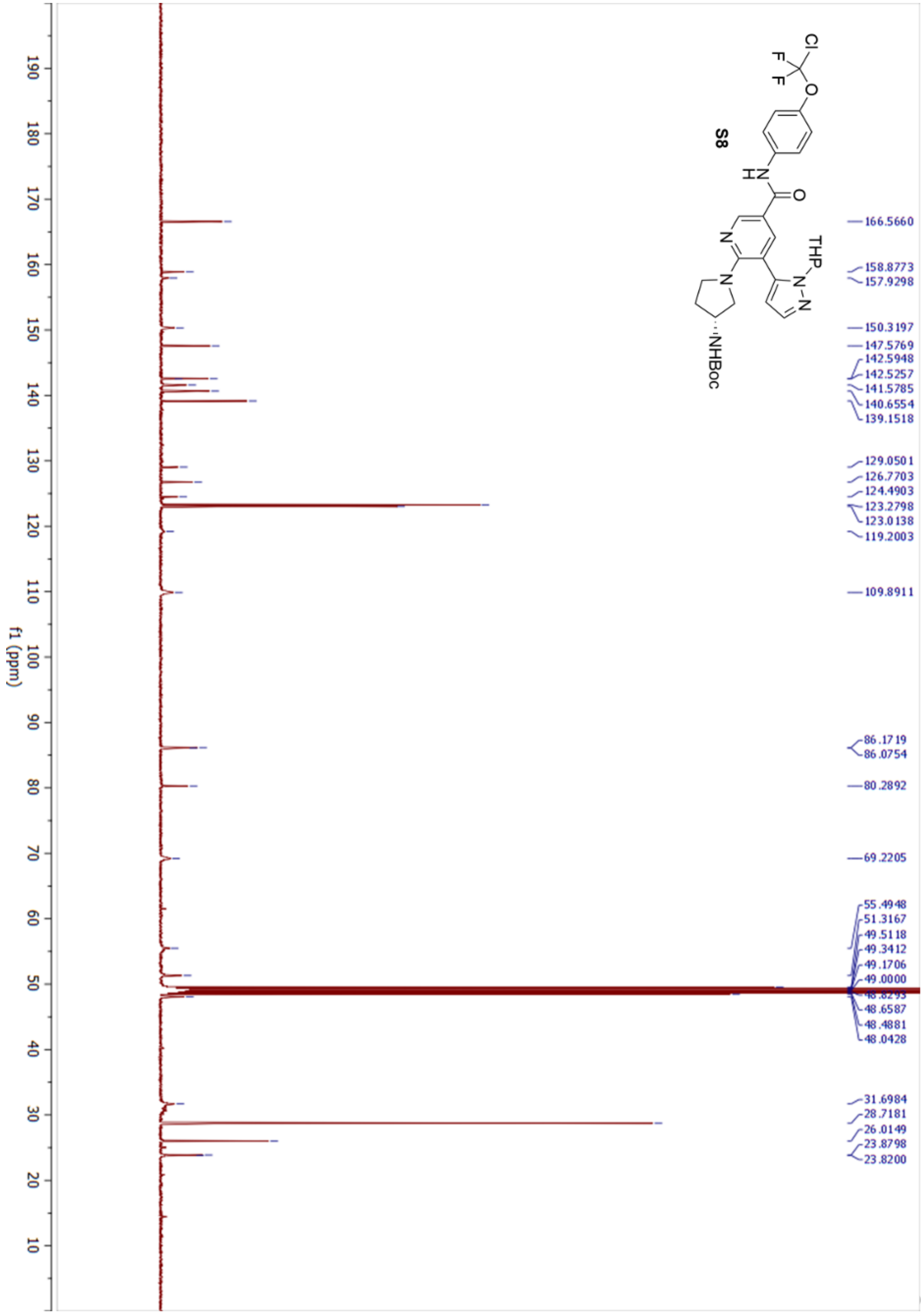
S8
THP
NHBoc
166.5660
158.8773
157.9298
150.3197
147.5769
142.5948
142.5257
141.5785
140.6554
139.1518
129.0501
126.7703
124.4903
123.2798
123.0138
119.2003
109.8911
86.1719
86.0754
80.2892
69.2205
55.4948
51.3167
49.5118
49.3412
49.1706
49.0000
48.6587
48.4881
48.0428
31.6984
28.7181
26.0149
23.8798
23.8200
f1 (ppm)

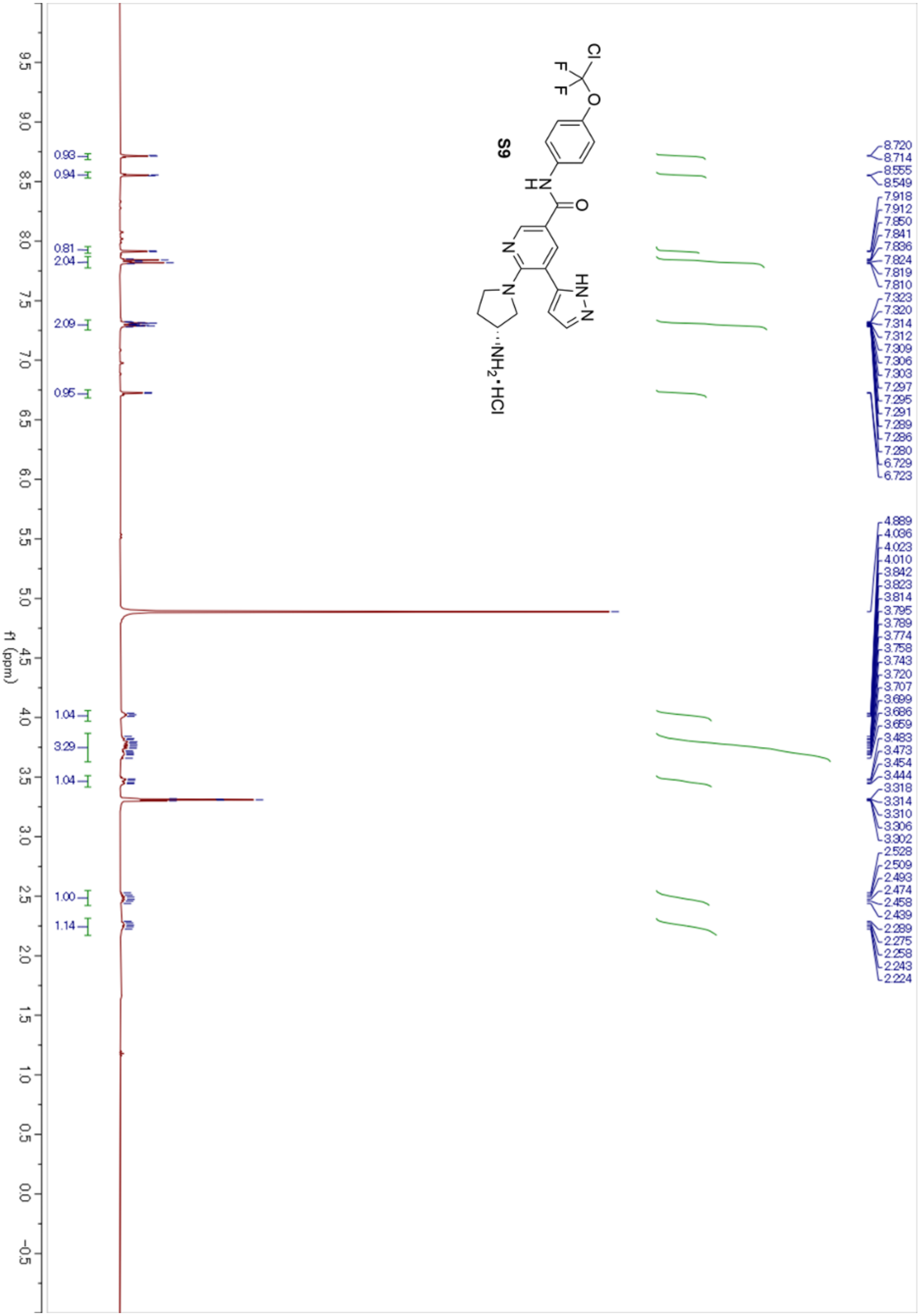
S9
f1 (ppm)

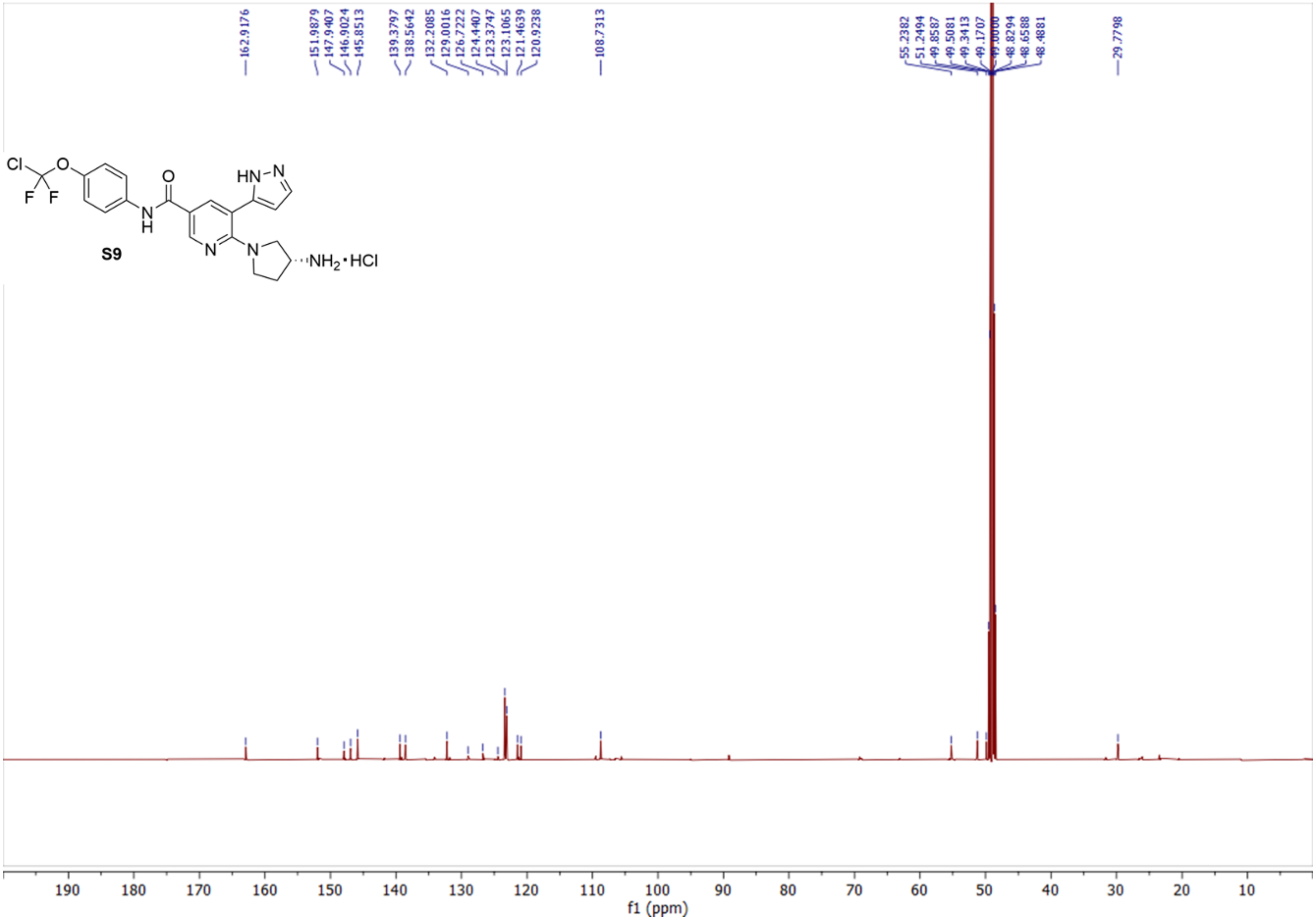
162.9176
151.9879
147.9407
146.9024
145.8513
139.3797
138.5642
132.2085
129.0016
126.7222
124.4407
123.3747
123.1065
121.4639
120.9238
108.7313
55.2382
51.2494
49.8587
49.5081
49.3413
49.1707
49.0000
48.8294
48.6588
48.4881
29.7798
S9
f1 (ppm)

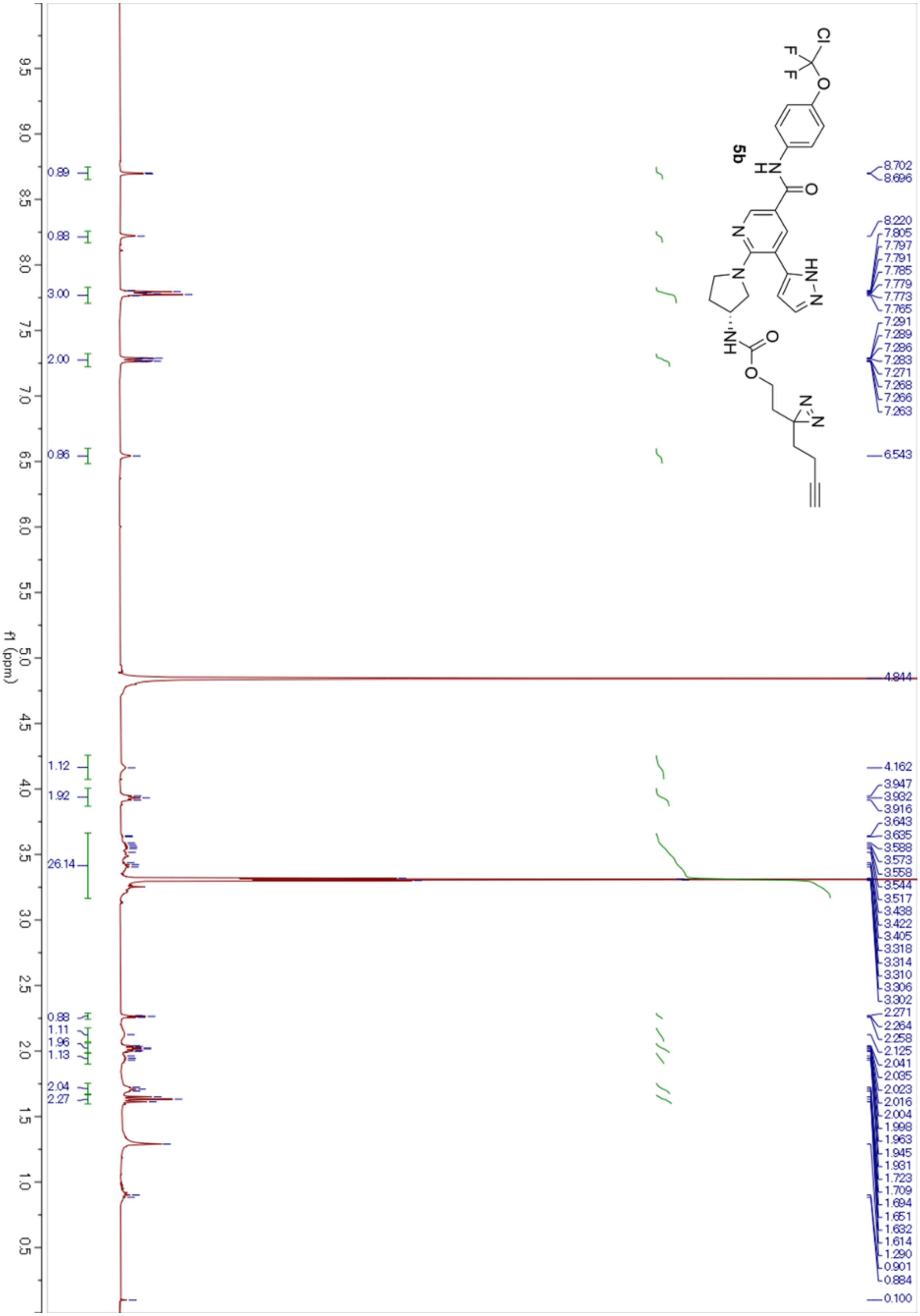

5b
8.702
8.696
8.220
7.805
7.797
7.791
7.785
7.779
7.773
7.765
7.291
7.289
7.286
7.283
7.271
7.268
7.266
7.263
6.543
4.844
4.162
3.947
3.932
3.916
3.643
3.635
3.588
3.573
3.558
3.544
3.517
3.438
3.422
3.405
3.318
3.314
3.310
3.306
3.302
2.271
2.264
2.258
2.125
2.041
2.035
2.023
2.016
2.004
1.998
1.963
1.945
1.931
1.723
1.709
1.694
1.651
1.632
1.614
1.290
0.901
0.884
0.100
0.89
0.88
3.00
2.00
0.86
1.12
1.92
26.14
0.88
1.11
1.96
1.13
2.04
2.27
f1 (ppm)

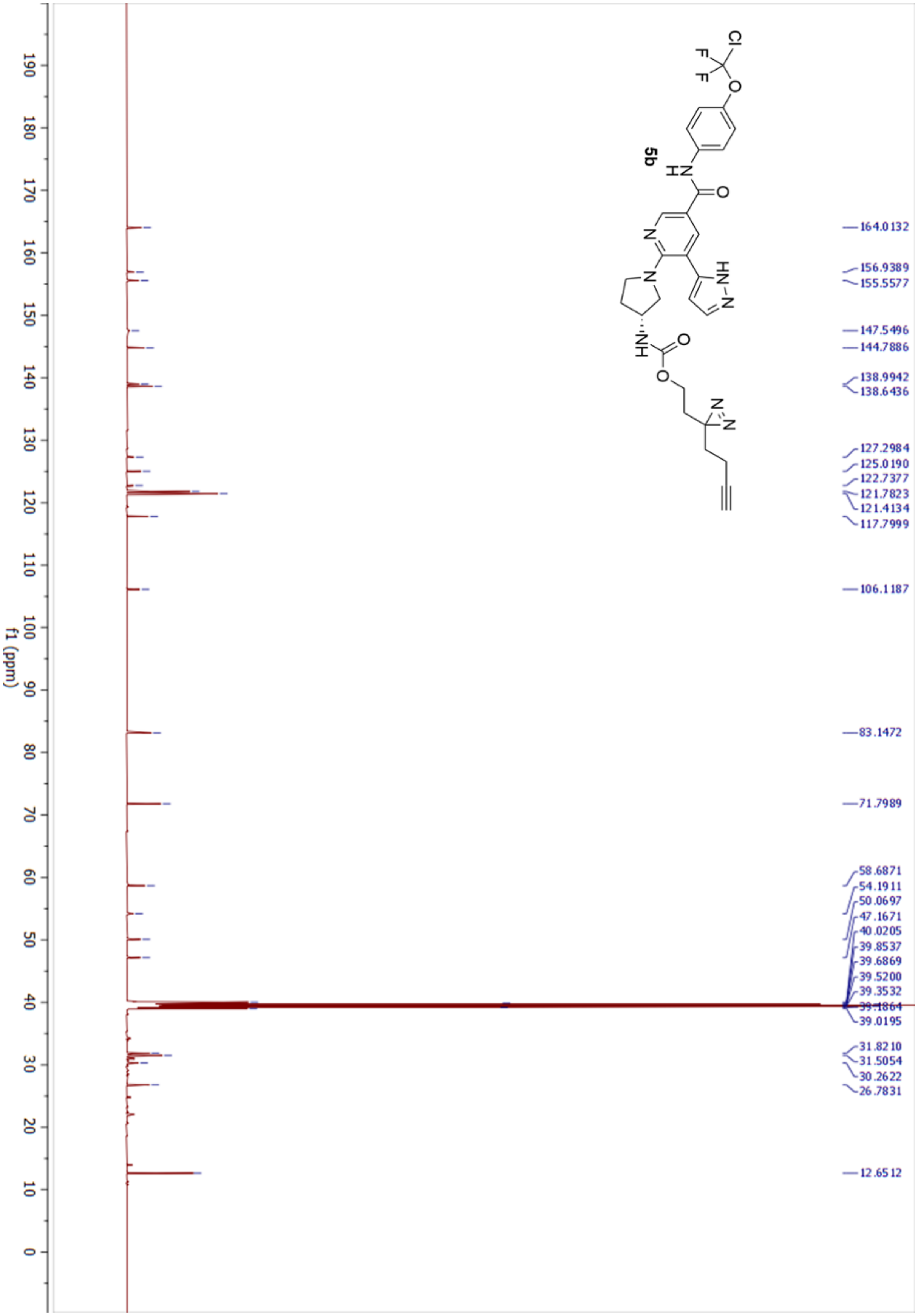
5b
f1 (ppm)
164.0132
156.9389
155.5577
147.5496
144.7886
138.9942
138.6436
127.2984
125.0190
122.7377
121.7823
121.4134
117.7999
106.1187
83.1472
71.7989
58.6871
54.1911
50.0697
47.1671
40.0205
39.8537
39.6869
39.5200
39.3532
39.1864
39.0195
31.8210
31.5054
30.2622
26.7831
12.6512

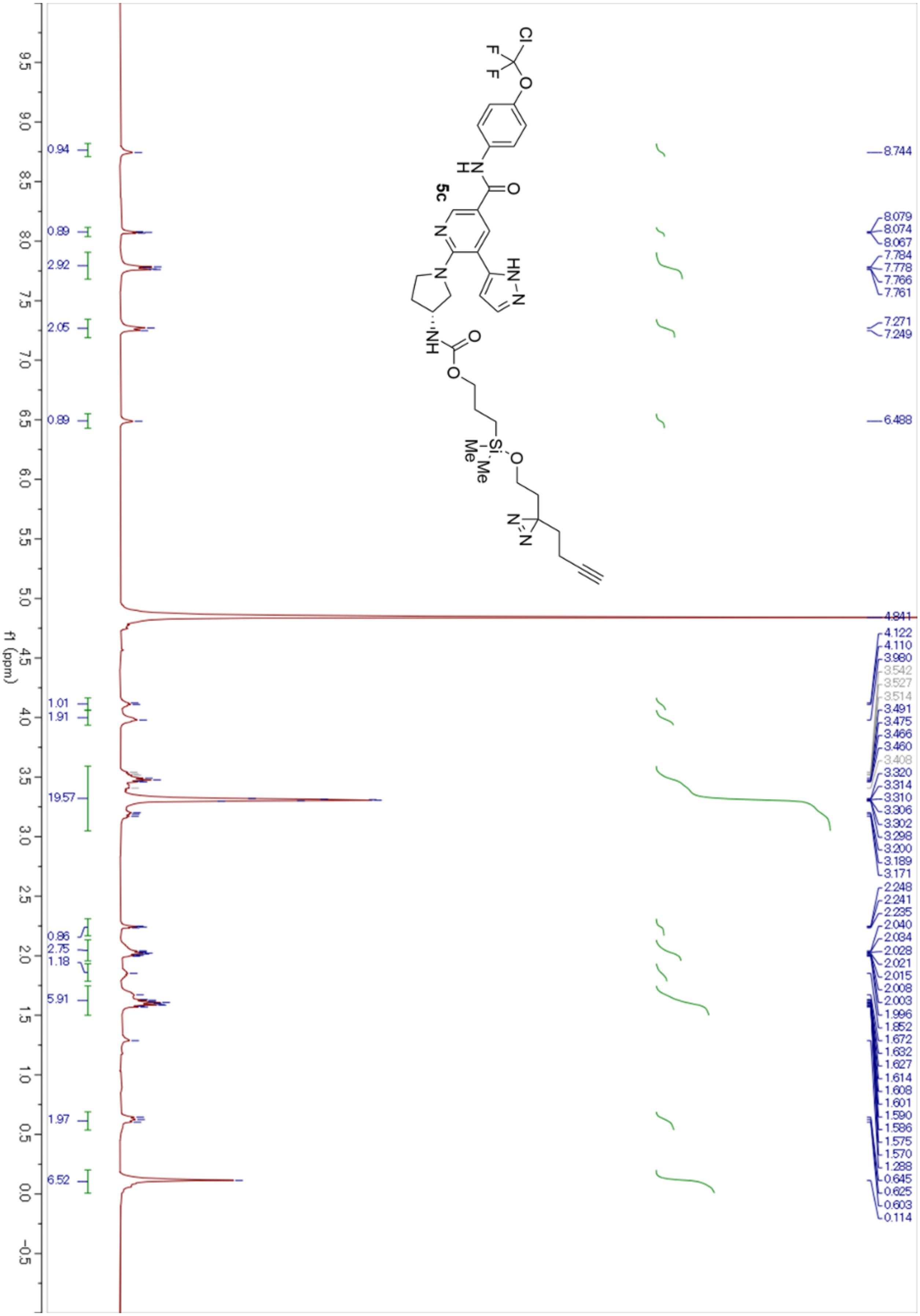
5c
f1 (ppm)

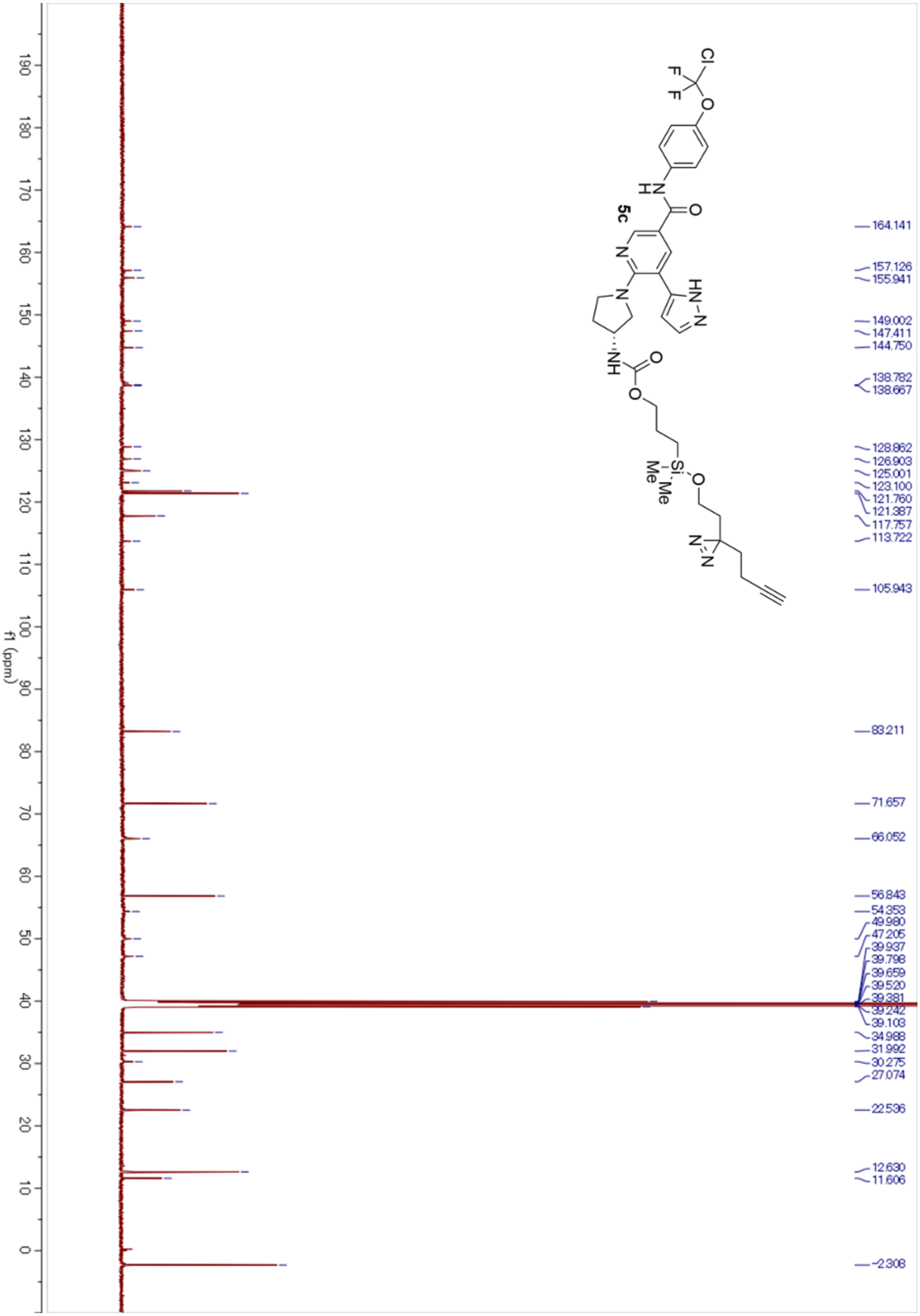
5c
164.141
157.126
155.941
149.002
147.411
144.750
138.782
138.667
128.862
126.903
125.001
123.100
121.760
121.387
117.757
113.722
105.943
83.211
71.657
66.052
56.843
54.353
49.980
47.205
39.937
39.798
39.659
39.520
39.381
39.242
39.103
34.988
31.992
30.275
27.074
22.536
12.630
11.606
-2.308
190
180
170
160
150
140
130
120
110
100
90
80
70
60
50
40
30
20
10
0
f1 (ppm)

(G) Supplementary References

1. Yang, K. L. *et al.* MSBooster: improving peptide identification rates using deep learning-based features. *Nat. Commun.* **14**, 4539 (2023).

2. Li, K., Vaudel, M., Zhang, B., Ren, Y. & Wen, B. PDV: an integrative proteomics data viewer. *Bioinformatics* **35**, 1249–1251 (2019).

3. Keller, A., Nesvizhskii, A. I., Kolker, E. & Aebersold, R. Empirical statistical model to estimate the accuracy of peptide identifications made by MS/MS and database search. *Anal. Chem.* **74**, 5383–5392 (2002).

4. Sievers, F. *et al.* Fast, scalable generation of high-quality protein multiple sequence alignments using Clustal Omega. *Mol. Syst. Biol.* **7**, 539 (2011).

5. Karaman, M. W. *et al.* A quantitative analysis of kinase inhibitor selectivity. *Nat. Biotechnol.* **26**, 127–132 (2008).

6. Zhao, Q. *et al.* Broad-Spectrum Kinase Profiling in Live Cells with Lysine-Targeted Sulfonyl Fluoride Probes. *J. Am. Chem. Soc.* **139**, 680–685 (2017).

7. Rix, U. *et al.* Chemical proteomic profiles of the BCR-ABL inhibitors imatinib, nilotinib, and dasatinib reveal novel kinase and nonkinase targets. *Blood* **110**, 4055–4063 (2007).

8. Martin, B. R., Giepmans, B. N. G., Adams, S. R. & Tsien, R. Y. Mammalian cell-based optimization of the biarsenical-binding tetracysteine motif for improved fluorescence and affinity. *Nat. Biotechnol.* **23**, 1308–1314 (2005).

9. Li, Z. *et al.* Design and synthesis of minimalist terminal alkyne-containing diazirine photo-crosslinkers and their incorporation into kinase inhibitors for cell- and tissue-based proteome profiling. *Angew. Chem. Int. Ed* **52**, 8551–8556 (2013).

10. Richardson, P. L. *et al.* Controlling cellular distribution of drugs with permeability modifying moieties. *Medchemcomm* **10**, 974–984 (2019).

11. Stepek, I. A. *et al.* Antibiotic Discovery with Synthetic Fermentation: Library Assembly, Phenotypic Screening, and Mechanism of Action of β-Peptides Targeting Penicillin-Binding Proteins. *ACS Chem. Biol.* **14**, 1030–1040 (2019).

12. Gao, J., Mfuh, A., Amako, Y. & Woo, C. M. Small molecule interactome mapping by photoaffinity labeling reveals binding site hotspots for the nsaids. *J. Am. Chem. Soc.* **140**, 4259–4268 (2018).

13. Wylie, A. A. *et al.* The allosteric inhibitor ABL001 enables dual targeting of BCR-ABL1. *Nature* **543**, 733–737 (2017).

(H) Source Data for Supplemental Figures

Supplemental Figure 4A

In-gel fluorescence

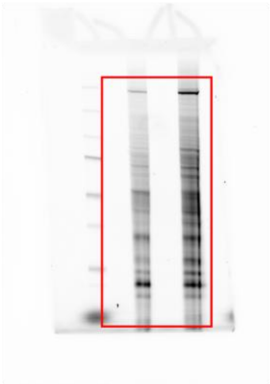

Coomassie

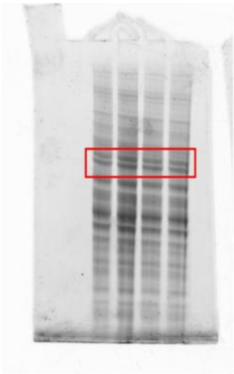

Supplemental Figure 4B

In-gel fluorescence

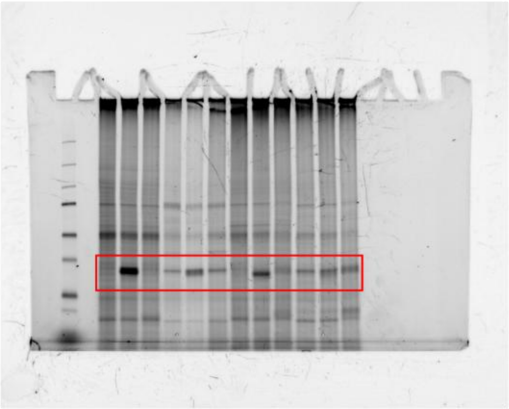

Coomassie

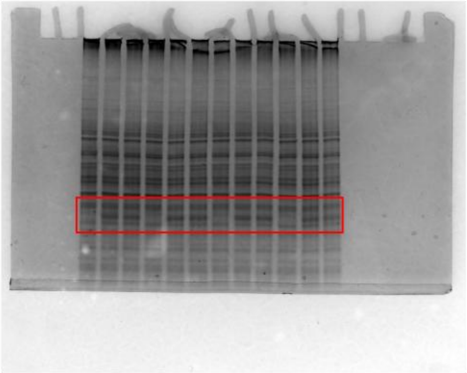

Supplemental Figure 4C

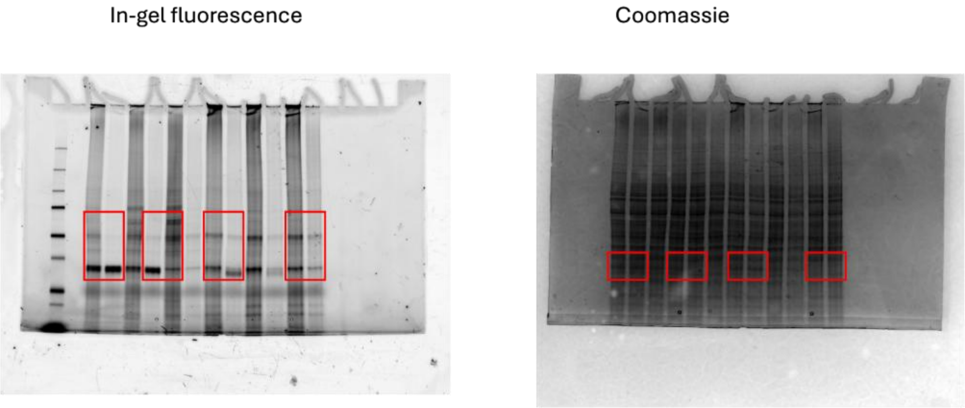

Supplemental Figure 7A

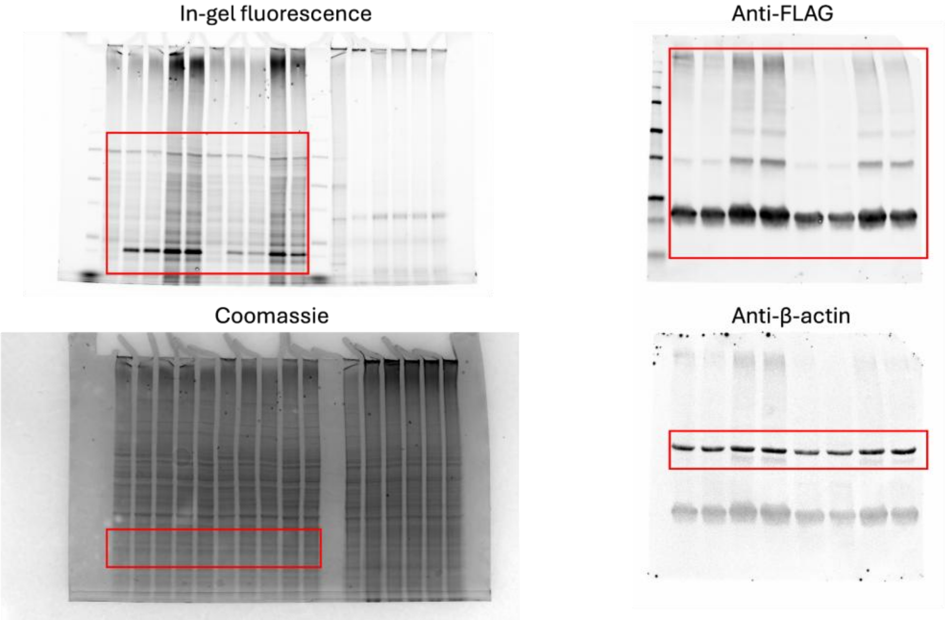

Supplemental Figure 7D

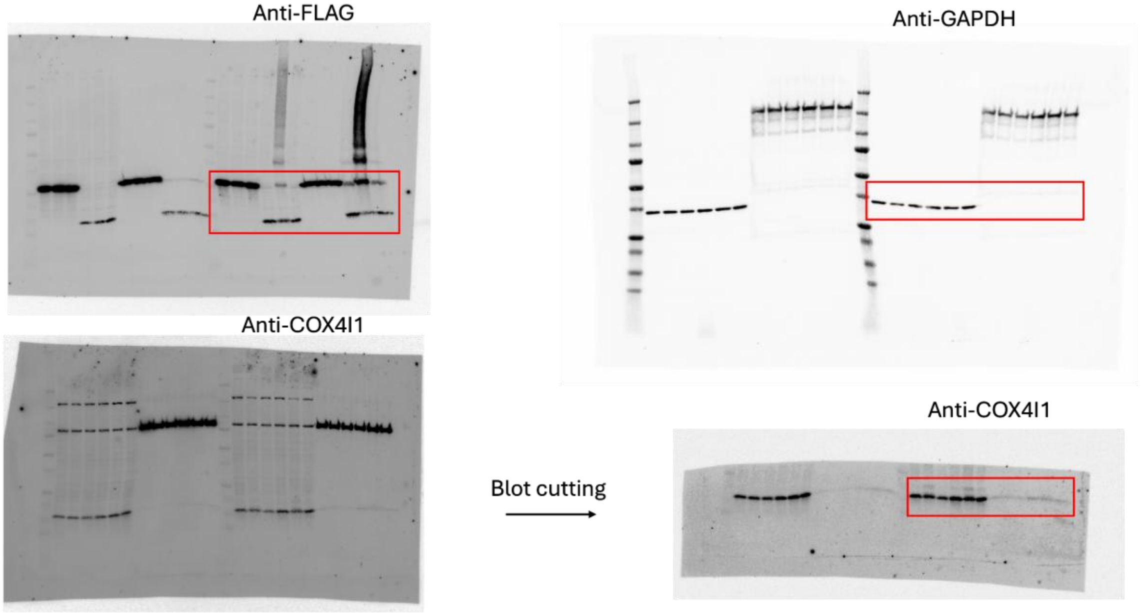

Supplemental Figure 7E

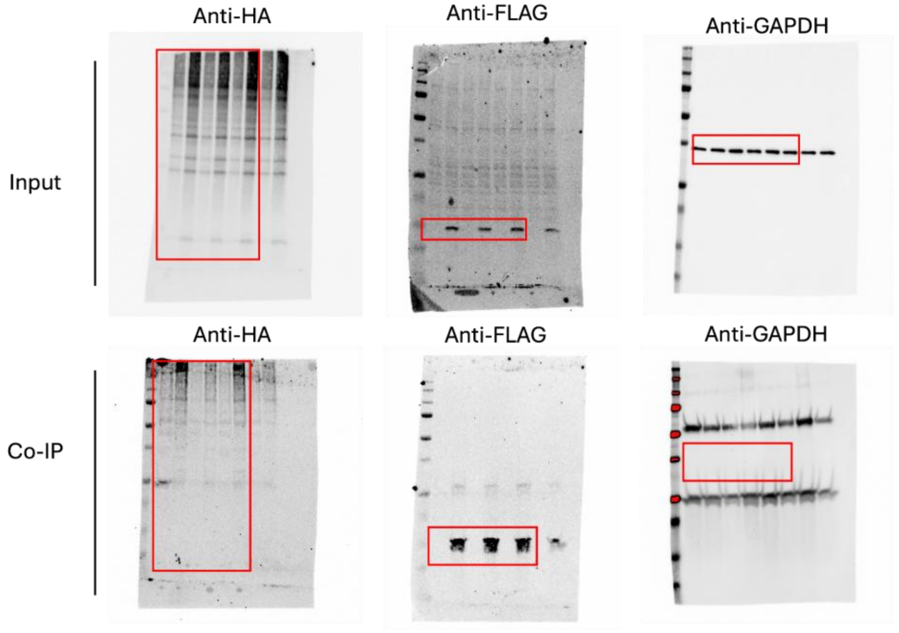

Supplemental Figure 7F

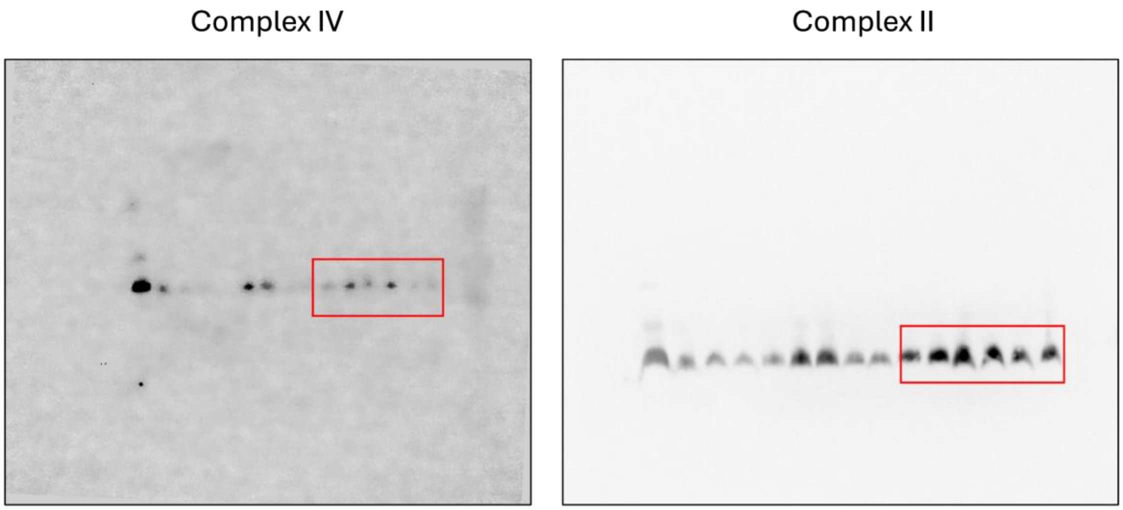

Supplement: Supplementary file 1 — Supplementary Notes 1–8, Supplementary Figures 1–7, Supplementary Schemes 1–4, synthetic procedures, nuclear magnetic resonance spectra and Supplementary Tables 1, 3, 8 and 11. [file 41557_2026_2127_MOESM1_ESM.pdf]
