# Supplementary material for: Small-molecule binding-site discovery using silyl ether-enabled chemoproteomics
Source: Nat Chem. 2026 Apr 27;18(8):1431–42. doi: 10.1038/s41557-026-02127-4 (PMC13423832; doi:10.1038/s41557-026-02127-4)
Supplement: Supplementary file 2 — Reporting Summary [file 41557_2026_2127_MOESM2_ESM.pdf]

Corresponding author(s): Backus

Last updated by author(s): Jan 19, 2026

Reporting Summary

Nature Portfolio wishes to improve the reproducibility of the work that we publish. This form provides structure for consistency and transparency in reporting. For further information on Nature Portfolio policies, see our Editorial Policies and the Editorial Policy Checklist.

Statistics

For all statistical analyses, confirm that the following items are present in the figure legend, table legend, main text, or Methods section.

n/a	Confirmed	
☐	☒	The exact sample size (*n*) for each experimental group/condition, given as a discrete number and unit of measurement
☐	☒	A statement on whether measurements were taken from distinct samples or whether the same sample was measured repeatedly
☐	☒	The statistical test(s) used AND whether they are one- or two-sided<br>*Only common tests should be described solely by name; describe more complex techniques in the Methods section.*
☒	☐	A description of all covariates tested
☐	☒	A description of any assumptions or corrections, such as tests of normality and adjustment for multiple comparisons
☐	☒	A full description of the statistical parameters including central tendency (e.g. means) or other basic estimates (e.g. regression coefficient) AND variation (e.g. standard deviation) or associated estimates of uncertainty (e.g. confidence intervals)
☐	☒	For null hypothesis testing, the test statistic (e.g. F, t, r) with confidence intervals, effect sizes, degrees of freedom and P value noted<br>*Give P values as exact values whenever suitable.*
☒	☐	For Bayesian analysis, information on the choice of priors and Markov chain Monte Carlo settings
☒	☐	For hierarchical and complex designs, identification of the appropriate level for tests and full reporting of outcomes
☒	☐	Estimates of effect sizes (e.g. Cohen's *d*, Pearson's *r*), indicating how they were calculated

Our web collection on statistics for biologists contains articles on many of the points above.

Software and code

Policy information about availability of computer code

Data collection	LC-MS/MS proteomic data were collected with an Easy=nLC1200 attached to a Thermo Scientific™ Orbitrap Eclipse™ Tribrid™ mass spectrometer using the Xcalibur (v4.6.67.17) software.<br>Immunoblots and fluorescent gels were imaged using a ChemiDocTM MP Imaging System (Bio-Rad #12003153) running Image Lab Touch.<br>For enzymatic activity assay, data were acquired with a Synergy H1 (BioTek) plate reader running Gen5 v3.04 software.
Data analysis	Raw data collected by LC-MS/MS were searched with MSFragger (v3.8 and v4.1) and FragPipe (v20.0, v21.2-build38 and v22.0) for all data under dataset identifiers PXD068136, PXD068139, PXD068140, and PXD070048.<br>Data for volcano plots was analyzed using R-studio: Version 2024.09.0+375 (2024.09.0+375).<br>Data with bar, violin and waterfall plots and statistics were analyzed using GraphPad Prism: Version 10.4.1 (532).<br>FragPipe is available on GitHub at https://github.com/Nesvilab/FragPipe. Code Availability: The scripts used for this work are available at https://github.com/BackusLab.<br>Depictions of protein structures obtained directly from PDB were generated using licensed PyMOL Molecular Graphics System, version 2.5.5.<br>Docking study was done using AutoDockTools (version 1.5.7) and ChimeraX 1.8 with binding energies calculated using Lamarckian Genetic Algorithm 6.0.16 and empirical free-energy scoring function in Autodock 4.2

For manuscripts utilizing custom algorithms or software that are central to the research but not yet described in published literature, software must be made available to editors and reviewers. We strongly encourage code deposition in a community repository (e.g. GitHub). See the Nature Portfolio guidelines for submitting code & software for further information.

Data

Policy information about availability of data

All manuscripts must include a data availability statement. This statement should provide the following information, where applicable:

- Accession codes, unique identifiers, or web links for publicly available datasets
- A description of any restrictions on data availability
- For clinical datasets or third party data, please ensure that the statement adheres to our policy

The MS data have been deposited to the ProteomeXchange Consortium (http://proteomecentral.proteomexchange.org) via the Proteomics Identification Database (PRIDE) partner repository with the dataset identifiers: PXD068136, PXD068139, PXD068140, and PXD070048. Raw data for Figures and Extended Figures are available in the accompanying Source Data Files as well as Supplementary Tables. All other data is available in the main text or the Supplemental Information. Publicly available database for LC-MS/MS analysis is obtained from the UniProt Consortium (https://www.uniprot.org/).

Human research participants

Policy information about studies involving human research participants and Sex and Gender in Research.

Reporting on sex and gender	N/A
Population characteristics	N/A
Recruitment	N/A
Ethics oversight	N/A

Note that full information on the approval of the study protocol must also be provided in the manuscript.

Field-specific reporting

Please select the one below that is the best fit for your research. If you are not sure, read the appropriate sections before making your selection.

☒ Life sciences ☐ Behavioural & social sciences ☐ Ecological, evolutionary & environmental sciences

For a reference copy of the document with all sections, see nature.com/documents/nr-reporting-summary-flat.pdf

Life sciences study design

All studies must disclose on these points even when the disclosure is negative.

Sample size	All proteomic experiments were performed with at least 2-3 biological replicates.
Data exclusions	No data were excluded from the analyses.
Replication	All attempts at replication were successful. All experiments were performed in biological/technical replicates stated in in figure legends, method sections and main text.
Randomization	Plates of cells were randomized to control/treatment groups.
Blinding	Blinding of the researcher was not relevant as data were collected with constant acquisition parameter.

Reporting for specific materials, systems and methods

We require information from authors about some types of materials, experimental systems and methods used in many studies. Here, indicate whether each material, system or method listed is relevant to your study. If you are not sure if a list item applies to your research, read the appropriate section before selecting a response.

Materials & experimental systems

n/a	Involved in the study
☐	☒ Antibodies
☐	☒ Eukaryotic cell lines
☒	☐ Palaeontology and archaeology
☒	☐ Animals and other organisms
☒	☐ Clinical data
☒	☐ Dual use research of concern

Methods

n/a	Involved in the study
☒	☐ ChIP-seq
☒	☐ Flow cytometry
☒	☐ MRI-based neuroimaging

Antibodies

Antibodies used

All primary antibodies in the study, used at 1:3000 dilution, include: DYKDDDDK (FLAG) (Cell Signaling Technology, #14793, #7), c-Abl (Cell Signaling, #2862S, #16), phospho-c-Abl (Y245) (Cell Signaling, #2861S, #9), STAT5 (D2O6Y) (Cell Signaling, #94205, #5), phospho-STAT5A (Y694) (ABclonal, #AP0758, #4000000176), CRKL (ABclonal, #A11735. #0030740301), phospho-CRKL (Y207) (ABclonal, #AP0824, #21156250301), COXIV (Proteintech, #11242-1-AP, #00163993 and #00110030), PMPCB (Proteintech, #16064-1-AP, #00040152), β-Actin (8H10D10) (Cell Signaling, #3700S, #21), GAPDH (Proteintech, #60004-1-Ig, #10080731) and SDHA (Invitrogen, #459200, #YB3840708). All secondary antibodies, used at 1:5000 dilution, include IRDye® 800CW Goat anti-Rabbit Secondary Antibody (Li-Cor Biotechnology, 926-32211, #D50528-07)IRDye® 800CW Goat anti-Rabbit Secondary Antibody (Li-Cor Biotechnology, 926-32211).

Validation

Antibodies were validated by their respective manufacturers and can be found at the following links:

DYKDDDDK (FLAG) (Cell Signaling Technology, #14793, #7) https://www.cellsignal.com/products/primary-antibodies/dykddddk-tag-d6w5b-rabbit-monoclonal-antibody-binds-to-same-epitope-as-sigma-aldrich-anti-flag-m2-antibody/14793?srsltid=AfmBOooT-fai2e1Eq1kFnCh4yl_DXRtbkHDcS1-tm4pH2MPq3vKGpBeN

c-Abl (Cell Signaling, #2862S, #16) https://www.cellsignal.com/products/primary-antibodies/c-abl-antibody/2862?srsltid=AfmBOord1H1wfqRdcyGRSY8BL-9t2RY3DGUTA9U4ULdF8l8bkKA6_Vsp

phospho-c-Abl (Y245) (Cell Signaling, #2861S, #9) https://www.cellsignal.com/products/primary-antibodies/phospho-c-abl-tyr245-antibody/2861?srsltid=AfmBOornypcbTREcaj8qbsrilRFWD0ZA1ZeLIpKlpoD0jVNgSxJEIrnZ

STAT5 (D2O6Y) (Cell Signaling, #94205, #5) https://www.cellsignal.com/products/primary-antibodies/stat5-d2o6y-rabbit-monoclonal-antibody/94205?srsltid=AfmBOorxFthjaxkXF8OQfFUysSulFVUOnq3_c6j9eOappQeV5fSrA6GQ

phospho-STAT5A (Y694) (ABclonal, #AP0758, #4000000176) https://abclonal.com/catalog-antibodies/PhosphoSTAT5AY694RabbitmAb/AP0758

CRKL (ABclonal, #A11735. #0030740301) https://abclonal.com/catalog-antibodies/CRKLRabbitpAb/A11735

phospho-CRKL (Y207) (ABclonal, #AP0824, #21156250301). The exact product is no longer available in ABClonal. Validation for the alternative antibody labeled as "phospho-CRKL (Y207) Rabbit pAb (ABclonal, #AP1452) is found here: https://abclonal.com/catalog-antibodies/PhosphoCRKLY207RabbitpAb/AP1452

COXIV (Proteintech, #11242-1-AP, #00163993 and #00110030) https://www.ptglab.com/products/COX4I1-Antibody-11242-1-AP.htm

PMPCB (Proteintech, #16064-1-AP, #00040152) https://www.ptglab.com/products/PMPCB-Antibody-16064-1-AP.htm

β-Actin (8H10D10) (Cell Signaling, #3700S, #21) https://www.cellsignal.com/products/primary-antibodies/beta-actin-8h10d10-mouse-monoclonal-antibody/3700

GAPDH (Proteintech, #60004-1-Ig, #10080731) https://www.ptglab.com/products/GAPDH-Antibody-60004-1-Ig.htm

SDHA (Invitrogen, #459200, #YB3840708) https://www.thermofisher.com/antibody/product/SDHA-Antibody-clone-2E3GC12FB2AE2-Monoclonal/459200

IRDye® 800CW Goat anti-Rabbit Secondary Antibody (Li-Cor Biotechnology, 926-32211, #D50528-07) https://www.licorbio.com/support/contents/reagents/irdye-secondary-antibodies/800cw/goat-anti-rabbit-igg.html

IRDye® 680RD Donkey anti-Mouse Secondary Antibody (Li-Cor Biotechnology, 926-68072, #D41217-05) https://www.licorbio.com/support/contents/reagents/irdye-secondary-antibodies/680rd/donkey-anti-mouse-igg.html

Eukaryotic cell lines

Policy information about cell lines and Sex and Gender in Research

Cell line source(s)	HEK293T (ATCC, CRL-3216), HeLa (ATCC, CCL-2), K562 (ATCC, CCL-243), KCL22 (ATCC, CRL-3349), MOLT4 (ATCC, CRL-1582)
Authentication	Authenticated by ensuring that the features of the cells match the ATCC descriptions (i.e. morphological features, splitting frequency).
Mycoplasma contamination	All cell lines are tested for Mycoplasma contamination monthly. All cell lines used tested negative for mycoplasma contamination.
Commonly misidentified lines (See ICLAC register)	No commonly misidentified cell lines were used in this study.
