# Supplementary material for: Small-molecule binding-site discovery using silyl ether-enabled chemoproteomics
Source: Nat Chem. 2026 Apr 27;18(8):1431–42. doi: 10.1038/s41557-026-02127-4 (PMC13423832; doi:10.1038/s41557-026-02127-4)

Figure 3B_Left Gel

In-gel fluorescence

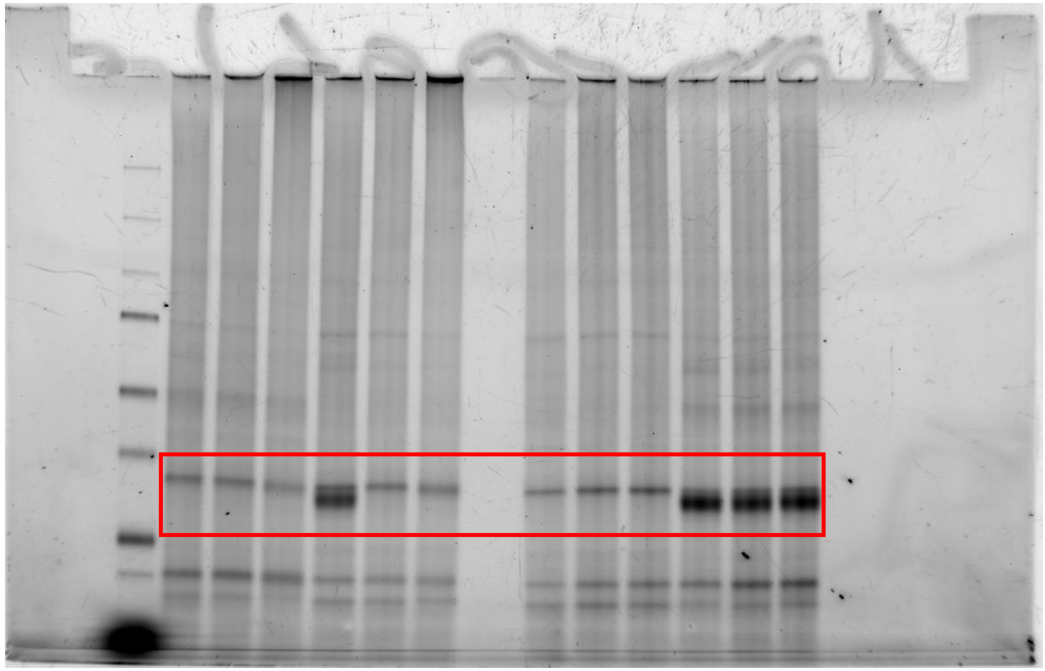

Coomassie

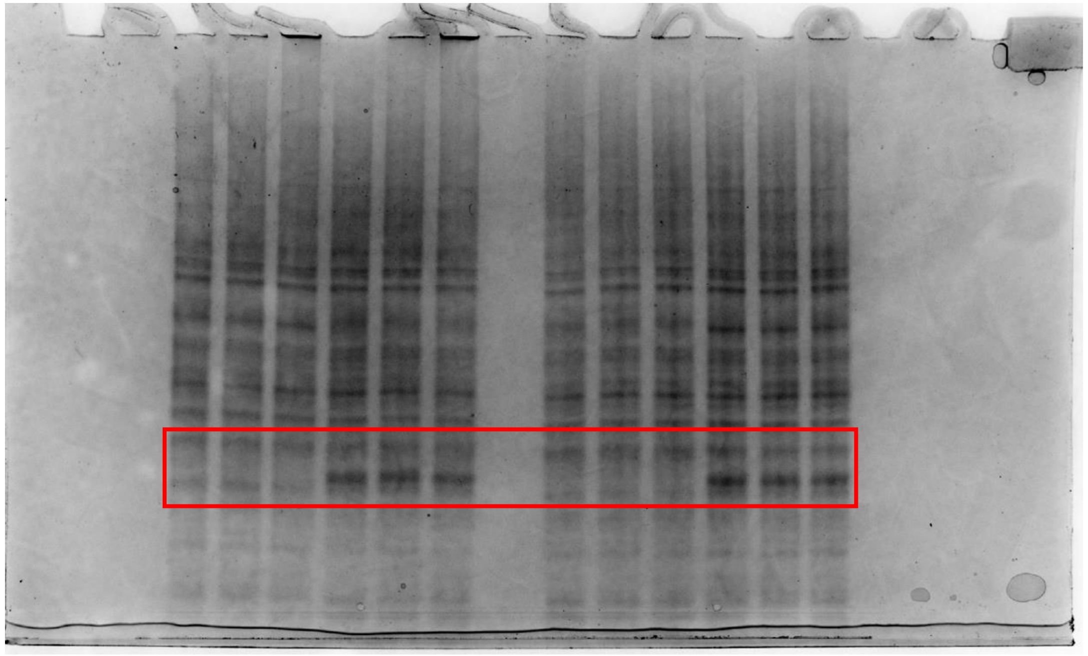

Figure 3B_Right Gel

In-gel fluorescence

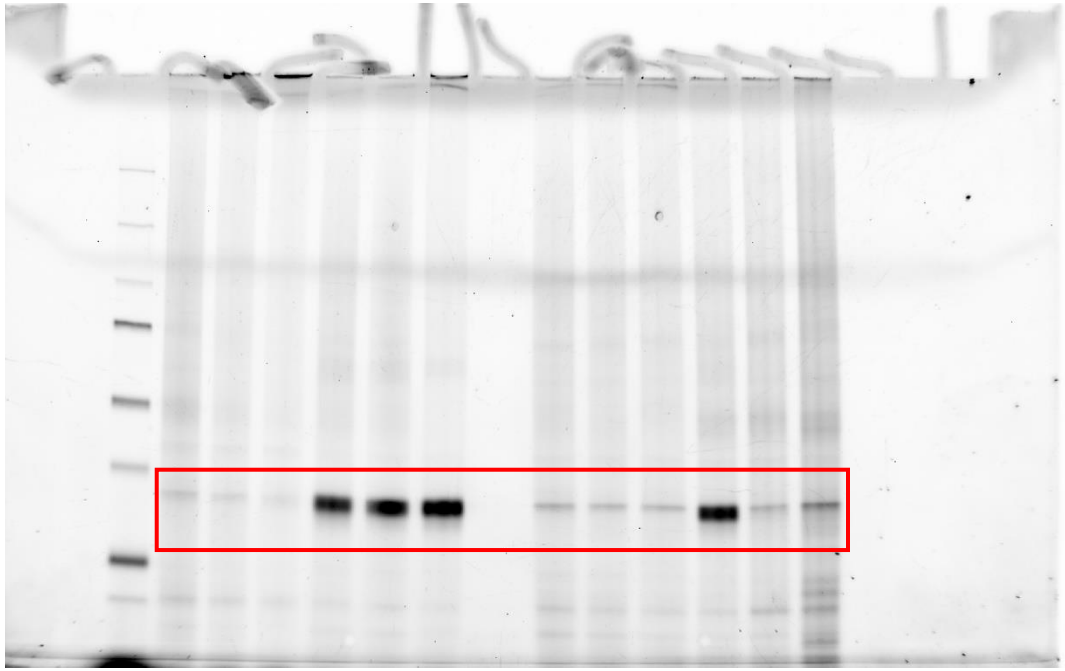

Coomassie

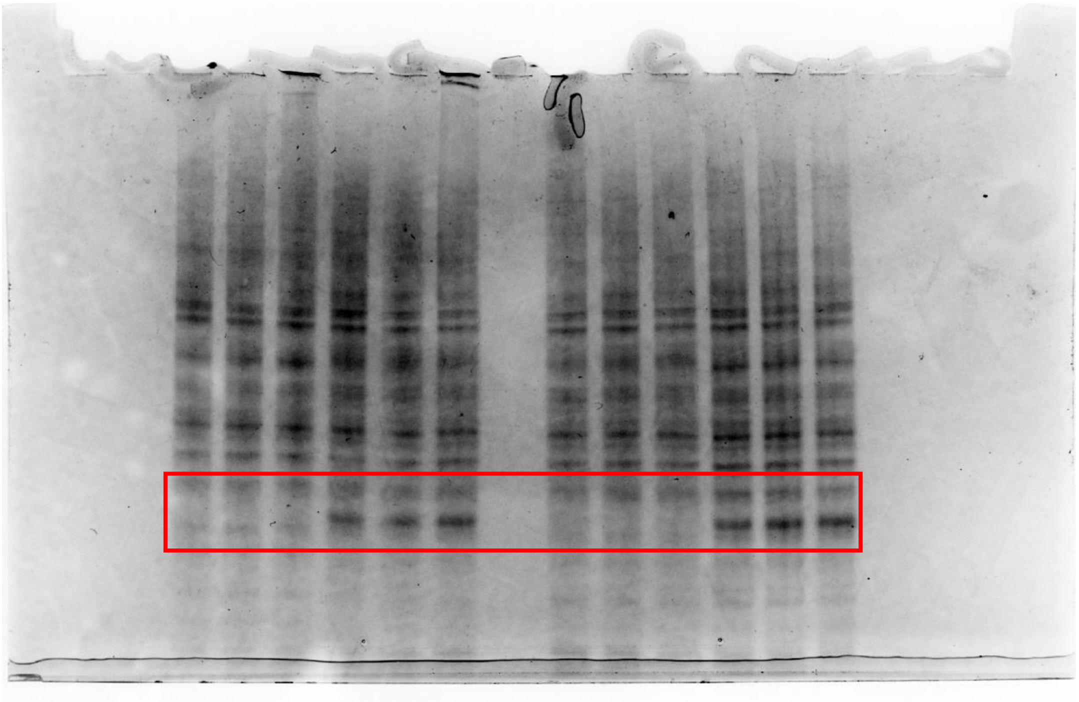

Supplement: Supplementary file 19 — Unprocessed gels. [file 41557_2026_2127_MOESM19_ESM.pdf]
