# Supplementary material for: Small-molecule binding-site discovery using silyl ether-enabled chemoproteomics
Source: Nat Chem. 2026 Apr 27;18(8):1431–42. doi: 10.1038/s41557-026-02127-4 (PMC13423832; doi:10.1038/s41557-026-02127-4)
Supplement: Supplementary file 20 — Unprocessed gels and western blots. [file 41557_2026_2127_MOESM20_ESM.pdf]

Figure 5D

In-gel fluorescence

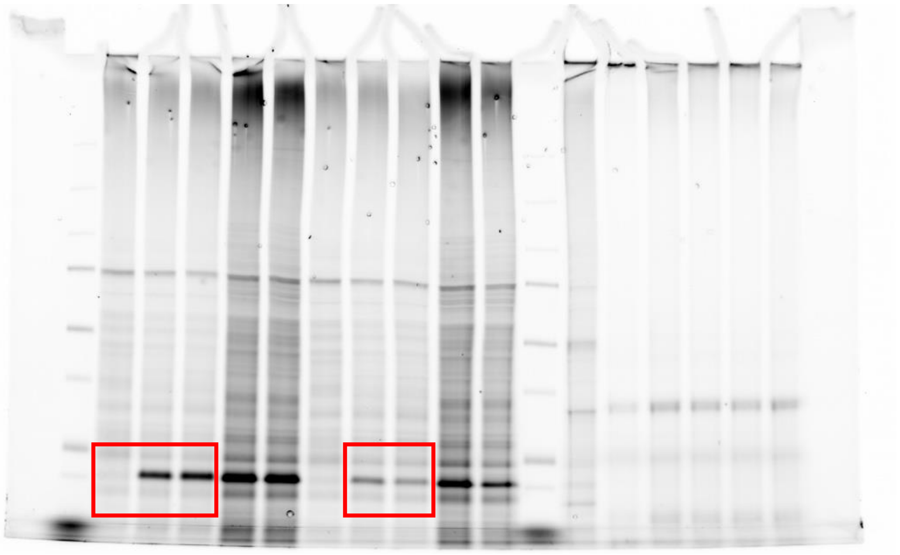

Anti-FLAG

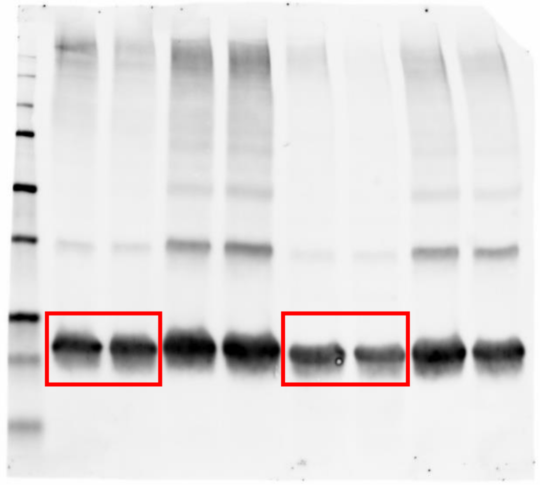

Anti-β-actin

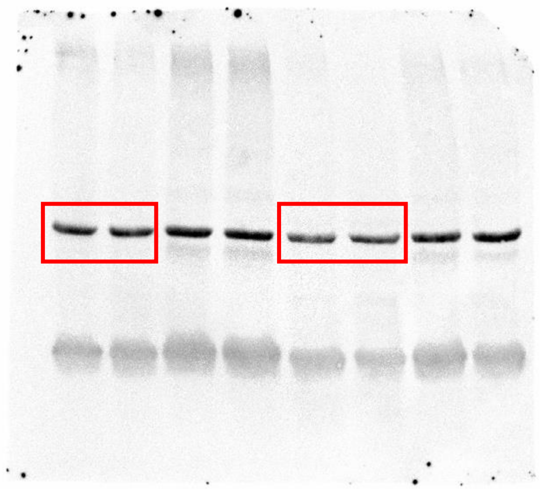

Figure 5E

In-gel fluorescence

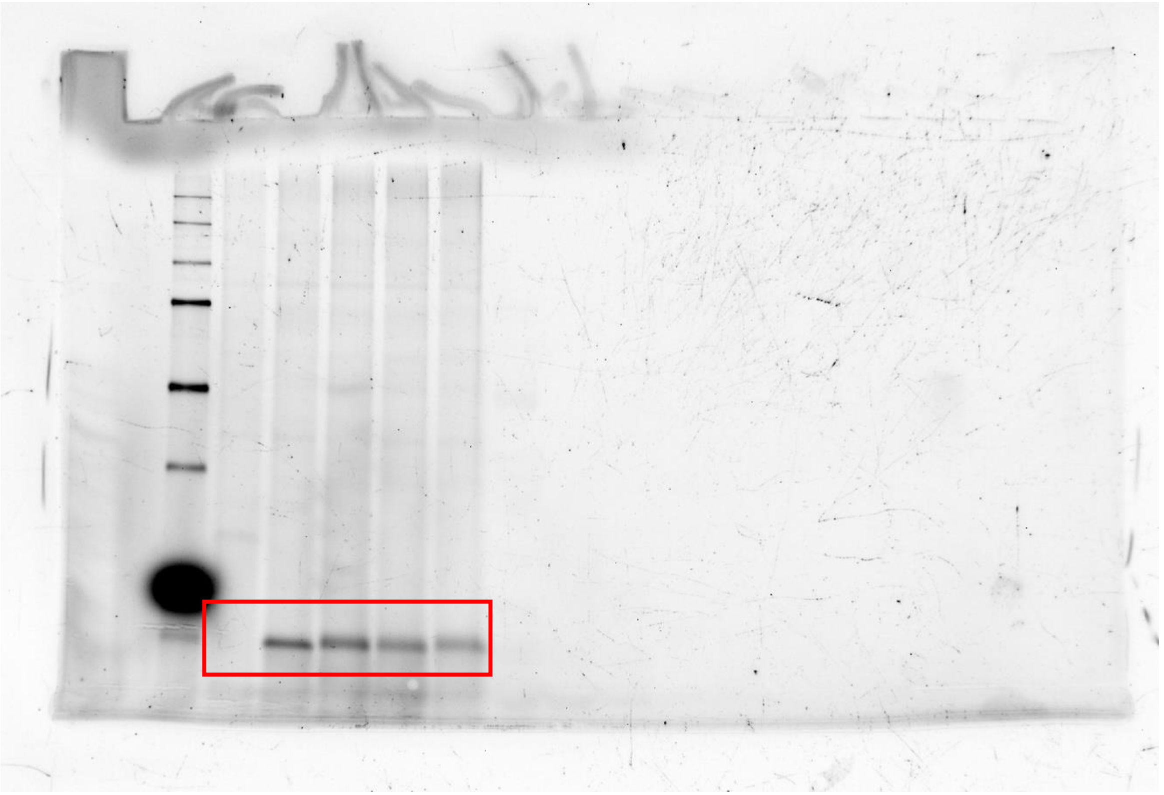

Anti-FLAG

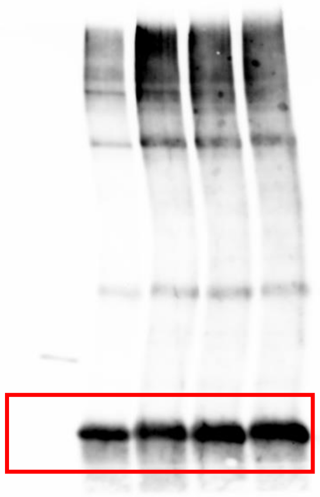

Figure 5J

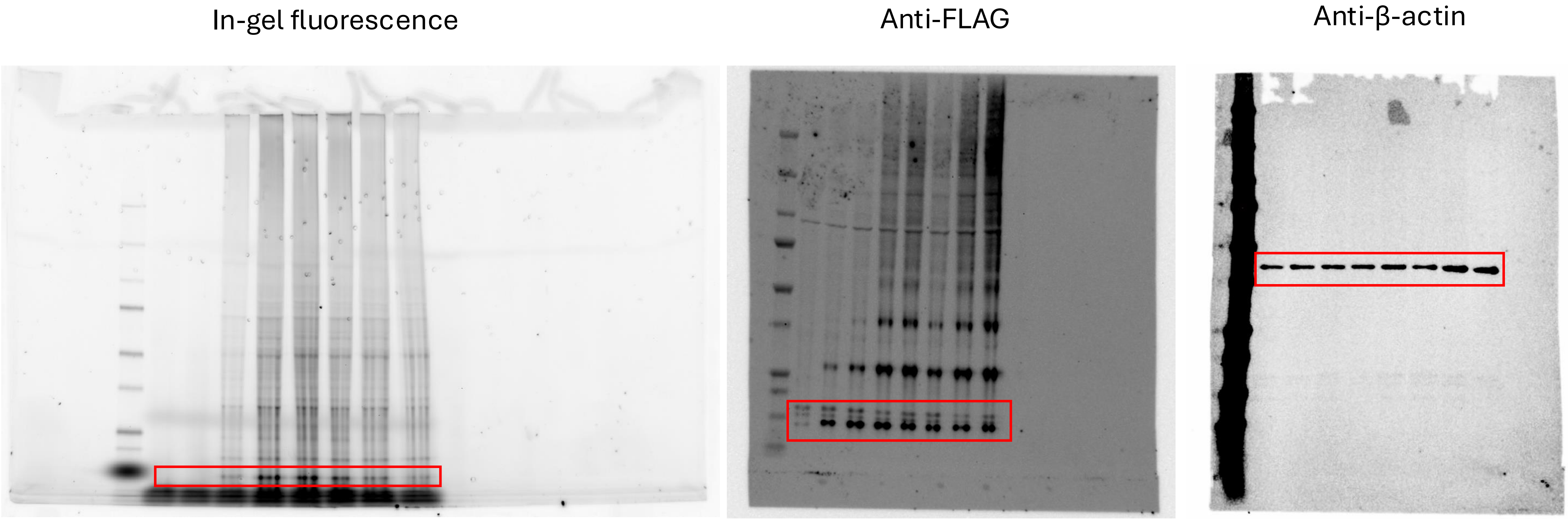

Figure 5K

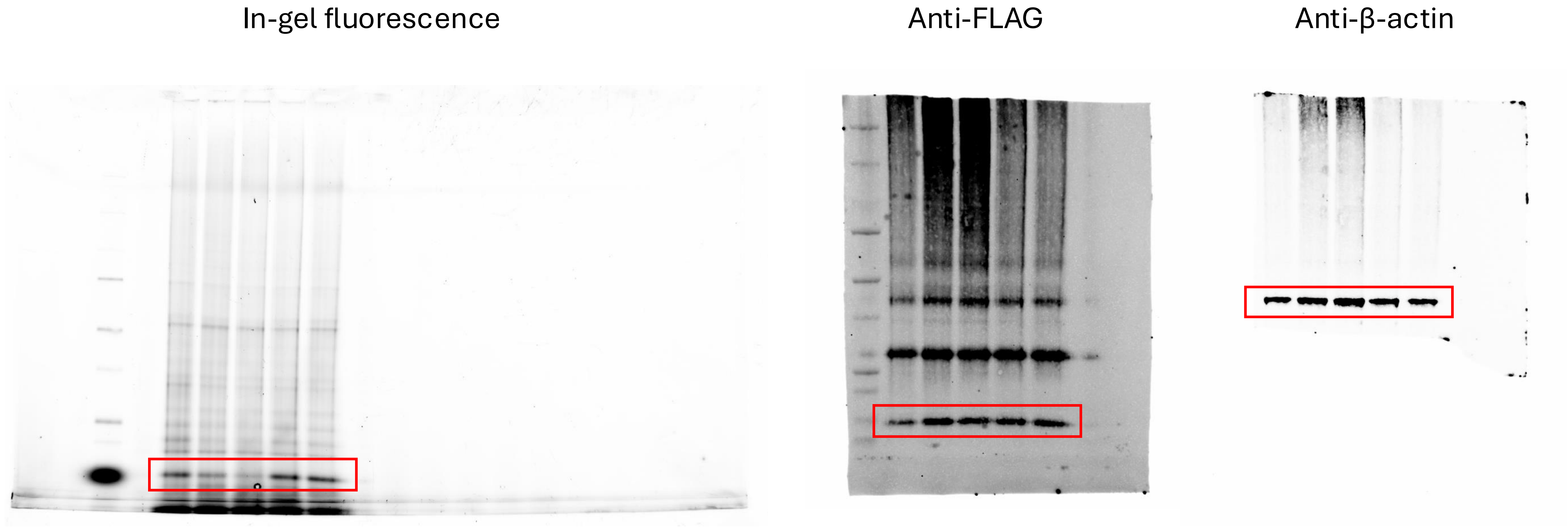

Figure 5L

In-gel fluorescence

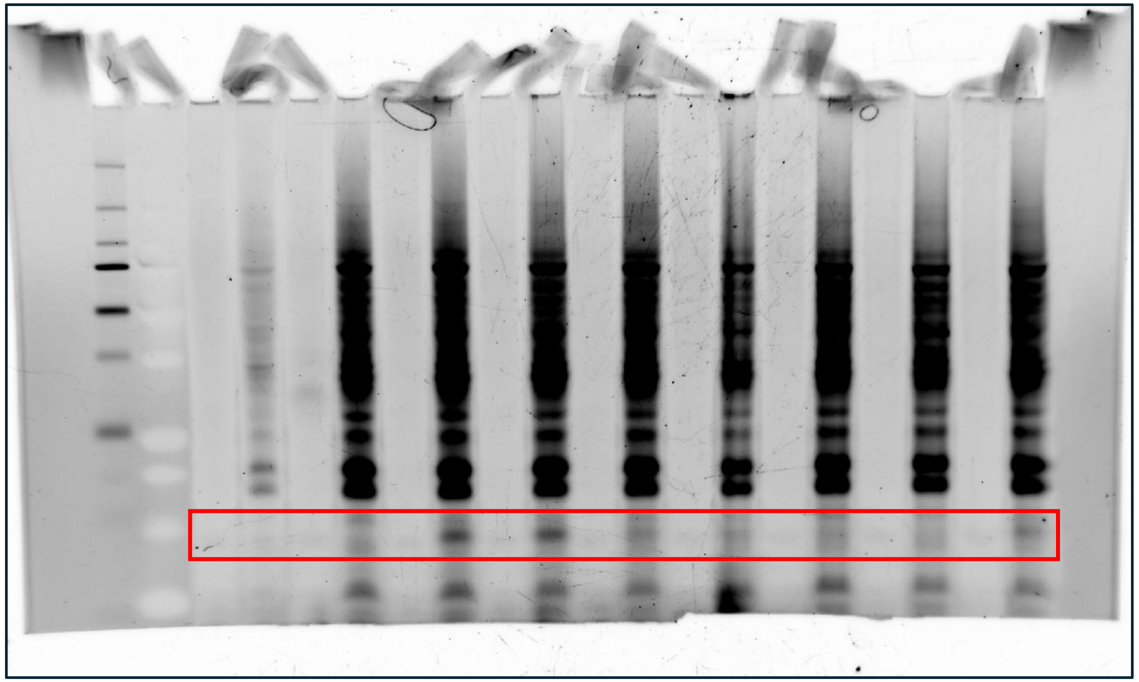

Anti-FLAG (cut blot before scanning)

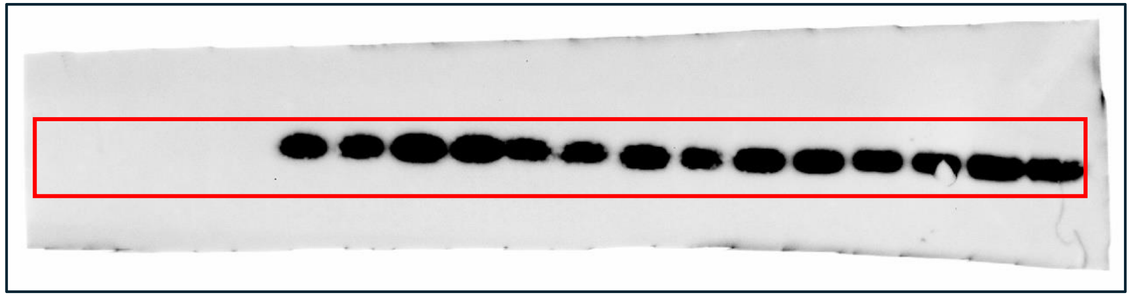

Anti-GAPDH

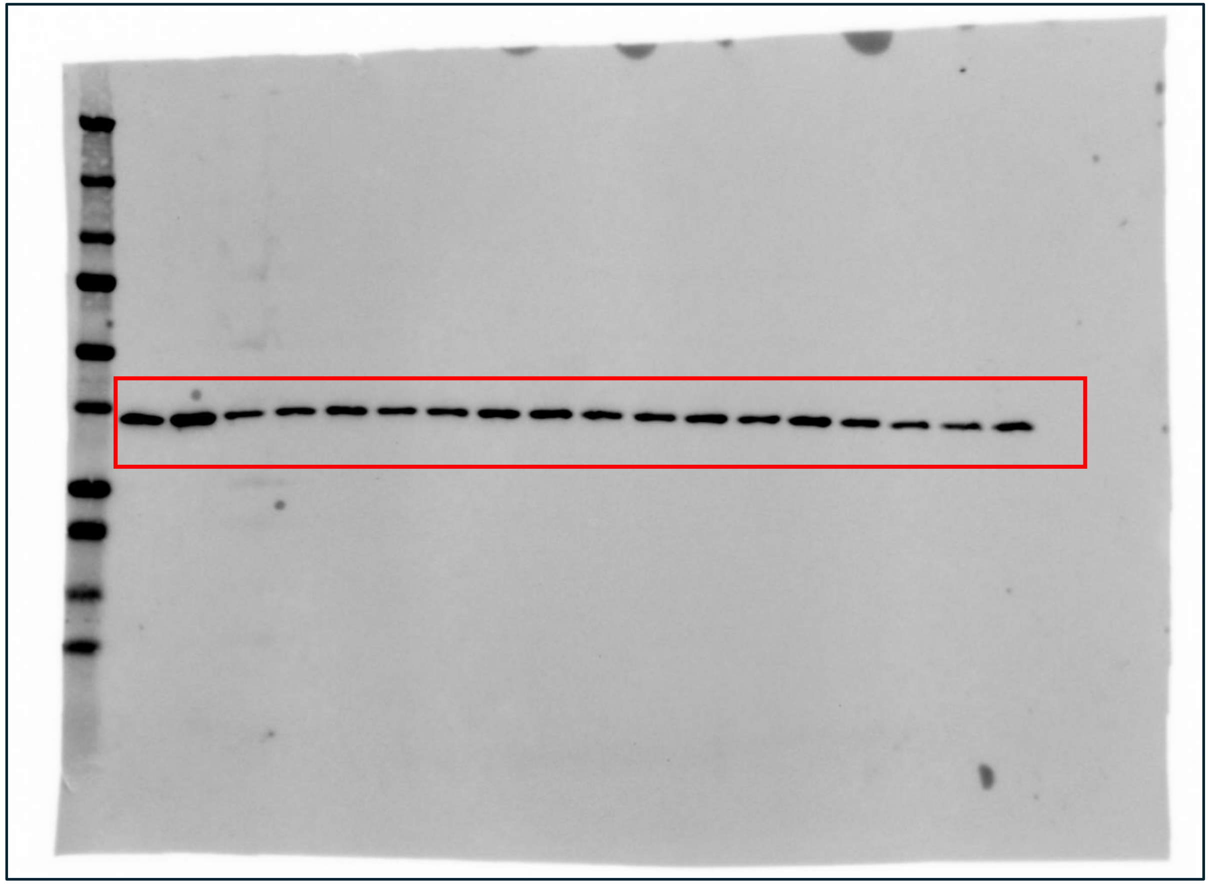

Figure 5M

Anti-FLAG

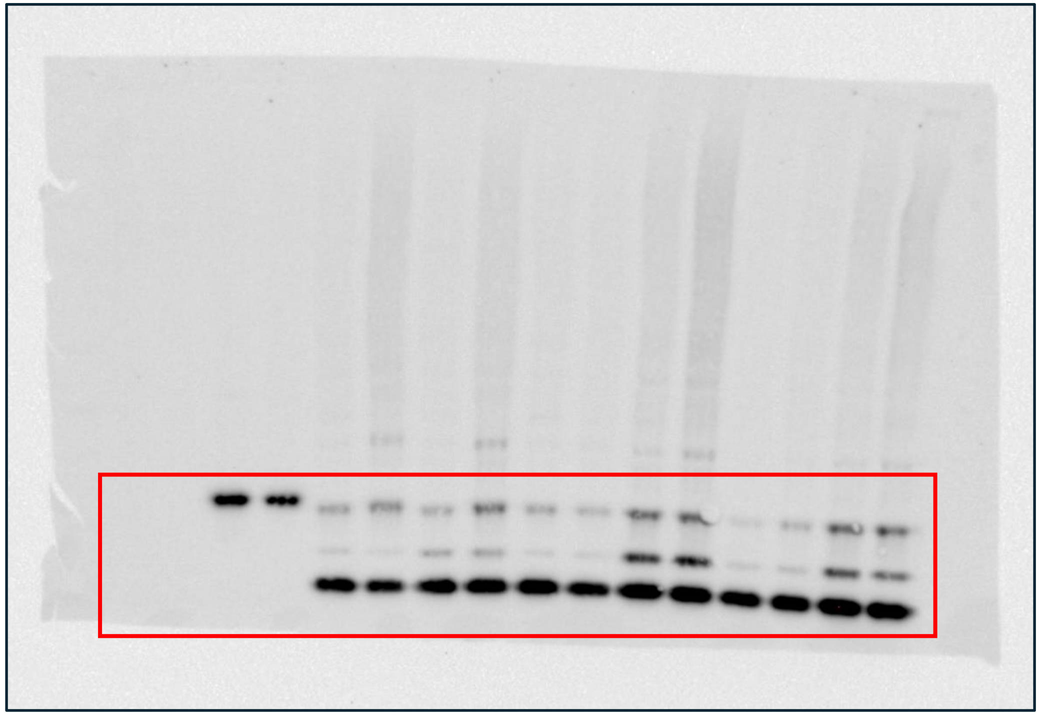

Anti-GAPDH

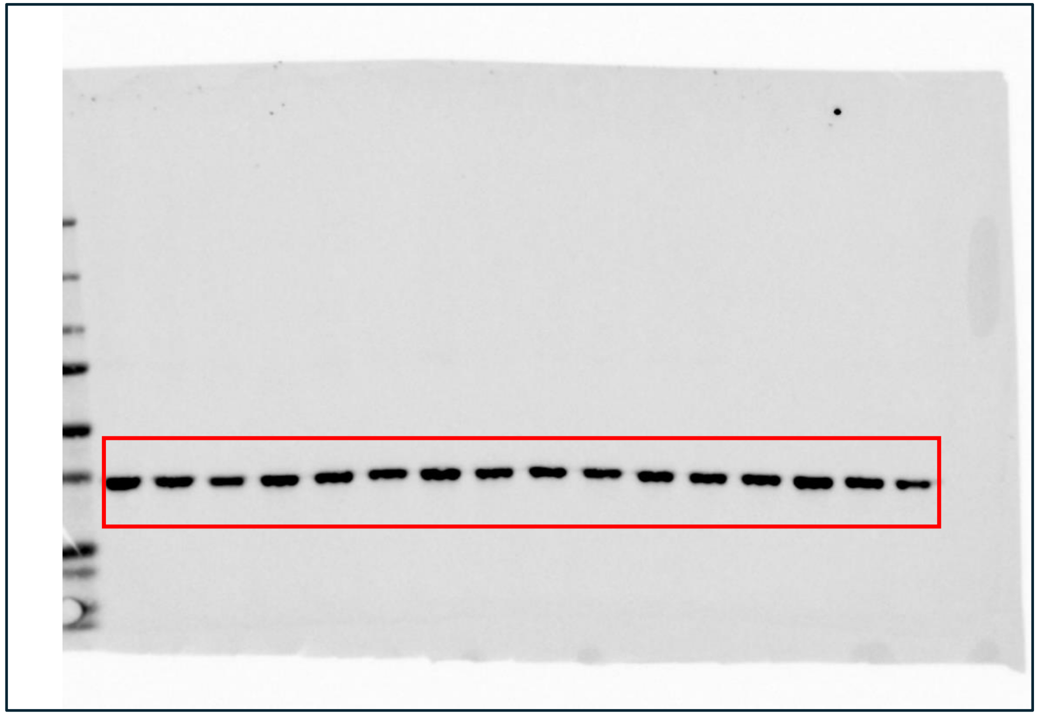

Figure 5N

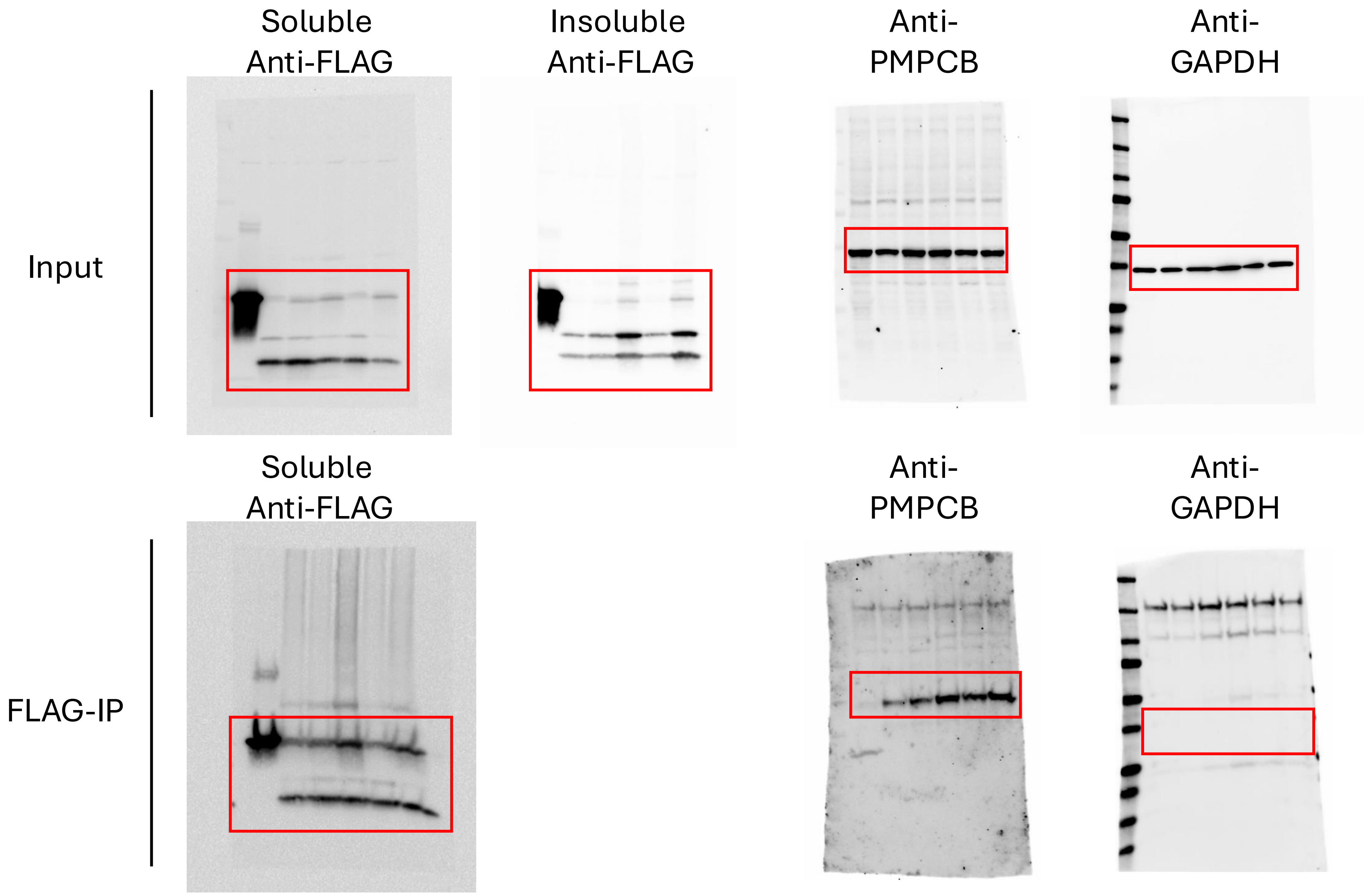
Soluble Anti-FLAG
Insoluble Anti-FLAG
Anti-PMPCB
Anti-GAPDH
Input
Soluble Anti-FLAG
Anti-PMPCB
Anti-GAPDH
FLAG-IP
